# Burnout increased among university students during the COVID-19 pandemic: a systematic review and meta-analysis

**DOI:** 10.1038/s41598-024-52923-6

**Published:** 2024-01-31

**Authors:** Amit Abraham, Karima Chaabna, Javaid I. Sheikh, Ravinder Mamtani, Anupama Jithesh, Salina Khawaja, Sohaila Cheema

**Affiliations:** 1 Institute for Population Health, Weill Cornell Medicine-Qatar, Education City, Qatar Foundation, P.O. Box 24144, Doha, Qatar; 2 Office of the Dean, Weill Cornell Medicine-Qatar, Doha, Qatar

**Keywords:** Public health, Epidemiology, Risk factors

## Abstract

Generally, university students are at risk of burnout. This likely was exacerbated during the COVID-19 pandemic. We aimed to investigate burnout prevalence among university students during the COVID-19 pandemic and examine its distribution across countries, sexes, fields of study, and time-period. PubMed, EMBASE, PsycINFO, World Health Organization’s Global COVID-19 database, Scopus, Epistemonikos, ERIC and Google Scholar were searched (protocol: https://doi.org/10.17605/OSF.IO/BYRXW). Studies were independently screened and extracted. Random-effects meta-analysis was performed. Study quality was appraised, and certainty of evidence was assessed using the Grading of Recommendations Assessment, Development, and Evaluation approach. We identified 44 primary studies comprising 26,500 students. Global prevalence rates were 56.3% for high emotional exhaustion (EE), 55.3% for high cynicism (CY) and 41.8% for low personal accomplishment (PA). Prevalence of EE, CY, and PA domains varied significantly across fields of study, countries and WHO and World Bank regions, but not sex. All studies demonstrated good internal validity, although substantial heterogeneity existed between studies. The certainty of evidence was rated as moderate. Considering its potentially severe consequences, burnout is a significant public health concern. The development and implementation of evidence-based localized interventions at organizational and individual levels are necessary to mitigate burnout.

## Introduction

Burnout is a recognized phenomenon resulting from chronic stress and overwhelming demands. It is characterized by high emotional exhaustion (EE), depersonalization (DP) and lower personal accomplishment (PA)^1,2^.

Burnout among university students occurs due to the academic workload, lack of time, and pressure from family^3,4^, with reported prevalence rates ranging from 7.4 to 71.0% prior to the COVID-19 pandemic^3,4^. The COVID-19 pandemic has exacerbated stressors, with public health measures (e.g., lockdowns, closure of student dormitories, physical distancing and virtual/online learning) significantly disrupting educational experiences and potentially increasing burnout risk^5,6^.

Since the onset of the COVID-19 pandemic, several systematic reviews (SRs) and/or meta-analyses (MAs) on burnout in university students have been published^7–12^. These SRs cover various aspects of the phenomenon, including burnout prevalence in medical students^8,12^, burnout prevalence in university students prior to the pandemic^7,10^, risk and protective factors of university student burnout before and during COVID-19^9^ and burnout prevalence among university students in low- and middle-income countries^11^.

The objectives of this SR/MA are to: (1) determine the burnout prevalence by domain among university students worldwide during the pandemic; (2) investigate patterns across countries, World Health Organization (WHO) regions and World Bank income groups, sexes, fields of study, and time periods; and (3) compile recommendations to guide the development of effective programs, policies, and targeted interventions aimed at addressing burnout among university students.

## Results

### Primary study characteristics

Overall, 5664 records were screened for eligibility, and 44 studies were found to be eligible (Supplementary Fig. S1 and Supplementary Table S5), with 26,500 participants from 31 countries. The MBI and its adapted versions (i.e., aMBI, MBI-SS, MBI-HSS) were the most used instruments (23/44 studies; 52.3%, Supplementary Table S6), followed by the OLBI and its student version (i.e., OLBI-S) (8/44; 18.2%, Supplementary Table S7), the ECE (3/44; 6.8% Supplementary Table S6), and other instruments (Supplementary Tables S8 and S9). Supplementary Table S5 summarizes the characteristics of primary studies.

### Quality assessment

Thirty-seven out of the 44 studies (84.1%) had a high risk of representation bias; 23/44 (52.3%) of the studies did not report the response rate and only 6/44 (13.6%) had a response rate ≥ 75% (Supplementary Table S10). Nearly half of the studies (19/44; 43.2%) invited all eligible participants in the target population; 12/44 (27.3%) used convenience sampling; 11/44 (25.0%) did not report sampling technique, one (2.3%) used cluster sampling and one (2.3%) used stratified-simple sampling. All 44 studies (100%) had low risk of bias (ROB) on the six questions assessing internal validity.

### Overall burnout

A synthesis of the prevalence of overall burnout (OB) can be found in Supplementary Box S3.

### Emotional exhaustion

Twenty-four prevalence measures from 21 countries included in our SR and MA reported the prevalence of students with a risk of high EE (Supplementary Table S6, Table 1, Supplementary Tables S11, S12 and S13). Pooled high EE prevalence was 56.3% worldwide during the COVID-19 pandemic (Table 1). It ranged from 14.2% in the USA to 95.1% in the UAE (Supplementary Table S13). The lowest prevalence was observed in the WHO’s Americas region (29.9%) and the World Bank’s UMIC group (46.7%), while the highest prevalence was observed in the Africa region (86.5%) and LIC (93.1%) group. Subgroup MA on time and geographical patterns revealed that high EE prevalence differed significantly between time periods, countries, and WHO regions and World Bank groups, but not between Western/non-Western countries. Additionally, during the COVID-19 pandemic, high EE prevalence was 43.1% in females and 53.6% in males but this difference was not statistically significant. However, it varied significantly across fields of study with the lowest prevalence observed in psychology students and the highest prevalence in medical students. Students learning via hybrid (a mix of both face-to-face and online) teaching had significantly lower prevalence of high EE than students learning via online teaching only during the COVID-19 pandemic.

**Table 1:** Global pooled prevalence of high emotional exhaustion among university students, measured with the Maslach burnout inventory, the Emotional exhaustion scale, and the Oldenburg burnout inventory.

	Number of prevalence measures	Total sample size	Prevalence range (%)	Effect size		Subgroup comparison (Q between subgroup tests p-value)	Heterogeneity between studies I^2^ (%)
				Weighted average prevalence (%)	95%CI		
World Health Organization region							
Africa	2	650	77.0–93.1	86.5	70.6–94.5	< 0.01	99
Americas	6	1461	12.6–65.7	29.9	15.6–49.5		98
Eastern Mediterranean	4	1578	29.5–95.1	76.3	44.8–92.8		99
Europe	9	2729	21.6–80.0	52.3	37.2–66.9		97
South East Asia	3	3385	31.2–80.6	65.7	37.5–86.0		100
World Bank income group							
HIC	13	4287	14.2–95.1	52.2	35.8–68.1	< 0.01	98
UMIC	6	1481	12.6–85.6	46.7	21.8–73.3		99
LMIC	4	3890	31.2–80.6	68.8	47.1–84.5		100
LIC	1	145	93.1	93.1	87.7–96.6		NA
Western/non-western							
Western	9	2818	14.2–76.4	43.4	28.6–59.4	0.09	98
Non-Western	15	6985	12.6–95.1	63.8	46.1–78.4		99
Sex							
Male	7	986	15.8–74.8	43.1	24.2–64.2	0.53	97
Female	7	2131	10.8–83.2	53.6	29.9–75.7		99
Field							
Medicine	18	8494	14.2–95.1	63.3	48.0–76.3	< 0.01	99
Pharmacy	1	47	44.7	44.7	30.2–59.9		NA
Psychology	1	134	21.6	21.6	15.0–29.6		NA
Mixed fields*	4	1128	12.6–65.7	36.5	18.9–58.6		98
Mode of teaching delivery							
Online	12	4050	14.4–85.6	55.9	40.7–70.0	< 0.01	98
Hybrid	1	254	14.2	14.2	10.1–19.1		NA
NR	11	5499	12.6–95.1	61.4	39.8–79.4		99
Period							
Pre-COVID-19	2	696	74.9–76.4	75.9	72.5–78.9	< 0.01	0
During COVID-19	24	9803	12.6–93.1	56.3	43.0–68.8		99

*EE* emotional exhaustion, *NR* not reported, *NA* not applicable, *HIC* high income countries, *UMIC* upper-middle income countries, *LMIC* lower-middle income countries, *LIC* low income countries, *COVID-19* Corona Virus Disease of 2019.

*Mixed fields include: accounting, administration, biological sciences, engineering, health sciences, law, psychology, Exact Sciences (mathematics, physics, astronomy), political science and social work.

Subgroup MA identified heterogeneity between studies due to the instruments (i.e., MBI, aMBI, OLBI, and ECE) used to assess EE among students (Supplementary S12). No variability was identified between studies reporting and not reporting their response rates or based on data collection methodology (online vs in-person).

### Depersonalization/cynicism

Twenty-four prevalence measures from 21 countries included in our SR reported the prevalence of students with a risk of high DP/CY (Supplementary Table S6). Our MA included 21 studies. Pooled high DP/CY prevalence was 55.3% worldwide during the COVID-19 pandemic (Table 2, Supplementary Table S14). The prevalence of high DP/CY ranged from 3.1% in the USA to 97.2% in Uganda (Supplementary Table S14). The lowest prevalence was observed in the Americas (21.9%) region and the HIC group (37.3%), while the highest prevalence was observed in the Africa (92.8%) region and LIC (97.2%) group. Students from Western countries had lower prevalence of high DP/CY (29.0%) than students from the non-Western countries (73.9%). Subgroup MA on time and geographical patterns revealed that high DP/CY prevalence differed significantly between countries, WHO and World Bank regions, and Western/non-Western countries, but not between time periods. Prevalence of high DP/CY was similar between female students (54.0%) and their male counterparts (54.9%). However, it varied significantly across fields of study with the lowest prevalence observed in ‘all fields’ students and the highest prevalence in medical students. Students experiencing hybrid learning had significantly lower prevalence of high DP/CY than students experiencing solely online learning during the COVID-19 pandemic.

**Table 2:** Global pooled prevalence of high depersonalization/cynicism among university students, measured with the Maslach burnout inventory, the Emotional exhaustion scale and the Oldenburg burnout inventory.

	Number of prevalence measures	Total sample size	Prevalence range (%)	Effect size		Subgroup comparison (Q between subgroup tests p-value)	Heterogeneity between studies I^2^ (%)
				Weighted average prevalence (%)	95%CI		
World Health Organization region							
Africa	2	650	84.6–97.2	92.8	77.3–98.0	< 0.01	92
Americas	3	540	3.1–77.3	21.9	3.0–71.5		99
Eastern Mediterranean	4	1578	33.3–81.3	68.3	48.1–83.3		99
Europe	9	2729	11.0–73.3	40.0	24.2–58.2		98
South East Asia	3	3385	30.7–93.2	77.0	38.7–94.7		100
World Bank income group							
HIC	12	3972	3.1–81.3	37.3	21.2–56.9	< 0.01	98
UMIC	4	875	16.9–77.8	61.8	33.3–84.0		98
LMIC	4	3890	30.7–93.2	79.1	51.5–93.1		100
LIC	1	145	97.2	97.2	93.1–99.2		NA
Western/non-western							
Western	9	2818	3.1–72.1	29.0	14.5–49.5	< 0.01	99
Non-western	12	6064	16.9–97.2	73.9	56.3–86.1		98
Sex							
Male	8	1222	27.4–85.2	54.9	40.3–68.8	0.94	94
Female	8	3325	9.7–85.6	54.0	35.3–71.7		97
Field							
Medicine	18	8494	3.1–97.2	60.5	41.2–77.1	< 0.01	99
Pharmacy	1	47	42.6	42.6	28.3–57.8		NA
Psychology	1	134	20.9	20.9	14.4–28.8		NA
Mixed fields*	1	207	16.9	16.9	12.1–22.7		NA
Mode of teaching delivery							
Online	10	3470	11.0–84.6	53.6	35.2–71.1	< 0.01	98
Hybrid	1	254	3.1	3.1	1.4–6.1		NA
NR	10	5158	16.9–93.2	65.6	39.8–84.6		99
Period							
Pre-COVID-19	2	696	55.6–69.1	62.3	52.5–71.2	0.49	92
During COVID-19	21	8882	3.1–97.2	55.3	37.7–71.6		99

*CY* cynicism, *DP* depersonalization, *NR* not reported, *NA* not applicable, *HIC* high income countries, *UMIC* upper-middle income countries, *LMIC* lower-middle income countries, *LIC* low income countries, *COVID-19* Corona Virus Disease of 2019.

*Mixed fields include biological sciences, health sciences, exact sciences (mathematics, physics, astronomy), and human/social sciences.

Subgroup MA identified heterogeneity between studies due to the instruments (i.e., MBI, aMBI, and OLBI) used to assess DP/CY among students (Supplementary Table S12) and between studies reporting or not reporting their methods of data collection (online vs face-to-face). No variability was identified between studies reporting and not reporting their response rates.

### Personal accomplishment/academic efficacy

Eleven prevalence measures from nine countries included in our SR and MA reported the prevalence of students with a risk of low PA/AE (Supplementary Table S6). Pooled low PA/AE prevalence was 41.8% worldwide during the COVID-19 pandemic (Table 3, Supplementary Table S15). Low PA/AE prevalence ranged from 8.3% in Belgium to 91.7% in Guatemala (Supplementary Table S15). The lowest prevalence was observed in the EMR region (18.0%) and the LIC group (29.0%), while the highest prevalence was observed in the Americas region (80.6%) and UMIC (69.5%) group. Subgroup MA on geographical patterns revealed that low PA/AE prevalence differed significantly between countries, WHO and World Bank regions, but not between Western/non-Western countries. During the COVID-19 pandemic, low PA/AE prevalence was 6.2% in females and 4.2% in males, although this difference was not statistically significant and based on merely two studies. Nevertheless, it significantly varied across fields of study with the lowest prevalence observed in psychology students and the highest prevalence in pharmacy students. Students who learned via hybrid teaching had lower PA/AE prevalence than students learning via online teaching only during the COVID-19 pandemic, but the difference was not statistically significant.

**Table 3:** Global pooled prevalence of low personal accomplishment/academic efficacy among university students, measured with the Maslach burnout inventory, the Emotional exhaustion scale and the Oldenburg burnout inventory.

	Number of prevalence measures	Total sample size	Prevalence range (%)	Effect size		Subgroup comparison (Q between subgroup tests p-value)	Heterogeneity between studies I^2^ (%)
				Weighted average prevalence (%)	95%CI		
World Health Organization region							
Africa	1	145	29.0	29.0	21.7–37.1	< 0.01	NA
Americas	2	386	62.2–91.7	80.6	52.2–94.1		97
Eastern Mediterranean	3	1193	4.4–32.5	18.0	6.0–43.2		98
Europe	4	1689	8.3–79.5	39.4	13.6–72.9		99
South East Asia	1	1947	66.7	66.7	64.6–68.8		NA
World Bank income group							
HIC	7	2712	4.4–79.5	32.6	13.7–59.6	< 0.01	99
UMIC	2	556	32.5–91.7	69.5	20.3–95.3		99
LMIC	1	1947	66.7	66.7	64.6–68.8		NA
LIC	1	145	29.0	29.0	21.7–37.1		NA
Western/non-western							
Western	6	2075	8.3–91.7	54.9	25.7–81.1	0.17	98
Non-western	5	3285	4.4–66.7	27.9	11.8–52.8		99
Sex							
Male	2	465	0.3–39.7	4.2	0.1–70.2	0.86	96
Female	2	1230	1.1–29.5	6.2	0.5–46.4		99
Field							
Medicine	9	5179	4.4–91.7	41.9	19.7–68.0	< 0.01	99
Pharmacy	1	47	59.6	59.6	44.3–73.6		NA
Psychology	1	134	25.4	25.4	18.3–33.6		NA
Mode of teaching delivery							
Online	6	2447	8.3–91.7	51.0	22.9–78.5	0.06	99
Hybrid	1	254	62.2	62.2	55.9–68.2		NA
NR	4	2659	4.4–66.7	25.2	8.2–55.9		99
Period							
During COVID-19^!^	11	5360	4.4–91.7	41.8	22.6–63.9	NA	99

*AE* academic efficacy, *PA* personal accomplishment, *NA* not applicable, *HIC* high income countries, *UMIC* upper-middle income countries, *LMIC* lower-middle income countries, *LIC* low income countries, *COVID-19* Corona Virus Disease of 2019.

^!^No studies reported PA/AE prior to the COVID-19 pandemic.

No heterogeneity was identified between studies due to the variability in the instrument use, differences in their response rates, or means of data collection (Supplementary Table S12).

### Recommendations

Most studies emphasized prevention methods, with some studies recommending treatment-based approaches for burnout management and urging further research on the subject (Table 4 and Supplementary Table S16).

**Table 4:** Summary of recommendations in primary studies.

1. Prevention
Individual-level interventions
Encourage healthy behaviors among students such as eating a healthy and nutritious diet, regular exercise, restful sleep and striking a balance between academic and leisure activities
Promote well-being and foster resilience for stress management utilizing cognitive behavioral therapy, mindfulness, yoga, etc Consider off-site clinic accessibility for students during the pandemic to preserve confidentiality
Organizational-level interventions
Implement interventions to raise awareness and understanding of—and reduce stigma around—psychological distress, burnout, and other mental health illness among students
Educators should consider adapting curricula to reduce workload, increase flexibility and incorporate virtual delivery
Innovations in teaching should be embraced and educators should make efforts to provide a supportive learning environment
Institutions should establish student-led services and encourage senior students’ mentorship programs
Wellness initiatives should be offered to students to overcome any academic, financial, educational and technological barriers
Educators should arrange research opportunities and career counseling to prepare students to join the workforce
2. Early diagnosis and management
Early diagnosis is critical to ensure reduced burden of burnout
Adapt and validate screening tools for local populations
Provide professional support services like counseling and psychological care outside the student campus to avoid embarrassment and stigma associated with accessing these services
Mental health services that are easily accessible, flexible (outside of study hours and during the weekends)
3. Research
Better designed prospective studies to enhance understanding of the prevalence of burnout
Further exploration of contributory factors such as sex, academic pressures, field of study, living situation, financial difficulties and cultural context is necessary to develop and deliver interventions to reduce the prevalence of burnout
Research should assess viability of various interventions such as specific curriculum changes or mindfulness programs
Regularly evaluate applied interventions and make modifications as appropriate

### Reporting bias and certainty of evidence

Our synthesis of the prevalence of burnout among university students during the COVID-19 pandemic was likely influenced by the sparsity of primary research, thereby limiting the worldwide representativeness of our pooled findings. Most of the primary studies had a low ROB related to internal validity, suggesting our pooled estimates were robust; however, they had a high risk of selection bias, which could impact their external validity. Additionally, identified heterogeneity between studies has likely lowered the precision of the pooled prevalence estimates. Therefore, the certainty of available evidence was rated moderate.

## Discussion

This study found that burnout was globally prevalent among university students regardless of sex, field of study, or country during the COVID-19 pandemic, consistent with the literature published prior to the pandemic^13,14^. While socioeconomic and institutional considerations and academic stress were known to contribute to burnout before the pandemic^2^, additional stressors during the pandemic included isolation, difficulty transitioning to online learning and knowing someone affected by COVID-19^15,16^. Wide variability in burnout prevalence was observed across countries. Sociocultural factors and different public health measures implemented in response to the pandemic likely contributed to this variability^11^.

High EE was prevalent, whether measured with the MBI, ECE, or the OLBI. At least half of students across all World Bank groups reported high EE, as did half of students across WHO regions (except for the Americas), and approximately half of both Western and Non-Western countries. As EE can have negative health outcomes, our findings indicate that this aspect of burnout must be addressed among university students worldwide. For instance, the Job Demands-Resources model^17^ indicates more demanding and less flexible schedules are associated with a higher risk of EE^18,19^. Potential interventions to address this include equipping students with a sense of control or autonomy by ensuring curriculum flexibility to accommodate diverse needs and enhance resilience, reducing the focus on stressful exams as a means of evaluation, and adopting hybrid modes of delivery (a mix of face-to-face and online learning)^20^.

High DP/CY was also common among students, whether measured with the MBI, ECE, or the OLBI. Over a third of students in HICs and over 50% of students in other World Bank groups reported high DP/CY. More than 40% of the students reported high DP/CY across WHO regions (except for the Americas), and the prevalence of high DP/CY in non-Western countries was approximately 2.5 times the prevalence reported in Western countries. Future studies, particularly in LICs, are needed to confirm the geographical pattern of high DP/CY prevalence. Studies have shown that individuals who find lower value in their regular routine are at a higher risk of high DP/CY^17–19^. As such, students should view university experiences as meaningful and empowering. This may be achieved by encouraging students to take on leadership and mentorship roles, inculcating a service-learning mindset, and providing community-engagement opportunities^21,22^.

Low PA/AE is also prevalent in university students worldwide: one third of students in HICs and LICs and two thirds of students in UMICs and LMICs have high PA/AE. While the Americas had a lower prevalence of high EE and high DP/CY when compared to other WHO regions, the region had a higher prevalence of low PA/AE. The geographical pattern for low PA/AE needs to be confirmed by further studies. Support and encouragement, together with rewards, both from peers and mentors can reduce the risk of lower PA/AE^18,19^.

The three domains of burnout are not entirely independent, but rather exist along a spectrum^18,19^. Emotional exhaustion and depersonalization are closely interconnected, where individuals who are emotionally exhausted may develop a detached and cynical attitude as a coping mechanism, serving as a means of self-preservation. Personal accomplishment is influenced by both emotional exhaustion and depersonalization. When individuals feel emotionally exhausted and disconnected from their work, their sense of personal accomplishment tends to diminish. Therefore, interventions targeting any one domain of burnout are likely yield positive effects across all domains of burnout^18,19^.

Our results indicate higher prevalence of high EE and DP/CY among non-Western countries, and low PA/AE among Western countries. Possible factors include socio-economic or cultural factors, or a lack of adequate resources or support for students and underdiagnosis^11,23^. However, these differences may also be due to publication bias and lack of burnout data from both regions. This warrants well-designed studies to better describe the geographical patterns of burnout among students.

Our findings demonstrate no statistical difference between male and female students for high EE, high DP/CY or low PA/AE when measured with the MBI, ECE and OLBI. However, the lack of data broken down by sex may have prevented us from identifying significant differences. The literature consistently reports higher burnout among women than men in the adult working population^24,25^. Unequal societal demands and workplace discrimination against women may influence sex differences^26,27^. Further research is needed to determine whether this is also the case among students or if these social determinants contribute to burnout only after students graduate and enter the workforce^26,27^. Studying the local socioeconomic context and cultural milieu may provide further insight into burnout, and institutions must ensure that vulnerable students receive the required support.

Primary studies used several different validated instruments, but also varying definitions of burnout and instrument cut-off values for each burnout domain. This lack of scientific consensus contributes to varying estimates of burnout. The use of different instruments across studies challenges the global and country-level estimation of the prevalence of burnout domains due to heterogeneity. The MBI is the gold standard for burnout diagnosis; however, its use requires a paid license^28^, which probably limits its access in the global south^11^. This is reflected in this SR/MA, where MBI was used predominantly studies undertaken in HICs (13/23; 56.5%), while other instruments were used more often in UMICs/LMICs (13/18, 72.2%). Disparities between the global north and south would be reduced if the MBI were more equitably available to economically disadvantaged countries that currently prefer using freely available tools^11^. This would help reduce the identified methodological heterogeneity between studies due to the variability in the use of instrument and improve data comparability between countries.

Further adding to heterogeneity, some studies use different cut-off values for the same instrument. From a public health perspective, consensus is needed to ensure unified use of instruments and cut-off values. Cut-off values to clinically assess burnout are considered imprecise^29,30^, so burnout assessed as a dichotomous measure (having burnout or not) rather than a spectrum can result in misleading interpretations of burnout diagnoses. However, some agreed cut-offs are necessary to quantify the burden of burnout for the implementation of public health interventions. A unified burnout definition with agreed instrument cut-off values would enhance burden assessment, cross-study comparison, and cost-effective public health interventions.

The trends in prevalence of burnout over time in our SR/MA are hard to interpret. Only two studies included in the MA, which were from different countries, reported pre-COVID-19 data together with data from during the COVID-19 pandemic. Other SRs/MAs in the literature conducted before the pandemic reported the prevalence of burnout domains to be approximately one-third or less of their student population (medical students^8,31,32^ and university students in the UMICs, LMICs and LICs^11^). However, this study reports at least two-thirds of medical students and one-half of students in the UMICs, LMICs and LICs having high EE and DP/CY, and between one- to two-thirds having low PA/AE. Despite the dearth of direct local comparisons, this suggests the prevalence of burnout increased globally during the pandemic. Longitudinal study designs with large sample sizes across multiple universities can help describe the burnout prevalence among students over time.

Our research emphasizes the importance of implementing burnout prevention strategies at the organizational level (Table 4), as organizational change best addresses the root causes of burnout^33,34^. Research assessing interventions to lower burnout in medical students recommend a pass/fail grading system and creating a more positive and interactive environment for student engagement and learning^35^. Seeking support for burnout still remains stigmatized, with individuals being wrongly labelled as weak or incompetent^36^. Educational leadership must raise awareness and provide flexible, stigma-free mental health counselling services, to reduce the burden of burnout^13^. Individual-level interventions, such as mindfulness and healthy lifestyle changes, have also shown promise, albeit to a lesser extent than organizational reform^37,38^. Future research should evaluate how various individual, organizational, and societal stressors, including lived experience, marital status, resilience, and socioeconomic status, can contribute to burnout.

One of this study's main strengths is its extensive search strategy across multiple databases (including gray literature) that allow the application of an a priori protocol to a large range of studies. We included data on students from various disciplines of study, providing a global picture of the evidence on burnout during the pandemic. A weakness of such wide-ranging data is its relatively high degree of heterogeneity, and self-reported data can always include some bias. Several primary studies scored poorly for the external validity component that may affect country-level generalizability. In particular, the variability in the use of instruments and their cut-offs adds to the heterogeneity in the study findings. Finally, publication bias was not assessed because of methodological issues in assessing it for proportion measures.

In summary, this SR/MA provides insight into burnout prevalence among university students globally during the COVID-19 pandemic. The findings highlight the high burnout prevalence across all domains, likely reflecting poor mental health and wellbeing in this population^39,40^. The implications of these findings are significant, as university students will eventually transition into the workforce, carrying the potential long-term consequences of burnout. Our SR/MA identified field of study and mode of teaching delivery (face-to-face/online/hybrid) as significantly associated with prevalence of burnout. Identifying risk factors is crucial for primary prevention of burnout, promoting resilience and developing coping strategies. Pertinent stakeholders including universities and governments must play an active role in prioritizing the mental health of students by fostering student autonomy and enhancing well-being initiatives. Since the three burnout domains exist along a spectrum, it is crucial to understand factors like unhappiness, demotivation and dysfunction that may be contributing to burnout in order to target mitigation and preventive efforts towards such antecedents. Additionally, locally relevant, large-scale, multi-center prospective studies must be conducted using standardized instruments to measure burnout. A consensus from global experts on homogenizing cut-off values for each burnout domain in the instruments is essential to achieve a better understanding of the global burnout burden.

## Methods

This SR/MA was reported according to the Preferred Reporting Items for Systematic Reviews and Meta-Analyses (PRISMA) checklist^41^ (Supplementary Table S1), the PRISMA for Abstracts Checklist^41^ (Supplementary Table S2), the PRISMA checklist for search strategy^42^ (Supplementary Table S3) and Meta-analyses of Observational Studies in Epidemiology (MOOSE) guidelines (Supplementary Table S4)^43^. The protocol was prospectively registered a priori on Open Science Framework (OSF) (available from: 10.17605/OSF.IO/BYRXW). Supplementary Box S1 comprehensively describes the study methodology.

### Eligibility criteria

#### Outcomes and measures

The primary outcome was burnout and/or its domains, and any recommendations by primary studies to address burnout were denoted as secondary outcomes. Studies were included if the outcome was point prevalence of burnout and if they used validated instruments such as the Maslach Burnout Inventory (MBI)^1^, the Maslach Burnout Inventory-Student Survey (MBI-SS)^2^ (with its domains emotional exhaustion (EE), cynicism (CY) and lower academic efficacy (AE)), the abbreviated MBI (aMBI), and the MBI-Human Services Survey (MBI-HSS), the Oldenburg Burnout Inventory (OLBI)^44^, the Copenhagen Burnout Inventory (CBI)^45^, or the Emotional Exhaustion Scale (Escala de Cansancio Emocional, ECE)^46^.

#### Population

We included studies reporting data on university and college students, regardless of field/discipline of study or academic level (undergraduate/graduate/postgraduate). Studies among the general population were included if data on university students as a subgroup was reported. Studies were excluded if students were pursuing vocational studies or if they had graduated.

#### Publication type and study design

We included cross-sectional, longitudinal, and interventional studies. Both gray and non-gray literature sources, like published articles, posters, theses and dissertations, pre-prints, and conference proceedings were eligible for inclusion. Viewpoints / commentaries were included only if they contained original burnout data on university students. Qualitative studies and book chapters were not considered. Systematic reviews were also excluded; however, all identified primary studies from any systematic review that met our eligibility criteria and not previously identified were included.

#### Timing and setting

Only studies with data collected after the onset of the COVID-19 pandemic were included. The WHO declared COVID-19 as a Public Health Emergency of International Concern (PHEIC) on 30 January, 2020 and as a pandemic on 11 March, 2020. On 5 May, 2023, the global emergency caused by the pandemic came to an end, even if transmission was still ongoing^47^. For all countries other than China, the period “during the COVID-19 pandemic” began when the WHO characterized the outbreak as a pandemic in March 2020^47^. However, for China, a public health emergency was declared in January 2020^48^. Thus, we considered the period “during the COVID-19 pandemic” starting in January 2020 for China, as has been done previously^49^.

### Search strategy

A broad literature search related to mental health among university students during the COVID-19 pandemic were conducted on PubMed, EMBASE, PsycINFO, World Health Organization’s Global COVID-19 database, Scopus, and ERIC until May 2021 (Supplementary Box S2). Specific literature searches related to burnout among university students during the COVID-19 pandemic were conducted on Google Scholar and Epistemonikos in May 2022. The literature search update conducted in March 2023 focused on Google Scholar because of its extensive coverage of diverse scholarly articles, including gray literature, preprints and those not yet indexed in traditional databases, making it well-suited for identifying emerging evidence in rapidly evolving topics like the COVID-19 pandemic.

Database selection and the search strategy design were finalized with a librarian. Searched concepts included ‘burnout’, ‘university students’ and ‘COVID-19’ and synonyms (Supplementary Box S2). References of included primary studies were hand-searched independently, and we also reached out to authors of primary studies via email to clarify queries, request missing data and to suggest other studies relevant to our research. The search was not limited by language, geographical area, study design, or publication year.

### Study selection

Two reviewers independently conducted title/abstract and full-text screening on the systematic review software, Rayyan^50^. Included primary studies were restricted to those reported in the languages the authors are fluent in (English, Arabic, French, Spanish, Urdu). Any discrepancies were resolved by discussion.

### Data extraction

Two reviewers independently extracted data using Microsoft Excel, including: (i) study design and setting, (ii) participant demographics, (iii) country, (iv) sample size, (v) time the study was conducted, (vi) outcomes of interest, (vii) instrument used and relevant cut-offs, (viii) recommendations, and (ix) funding and conflicts of interest. Any discrepancies were resolved by discussion.

### Quality assessment

We assessed methodological quality by examining four domains of bias, based on the study by Hoy et al.^51^: (i) selection bias (external validity), (ii) non-response bias (external validity) (iii) measurement bias (internal validity) and (iv) bias related to the analysis (internal validity). Two reviewers independently appraised the studies; no summary score was calculated, as per COSMOS-E guidance^52^. The studies were graded as either high or low risk of bias (ROB). Any disagreements were resolved by discussion.

### Qualitative synthesis

Supplementary Table S5 presents characteristics of primary studies. The list of the excluded studies is provided in eText 1. The ECE (Escala de Cansancio Emocional) is based on the MBI’s EE domain^53^ and the OLBI’s domains of exhaustion and disengagement correspond to the MBI’s EE and CY domains^54^. Therefore, prevalence of high EE and high DP/CY included data measured with MBI, MBI-SS, aMBI, ECE, and OLBI, as relevant.

### Quantitative synthesis

Random-effect MAs were performed when at least two prevalence estimates for the same burnout domain were available. Prevalence was pooled for each burnout domain (EE, DP/CY and PA/AE) and by classification of level of burnout domain (high or not; if not reported, it was considered high). Subgroup MA was conducted by country, World Bank^55^ and World Health Organization regions^56^, Western/non-Western classification, sex, field of study and time period. The Western/non-Western classification was modelled on a previous SR/MA by al Mutairi et al.^8,56^. Statistical significance was set at α = 0.05. To be included in the MA, the minimum study sample size was 25^57^.

Heterogeneity between studies was assessed using the I^2^ statistic^58^ and was considered as substantial when I^2^ > 50%^59^. To explore variability between studies, subgroup MA was conducted considering the primary studies’ response rate and instrument used to assess burnout. The MA was generated using the *meta* package in R software (version 64 4.0.0).

### Reporting bias and certainty assessment

Confidence in the body of evidence on burnout prevalence during the COVID-19 pandemic was assessed by evaluating the validity and reliability of our estimates, based on the Grading of Recommendations Assessment, Development and Evaluation (GRADE) tool^60^.

### Large-scale language model usage statement

ChatGPT was used solely for language touch-ups and to enhance language readability in the Discussion section and did not affect the study's content or methodology.

### Supplementary Information

Supplementary Information.

## Data Availability

Data are available from the corresponding author on reasonable request.
