# Supplementary material for: Burnout increased among university students during the COVID-19 pandemic: a systematic review and meta-analysis
Source: Sci Rep. 2024 Jan 31;14:2569. doi: 10.1038/s41598-024-52923-6 (PMC10831088; doi:10.1038/s41598-024-52923-6)
Supplement: Supplementary file 1 — Supplementary Information. [file 41598_2024_52923_MOESM1_ESM.docx]

**BURNOUT INCREASED AMONG UNIVERSITY STUDENTS DURING THE COVID-19 PANDEMIC: A SYSTEMATIC REVIEW AND META-ANALYSIS**

## Supplementary Table 1: PRISMA 2020 checklist

| **Section and Topic** | **Item #** | **Checklist item** | **Location where item is reported** |
| --- | --- | --- | --- |
| **TITLE** | | |  |
| Title | 1 | Identify the report as a systematic review. | 1 |
| **ABSTRACT** | | |  |
| Abstract | 2 | See the PRISMA 2020 for Abstracts checklist. | 2 |
| **INTRODUCTION** | | |  |
| Rationale | 3 | Describe the rationale for the review in the context of existing knowledge. | 4 |
| Objectives | 4 | Provide an explicit statement of the objective(s) or question(s) the review addresses. | 4 |
| **METHODS** | | |  |
| Eligibility criteria | 5 | Specify the inclusion and exclusion criteria for the review and how studies were grouped for the syntheses. | 5 |
| Information sources | 6 | Specify all databases, registers, websites, organisations, reference lists and other sources searched or consulted to identify studies. Specify the date when each source was last searched or consulted. | 5, Supplementary Box 1 |
| Search strategy | 7 | Present the full search strategies for all databases, registers and websites, including any filters and limits used. | 5, Supplementary Box 1 |
| Selection process | 8 | Specify the methods used to decide whether a study met the inclusion criteria of the review, including how many reviewers screened each record and each report retrieved, whether they worked independently, and if applicable, details of automation tools used in the process. | 6, Supplementary Box 1 |
| Data collection process | 9 | Specify the methods used to collect data from reports, including how many reviewers collected data from each report, whether they worked independently, any processes for obtaining or confirming data from study investigators, and if applicable, details of automation tools used in the process. | 6, Supplementary Box 1 |
| Data items | 10a | List and define all outcomes for which data were sought. Specify whether all results that were compatible with each outcome domain in each study were sought (e.g. for all measures, time points, analyses), and if not, the methods used to decide which results to collect. | 5, Supplementary Box 1 |
|  | 10b | List and define all other variables for which data were sought (e.g. participant and intervention characteristics, funding sources). Describe any assumptions made about any missing or unclear information. | 5-6, Supplementary Box 1 |
| Study risk of bias assessment | 11 | Specify the methods used to assess risk of bias in the included studies, including details of the tool(s) used, how many reviewers assessed each study and whether they worked independently, and if applicable, details of automation tools used in the process. | 6-7, Supplementary Box 1 |
| Effect measures | 12 | Specify for each outcome the effect measure(s) (e.g. risk ratio, mean difference) used in the synthesis or presentation of results. | 6-7, Supplementary Box 1 |
| Synthesis methods | 13a | Describe the processes used to decide which studies were eligible for each synthesis (e.g. tabulating the study intervention characteristics and comparing against the planned groups for each synthesis (item #5)). | 6-7, Supplementary Box 1 |
|  | 13b | Describe any methods required to prepare the data for presentation or synthesis, such as handling of missing summary statistics, or data conversions. | 6-7, Supplementary Box 1 |
|  | 13c | Describe any methods used to tabulate or visually display results of individual studies and syntheses. | 6-7, Supplementary Box 1 |
|  | 13d | Describe any methods used to synthesize results and provide a rationale for the choice(s). If meta-analysis was performed, describe the model(s), method(s) to identify the presence and extent of statistical heterogeneity, and software package(s) used. | 6-7, Supplementary Box 1 |
|  | 13e | Describe any methods used to explore possible causes of heterogeneity among study results (e.g. subgroup analysis, meta-regression). | 7, Supplementary Box 1 |
|  | 13f | Describe any sensitivity analyses conducted to assess robustness of the synthesized results. | 7, Supplementary Box 1 |
| Reporting bias assessment | 14 | Describe any methods used to assess risk of bias due to missing results in a synthesis (arising from reporting biases). | 7, Supplementary Box 1 |
| Certainty assessment | 15 | Describe any methods used to assess certainty (or confidence) in the body of evidence for an outcome. | 7, Supplementary Box 1 |
| **RESULTS** | | |  |
| Study selection | 16a | Describe the results of the search and selection process, from the number of records identified in the search to the number of studies included in the review, ideally using a flow diagram. | 7, Supplementary Figure 1 |
|  | 16b | Cite studies that might appear to meet the inclusion criteria, but which were excluded, and explain why they were excluded. | eText 1 |
| Study characteristics | 17 | Cite each included study and present its characteristics. | Supplementary Table 5 |
| Risk of bias in studies | 18 | Present assessments of risk of bias for each included study. | 7, Supplementary Figure 1, Supplementary Table 10 |
| Results of individual studies | 19 | For all outcomes, present, for each study: (a) summary statistics for each group (where appropriate) and (b) an effect estimate and its precision (e.g. confidence/credible interval), ideally using structured tables or plots. | 7-14, Supplementary Tables 6-9, |
| Results of syntheses | 20a | For each synthesis, briefly summarise the characteristics and risk of bias among contributing studies. | 7-14, Supplementary Table 10 |
|  | 20b | Present results of all statistical syntheses conducted. If meta-analysis was done, present for each the summary estimate and its precision (e.g. confidence/credible interval) and measures of statistical heterogeneity. If comparing groups, describe the direction of the effect. | 7-14, Tables 1-3, Supplementary Tables 13-15 |
|  | 20c | Present results of all investigations of possible causes of heterogeneity among study results. | 7-14, Supplementary Table 12 |
|  | 20d | Present results of all sensitivity analyses conducted to assess the robustness of the synthesized results. | 7-14, Supplementary Table 12 |
| Reporting biases | 21 | Present assessments of risk of bias due to missing results (arising from reporting biases) for each synthesis assessed. | 15-16 |
| Certainty of evidence | 22 | Present assessments of certainty (or confidence) in the body of evidence for each outcome assessed. | 15-16 |
| **DISCUSSION** | | |  |
| Discussion | 23a | Provide a general interpretation of the results in the context of other evidence. | 16-19 |
|  | 23b | Discuss any limitations of the evidence included in the review. | 19 |
|  | 23c | Discuss any limitations of the review processes used. | 19 |
|  | 23d | Discuss implications of the results for practice, policy, and future research. | 16-19 |
| **OTHER INFORMATION** | | |  |
| Registration and protocol | 24a | Provide registration information for the review, including register name and registration number, or state that the review was not registered. | 2, Supplementary Box 1 |
|  | 24b | Indicate where the review protocol can be accessed, or state that a protocol was not prepared. | 2, Supplementary Box 1 |
|  | 24c | Describe and explain any amendments to information provided at registration or in the protocol. | NA |
| Support | 25 | Describe sources of financial or non-financial support for the review, and the role of the funders or sponsors in the review. | 20 |
| Competing interests | 26 | Declare any competing interests of review authors. | 20 |
| Availability of data, code and other materials | 27 | Report which of the following are publicly available and where they can be found: template data collection forms; data extracted from included studies; data used for all analyses; analytic code; any other materials used in the review. | 20 |

*From:*  Page MJ, McKenzie JE, Bossuyt PM, Boutron I, Hoffmann TC, Mulrow CD, et al. The PRISMA 2020 statement: an updated guideline for reporting systematic reviews. BMJ 2021;372:n71. doi: 10.1136/bmj.n71

For more information, visit: <http://www.prisma-statement.org/>

## Supplementary Table 2: PRISMA 2020 for abstracts checklist

| **Section and Topic** | **Item #** | **Checklist item** | **Reported (Yes/No)** |
| --- | --- | --- | --- |
| **TITLE** | | |  |
| Title | 1 | Identify the report as a systematic review. | 2 |
| **BACKGROUND** | | |  |
| Objectives | 2 | Provide an explicit statement of the main objective(s) or question(s) the review addresses. | 2 |
| **METHODS** | | |  |
| Eligibility criteria | 3 | Specify the inclusion and exclusion criteria for the review. | 2 |
| Information sources | 4 | Specify the information sources (e.g. databases, registers) used to identify studies and the date when each was last searched. | 2 |
| Risk of bias | 5 | Specify the methods used to assess risk of bias in the included studies. | 2 |
| Synthesis of results | 6 | Specify the methods used to present and synthesise results. | 2 |
| **RESULTS** | | |  |
| Included studies | 7 | Give the total number of included studies and participants and summarise relevant characteristics of studies. | 2 |
| Synthesis of results | 8 | Present results for main outcomes, preferably indicating the number of included studies and participants for each. If meta-analysis was done, report the summary estimate and confidence/credible interval. If comparing groups, indicate the direction of the effect (i.e. which group is favoured). | 2 |
| **DISCUSSION** | | |  |
| Limitations of evidence | 9 | Provide a brief summary of the limitations of the evidence included in the review (e.g. study risk of bias, inconsistency and imprecision). | 2 |
| Interpretation | 10 | Provide a general interpretation of the results and important implications. | 2 |
| **OTHER** | | |  |
| Funding | 11 | Specify the primary source of funding for the review. | 2 |
| Registration | 12 | Provide the register name and registration number. | 2 |

*From:*  Page MJ, McKenzie JE, Bossuyt PM, Boutron I, Hoffmann TC, Mulrow CD, et al. The PRISMA 2020 statement: an updated guideline for reporting systematic reviews. BMJ 2021;372:n71. doi: 10.1136/bmj.n71

For more information, visit: <http://www.prisma-statement.org/>

## Supplementary Table 3: PRISMA-S 2021 checklist

| **Section/topic** | **#** | **Checklist item** | **Location(s) Reported** |
| --- | --- | --- | --- |
| **INFORMATION SOURCES AND METHODS** | | | |
| Database name | 1 | Name each individual database searched, stating the platform for each. | 5, Supplementary Box 1 |
| Multi-database searching | 2 | If databases were searched simultaneously on a single platform, state the name of the platform, listing all of the databases searched. | NA |
| Study registries | 3 | List any study registries searched. | NA |
| Online resources and browsing | 4 | Describe any online or print source purposefully searched or browsed (e.g., tables of contents, print conference proceedings, web sites), and how this was done. | 5, Supplementary Box 1 |
| Citation searching | 5 | Indicate whether cited references or citing references were examined, and describe any methods used for locating cited/citing references (e.g., browsing reference lists, using a citation index, setting up email alerts for references citing included studies). | Supplementary Box 1 |
| Contacts | 6 | Indicate whether additional studies or data were sought by contacting authors, experts, manufacturers, or others. | Supplementary Box 1 |
| Other methods | 7 | Describe any additional information sources or search methods used. | Supplementary Box 1 |
| **SEARCH STRATEGIES** | | | |
| Full search strategies | 8 | Include the search strategies for each database and information source, copied and pasted exactly as run. | Supplementary Box 2 |
| Limits and restrictions | 9 | Specify that no limits were used, or describe any limits or restrictions applied to a search (e.g., date or time period, language, study design) and provide justification for their use. | 5, Supplementary Boxes 1-2 |
| Search filters | 10 | Indicate whether published search filters were used (as originally designed or modified), and if so, cite the filter(s) used. | 4, Supplementary Boxes 1-2 |
| Prior work | 11 | Indicate when search strategies from other literature reviews were adapted or reused for a substantive part or all of the search, citing the previous review(s). | NA |
| Updates | 12 | Report the methods used to update the search(es) (e.g., rerunning searches, email alerts). | 5,Supplementary Box 1 |
| Dates of searches | 13 | For each search strategy, provide the date when the last search occurred. | 5, Supplementary Box 1 |
| **PEER REVIEW** | | | |
| Peer review | 14 | Describe any search peer review process. | Supplementary Box 1 |
| **MANAGING RECORDS** | | | |
| Total Records | 15 | Document the total number of records identified from each database and other information sources. | Supplementary Figure 1 |
| Deduplication | 16 | Describe the processes and any software used to deduplicate records from multiple database searches and other information sources. | 6, Supplementary Box 1 |
|  |  |  |  |
| PRISMA-S: An Extension to the PRISMA Statement for Reporting Literature Searches in Systematic Reviews | | |  |
| Rethlefsen ML, Kirtley S, Waffenschmidt S, Ayala AP, Moher D, Page MJ, Koffel JB, PRISMA-S Group. | | |  |
| Last updated February 27, 2020. | |  |  |

## Supplementary Table 4: MOOSE Checklist for Meta-analyses of Observational Studies

| **Item No** | **Recommendation** | **Reported on Page No** |
| --- | --- | --- |
| Reporting of background should include | | |
| 1 | Problem definition | 4 |
| 2 | Hypothesis statement | NA |
| 3 | Description of study outcome(s) | 5, Supplementary Box 1 |
| 4 | Type of exposure or intervention used | 5, Supplementary Box 1 |
| 5 | Type of study designs used | 5, Supplementary Box 1 |
| 6 | Study population | 5, Supplementary Box 1 |
| Reporting of search strategy should include | | |
| 7 | Qualifications of searchers (eg, librarians and investigators) | 5, Supplementary Box 1 |
| 8 | Search strategy, including time period included in the synthesis and key words | 5, Supplementary Boxes 1-2 |
| 9 | Effort to include all available studies, including contact with authors | 5, Supplementary Box 1 |
| 10 | Databases and registries searched | 4, Supplementary Box 1 |
| 11 | Search software used, name and version, including special features used (eg, explosion) | 5, Supplementary Box 1 |
| 12 | Use of hand searching (eg, reference lists of obtained articles) | Supplementary Box 1 |
| 13 | List of citations located and those excluded, including justification | Supplementary Figure 1, Supplementary Table 12, eText 1 |
| 14 | Method of addressing articles published in languages other than English | 5, Supplementary Box 1 |
| 15 | Method of handling abstracts and unpublished studies | Supplementary Box 1 |
| 16 | Description of any contact with authors | Supplementary Box 1 |
| Reporting of methods should include | | |
| 17 | Description of relevance or appropriateness of studies assembled for assessing the hypothesis to be tested | 6 |
| 18 | Rationale for the selection and coding of data (eg, sound clinical principles or convenience) | 6 |
| 19 | Documentation of how data were classified and coded (eg, multiple raters, blinding and interrater reliability) | 6 |
| 20 | Assessment of confounding (eg, comparability of cases and controls in studies where appropriate) | NA |
| 21 | Assessment of study quality, including blinding of quality assessors, stratification or regression on possible predictors of study results | 6 |
| 22 | Assessment of heterogeneity | 7 |
| 23 | Description of statistical methods (eg, complete description of fixed or random effects models, justification of whether the chosen models account for predictors of study results, dose-response models, or cumulative meta-analysis) in sufficient detail to be replicated | 6-7 |
| 24 | Provision of appropriate tables and graphics | Tables 1-4  Supplementary Tables 1-12 |
| Reporting of results should include | | |
| 25 | Graphic summarizing individual study estimates and overall estimate | Tables 1-4, Supplementary Tables 5-11, Supplementary Tables 13-15, Supplementary Figure 1 |
| 26 | Table giving descriptive information for each study included | Supplementary Table 5 |
| 27 | Results of sensitivity testing (eg, subgroup analysis) | 8-12, Supplementary Table 12 |
| 28 | Indication of statistical uncertainty of findings | 8-12 |
| Reporting of discussion should include | | |
| 29 | Quantitative assessment of bias (eg, publication bias) | 17-19 |
| 30 | Justification for exclusion (eg, exclusion of non-English language citations) | Supplementary Box 1, Supplementary Table 12, eText 1 |
| 31 | Assessment of quality of included studies | Supplementary Table 10 |
| Reporting of conclusions should include | | |
| 32 | Consideration of alternative explanations for observed results | 16-19 |
| 33 | Generalization of the conclusions (ie, appropriate for the data presented and within the domain of the literature review) | 16-19 |
| 34 | Guidelines for future research | 118-19 |
| 35 | Disclosure of funding source | 20 |

*From*: Stroup DF, Berlin JA, Morton SC, et al, for the Meta-analysis Of Observational Studies in Epidemiology (MOOSE) Group. Meta-analysis of Observational Studies in Epidemiology. A Proposal for Reporting. *JAMA*. 2000;283(15):2008-2012. doi: 10.1001/jama.283.15.2008.

## Supplementary Box 1: Detailed Methods

Protocol and Registration

- Followed the:
  - Cochrane Handbook of Systematic Reviews^1^
  - AMSTAR 2 checklist^2^
- Reported according to the:
  - Preferred Reporting Items for Systematic Reviews and Meta-Analyses (PRISMA) checklist^3^
  - the PRISMA for Abstracts Checklist^3^
  - Meta-analyses of Observational Studies in Epidemiology (MOOSE) guidelines (Supplementary Table 4)^4^
- The protocol was prospectively registered *a priori* on Open Science Framework (OSF)
  - Available from: <https://doi.org/10.17605/OSF.IO/BYRXW>

Eligibility Criteria

Eligibility criteria were determined *a priori* and documented in the protocol.

*Outcomes*

- Primary outcome: point of burnout and/or its domains^5,6^.
  - High EE: Loss of energy and exhaustion due to demands of studies
  - High DP/CY: distant or indifferent attitude towards studies
  - Low PA/AE: generalized poor professional self-esteem and perceived competence
- Secondary outcome: Any recommendations by primary study authors on means to address burnout in this population.

*Measures*

- Included if:
  - Validated instruments were used. These include the Maslach Burnout Inventory (MBI)^5^, the Oldenburg Burnout Inventory (OLBI)^7^, the Copenhagen Burnout Inventory (CBI)^8^, or the Emotional Exhaustion Scale (Escala de Cansancio Emocional, ECE)^9^, amongst others.
  - All versions and adaptations of the MBI were included, such the Maslach Burnout Inventory-Student Survey (MBI-SS)^6^ (with its domains emotional exhaustion (EE), cynicism (CY) and lower academic efficacy (AE)), the abbreviated MBI (aMBI), and the MBI-Human Services Survey (MBI-HSS).

*Population*

- Included university and college students, regardless of:
  - Field/discipline of study
  - Academic level (undergraduates/graduates/postgraduates)
  - Setting (university campuses or quarantine/isolation settings)
  - Studies of general population were included if data on university students as a subgroup were reported.
- Studies were excluded if:
  - Students were pursuing vocational studies
  - Students who had completed graduation (e.g.: interns)
  - Studies that did not have data on student populations separately (e.g.: a combined study population of doctors and medical students)

*Publication Type and Study Design*

- Cross-sectional and longitudinal studies that had data on university students
- Interventional studies were included to extract burnout data among the control group at baseline
- Both gray and non-gray literature sources, such as published articles, theses and dissertations, conference proceedings and pre-prints were considered eligible
- Viewpoints / commentaries were included only if they contained original burnout data in university students.
- Qualitative studies were not considered
- Systematic reviews were also excluded; however, all identified primary studies from any systematic review that met our eligibility criteria and were not previously identified were included

*Timing / Setting*

- Studies utilizing either in-person or online/virtual recruitment were included
- Only studies with data collected after the onset of the COVID-19 pandemic were eligible. These included studies that compared data collected prior to and during the pandemic.
- Primary studies were restricted to the languages that the authors are fluent in (English, Arabic, French, Spanish, Urdu).

Search Strategy

- The search strategy was reported consistent with the PRISMA Statement for Reporting Literature Searches in Systematic Reviews^10^
  - Two authors (AA & KC) systematically searched PubMed (via NLM), EMBASE (via OVID), PsycINFO (via EBSCO), World Health Organization’s Global COVID-19 database^11^, Scopus (via Elsevier) and ERIC (via OVID) from inception until May 31, 2021.
  - First 10 pages of Google Scholar^12^ and Epistemonikos^13^ were searched on May 18-19, 2022 to identify gray and non-gray literature.
  - A final updated Google Scholar search was conducted on March 14, 2023.
- Search strategy was broad and included synonyms of ‘mental health’, university students, and COVID-19
- The strategy comprised both controlled vocabulary and free-text terms that were tailored to each database (Supplementary Box 2)
- The WHO Global COVID-19 Research Database, Google Scholar, and Epistemonikos allow only keyword searches.
- Database selection and the search strategy were conducted in consultation with a senior librarian from Weill Cornell Medicine-Qatar.
- The literature search was not limited to any language, geographical area, study design or year of publication.
- Bibliographies of all included primary studies for any relevant studies not otherwise identified were searched.
- We also reached out to the corresponding authors of primary studies via email to clarify queries, request missing data and to suggest other relevant studies.

Study Selection

- AA and AJ removed duplicates using the online systematic review software, Rayyan^14^
- AA and AJ independently conducted title and abstract screening.
- Full-text screening was independently conducted by two reviewers (divided between AA, AJ, and JA)
- Studies in French and Spanish were independently double screened by KC and SK
- Exclusion reasons were recorded at each step.
- Discrepancies at both stages were resolved with a third reviewer (KC) under supervision of the senior authors (SC and RM)
- Any study including data on mental health, but not having any data on burnout, or were in languages other than English, Arabic, French, Spanish or Urdu were excluded.
- Attempts were made to ensure that none of the primary studies were retracted by examining the journal and database web pages, and the Retraction Watch website^15^.

Data Extraction

- After piloting on a small study sample, data extraction was conducted independently by two reviewers (divided between AA, AJ, and SK)
- A standardized table to extract relevant information was developed using Microsoft Excel. Extracted information includes:

1. study design and setting
2. country
3. participant demographics
4. sample size
5. time the study was conducted
6. outcomes of interest
7. instrument used and relevant cut-offs
8. recommendations
9. funding and conflicts of interest.

- If there were multiple publications from the same study (e.g.: one poster and one publication), the more comprehensive study was prioritized.
- A consensus meeting was held between AA, AJ, SK and KC to resolve any disagreement, under supervision of the senior authors (SC and RM).

Quality Assessment

- The methodological quality was assessed using the quality assessment instrument developed by Hoy *et al.* for prevalence studies^16^
- This instrument comprises 10 items addressing four domains of bias, in addition to a summary risk of bias assessment. The four domains evaluate the presence of: (i) selection bias (external validity), (ii) non-response bias (external validity) (iii) measurement bias (internal validity) and (iv) bias related to the analysis (internal validity)
- AA and AJ independently appraised the studies for presence of risk of bias and no summary score was calculated, as per COSMOS-E guidance^17^.
- Studies that did not provide information regarding any of the above-mentioned domains (selection and non-response bias) were designated as having a high risk of bias, as recommended by Hoy et al^16^.
- Any disagreement was resolved by discussion with KC.

Qualitative Synthesis

- We narratively synthesized the findings from all the included primary studies (Supplementary Table 5).
- The list of the excluded studies at the end of full text screening is provided in eText 1.
- ECE is based on the MBI’s EE domain^9,18,19^. and the OLBI’s domains of exhaustion and disengagement domains correspond to the EE and CYcomponents of the MBI^20^. Therefore, prevalence of having high EE included data measured with MBI, MBI-SS, aMBI, OLBI and ECE, while the prevalence of having high DP/CY included data measured with MBI, MBI-SS, aMBI and OLBI.
- If prevalence of overall burnout (as defined by the authors of primary studies) or its domains was not provided but the individual data was available, prevalence was calculated by one author (SK) and checked by a second (AA/KC).
- We classified the reported recommendations into three categories: (i) prevention, (ii) early diagnosis and management, and (iii) research.

Recommendations

- We also provide a summary of recommendations to manage burnout among university students by the authors of the primary studies (Supplementary Table 15)
- Most studies prioritized preventive techniques at the individual level, with some studies proposing treatment-based approaches to managing burnout, and urging further research in the field. A summary of these recommendations can be found in Table 4.

Quantitative Synthesis

- Meta-analyses were performed when at least two prevalence estimates for the same burnout domain were available.
- Pooled prevalence was estimated during the COVID-19 pandemic by country, World Bank income group^21^, World Health Organization geographical region^22^, Western / non-western classification, sex, and field of study. The Western/non-western classification was modelled on a previous SR/MA by al Mutairi *et al.*^23^.
- For each burnout domain (described below), pooled prevalence and subgroup analysis by period (before and during the COVID-19 pandemic) was also conducted.
  - EE (included in the MBI, its adapted versions, OLBI and ECE)
  - DP/CY (included in the MBI, its adapted versions and OLBI)
  - PA/AE included in the MBI and its adapted versions
- Statistical significance was set at α=0.05
- Clopper-Pearson confidence intervals were computed for individual prevalence measures.
- Prevalence measures and their 95% confidence intervals (95% CI) were pooled based on the random-effects model with the logit transformation of the proportion.
- To be included in the meta-analysis, there was a minimum study sample size of 25^24^.
- Heterogeneity between studies was assessed using the I^2^ statistic, which describes the percentage of variability across studies that is due to true heterogeneity rather than chance^25^.
- Heterogeneity between studies was considered as substantial when I^2^ > 50%^1^.
- To explore variability between studies, subgroup meta-analysis was conducted considering primary studies’ response rate and instrument used to assess burnout.
- Meta-analysis and forest plots were generated using the meta package in R software (version 64 4.0.0).

Reporting bias and certainty assessment

- Confidence in the body of evidence related to burnout prevalence during the COVID-19 pandemic was assessed by evaluating the validity and reliability of our estimates, based on the Grading of Recommendations Assessment, Development and Evaluation (GRADE) tool^26^
- The GRADE tool evaluates the quality and risk of bias in a body of evidence, precision of the meta-analysis effect estimates, the consistency of the primary study results, and how the body of evidence answers the research question.

## Supplementary Box 2: Search Strategy

**PubMed**

Date of search: May 31, 2021

Number of Results: 2151

No filters

(("education" or "training") and (adult or youth)) OR (("university" or "college" or "undergraduate" OR "graduate" OR "postgraduate") and ("students" OR "Students"[Mesh]))

AND

((((((((((((((((((((((“Betacoronavirus”[MeSH Terms] OR “Coronavirus Infections”[MeSH Terms]) OR “COVID-19”[Supplementary Concept]) OR “Coronavirus”[MeSH Terms]) OR “Severe Acute Respiratory Syndrome Coronavirus 2”[Supplementary Concept]) OR “2019nCoV”[All Fields]) OR “betacoronavirus*”[All Fields]) OR “corona virus*”[All Fields]) OR “coronavirus*”[All Fields]) OR “coronovirus*”[All Fields]) OR “CoV”[All Fields]) OR “CoV2”[All Fields]) OR “COVID”[All Fields]) OR ((“COVID-19”[Supplementary Concept] OR “COVID-19”[All Fields]) OR “covid19”[All Fields])) OR (((((((“COVID-19”[All Fields] OR “covid 2019”[All Fields]) OR “Severe Acute Respiratory Syndrome Coronavirus 2”[Supplementary Concept]) OR “Severe Acute Respiratory Syndrome Coronavirus 2”[All Fields]) OR “2019 ncov”[All Fields]) OR “SARS CoV 2”[All Fields]) OR “2019nCoV”[All Fields]) OR ((“wuhan”[All Fields] AND (“Coronavirus”[MeSH Terms] OR “Coronavirus”[All Fields])) AND (2019/12/1:2019/12/31[Date - Publication] OR 2020/1/1:2020/12/31[Date - Publication])))) OR “HCoV-19”[All Fields]) OR “nCoV”[All Fields]) OR “SARS CoV 2”[All Fields]) OR “SARS2”[All Fields]) OR “SARSCoV”[All Fields]) OR ((((“sars virus”[MeSH Terms] OR (“sars”[All Fields] AND “virus”[All Fields])) OR “sars virus”[All Fields]) OR (“sars”[All Fields] AND “CoV”[All Fields])) OR “sars cov”[All Fields])) OR ((“Severe Acute Respiratory Syndrome Coronavirus 2”[Supplementary Concept] OR “Severe Acute Respiratory Syndrome Coronavirus 2”[All Fields]) OR “SARS CoV 2”[All Fields])) OR “severe acute respiratory syndrome cov*”[All Fields]) AND (2019/11/17:3000/12/31[Date - Entry] OR 2019/11/17:3000/12/31[Date - Publication])

AND

("Mental Health"[MeSH Terms] or "mental disorders"[MeSH Terms] OR ("mental"[All Fields] AND "health"[All Fields]) OR "mental health"[All Fields] OR "Burnout, Psychological"[Mesh] OR Burn-out OR "Burn out" OR Burnout OR "Caregiver Exhaustion" OR "personalisation" OR

"personalization" OR "personal accomplishment" OR "emotional exhaustion" OR "Anxiety"[Mesh] OR Hypervigilance OR Nervousness OR Anxiet* OR Phobia* OR Panic OR "overanxious disorder" OR obsessive OR OCD OR obsessi* OR compulsi* OR "depression"[Mesh] OR "depressive Disorder"[Mesh] OR "Affective Disorders, Psychotic"[Mesh] OR depress* OR Melancholi* OR Dysthymi* OR "Reactive Disorder" OR "Reactive Disorder" OR "Adjustment Disorder" OR "Affective Psychoses" OR "Affective Psychosis" OR "Psychotic Affective Disorder" OR "Psychotic Mood Disorder" OR "Psychotic Mood Disorder" OR "Psychotic Affective Disorder" OR "Stress, Psychological"[Mesh] OR "Adaptation, Psychological"[Mesh] OR Coping OR "Psychologic Adaptation" OR "Psychologic Adaptations" OR Adaptive Behavior OR Adaptive Behaviors OR Stress* OR "Mental Suffering" OR Anguish OR "Compassion Fatigue" OR "Suicide"[Mesh] OR "Self-Injurious Behavior"[Mesh] OR "self mutilation"[MeSH Terms] OR Suicid* OR self harm OR Self injury OR Self destructive OR self inflict* OR self mutilat*)

**EMBASE**

Date of search: May 31, 2021

Number of Results: 199

Restrict to COVID-19 and exclude Medline journals

college student/ or graduate student/ or exp health student/ or non-medical student/ or phd student/ or postgraduate student/ or undergraduate student/ or university student/ or ((university or college or undergraduate or postgraduate or graduate or education or training) and students).mp.

AND

exp depression/ or depress*.mp. or bipolar disorder?.mp. or Adjustment Disorder?.mp. or dysphoria.mp. or dysthymia.mp. or melancholia.mp. or mourning syndrome.mp. or exp affective psychosis/ or exp bipolar disorder/ or Melancholi*.mp. or Dysthymi*.mp. or Reactive Disorder?.mp. or Transient Situational Disturbance.mp. or exp anxiety disorder/ or anxiety.mp. or exp anxiety/ or Hypervigilance.mp. or Anxiety Disorder?.mp. or Nervousness.mp. or jitteriness.mp. or nervosity.mp. or neurosis.mp. or phobic.mp. or Anxieties.mp. or Phobia?.mp. or Panic.mp. or overanxious disorder.mp. or OCD.mp. or obsessi*.mp. or compulsi*.mp. or fear.mp. or exp suicidal behavior/ or Suicid*.mp. or self harm.mp. or Parasuicid*.mp. or Self Injurious.mp. or Self Injury.mp. or Self Injuries.mp. or Self destructive.mp. or self inflict.mp. or self mutilat*.mp. or self wounding.mp. or exp stress/ or Stress.mp. or Coping.mp. or exp coping behavior/ or Psychologic Adaptation?.mp. or Psychological Adaptation?.mp. or Adaptive Behavior?.mp. or Stress*.mp. or Mental Suffering.mp. or Mental Stress.mp. or Anguish.mp. or Compassion Fatigue.mp. or alarm reaction.mp. or mental tension.mp. or psychic tension.mp.

**PsycINFO**

Date of search: May 31, 2021

Number of Results: 422

No results

“Mental Health” OR "Major Depression" OR "Anaclitic depression" OR "Dysthymic Disorder" OR "Endogenous depression" OR "Late Life depression" OR "Postpartum depression" OR "Reactive depression" OR "Recurrent depression" OR "Treatment Resistant depression" OR "depression (Emotion)" OR "Bipolar Disorder " OR "Cyclothymic Disorder OR "Affective Disorder " OR "Disruptive Mood Dysregulation Disorder " OR "Major depression" OR "Seasonal Affective Disorder" OR "Adjustment Disorder " OR "Affective Disorder" OR "Major depression" OR "Seasonal Affective Disorder" OR depressi* OR Dysthymi* OR Melanchol* OR "Adjustment Disorder" OR coping OR cope OR cyclothymi* OR dysphor* OR "Reactive Disorder" OR "Mood dysregulation" OR "mood disorder" OR "mood disorder" OR Affective psychos?s OR "Occupational Stress" OR "Compassion Fatigue" OR "Compassion fatigue" OR "burn out" OR "Caregiver Exhaustion" OR “personalization” OR “personalization” OR "personal accomplishment" OR "emotional exhaustion" OR "Anxiety" OR "Anxiety Sensitivity" OR "Anxiety Disorder" OR "Generalized Anxiety Disorder" OR "Obsessive Compulsive Disorder" OR "Panic Attack" OR "Panic Disorder" OR "Phobias" OR Emotion* OR Agitat* OR Fear* OR Guilt* OR panic OR phobi* OR shame OR angst OR worry OR apprehensi* OR Hypochondria* OR hypervigilan* OR anxiet* OR anxious* OR overanxi* OR OCD OR obsessive OR compulsive OR obsession OR compulsion OR "Caregiver Burden" OR "Financial Strain" OR "Adjustment Disorder" OR "Posttraumatic Stress Disorder" OR "Compassion Fatigue" OR Stress* OR "adjustment Disorder" OR adversity OR coping OR "compassion fatigue" OR "emotional control" OR helpless* OR resilien* OR "Psychologic Adaptation" OR "Adaptive behavior" OR "Adaptive behaviors" OR "Adaptive behaviour" OR "Adaptive behaviours" OR "Psychologic Adaptations" OR suffer* OR "Suicide" OR "Suicidality" OR "Suicidology" OR "Self Inflicted" OR Self Injurious OR Self Inflict* OR Self Mutilat* OR Self Poison* OR self harm OR self injury OR self destructive

AND

covid-19 or coronavirus or 2019-ncov or sars-cov-2 or cov-19

AND

students or college students or higher education or further education or university students or DE "Business Students" OR DE "College Graduates" OR DE "College Students" OR DE "College Athletes" OR DE "Community College Students" OR DE "Education Students" OR DE "Junior College Students" OR DE "Nursing Students" OR DE "ROTC Students" OR DE "Dental Students" OR DE "Elementary School Students" OR DE "Intermediate School Students" OR DE "Primary School Students" OR DE "Graduate Students" OR DE "Law Students" OR DE "Medical Students" OR DE "Postgraduate Students"

**SCOPUS**

Date of search: May 31, 2021

Number of Results: 1818

No filters

( "Mental Health" OR "Major depression" OR depress* OR stress OR "Compassion Fatigue" OR burnout OR "Caregiver Exhaustion" OR personalization OR "personal accomplishment" OR "emotional exhaustion" OR "Anxiety" OR "Phobias" OR gad OR emotion* OR agitat* OR fear* OR guilt* OR "Internalizing symptom" OR panic OR phobi* OR obsessive OR compulsive OR obsession OR compulsion OR stress OR "Adjustment Disorder" OR coping OR suicid* OR "self-harm" OR "self-injury" OR self-mutilat* OR self-inflict* ) AND ( covid-19 OR coronavirus OR 2019-ncov OR sars-cov-2 OR cov-19 OR severe AND acute AND respiratory AND syndrome AND coronavirus 2 OR ( "wuhan" AND "coronavirus" ) ) AND ( ("university" or "college" or “graduate” or “undergraduate” or “postgraduate”) and ("student") )

**WHO Global COVID Database (**[**https://search.bvsalud.org/global-literature-on-novel-coronavirus-2019-ncov/advanced/?lang=en**](https://search.bvsalud.org/global-literature-on-novel-coronavirus-2019-ncov/advanced/?lang=en)**)**

Date of search: May 31, 2021

Number of Results: 831

Exclude Pubmed, SCOPUS, EMBASE and PsycInfo results

(tw:(Mental Health or depression or anxiety or stress or suicide or burnout)) AND (tw:(university or college or students or graduate or undergraduate or postgraduate))

**ERIC (OVID)**

Date of search: May 31, 2021

Number of Results: 176

No filters

college student/ or graduate student/ or exp health student/ or non-medical student/ or phd student/ or postgraduate student/ or undergraduate student/ or university student/ or ((university or college or undergraduate or postgraduate or graduate or education or training) and students).mp.

AND

exp depression/ or depress*.mp. or bipolar disorder?.mp. or Adjustment Disorder?.mp. or dysphoria.mp. or dysthymia.mp. or melancholia.mp. or mourning syndrome.mp. or exp affective psychosis/ or exp bipolar disorder/ or Melancholi*.mp. or Dysthymi*.mp. or Reactive Disorder?.mp. or Transient Situational Disturbance.mp. or exp anxiety disorder/ or anxiety.mp. or exp anxiety/ or Hypervigilance.mp. or Anxiety Disorder?.mp. or Nervousness.mp. or jitteriness.mp. or nervosity.mp. or neurosis.mp. or phobic.mp. or Anxieties.mp. or Phobia?.mp. or Panic.mp. or overanxious disorder.mp. or OCD.mp. or obsessi*.mp. or compulsi*.mp. or fear.mp. or exp suicidal behavior/ or Suicid*.mp. or self harm.mp. or Parasuicid*.mp. or Self Injurious.mp. or Self Injury.mp. or Self Injuries.mp. or Self destructive.mp. or self inflict.mp. or self mutilat*.mp. or self wounding.mp. or exp stress/ or Stress.mp. or Coping.mp. or exp coping behavior/ or Psychologic Adaptation?.mp. or Psychological Adaptation?.mp. or Adaptive Behavior?.mp. or Stress*.mp. or Mental Suffering.mp. or Mental Stress.mp. or Anguish.mp. or Compassion Fatigue.mp. or alarm reaction.mp. or mental tension.mp. or psychic tension.mp.

AND

exp COVID-19/ or (coronavirus or "corona virus" or "corona pandemic" or coronavirinae or coronaviridae or betacoronavirus or covid19 or covid or nCoV or "CoV 2" or CoV2 or sars2 or sarscov2 or 2019nCoV or "novel CoV").mp.

**Epistominokos**

Date of search: May 19, 2022

Number of Results: 5

Search 1:

Burnout AND university students AND COVID-19

Filter: Primary studies only

Up to the first 10 pages were searched

Search 2: Burnout AND university students AND COVID-19

Filter: Systematic reviews only

Up to the first 10 pages were searched

Search 3: Burnout AND college students AND COVID-19

Filter: Primary studies only

Up to the first 10 pages were searched

Search 4: Burnout AND college students AND COVID-19

Filter: Systematic reviews only

Up to the first 10 pages were searched

**Google Scholar**

Date of search: May 18-19, 2022; March 14, 2023

Number of Results: 13; 9

Search 1:

Burnout AND university students AND COVID-19

The first 10 pages were searched

Search 2: Burnout AND college students AND COVID-19

The first 10 pages were searched

**Supplementary Table 5: Characteristics of primary studies**

| **Study No** | **Citation** | **Study Period** | **Country** | **Response rate (%)** | **Study**  **Design** | **Sampling Technique** | **Sample**  **size** | **Age (yrs) Mean (SD) Range** | **Sex n (%)** | **Field of study** | **Year of study** | **Instrument Used** |
| --- | --- | --- | --- | --- | --- | --- | --- | --- | --- | --- | --- | --- |
| 1 | Abdel Aziz et al., 2023^27^ | April – June 2022 | United Arab Emirates | 68.5 | CS | All students  were invited | 385 | 20.5 (2.38) | M: 77 (20%) F: 308 (80%) | Medicine | Y1-Y6 | OLBI |
| 2 | Aguayo-Estremera et al., 2023^28^ | Quarter 1, 2021 (unspecified) | Spaun | NR | CS | Non-  probabilistic | 134 | 18 (NR) | M: 49 (16%) F: 85 (84%) | Psychology | Y1 | MBI-SS |
| 3 | Alqifari et al., 2021^29^ | March 2-23, 2020 | Saudi Arabia | 36.6 | CS | NR | 336 | NR  18-34 | M: 146 (43.5) F: 190 (56.5) | Medicine | Y1-Y6 | MBI-SS |
| 4 | Alsaad et al., 2021^30^ | July – September 2020 | Saudi Arabia | NR | CS | NR | 439 | 21.6 (6.8) 18-32 | M: 120 (27.3)  F: 319 (72.7) | All fields | Y1-Y6 | MBI-HSS |
| 5 | Ayinde et al., 2022^31^ | May – August 2020 | Nigeria | NR | CS | Cluster  sampling | 505 | NR | M: 285 (56.4%) F: 219 (43.4%) Other : 1 (0.2%) | Medicine | Y1-Y6 | OLBI |
| 6 | Azzi et al, 2022a^32^ | August 7-26, 2020 | Brazil | NR | CS | Snowball (convenience) | 703 | 23.7 (3.7) (F) 24.6 (4.7) (M) 17-62 | M: 200 (28.5) F: 503 (71.6) | All fields | NR | BCSQ-12-SS |
| 7 | Azzi et al, 2022b^33^ | February 2-21, 2020;  September 21- October 17, 2020 | Brazil | NR | LS | NR | 63 | 23.2 (NR) 18-48 | M: 17 (26.9) F: 46 (73) | All fields | NR | MBI-SS |
| 8a**** | Bolatov et al., 2021^34^ | October – November 2019 | Kazakhstan | NR | LS | NR | 619 | 19.14 (NR) 17-27 | M: 155 (25.04) F: 464 (74.96) | Medicine | Y1-Y5 | CBI-S |
| 8b**** | Bolatov et al., 2021^34^ | April 13-19, 2020 | Kazakhstan | NR | LS | NR | 798 | 20.31 (NR)  17-33 | M: 194 (24.3)  F: 604 (76.7) | Medicine | Y1-Y5 | CBI-S |
| 9 | Chi et al, 2021^35^ | August 19 – September 15, 2020 | USA | 35.5 | CS | All students  were invited | 109 | NR | M: 43 (41.4) F: 61 (58.7) Other: 3 (2.8) Missing: 2 (1.8) | Dentistry | Y1-Y4 | PWS |
| 10 | Chumakov et al., 2021^36^ | May - June 2020 | Russia | 43.2 | CS | All students  were invited | 165 | NR | M: 33 (20.0) F: 132 (80.0) | Medicine | Y1-Y6 | OLBI |
| 11 | Cipta et al., 2022^37^ | April – May 2021 | Indonesia | 19.47 | CS | All students  were invited | 1947 | Median: 19  Range: 14-23 | M: 480 (24.7%) F: 1467 (75.3%) | Medicine | Y1-Y4 | MBI-SS |
| 12 | Dahanayake et al., 2022^38^ | July – August 2020 | Sri Lanka | NR | CS | All students  were invited | 1097 | NR | M: 353 (32.2%) F: 744 (67.8%) | Medicine | Y1-Y6 | OLBI |
| 13 | Dlugosz et al., 2021^39^ | June 1-10, 2020 | Poland | NR | CS | Convenience sampling | 1870 | NR | M: 321 (16.8) F: 1593 (83.2) | All fields | Y1-Y5 | LBQ |
| 14 | El Mouedden et al., 2022^40^ | April 22 – May 4, 2020 | Belgium | NR | CS | Convenience sampling | 145 | 24.95 (2.53)  NR | M: 40 (20.5) F: 154 (79.5) | Medicine | Y4-Y6 | MBI-SS |
| **Study No** | **Citation** | **Study Period** | **Country** | **Response rate (%)** | **Study Design** | **Sampling Technique** | **Sample**  **size** | **Age (yrs Mean (SD) Range** | **Sex n (%)** | **Field of study** | **Year of study** | **Instrument Used** |
| 15 | Estrada Araoz, 2021^41^ | June 2021 | Peru | NR | CS | NR | 265 | NR  ≥16 | M: 117 (44.2) F: 148 (55.8) | All fields | NR | ECE |
| 16 | Forycka et al., 2022^42^ | January 5 – February 6, 2021 | Poland | NR | CS | All students  were invited | 1858 | NR  ≥18 | M: 319 (23.4) F: 1040 (76.3) Other: 4 (0.29) | Medicine | Y1-Y6 | MBI-SS |
| 17 | Jezzini-Martinez et al, 2022^43^ | June 22-26, 2020 | Mexico | 36.4 | CS | All students  were invited | 154 | 18.9 (0.9)  18-24 | M: 61 (39.6) F: 93 (60.4) | Medicine | Y1 | MBI-SS |
| 18 | Johns et al., 2022^44^ | September 27 2020 – January 31 2021 | UK | NR | CS | All students  were invited | 82 | NR | NR | Medicine | Final Year | aMBI |
| 19 | Kajjimu et al., 2021^45^ | September 6-20, 2020 | Uganda | 35.5 | CS | All students  were invited | 145 | NR  18-40 | M: 102 (70.3) F: 43 (29.7) | Medicine | Y1-Y5 | MBI-SS |
| 20 | Khalafallah et al., 2021^46^ | 2 weeks in May 2020 | USA | 14.5 | CS | All students  were invited | 254 | NR  20–40 | M: 140 (55.1) F: 114 (44.9) | Medicine | Y1-Final year; Research or Elective year | aMBI |
| 21 | Kjær et al., 2022^47^ | February 21 – August 17, 2020 | Denmark | NR | CS | All students  were invited | 647 | NR | M: 148 (22.9%)  F: 496 (76.6%) Other: 2 (0.3%)  NR: 1 | Medicine | Y1-Y6 | OLBI |
| 22 | Martinez-Libano et al., 2021^19^ | November 20 | Chile | NR | CS | NR | 315 | NR  18-59 | M: 68 (21.6) F: 247 (78.4) | All fields | Y1-Y5 | ECE |
| 23 | Moreno-Fernandez et al., 2020^48^ | During confienement period | Spain | NR | CS | Convenience sampling | 47 | 20 (2.1) (M) 20 (1.8) (F)  NR | M: 19 (40.42) F: 28 (59.58) | Pharmacy | Y1 | MBI-SS |
| 24 | Muaddi et al., 2023^49^ | October – November 2020 | Saudi Arabia | 46 | CS | All students  were invited | 433 | NR | M: 226 (50.9%) F: 218 (49.1%) | Medicine | Y2-Y6 | MBI-SS |
| 25 | Nurhidayati et al., 2021^50^ | June 20 | Indonesia | NR | CS | Purposive sampling | 83 | NR  17-22 | M: 6 (7.22) F: 77 (92.77) | Nursing | Undergraduate | Burnout scale* |
| 26 | Periasamy et al., 2021^51^ | June 21 | India | 36.8 | CS | All students  were invited | 154 | 19.7 (1.26) 17-24 | M: 63 (40.9) F: 91 (59.1) | Medicine | Y1-Y4 | MBI-SS |
| 27 | Philip et al., 2021^52^ | February 9 – August 31, 2020 | India | NR | CS | All students  were invited | 344 | NR | M: 135 (39.0) F: 208 (60.0) Other: 1 (1.0) | Medicine | Y1-Y6 | OLBI |
| 28 | Ramos et al., 2021^53^ | 3 weeks during the COVID-19 pandemic | Philippines | NR | CS | NR | 245 | NR  17-35 | M: 67 (27.4) F: 178 (72.7) | All fields | Y1-Y4 | de novo tool** |
| **Study No** | **Citation** | **Study Period** | **Country** | **Response rate (%)** | **Study Design** | **Sampling Technique** | **Sample**  **size** | **Age (yrs Mean (SD) Range** | **Sex n (%)** | **Field of study** | **Year of study** | **Instrument Used** |
| 29 | Remitha et al., 2020^54^ | June - August 2020 | Indonesia | 35.71 | CS | All students  were invited | 175 | NR | M: 63 (36.0) F: 112 (64.0) | Medicine | Y1-Y2 | Burnout scale*** |
| 30 | Rohmani et al., 2021^55^ | September 20 | Indonesia | NR | CS | NR | 69 | 19 (0.85) 17–23 | M: 15 (21.7) F: 54 (78.3) | Nursing | Y1 | MBI-SS |
| 31 | Rolland et al., 2022^56^ | May 27 – June 27, 2021 | France | 15.3 | CS | All students  were invited | 7952 | NR  ≥18 | M: 2200 (27.7) F: 5710 (71.8) Missing: 42 (0.5) | Medicine | Y2-Y6 | MBI-SS & MBI-HSS |
| 32a**** | Ruiz et al., 2022^57^ | 2017 | Guatemala | 56.79 | CS | All students  were invited | 159 | NR | M/F | Medicine | NR | MBI-SS |
| 32b**** | Ruiz et al., 2022^57^ | 2020 | Guatemala | 48.53 | CS | All students  were invited | 132 | NR | M/F | Medicine | NR | MBI-SS |
| 33 | Salgado et al., 2021^58^ | March 30 - April 30, 2020 | Portugal | NR | CS | NR | 207 | NR | M: 64 (31%) F: 143 (69%) | All fields | NR | MBI-SS |
| 34 | Seperak-Viera et al., 2021^59^ | July 12 – August 9, 2020 | Peru | 32.7 | CS | Non-  probabilistic | 341 | 21.87 (4.157) 17-72 | M: 82 (24.0)  F: 259 (76.0) | All fields | Y2-Y4 | ECE |
| 35 | Shreshtha et al., 2021^60^ | January 14 – March 7, 2021 | Nepal | 95.82 | CS | Stratified sampling | 229 | 22.05 (1.84)  Median 22  IQR 21–23 | M: 148 (64.6) F: 81 (35.4) | Medicine | Y1-Y5 | OLBI-S |
| 36 | Silistraru et al., 2022^61^ | December 2020 – April 2021 | Romania | NR | CS | NR | 126 | University 1:  25.01 (4.40) University 2:  24.69 (2.58)  NR | M: 31 (24.6) F: 95 (75.4) | Medicine | NR | MBI-SS |
| 37 | Tee et al., 2022^62^ | May – July 2021 | Malaysia | 83.1 | CS | “Universal” | 250 | 23.1 (1.0)  21-26 | M: 128 (33.9%) F: 250 (66.1%) | Medicine | Y3-Y5 | CBI |
| 38 | Toubasi et al., 2022^63^ | May 26 - September 25, 2021 | Jordan | NR | CS | Convenience sampling | 587 | 20.5 (2.6)  18-62 | M: 143 (24.4%) F: 443 (75.6%) | All fields | Y1-Y6 | MBI-SS |
| 39 | Wang J et al., 2021^64^ | April – June 2020 | China | 96.8 | CS | Convenience sampling | 733 | 20.02 (1.45) 17-24 | M: 131 (17.9) F: 602 (82.1) | Nursing | Y1-Y4 | ABS |
| 40 | Yahya et al., 2021^65^ | June 11 - July 3, 2020 | Iraq | 42 | CS | All students  were invited | 424 | 20.74 (2.0)  18-33 | M: 129 (30.4%) F: 295 (69.6%) | Medicine | Y1-Y6 | MBI-SS |
| 41 | Zhang et al., 2021^66^ | April 2020 | China | 30.9 | CS | Convenience sampling | 683 | 20 (NR) 17-24 | M: 290 (42.5) F: 393 (57.5) | Medicine | Y2 and above | LBS |
| 42a**** | Zis et al., 2021^67^ | January 2020 | Cyprus | 96.30 | LS | All students  were invited | 182 | NR | NR | Medicine | Y1-Y6 | MBI-SS |
| 42b**** | Zis et al., 2021^67^ | May 2020 | Cyprus | 81.48 | LS | All students  were invited | 154 | 22.6 (4.1) 18-52 | M: 47 (30.5) F 107 (69.5) | Medicine | Y1-Y6 | MBI-SS |
| 43a**** | Žuljević et al., 2021^68^ | December 2019 – January 2020 | Croatia | NR | LS | All students  were invited | 437 | NR  18-28 | M: 125 (31.2) F: 276 (68.8)  Missing: 36 | Medicine | Y1-Y6 | OLBI |
| **Study No** | **Citation** | **Study Period** | **Country** | **Response rate (%)** | **Study Design** | **Sampling Technique** | **Sample**  **size** | **Age (yrs Mean (SD) Range** | **Sex n (%)** | **Field of study** | **Year of study** | **Instrument Used** |
| 43b**** | Žuljević et al., 2021^68^ | June 1-20, 2020 | Croatia | NR | LS | All students  were invited | 199 | NR  18-28 | M: 64 (32.2) F: 135 (67.8) | Medicine | Y1-Y6 | OLBI |
| 43c**** | Žuljević et al., 2021^68^ | December 2019 – January 2020 | Croatia | NR | LS | All students  were invited | 342 | NR  18-28 | M: 106 (31.0) F: 236 (69.0) | Medicine | Y1-Y6 | CBI-modified |
| 43d**** | Žuljević et al., 2021^68^ | June 1-20, 2020 | Croatia | NR | LS | All students  were invited | 181 | NR  18-28 | M: 51 (28.2) F: 130 (71.8) | Medicine | Y1-Y6 | CBI-modified |
| 44 | Zuniga et al., 2021^69^ | April - May 2020 | Chile | 100 | CS | All students  were invited | 123 | NR | M: 63 (51.2) F: 60 (48.8) | Medicine | Y4 | MBI-HSS |

M=Male; F= Female; LS= Longitudinal study; CS= Cross-sectional study; NR= Not reported; Y=Year;; IQR=Inter-Quartile Range MBI-SS= Maslach Burnout Inventory Student Survey; MBI-HSS= Maslach Burnout Inventory Human Service Survey; aMBI= abbreviated Maslach Burnout Inventory; OLBI= Oldenburg Burnout Inventory; OLBI-S= Oldenburg Burnout Inventory- Student version; CBI= Copenhagen Burnout Inventory ; CBI-modified= Copenhagen Burnout Inventory (modified version); CBI-S= Copenhagen Burnout Inventory-Students; LBQ= Link Burnout Questionnaire; ABS= Academic Burnout Scale; LBS= Learning Burnout Scale; ECE= Emotional Exhaustion Scale; PWS=Physician Worklife Study; BCSQ-12-SS=Burnout clinical subtype questionnaire students survey

*Burnout scale adapted from: ^!^Unknown reference – citation in included study is incorrect.

**^*^based on the Student School Burnout Scale (SSBS) by Aypay (2012), Academic Burnout Questionnaire designed by Breso et al. (2007), CBI (Fiorilli et al., 2015), the Italian version of School Burnout Inventory (Platania et al., 2020), OLBI-S (Campo et al., 2012), ABS developed by Rong et al. (2005) and the School Burnout Inventory developed by Salmera-Aro and Naatanen (2005) (unspecified language)

***Burnout scale: Hubungan antara stres dengan burnout pada mahasiswa fakultas psikologi universitas islam negeri (uin) maulana malik ibrahim malang. Skripsi. Universitas Islam Negeri Maulana Malik Ibrahim

**** These studies used multiple instruments or collected data across multiple time periods. Consequently, the total sample size of the SR cannot be calculated by summing the total number of participants in this table due to overlap.

## Supplementary Figure 1: PRISMA 2020 flow diagram for new systematic reviews which included searches of databases, registers and other sources

**Identification of studies via other methods**

**Identification of studies via databases and registers**

Records (n = 5597) identified from*:

PubMed (n = 2151)

EMBASE (n = 199)

PsycINFO (n = 422)

WHO Global COVID Database (n = 831)

Scopus (n = 1818)

ERIC (n = 176)

Registers (n = 0)

Records (n = 67) identified from:

Google Scholar (n=22) Epistemonikos (n = 5)

Citation searching (n = 22)

Contacting experts (n = 0)

Identified via excluded SRs (n=18)

Records (n = 680) removed *before screening*:

Duplicate records removed (n = 679)

Records marked as ineligible by automation tools (n = 1)

Records removed for other reasons (n = 0)

**Identification**

Records screened

(n = 4917)

Records excluded**

(n = 3600)

Reports not retrieved

(n = 0)

Reports sought for retrieval

(n = 67)

Reports sought for retrieval

(n = 1317)

Reports not retrieved

(n = 2)

**Screening**

Reports (n = 1306) excluded:

No burnout prevalence (n = 746)

Non-university students (n = 475)

Wrong publication type (n = 68)

Unvalidated questionnaire (n = 10)

Duplicates (n = 6)

Paper retracted due to duplication (n = 1)

Reports assessed for eligibility

(n = 67)

Reports (n = 32) excluded:

Data not collected during COVID-19 pandemic (n = 28)

Other language (n = 2)

No prevalence data (n = 1)

Unvalidated questionnaire (n = 1)

Reports assessed for eligibility

(n = 1315)

Studies included in review

(n = 44)

Reports of included studies

(n = 44)

**Included**

*Consider, if feasible to do so, reporting the number of records identified from each database or register searched (rather than the total number across all databases/registers).

**If automation tools were used, indicate how many records were excluded by a human and how many were excluded by automation tools.

*From:*  Page MJ, McKenzie JE, Bossuyt PM, Boutron I, Hoffmann TC, Mulrow CD, et al. The PRISMA 2020 statement: an updated guideline for reporting systematic reviews. BMJ 2021;372:n71. doi: 10.1136/bmj.n71. For more information, visit: <http://www.prisma-statement.org/>

Note:

Overall, 5664 records were screened for eligibility, and 44 studies were found to be eligible (Supplementary Figure 1 and Supplementary Table 5), with 26,500 participants from 31 countries. These were classified as being from the World Bank’s High-Income Countries (HICs) (21/44 studies; 47.7%), Upper-Middle-Income Countries (UMICs) (12/44; 27.3%), Lower-Middle Income Countries (LMICs) (10/44; 22.3%) and Lower Income Countries (LIC) (1/44; 2.3%). Most studies were from Europe (14/44; 31.8%), followed by the Americas (10/44; 22.7%), South East Asia (8/44; 18.2%), the Eastern Mediterranean (6/44; 13.6%), the Western Pacific (4/44; 9.1%) and Africa (2/44; 4.5%). The MBI and its adapted versions (i.e., aMBI, MBI-SS, MBI-HSS) were the most used instruments (23/44 studies; 52.3%, Supplementary Table 6), followed by the OLBI and its student version (i.e., OLBI-S) (8/44; 18.2%, Supplementary Table 7), the ECE (3/44; 6.8% Supplementary Table 6), and other instruments (Supplementary Tables 8 and 9). Almost all studies included in the SR (41/44; 93.2%) were cross-sectional; the remaining three were longitudinal – consisting of two phases conducted before and during the pandemic. Nearly two-thirds of the studies (27/44; 61.4%) focused on medical students, with the remainder covering all fields (11/44; 25.0%), nursing (3/44; 6.8%), dentistry (1/44; 3.0%), psychology (1/44; 2.3%), and pharmacy (1/44; 2.3%). Most studies (38/44; 86.4%) identified sexes as male and female, four (9.1%) identified male, female, and other sexes, and two (4.5%) did not mention sex. Supplementary Table 5 summarizes the characteristics of primary studies.

**Supplementary Table 6: Reported prevalence of overall burnout and domains by MBI and ECE**

Twenty-four prevalence measures from 21 countries included in our SR reported the prevalence of students with a risk of high EE (Supplementary Table 6). These studies comprised medical students (12/24, 50.0%), students from all disciplines of study (unspecified, 7/24, 29.7%), psychology students (1/24, 4.2%) and pharmacy students (1/24, 4.2%). Seven countries reported EE prevalence stratified by sex: India, Mexico, Poland, Peru, Russia, and Saudi Arabia.

Twenty-four prevalence measures from 21 countries included in our SR reported the prevalence of students with a risk of high DP/CY (Supplementary Table 6). DP/CY prevalence measures stratified by sex were reported in seven countries: Croatia, Denmark, India, Mexico, Poland, Russia, and Saudi Arabia.

Eleven prevalence measures from nine countries included in our SR reported the prevalence of students with a risk of low PA/AE (Supplementary Table 6). PA/AE prevalence measures stratified by sex were reported in two countries: Poland and Saudi Arabia.

| **Study No** | **Citation** | **Study Period** | **Country** | **Sample size** | **Field of study** | **Sex** | **Year of study** | **Definition of OB** | **Reported cutoffs** | | | | **Prevalence n (%)** | | | |
| --- | --- | --- | --- | --- | --- | --- | --- | --- | --- | --- | --- | --- | --- | --- | --- | --- |
|  |  |  |  |  |  |  |  |  | **OB** | **EE** | **DP/CY** | **PA/AE** | **OB (%)** | **EE (%)** | **DP/CY (%)** | **PA/AE (%)** |
| **High-Income Countries- MBI-SS** | | | | | | | | | | | | | | | | |
| 2 | Aguayo-Estremera et al., 2023^28^ | During COVID | Spain | 134 | Psychology | All | Y1 | NR | NR | ≥ 27 | ≥10 | ≤ 33 | NR | 29 (21.6) | 28 (21.1) | 34 (25.4) |
| 2 | Alqifari et al., 2021^29^ | During COVID | Saudi Arabia | 336 | Medicine | All | Y1-Y6 | High EE and CY and low PE or High EE and CY | NR | ≥26 | ≥20 | ≤18 | 30 (8.9) | 99  (29.5) | 112 (33.3) | 114 (31.8) |
| 3 | Alqifari et al., 2021^29^ | During COVID | Saudi Arabia | 336 | Medicine | All | Y1-Y6 | High EE and CY | NR | ≥26 | ≥20 | ≤18 | 61 (18.2) | 99  (29.5) | 112 (33.3) | 107 (33.9) |
| 14 | El Mouedden et al., 2022^40^ | During COVID | Belgium | 145 | Medicine | All | Y4-Y6 | High EE and CY and low AE | NR | ≥ 23 | ≥18 | ≤16 | 1 (0.5) | 34 (23.4) | 16 (10.8) | 12 (8.2) |
| 16 | Forycka et al., 2022^42^ | During COVID | Poland | 1858 | Medicine | All | Y1-Y6 | High EE and CY and low AE | NR | ≥16 | ≥13 | 0-23 | 711 (38.2) | 952 (51.2) | 924 (49.7) | 1084 (58.3) |
| 16 | Forycka et al., 2022^42^ | During COVID | Poland | 1363 | Medicine | All | Y1-Y6 | High EE and CY | NR | ≥16 | ≥13 | 0-23 | 786 (59.9) | 952 (69.8) | 924 (70.5) | 1084 (82.7) |
| 23 | Moreno-Fernandez et al., 2020^48^ | During COVID | Spain | 47 | Pharmacy | All | Y1 | NR | NR | NR | NR | NR | 30 (63.5) | 21 (44.6) | 20 (41.7) | 28 (60.3) |
| 24 | Muaddi et al., 2023^49^ | During COVID | Saudi Arabia | 433 | Medicine | All | Y2-Y6 | High EE and CY and low AE | NR | >14 | >6 | <23 | 238 (55.0) | 301 (69.5) | 319 (73.7) | 394 (91.0) |
| 31 | Rolland et al., 2022^56^ | During COVID | France | 3167 | Medicine | All | Y2-Y3 | High EE and CY and low AE | NR | NR | NR | NR | 1235 (39.0) | NR | NR | NR |
| 33 | Salgado et al., 2021^58^ | During COVID | Portugal | 207 | All fields | All | NR | Average of 3 subscales>3 | NR | NR | NR | NR | 60 (29.0) | 77 (37.2) | 35 (17.0) | 75 (36.0) |
| 36 | Silistraru et al., 2022^61^ | During COVID | Romania | 126 | Medicine | All | NR | High EX and CY and low PE | NR | NR | NR | NR | 46 (36.5) | NR | NR | NR |
| **Study No** | **Citation** | **Study Period** | **Country** | **Sample size** | **Field of study** | **Sex** | **Year of study** | **Definition of Burnout** | **Reported cutoffs** | | | | **Prevalence n (%)** | | | |
|  |  |  |  |  |  |  |  |  | **OB** | **EE** | **DP/CY** | **PA/AE** | **OB** | **EE** | **DP/CY** | **PA/AE** |
| 42a | Zis et al., 2021^67^ | Pre-COVID | Cyprus | 182 | Medicine | All | Y1-Y6 | High EE and CY, or High EE and a Low EF | NR | NR | NR | NR | 33 (18.1) | NR | NR | NR |
| 42b | Zis et al., 2021^67^ | During COVID | Cyprus | 154 | Medicine | All | Y1-Y6 | High EE and CY, or High EE and Low EF | NR | NR | NR | NR | 28 (18.2) | NR | NR | NR |
| **High-Income Countries- MBI-HSS** | | | | | | | | | | | | | | | | |
| 4 | Alsaad et al., 2021^30^ | During COVID | Saudi Arabia | 439 | All fields | All | Y1-Y6 | High EE | NR | NR | NR | NR | 134 (30.5) | NR | NR | NR |
| 4 | Alsaad et al., 2021^30^ | During COVID | Saudi Arabia | 120 | All fields | Males | Y1-Y6 | High EE | NR | NR | NR | NR | 42 (35.0) | NR | NR | NR |
| 4 | Alsaad et al., 2021^30^ | During COVID | Saudi Arabia | 319 | All fields | Females | Y1-Y6 | High EE | NR | NR | NR | NR | 92 (28.8) | NR | NR | NR |
| 31 | Rolland et al., 2022^56^ | During COVID | France | 4785 | Medicine | All | Y4-Y6 | High EE and DP | NR | NR | NR | NR | 3277 (68.0) | NR | NR | NR |
| 44 | Zuniga et al., 2021^69^ | During COVID | Chile | 123 | Medicine | All | Y4 | High EE or DP | NR | NR | NR | NR | 59 (48.0) | NR | NR | NR |
| **High-Income Countries- aMBI** | | | | | | | | | | | | | | | | |
| 18 | Johns et al., 2022^44^ | During COVID | UK | 82 | Medicine | All | Final Year | High EE and DP and low PA | NR | ≥27 | ≥10 | ≤33 | 8 (9.8) | 38 (46.3) | 19 (23.2) | 28 (34.1) |
| 20 | Khalafallah et al., 2021^46^ | During COVID | USA | 254 | Medicine | All | All years & research / elective year | High EE or DP | NR | 13-18 | 13-18 | 0-6 | 38 (15.0) | 36 (14.2) | 8 (3.1) | 158 (62.2) |
| **High-Income Countries- ECE** | | | | | | | | | | | | | | | | |
| 22 | Martinez-Libano et al., 2021^19^ | During COVID | Chile | 315 | All fields | All | Y1-Y5 | NA | NA | 207 (65.7) | NA | NA | NA | NR | NA | NA |
| **Upper- Middle-Income Countries- MBI-SS** | | | | | | | | | | | | | | | | |
| 7 | Azzi et al, 2022b^33^ | Overall | Brazil | 63 | All fields | All | NR | High EE and CY & low AE | NR | >14 | >6 | <23 | 10 (66.6) | 45 (71.4) | 63.0 (100) | 58 (92.1) |
| 11 | Cipta et al., 2022^37^ | During COVID | Indonesia | 1947 | Medicine | All | Y1-Y4 | High EE and/or high DP | NR | ≥27 | ≥10 | ≤33 | 692 (35.5) | 607 (31.2) | 360 (18.5) | 980 (50.3) |
| 32a | Ruiz et al., 2022^57^ | Pre-COVID | Guatemala | 159 | Medicine | All | NR | High EE and CY & low PA | NR | >14 | >6 | <23 | 8 (5.0) | 8.0 (5.0) | 137 (86.2) | 158 (99.4) |
| 32b | Ruiz et al., 2022^57^ | During COVID | Guatemala | 132 | Medicine | All | NR | High EE and CY & low PA | NR | >14 | >6 | <23 | 18 (13.6) | 19 (14.4) | 102 (77.3) | 121 (91.7) |
| 17 | Jezzini-Martinez et al, 2022^43^ | During COVID | Mexico | 154 | Medicine | All | NR | High EE and CY & low AE | NR | >14 | >6 | <22 | 13 (14.9) | 83 (53.9) | 26 (16.9) | 101 (65.6) |
| 17 | Jezzini-Martinez et al, 2022^43^ | During COVID | Mexico | 61 | Medicine | Males | NR | High EE and CY & low AE | NR | NR | NR | NR | 16 (26.2) | NR | NR | NR |
|  |  |  |  |  |  |  |  |  |  | | | |  | | | |
| **Study No** | **Citation** | **Study Period** | **Country** | **Sample size** | **Field of study** | **Sex** | **Year of study** | **Definition of Burnout** | **Reported cutoffs** | | | | **Prevalence n (%)** | | | |
|  |  |  |  |  |  |  |  |  | **OB** | **EE** | **DP/CY** | **PA/AE** | **OB** | **EE** | **DP/CY** | **PA/AE** |
| 17 | Jezzini-Martinez et al, 2022^43^ | During COVID | Mexico | 93 | Medicine | Females | NR | High EE and CY & low AE | NR | NR | NR | NR | 7 (7.5) | NR | NR | NR |
| 38 | Toubasi et al., 2022^63^ | During COVID | Jordan | 587 | All fields | All | Y1-Y6 | High EE and CY & low AE | NR | >12.5 | >7.5 | <10.5 | 39 (6.6) | 572 (97.4) | 533 (93.8) | 39 (6.6) |
| 40 | Yahya et al., 2021^65^ | During COVID | Iraq | 424 | Medicine | All | Y1-Y6 | High EE and CY & low AE | NR | >14 | >6 | >27 | 162 (38.2) | 363 (85.6) | 330 (77.8) | 193 (45.5) |
| **Upper- Middle-Income Countries- ECE** | | | | | | | | | | | | | | | | |
| 15 | EstradaAraoz, 2021^41^ | During COVID | Peru | 265 | All fields | All | NR | NR | NR | NR | NR | NR | NR | 105 (39.6) | NA | NA |
| 34 | Seperak-Viera et al., 2021^59^ | During COVID | Peru | 341 | All fields | All | Y2-Y4 | NR | NR | NR | NR | NR | NR | 43 (12.6) | NA | NA |
| **Lower- Middle-Income Countries- MBI-SS** | | | | | | | | | | | | | | | | |
| 19 | Kajjimu et al., 2021^45^ | During COVID | Uganda | 145 | Medicine | All | Y1-Y5 | High EE and CY & low PE | NR | >14 | >6 | <23 | 79 (54.5) | 135 (93.1) | 141 (97.2) | 42 (29.0) |
| 19 | Kajjimu et al., 2021^45^ | During COVID | Uganda | 102 | Medicine | Males | Y1-Y5 | High EE and CY & low PE | NR | >14 | >6 | <23 | 56 (70.9) | NR | NR | NR |
| 19 | Kajjimu et al., 2021^45^ | During COVID | Uganda | 43 | Medicine | Females | Y1-Y5 | High EE and CY & low PE | NR | >14 | >6 | <23 | 23 (29.1) | NR | NR | NR |
| 26 | Periasamy et al., 2021^51^ | During COVID | India | 154 | Medicine | All | Y1-Y4 | High EE and CY & low AE | NR | NR | NR | NR | 28 (18.2) | NR | NR | NR |
| 26 | Periasamy et al., 2021^51^ | During COVID | India | 63 | Medicine | Males | Y1-Y4 | High EE and CY & low AE | NR | NR | NR | NR | 12 (19.1) | NR | NR | NR |
| 26 | Periasamy et al., 2021^51^ | During COVID | India | 91 | Medicine | Females | Y1-Y4 | High EE and CY & low AE | NR | NR | NR | NR | 16 (17.6) | NR | NR | NR |
| 30 | Rohmani et al., 2021^55^ | During COVID | Indonesia | 69 | Nursing | All | Y1 | NR | ≥97 | NR | NR | NR | 32.0 (46.4) | NR | NR | NR |

Y=Year; NR=Not reported; NA=Not Applicable; MBI-SS= Maslach Burnout Inventory Student survey; MBI-HSS= Maslach Burnout Inventory Human Service Survey; aMBI= abbreviated Maslach Burnout Inventory; ECE= Emotional Exhaustion Scale; OB= Overall burnout; EE= Emotional exhaustion; CY= Cynicism; DP=Depersonalisation; AE= Academic Efficacy; EF= efficacy; PA= Personal accomplishment

**Supplementary Table 7: Reported prevalence of overall burnout and domains by OLBI**

| **Study No** | **Citation** | **Study Period** | **Country** | **Sample size** | **Field of study** | **Year of study** | **Definition of Burnout** | **Reported domains and cut-offs** | | | **Prevalence n (%)** | | |
| --- | --- | --- | --- | --- | --- | --- | --- | --- | --- | --- | --- | --- | --- |
|  |  |  |  |  |  |  |  | **OB** | **EE** | **DE** | **OB** | **EE** | **DE** |
| **High-Income Country** | | | | | | | | | | | | | |
| 1 | Abdel Aziz et al., 2023^27^ | During COVID | United Arab Emirates | 385 | Medicine | Y1-Y6 | High EE + High DE | NR | ≥2.25 | ≥2.1 | 298 (77.4) | 366 (95.1) | 313 (81.3) |
| 21 | Kjær et al., 2022^47^ | Pre-COVID | Denmark | 259 | Medicine | Y1-Y6 | NR | NR | ≥2.25 | ≥2.1 | NR | 194 (74.9) | 179 (69.1) |
| 21 | Kjær et al., 2022^47^ | During COVID | Denmark | 387 | Medicine | Y1-Y6 | NR | NR | ≥2.25 | ≥2.1 | NR | 259 (66.9) | 279 (72.1) |
| 43a | Žuljević et al., 2021^68^ | Pre-COVID | Croatia | 401* | Medicine | Y1-Y6 | NR | NR | ≥2.25 | ≥2.1 | 225 (51.5)* | 334 (83.3)* | 243 (55.6)* |
| 43b | Žuljević et al., 2021^68^ | During COVID | Croatia | 199* | Medicine | Y1-Y6 | NR | NR | ≥2.25 | ≥2.1 | 91 (45.7)* | 152 (76.4)* | 104 (52.2)* |
| **Upper- Middle-Income Countries** | | | | | | | | | | | | | |
| 10 | Chumakov et al., 2021^36^ | During COVID | Russia | 165 | Medicine | Y1-Y6 | Combined mean score of 2.25 for EE and 2.10 for DE | NR | ≥2.25 | ≥2.1 | NR | 132 (80.0) | 121 (73.3) |
| **Lower-Middle- Income Country** | | | | | | | | | | | | | |
| 5 | Ayinde et al., 2022^31^ | During COVID | Nigeria | 505 | Medicine | Y1-Y6 | NR | NR | ≥2.25 | ≥2.1 | NR | 389 (77.0) | 427 (84.6) |
| 12 | Dahanayake et al., 2022^38^ | During COVID | Sri Lanka | 1097 | Medicine | Y1-Y6 | NR | NR | ≥2.25 | ≥2.1 | NR | 867 (79.0) | 1022 (93.1) |
| 27 | Philip et al., 2021^52^ | During COVID | India | 344 | Medicine | Y1-Y6 | NR | NR | ≥2.25 | ≥2.1 | NR | 275 (80.6) | 294 (86.2) |
| 35 | Shreshtha et al., 2021^60^ | During COVID | Nepal | 229 | Medicine | Y1-Y5 | High EE and DE | NR | ≥2.25 | ≥2.1 | 151 (65.9) | 29 (12.7) | 26 (11.4) |

*Calculated using raw data; OLBI= Oldenburg Burnout Inventory

OB=Overall Burnout; Y=Year; NR=Not reported; EE=Exhaustion; DE=Disengagement

**Supplementary Table 8: Reported prevalence of overall burnout and domains by CBI**

| **Study No** | **Citation** | **Study Period** | **Country** | **Sample size** | **Field of study** | **Year of study** | **Sex** | **Definition of Burnout** | **Prevalence n (%)** | | | | | | | |
| --- | --- | --- | --- | --- | --- | --- | --- | --- | --- | --- | --- | --- | --- | --- | --- | --- |
|  |  |  |  |  |  |  |  |  | **OB** | **Professor-related burnout** | **Patient-related burnout** | **Student-related burnout** | **Personal related burnout** | **Studies related burnout** | **Colleague related burnout** | **Teacher related burnout** |
| **High-Income Country** | | | | | | | | | | | | | | | | |
| 43c | Žuljević et al., 2021^68$^ | Pre COVID | Croatia | 342* | Medicine | Y1-Y6 | All | NR | NR | 98 (28.7)* | 43 (12.6)* | 72 (12.1)* | 224 (65.5)* | 207 (60.5)* | NA | NA |
| 43c | Žuljević et al., 2021^68$^ | Pre COVID | Croatia | 106* | Medicine | Y1-Y6 | Males | NR | NR | 26 (24.5)* | 20 (18.9)* | 22 (20.8)* | 62 (58.5)* | 56 (52.8)* | NA | NA |
| 43c | Žuljević et al., 2021^68$^ | Pre COVID | Croatia | 236* | Medicine | Y1-Y6 | Females | NR | NR | 72 (30.5)* | 23 (9.7)* | 50 (21.2)* | 162 (68.6)* | 151 (64)* | NA | NA |
| 43d | Žuljević et al., 2021^68$^ | During COVID | Croatia | 181* | Medicine | Y1-Y6 | All | NR | NR | 48 (26.5)* | 27 (14.9)* | 45 (24.9)* | 51 (28.2)* | 87 (48.1)* | NA | NA |
| 43d | Žuljević et al., 2021^68$^ | During COVID | Croatia | 51* | Medicine | Y1-Y6 | Males | NR | NR | 14 (27.5)* | 10 (19.6)* | 11 (21.6)* | 20 (39.2)* | 22 (43.1)* | NA | NA |
| 43d | Žuljević et al., 2021^68$^ | During COVID | Croatia | 130* | Medicine | Y1-Y6 | Females | NR | NR | 34 (26.2)* | 17 (13.1)* | 34 (26.2)* | 31 (23.8)* | 65 (50)* | NA | NA |
| **Upper-Middle-Income Country** | | | | | | | | | | | | | | | | |
| 8a | Bolatov et al., 2021^34#^ | Pre COVID | Kazakhstan | 619 | Medicine | Y1-Y5 | All | combined scores of all 4 subscales | 171 (27.6) | NA | NA | NA | 345 (55.7) | 340 (54.9) | 81 (13.1) | 149 (24.1) |
| 8b | Bolatov et al., 2021^34#^ | During COVID | Kazakhstan | 798 | Medicine | Y1-Y5 | All | combined scores of all 4 subscales | 133 (16.7) | NA | NA | NA | 249 (31.2) | 271 (34.0) | 136 (17.0) | 143 (17.9) |
| 37 | Tee *et al.,* 2022^62^ | During COVID | Malaysia | 378 | Medicine | Y3-Y5 | All | The mean of the total score of each subscale | 84 (22.2) | NR | NR | NR | NR | NR | NR | NR |

*Calculated using raw data & combined moderate, high and severe scores; ^#^Used CBI-S; ^$^Used modified version of CBI; CBI-S= Copenhagen Burnout Inventory-Students

The cut-offs for burnout and the domains were either unclear or not reported

OB= Overall Burnout; NR=Not reported; NA=Not applicable

**Supplementary Table 9: Reported prevalence of overall burnout and domains by other instruments**

| **Study No** | **Citation** | **Study Period** | **Country** | **World Bank Income Group** | **Sample size** | **Field of study** | **Year of study** | **Definition of Burnout** | **Reported domains & cut-offs** | | | | **Prevalence n (%)** | | | |
| --- | --- | --- | --- | --- | --- | --- | --- | --- | --- | --- | --- | --- | --- | --- | --- | --- |
| Academic Burnout Scale | | | | | | | | | **OB** | **IB** | **DE** | **PA** | **OB** | **IB** | **DE** | **PA** |
| 39 | Wang J et al., 2021^64^ | During COVID | China | UMIC | 733 | Nursing | Y1-Y4 | >average score of the sample | ≥4 | NR | NR | NR | 288 (39.29) | NR | NR | NR |
| Burnout Clinical Subtype Questionnaire Students Survey (BCSQ-12-SS) | | | | | | | | | **OB** | **O** | **LD** | **N** | **OB** | **O** | **LD** | **N** |
| 6 | Azzi et al, 2022a^32^ | During COVID | Brazil | UMIC | 703 | All fields | NR | NR | NR | ≥ 18 | ≥ 18 | ≥ 18 | NR | 129 (18.35) | 83 (11.8) | 35 (4.9) |
| de novo instrument based on multiple instruments* | | | | | | | | | **OB** | | | | **OB** | | | |
| 28 | Ramos et al., 2021^53^ | During COVID | Philippines | LMIC | 245 | All fields | Y1-Y4 | Three dimensional | >70 | | | | 139 (56.8) | | | |
| Learning Burnout Scale (LBI) | | | | | | | | | **OB** | **IB** | **DE** | **PA** | **OB** | **IB** | **DE** | **PA** |
| 41 | Zhang et al., 2021^66^ | During COVID | China | UMIC | 683 | Medicine | Y2 and above | NR | >3 | NR | NR | NR | 315 (46.1) | 328 (48.0) | 370 (54.17) | 300 (43.92) |
| Link Burnout Questionnaire (LBQ) | | | | | | | | | **OB** | | | | **OB** | | | |
| 13 | Dlugosz et al., 2021^39^ | During COVID | Poland | HIC | 1870 | All fields | Y1-Y5 | NR | ≥59 | | | | 1643 (87.9) | | | |
| Physician Worklife Survey (PWS) | | | | | | | | | **OB** | | | | **OB** | | | |
| 9 | Chi et al, 2021^35^ | During COVID | USA | HIC | 109 | Dentistry | Y1-Y4 | 1 or more symptoms of burnout | >70 | | | | 31 (12.7) | | | |
| Instrument validated by Kurniathi et al., 2012^$^ (Indonesian language) | | | | | | | | | **OB** | | | | **OB** | | | |
| 29 | Remitha et al., 2020^54^ | During COVID | Indonesia | LMIC | 175 | Medicine | Y1-Y2 | NR | NR | | | | 158 (90.3) | | | |
| Instrument validated by Budiman et al., 2016 (Indonesian language)^!^ | | | | | | | | | **OB** | | | | **OB** | | | |
| 25 | Nurhidayati et al., 2021^50^ | During COVID | Indonesia | LMIC | 83 | Nursing | Under graduate | NR | NR | | | | 93 (100.0) | | | |

OB=Overall Burnout; IB=Inappropriate behaviour; DE=Dejection; PA=Personal accomplishment; O=Overload; LD= Lack of Development; N=Neglect; NR=Not reported; Y=Year; HIC=High Income Country; UMIC=Upper Middle Income Country; LMIC=Lower Middle Income Country

^*^based on the Student School Burnout Scale (SSBS) by Aypay (2012), Academic Burnout Questionnaire designed by Breso et al. (2007), Copenhagen Burnout Inventory (Fiorilli et al., 2015), the Italian version of School Burnout Inventory (Platania et al., 2020), Oldenburg Burnout Inventory -student version (Campo et al., 2012), Academic Burnout Scale developed by Rong et al. (2005) and the School Burnout Inventory developed by Salmera-Aro and Naatanen (2005) (unspecified language)

^$^Kurniati F (2012) Hubungan antara stres dengan burnout pada mahasiswa fakultas psikologi universitas islam negeri (uin) maulana malik ibrahim malang. Skripsi. Universitas Islam Negeri Maulana Malik Ibrahim.

^!^Unknown reference – citation in included study is incorrect

**Supplementary Table 10: Quality assessment of primary studies**

| Study | Q1 | Q2 | Q3 | Q4 | Q5 | Q6 | Q7 | Q8 | Q9 | Q10 | COI | Funding |
| --- | --- | --- | --- | --- | --- | --- | --- | --- | --- | --- | --- | --- |
| Abdel Aziz *et al.*, 2023^27^ |  |  |  |  |  |  |  |  |  |  |  |  |
| Aguayo-Estremera *et al.*, 2023^28#^ |  |  |  |  |  |  |  |  |  |  |  |  |
| Alqifari *et al.*, 2021^29^ |  |  |  |  |  |  |  |  |  |  |  |  |
| Alsaad *et al.*, 2021^30^ |  |  |  |  |  |  |  |  |  |  |  |  |
| Ayinde *et al.*, 2022^31^ |  |  |  |  |  |  |  |  |  |  |  |  |
| Azzi *et al.*, 2022a^32^ |  |  |  |  |  |  |  |  |  |  |  |  |
| Azzi *et al.*, 2022b^33^ |  |  |  |  |  |  |  |  |  |  |  |  |
| Bolatov *et al.*, 2021^34^ |  |  |  |  |  |  |  |  |  |  |  |  |
| Chi *et al.*, 2021^35^ |  |  |  |  |  |  |  |  |  |  |  |  |
| Chumakov *et al.*, 2021^36^ |  |  |  |  |  |  |  |  |  |  |  |  |
| Cipta *et al.*, 2022^37^ |  |  |  |  |  |  |  |  |  |  |  |  |
| Dahanayake *et al.*, 2022^38^ |  |  |  |  |  |  |  |  |  |  |  |  |
| Dlugosz *et al.*, 2021^39^ |  |  |  |  |  |  |  |  |  |  |  |  |
| El Mouedden *et al.,* 2022^40^ |  |  |  |  |  |  |  |  |  |  |  |  |
| Estrada Araoz *et al.,* 2021^41^ |  |  |  |  |  |  |  |  |  |  |  |  |
| Forycka *et al.,* 2022^42^ |  |  |  |  |  |  |  |  |  |  |  |  |
| Jezzini-Martinez *et al.,* 2022^43^ |  |  |  |  |  |  |  |  |  |  |  |  |
| Johns *et al.,* 2022^44^ |  |  |  |  |  |  |  |  |  |  |  |  |
| Kajjimu *et al.,* 2021^45^ |  |  |  |  |  |  |  |  |  |  |  |  |
| Khalafallah *et al.,* 2021^46^ |  |  |  |  |  |  |  |  |  |  |  |  |
| Kjær *et al.,* 2022^47$^ |  |  |  |  |  |  |  |  |  |  |  |  |
| Martinez-Libano *et al.,* 2021^19^ |  |  |  |  |  |  |  |  |  |  |  |  |
| Moreno-Fernandez *et al.,* 2020^48^ |  |  |  |  |  |  |  |  |  |  |  |  |
| Muaddi *et al.,* 2023^49^ |  |  |  |  |  |  |  |  |  |  |  |  |
| Nurhidayati *et al.,* 2021^50^ |  |  |  |  |  |  |  |  |  |  |  |  |
| Periasamy *et al.,* 2021^51^ |  |  |  |  |  |  |  |  |  |  |  |  |
| Philip *et al.,* 2021^52^ |  |  |  |  |  |  |  |  |  |  |  |  |
| Ramos *et al.,* 2021^53^ |  |  |  |  |  |  |  |  |  |  |  |  |
| Remitha *et al.,* 2020^54^ |  |  |  |  |  |  |  |  |  |  |  |  |
| Rohmani *et al.,* 2021^55^ |  |  |  |  |  |  |  |  |  |  |  |  |
| Rolland *et al.,* 2022^56^ |  |  |  |  |  |  |  |  |  |  |  |  |
| Ruiz *et al.,* 2022^57^ |  |  |  |  |  |  |  |  |  |  |  |  |
| Salgado *et al.,* 2021^58^ |  |  |  |  |  |  |  |  |  |  |  |  |
| Seperak-Viera *et al*., 2021^70^ |  |  |  |  |  |  |  |  |  |  |  |  |
| Shreshtha *et al.*, 2021^60^ |  |  |  |  |  |  |  |  |  |  |  |  |
| Silistraru *et al.,* 2022^61^ |  |  |  |  |  |  |  |  |  |  |  |  |
| Tee *et al.,* 2022^62^ |  |  |  |  |  |  |  |  |  |  |  |  |
| Toubasi *et al.,* 2022^63^ |  |  |  |  |  |  |  |  |  |  |  |  |
| Wang J *et al.,* 2021^64^ |  |  |  |  |  |  |  |  |  |  |  |  |
| Yahya *et al.,* 2021^65^ |  |  |  |  |  |  |  |  |  |  |  |  |
| Zhang *et al.,* 2021^66^ |  |  |  |  |  |  |  |  |  |  |  |  |
| Zis *et al*., 2021^67^ |  |  |  |  |  |  |  |  |  |  |  |  |
| Žuljević *et al.,* 2021^68^ |  |  |  |  |  |  |  |  |  |  |  |  |
| Zuniga *et al.,* 2021^69^ |  |  |  |  |  |  |  |  |  |  |  |  |

Study was marked as high risk of bias if data was not reported

Red: High risk of bias

Green: Low risk of bias

^#^Funded by FEDER/Consejería de Universidad, Investigación e Inovación de la Junta de Andalucía. Project P20-00637.

^$^JNK has received honorarium from Lundbeck Pharma A/S and Otsuka Pharma Scandinavia AB.

Q1. Was the study's target population a close representation of the national population in relation to relevant variables, e.g. age, sex, occupation?

Q2. Was the sampling frame a true or close representation of the target population?

Q3. Was some form of random selection used to select the sample, OR was a census undertaken?

Q4. Was the likelihood of non-response bias minimal?

Q5. Were data collected directly from the subjects (as opposed to a proxy)

Q6. Was an acceptable case definition used in the study?

Q7. Was the study instrument that measured the parameter of interest (e.g. prevalence of low back pain) shown to have reliability and validity(if necessary)?

Q8. Was the same mode of data collection used for all subjects?

Q9. Was the length of the shortest prevalence period for the parameter of interest appropriate?

Q10. Were the numerator(s) and denominator(s) for the parameter of interest appropriate?

COI- If Conflict of Interest was declared it was considered low risk of bias

Funding- If funding details were reported, it was considered low risk of bias

**Supplementary Box 3: Synthesis of prevalence of overall burnout and other instruments**

Overall burnout for MBI, ECE and OLBI

*Geographical patterns and fields of study*

Most studies (24/31, 77.4%) specified the prevalence of overall burnout (OB) (Supplementary Table 6). The reported OB prevalence among medical students ranged from 0.5% in Belgium^40^-68.0% in France^56^ (Supplementary Table 6). OB prevalence across all disciplines of study ranged from 29.0% in Portugal^58^-30.5% in Saudi Arabia^30^. Only one Spanish study reported prevalence among pharmacy students^48^ (63.5%).

*Sex*

Overall burnout stratified by sex was reported in four countries: India^51^, Mexico^43^, Saudi Arabia^30^ and Uganda^45^ (Supplementary Table 6). Male and female ranges for OB were 19.05-70.9% and 7.5-29.1% respectively.

OB time trends across all instruments

Only four studies reported OB prevalence before and during COVID-19 pandemic (Supplementary Tables 6, 7 and 8). OB decreased during the pandemic in Kazakhstan^34^ from 27.6-16.7% (as measured with the CBI^34^) and in Croatia^68^ from 51.5-45.7% (as measured with the OLBI). However, it remained the same in Cyprus (using the MBI-SS)^67^. Ruiz *et al.*^57^ documented a rise in OB prevalence (from 5.0-13.6%) using the MBI-SS. Only Ruiz *et al.* reported a statistically significant difference (p =0.000137).

Other instruments

Three studies used the CBI (Supplementary Table 8). Six studies used one of the following instruments: Learning Burnout Inventory, Burnout Clinical Subtype Questionnaire Students Survey, Link Burnout Questionnaire, Academic Burnout Scale, Physician Work life Survey and a de novo instrument (Supplementary Table 9).

CBI domains

Three studies conducted on medical students in Croatia, Malaysia and Kazakhstan used a version of the CBI (Supplementary Table 9). Bolatov *et al*., 2021^34^ used the student version, which has four domains: personal burnout, studies-related burnout, colleague-related burnout and teacher-related burnout. Žuljević *et al.,* 2021^68^ modified the CBI, resulting in five domains: personal burnout, studies-related burnout, professor-related burnout, student-related burnout, and patient-related burnout. Tee *et al.*, 2022^62^ used the standard CBI, and reported overall burnout (22.2%).

The prevalence of personal-related burnout varied between 28.2-31.2%, and studies-related burnout varied between 34.0-48.1%. For the remaining domains (professor-related burnout, student-related burnout, patient-related burnout, colleague-related burnout, and teacher-related burnout) only one of the two studies reported prevalence. Both primary studies were conducted in medical students. Žuljević *et al*. 2021^68^ reported sex stratified prevalence data.

Both studies reported time trends (pre-COVID-19 vs during COVID-19) by domain. Bolatov *et al*., 2021^34^ reported a decrease in personal-, studies- and teacher-related burnout, and a slight increase in colleague-related burnout after the onset of the COVID-19 pandemic. Žuljević *et al*., 2021^68^ reported an increase in patient- and student-related burnout, and a decrease in professor-, personal-and studies-related burnout.

**Supplementary Table 11: Studies included in the SR but excluded from the MA**

| **No** | **Citation** | **Reason for Exclusion** |
| --- | --- | --- |
| 1 | Alsaad et al., 2021^30^ | Prevalence for domains not provided |
| 2 | Azzi et al., 2022a^32^ | Too few studies used this tool to be included in the MA |
| 3 | Azzi et al., 2022b^33^ | Cut-offs used were too different compared to other studies |
| 4 | Bolatov et al., 2021^34^ | Too few studies used this tool to be included in the MA |
| 5 | Chi et al, 2021^35^ | Too few studies used this tool to be included in the MA |
| 6 | Dlugosz et al., 2021^39^ | Too few studies used this tool to be included in the MA |
| 7 | Nurhidayati et al., 2021^50^ | Too few studies used this tool to be included in the MA |
| 8 | Periasamy et al., 2021^51^ | Prevalence for domains not provided |
| 9 | Ramos et al., 2021^53^ | Too few studies used this tool to be included in the MA |
| 10 | Remitha et al., 2020^54^ | Too few studies used this tool to be included in the MA |
| 11 | Rohmani et al., 2021^55^ | Prevalence for domains not provided |
| 12 | Rolland et al., 2022^56^ | Prevalence for domains not provided |
| 13 | Silistraru et al., 2022^61^ | Prevalence for domains not provided |
| 14 | Tee et al., 2022^62^ | Too few studies used this tool to be included in the MA |
| 15 | Wang J et al., 2021^64^ | Too few studies used this tool to be included in the MA |
| 16 | Tee et al., 2022^62^ | Too few studies used this tool to be included in the MA |
| 17 | Toubasi et al., 2022^63^ | Cut-offs used were too different compared to other studies |
| 18 | Zhang et al., 2021^66^ | Too few studies used this tool to be included in the MA |
| 19 | Zis et al., 2021^67^ | Prevalence for domains not provided |
| 20 | Zuniga et al., 2021^69^ | Prevalence for domains not provided |

**Supplementary Table 12: Subgroup analysis by instrument (MBI-ECE-OLBI), response rate and means of data collection**

|  | Number of prevalence measures | | Total sample size | Prevalence range (%) | | | Effect size | | Subgroup comparison (Q between subgroup tests p-value) | Heterogeneity between studies I^2^ (%) |
| --- | --- | --- | --- | --- | --- | --- | --- | --- | --- | --- |
|  |  |  |  |  |  |  | Weighted average prevalence (%) | 95%CI |  |  |
| **MBI-ECE-OLBI-EE-High** | | | | | | | | | | |
| **Instrument** | | | | | | | | | | |
| MBI | 12 | | 5467 | 14.4-93.1 | | | 49.0 | 32.0-66.2 | <0.01 | 99 |
| aMBI | 2 | | 336 | 14.2-46.3 | | | 27.1 | 10.5-54.0 |  | 97 |
| ECE | 3 | | 921 | 12.6-65.7 | | | 36.2 | 14.5-65.4 |  | 99 |
| OLBI | 7 | | 3079 | 66.9-95.1 | | | 80.9 | 72.7-87.1 |  | 93 |
| **RR** | | | | | | | | | | |
| RR=<75 | 11 | | 4716 | 12.6-95.1 | | | 55.7 | 31.2-77.6 | 0.92 | 99 |
| RR= NR | 13 | | 5087 | 21.6-80.6 | | | 56.3 | 44.7-68.7 |  | 98 |
| **Means of Data Collection** | | | | | | | | | | |
| Online | 21 | | 9415 | 12.6-95.1 | | | 59.5 | 45.0-72.6 | <0.01 | 99 |
| NR | 3 | | 388 | 21.6-44.7 | | | 33.1 | 23.2-44.7 |  | 83 |
| **MBI-ECE-OLBI-DP/CY-High** | | | | | | | | | | |
| **Instrument** | | | | | | | | | | |
| MBI | 12 | | 5467 | | 11.0-97.2 | 48.4 | | 28.0-69.3 | <0.01 | 99 |
| aMBI | 2 | | 336 | | 3.1-23.2 | 8.9 | | 2.0-31.9 |  | 96 |
| OLBI | 7 | | 3079 | | 52.3-93.2 | 80.0 | | 69.8-87.4 |  | 97 |
| **RR** | | | | | | | | | | |
| RR=<75 | 10 | | 4375 | | 3.1-97.2 | 57.4 | | 30.0-80.9 | 0.83 | 99 |
| RR= NR | 11 | | 4507 | | 11.0-93.2 | 53.4 | | 32.0-73.6 |  | 99 |
| **Means of Data Collection** | | | | | | | | | | |
| Online | 18 | | 8494 | 3.1-97.2 | | | 60.5 | 41.2-77.1 | <0.01 | 99 |
| NR | 3 | | 388 | 16.9-42.6 | | | 24.4 | 15.0-37.2 |  | 86 |
| **MBI-ECE-OLBI-PA/AE-Low** | | | | | | | | | | |
| **Instrument** | | | | | | | | | | |
| MBI | 10 | 5106 | | 4.4-91.7 | | 39.8 | | 19.9-63.8 | 0.08 | 99 |
| aMBI | 1 | 254 | | 62.2 | | 62.2 | | 55.9-68.2 |  | NA |
| **RR** | | | | | | | | | | |
| RR=<75 | 7 | 3671 | | 4.4-91.7 | | 43.2 | | 19.2-70.9 | 0.87 | 99 |
| RR= NR | 4 | 1689 | | 8.3-79.5 | | 39.4 | | 13.6-72.9 |  | 99 |
| **Means of Data Collection** | | | | | | | | | | |
| Online | 9 | | 5179 | 4.4-91.7 | | | 41.9 | 19.7-68.0 | 0.94 | 99 |
| NR | 2 | | 181 | 25.4-59.6 | | | 40.6 | 19.7-65.6 |  | 94 |

MBI-Maslach Burnout Inventory; aMBI- abbreviated Maslach Burnout Inventory; ECE-Emotional Exhaustion Scale; OLBI-Oldenburg Burnout Inventory; EE-emotional exhaustion; DP-Depersonalization; PA-Personal Accomplishment; CY—cynicism; AE – academic efficacy; RR- Response rate

**Supplementary Table 13: Global pooled prevalence of high emotional exhaustion among university students by country, measured with the Maslach Burnout Inventory, the Emotional Exhaustion Scale, and the Oldenburg Burnout Inventory**

|  | Number of prevalence measures | Total sample size | Prevalence range (%) | Effect size | | Subgroup comparison (Q between subgroup tests p-value) | Heterogeneity between studies I^2^ (%) |
| --- | --- | --- | --- | --- | --- | --- | --- |
|  |  |  |  | Weighted average prevalence (%) | 95%CI |  |  |
| **Country** | | | | | | | |
| Belgium | 1 | 145 | 23.4 | 23.4 | 16.8-31.2 | 0.00 | NA |
| Croatia | 1 | 199 | 76.4 | 76.4 | 69.9-82.1 |  | NA |
| Chile | 1 | 315 | 65.7 | 65.7 | 60.2-70.9 |  | NA |
| Denmark | 1 | 387 | 66.9 | 66.9 | 62.0-71.6 |  | NA |
| Guatemala | 1 | 132 | 14.4 | 14.4 | 8.9-21.6 |  | NA |
| India | 1 | 341 | 80.6 | 80.6 | 76.0-84.7 |  | NA |
| Indonesia | 1 | 1947 | 31.2 | 31.2 | 29.1-33.3 |  | NA |
| Iraq | 1 | 424 | 85.6 | 85.6 | 81.9-88.8 |  | NA |
| Mexico | 1 | 154 | 53.9 | 53.9 | 45.7-61.9 |  | NA |
| Nigeria | 1 | 505 | 77.0 | 77.0 | 73.1-80.6 |  | NA |
| Peru | 2 | 606 | 12.6-39.6 | 23.6 | 9.7-46.9 |  | 98 |
| Poland | 1 | 1363 | 69.8 | 69.8 | 67.3-72.3 |  | NA |
| Portugal | 1 | 207 | 37.2 | 37.2 | 30.6-44.2 |  | NA |
| Russia | 1 | 165 | 80.0 | 80.0 | 73.1-85.8 |  | NA |
| Saudi Arabia | 2 | 769 | 29.5-69.5 | 49.4 | 23.1-76.1 |  | 99 |
| Sri Lanka | 1 | 1097 | 79.0 | 79.0 | 76.5-81.4 |  | NA |
| Spain | 2 | 181 | 21.6-44.7 | 31.0 | 17.5-48.7 |  | 89 |
| Uganda | 1 | 145 | 93.1 | 93.1 | 87.7-96.6 |  | NA |
| United Arab Emirates | 1 | 385 | 95.1 | 95.1 | 92.4-97.0 |  | NA |
| United Kingdom | 1 | 82 | 46.3 | 46.3 | 35.3-57.7 |  | NA |
| United States of America | 1 | 254 | 14.2 | 14.2 | 10.1-19.1 |  | NA |

EE-Emotional Exhaustion; NR-Not reported; NA-Not applicable; COVID-19- Corona Virus Disease of 2019

**Supplementary Table 14: Global pooled prevalence of high depersonalization / cynicism among university students by country, measured with the Maslach Burnout Inventory, the Emotional Exhaustion Scale and the Oldenburg Burnout Inventory**

|  | Number of prevalence measures | Total sample size | Prevalence range (%) | Effect size | | Subgroup comparison (Q between subgroup tests p-value) | Heterogeneity between studies I^2^ (%) |
| --- | --- | --- | --- | --- | --- | --- | --- |
|  |  |  |  | Weighted average prevalence (%) | 95%CI |  |  |
| **Country** | | | | | | | |
| Belgium | 1 | 145 | 11.0 | 11.0 | 6.4-17.3 | 0.00 | NA |
| Croatia | 1 | 199 | 52.3 | 52.3 | 45.1-59.4 |  | NA |
| Denmark | 1 | 387 | 72.1 | 72.1 | 67.3-76.5 |  | NA |
| Guatemala | 1 | 132 | 77.3 | 77.3 | 69.2-84.1 |  | NA |
| India | 1 | 341 | 86.2 | 86.2 | 82.1-89.7 |  | NA |
| Indonesia | 1 | 1947 | 30.7 | 30.7 | 28.6-32.8 |  | NA |
| Iraq | 1 | 424 | 77.8 | 77.8 | 73.6-81.7 |  | NA |
| Mexico | 1 | 154 | 16.9 | 16.9 | 11.3-23.8 |  | NA |
| Nigeria | 1 | 505 | 84.6 | 84.6 | 81.1-87.6 |  | NA |
| Poland | 1 | 1363 | 67.8 | 67.8 | 65.2-70.3 |  | NA |
| Portugal | 1 | 207 | 16.9 | 16.9 | 12.1-22.7 |  | NA |
| Russia | 1 | 165 | 73.3 | 73.3 | 65.9-79.9 |  | NA |
| Saudi Arabia | 2 | 769 | 33.3-73.7 | 54.2 | 26.4-79.7 |  | 99 |
| Sri Lanka | 1 | 1097 | 93.2 | 93.2 | 91.5-94.6 |  | NA |
| Spain | 2 | 181 | 20.9-42.6 | 29.6 | 17.0-46.3 |  | 88 |
| Uganda | 1 | 145 | 97.2 | 97.2 | 93.1-99.2 |  | NA |
| United Arab Emirates | 1 | 385 | 81.3 | 81.3 | 77.0-85.1 |  | NA |
| United Kingdom | 1 | 82 | 23.2 | 23.2 | 14.6-33.8 |  | NA |
| United States of America | 1 | 254 | 3.1 | 3.1 | 1.4-6.1 |  | NA |

CY-Cynicism; DP-Depersonalization; NR- Not reported; NA-Not applicable; COVID-19- Corona Virus Disease of 2019

**Supplementary Table 15: Global pooled prevalence of low personal accomplishment / academic efficacy among university students by country, measured with the Maslach Burnout Inventory and the Emotional Exhaustion Scale**

|  | Number of prevalence measures | Total sample size | Prevalence range (%) | Effect size | | Subgroup comparison (Q between subgroup tests p-value) | Heterogeneity between studies I^2^ (%) |
| --- | --- | --- | --- | --- | --- | --- | --- |
|  |  |  |  | Weighted average prevalence (%) | 95%CI |  |  |
| **Country** | | | | | | | |
| Belgium | 1 | 145 | 8.3 | 8.3 | 4.3-14.0 | <0.01 | NA |
| Guatemala | 1 | 132 | 91.7 | 91.7 | 85.6-95.8 |  | NA |
| Indonesia | 1 | 1947 | 66.7 | 66.7 | 64.6-68.8 |  | NA |
| Iraq | 1 | 424 | 32.5 | 32.5 | 28.1-37.2 |  | NA |
| Poland | 1 | 1363 | 79.5 | 79.5 | 77.3-81.6 |  | NA |
| Saudi Arabia | 2 | 769 | 4.4-31.8 | 12.8 | 2.8-42.7 |  | 99 |
| Spain | 2 | 181 | 25.4-59.6 | 40.6 | 19.7-65.6 |  | 94 |
| Uganda | 1 | 145 | 29.0 | 29.0 | 21.7-37.1 |  | NA |
| United States of America | 1 | 254 | 62.2 | 62.2 | 55.9-68.2 |  | NA |

AE-Academic Efficacy; PA-Personal Accomplishment; NA-Not applicable;

COVID-19- Corona Virus Disease of 2019

**Supplementary Table 16: Study-specific research gaps and recommendations**

| **Citation** | **Study Period** | **Country** | **Instrument Used** | **Research Gaps** | **Recommendations** |
| --- | --- | --- | --- | --- | --- |
| Abdel Aziz et al., 2023^27^ | Apr – Jun 2022 | United Arab Emirates | OLBI | We recommend that future studies have a longitudinal, prospective design as this would allow for more accurate investigation of causal relationships and would also help reduce any potential bias as the method used in this study to determine when mental health conditions were diagnosed might be prone to recall bias. | NR |
| Aguayo-Estremera et al., 2023^28^ | 1^st^ quarter of 2021 | Spain | MBI-SS | It would be desirable to carry out future research with students from other regions and countries to increase the generalizability of the results. Future studies could test confirmatory models based on the results of this study combining a well-defined theoretical framework. it would be desirable to carry out new studies with longitudinal designs, which are appropriate for this type of objectives. Future research with a larger sample size could further investigate this topic. | NR |
| Alqifari et al., 2021^29^ | Mar 2-23, 2020 | Saudi Arabia | MBI-SS | Longitudinal research is essential to investigate the burnout trend of medical students from entry to graduation.  Other factors suggested to be studied include the association between burnout and social factors, and psychological and biological illnesses among medical students. | No change in medical education style is needed to reduce burnout, based on our study results.  Implementing strategies that might decrease burnout are recommended, including problem-solving, constructive reinterpretation, and emotional expression. Additional recommendations include establishing student-led services to encourage senior students’ mentorship of junior students and encouraging students to get regular physical exercise and enough sleep to improve their wellbeing |
| Alsaad et al., 2021^30^ | Jul - Sep 2020 | Saudi Arabia | MBI-HSS | NR | NR |
| Ayinde et al., 2022^31^ | May - Aug 2020 | Nigeria | OLBI | NR | NR |
| Azzi et al, 2022a^32^ | Aug 7-26, 2020 | Brazil | BCSQ-12-SS | Future studies should consider the perspective of COVID-19 vaccination and return to face-to-face activities on burnout. | NR |
| Azzi et al, 2022b^33^ | Feb 2-21, 2020; Sep 21- Oct 17, 2020 | Brazil | MBI-SS | NR | Thus, the inclusion of educational policies that encourage the practice of physical activities in agricultural sciences students can be a favorable strategy to avoid academic burnout. Maintaining some academic content with online or hybrid activities (online theoretical classes and in-person practical activities) in post-pandemic. |
| Bolatov et al., 2021^34^ | Phase 1: Oct - Nov 2019  Phase 2: Apr 13-19, 2020 | Kazakhstan | CBI-S | NR | NR |
|  |  |  |  |  |  |
|  |  |  |  |  |  |
|  |  |  |  |  |  |
|  |  |  |  |  |  |
| **Citation** | **Study Period** | **Country** | **Instrument Used** | **Research Gaps** | **Recommendations** |
| Chi et al, 201^35^ | Aug 19 - Sep 15, 2020 | USA | PWS | Longitudinal research is essential to investigate the burnout trend of medical students from entry to graduation. | There are several interventional approaches that could help address the unmet mental health care needs of dental trainees. The first is to make professional resources available and to remove barriers to use. More than 70% of participants had never used resources such as counseling services or psychological care. Accessing resources can be a challenge for dental students with inflexible schedules, which reinforces the need to make such resources available outside of training hours and in close physical proximity to the school to reduce the related barrier of time-consuming commutes. Some students may have copayments or deductibles, which highlights the need for dental schools to eliminate cost related barriers to care of trainees. In addition, owing to the embarrassment and stigma associated with accessing services and potential concerns about confidentiality, especially among health science students, services may need to be provided outside of the physical dental school space. Another approach to addressing unmet mental healthcare needs amongst dental trainees is to provide comprehensive wellness programming to all trainees. Such a process should include screening and referrals for clinical depression and anxiety, monitoring for burnout and ensuring that students are engaged with school. Trainee engagement should be enhanced with opportunities for extracurricular school activities social activities to address isolation (46.8% of survey respondents reported feeling isolated from their peers), service learning and public health outreach and research. Universal wellness programming that teaches trainees evidence based strategies (for example cognitive behavioral techniques) for stress management may be helpful especially if delivered early in training and reinforced regularly. Wellness initiatives might also include the universal, consistent provision of formal opportunities for trainees to convey acute financial educational and technological needs, such as library tutoring services, internet access or hardware needs, all of which were amongst the most common unmet needs for survey participants during the COVID-19 pandemic. |
| Chumakov et al., 2021^36^ | May - Jun, 2020 | Russia | OLBI | Further studies are required to assess the possible causes of these cultural differences (lower rates among Russian students compare to other countries), as well as the socio-cultural factors potentially associated with them. Therefore, it would be useful to conduct the study in dynamics after the removal of all social restrictions. Also, it would have been interesting and useful to conduct a comparison with students from other disciplines such as psychology, social workers students, and dentistry students. | NR |
| Cipta et al., 2022^37^ | Apr – May 2021 | Indonesia | MBI-SS | We suggest further research to identify specific factors contributing to burnout and develop appropriate interventions at the personal, commu- nity and organizational levels. A qualitative approach will also help us measure the right thing concerning the burn- out phenomenon. | It is essential to remind curriculum developers that medical students are generally a high-risk population for developing burnout and other mental disor- ders and have less flexibility in scheduling than their coun- terparts from other academic majors |
| Dahanayake et al., 2022  ^38^ | Jul – Aug 2020 | Sri Lanka | OLBI | NR | NR |
| Dlugosz et al., 2021^39^ | Jun 1-10, 2020 | Poland | LBQ | NR | NR |
| El Mouedden et al., 2022^40^ | Apr 22 -May 4, 2020 | Belgium | MBI-SS | Further research may wish to examine whether specific tasks (e.g., triage, administrative) are associated differently with different burnout dimensions | NR |
| **Citation** | **Study Period** | **Country** | **Instrument Used** | **Research Gaps** | **Recommendations** |
| Estrada Araoz, 2021^41^ | Jun 2021 | Peru | ECE | Future studies increase the number of participants and include students from other universities so that the results can be generalized. | Students should be encouraged to develop coping strategies for stressful situations, typical of academic life and the context of the pandemic. |
| Forycka et al., 2022^42^ | Jan 5 - Feb 6, 2021 | Poland | MBI-SS | NR | Our study should encourage both faculty and student organizations to develop proper resilience-building strategies. Besides, there is a strong need to support students’ mental health and to monitor students’ well-being during the time of recovery from the pandemic. Today’s students are the physicians of tomorrow and their ability to adapt to challenges is crucial for effective and efficient patient care in the future. |
| Jezzini-Martinez et al, 2022^43^ | Jun 22-26, 2020 | Mexico | MBI-SS | NR | Universities need to provide guidance of support alternatives to improve mental health due to the impact this syndrome may cause on the quality of life. The Department of Psychiatry started to receive free-of-charge all medical students who wanted counseling through a hybrid method. Students could decide between virtual or face-to-face. With the beginning of the COVID-19 pandemic, counseling needed to be adapted to an online format that benefits the students. It is important universities encourage their alumni to exercise and obtain quality sleep, as both have been associated with prevention and reduced levels of burnout. In a university with over 7,000 students total, mental health programs need to be increased and adapted to social distancing norms. Further epidemiological studies of burnout syndrome in medical students are needed, and  Schools should consider promoting mental health and making programs available for their students to help overcome the emotional and social challenges during the pandemic. |
| Johns et al., 2022^44^ | Sep 27, 2020 - Jan 31, 2021 | UK | aMBI | NR | NR |
| Kajjimu et al., 2021^45^ | Sep 6-20, 2020 | Uganda | MBI-SS | There is a need for a multi-center, prospective longitudinal study. | There needs to be an investment in the provision of mental health support to medical students through interventions to raise awareness, understanding, and prevention of the development of psychological distress, burnout, and mental health disorders like depression and anxiety among medical students. |
|  |  |  |  |  |  |
|  |  |  |  |  |  |
|  |  |  |  |  |  |
| **Citation** | **Study Period** | **Country** | **Instrument Used** | **Research Gaps** | **Recommendations** |
| Khalafallah et al., 2021^46^ | 2 weeks in May 2020 | USA | aMBI | NR | One intervention that has been proposed includes resilience training to help students cope with difficult team interactions, find meaning in their work, and deal with disappointment and setbacks. Mindfulness-based interventions have also been successful and have been reported to decrease rates of depression, stress, and burnout in medical students. Other studies have demonstrated that conversion to a pass/fail grading system, communications exercises, and relaxation exercises are also linked to decreased burnout. It will be essential to continue to study these interventions and convert them to online formats as medical students continue to face burnout during the pandemic. Numerous solutions have also been proposed to ensure the continued recruitment of medical students to neurosurgery and decrease student anxieties related to the neurosurgery residency application. Studies have demonstrated that neurosurgery interest groups, strong mentorship, earlier preclinical exposure, and research opportunities are essential in matching more students into neurosurgery. Other studies have demonstrated the efficacy of programs such as small conferences or neurosurgery training camps in educating early-career medical students on the lifestyle of neurosurgery and sur- gical skills and recruiting medical students to the field. Similar programs should be implemented during and after the pandemic in order to decrease student uncertainty about the field and support those students who are unsure about pursuing a career in neurosurgery, as these students are also likely to experience burnout. Lastly, interventions related to the residency application cycle have been proposed, such as having programs organize online question-and-answer sessions for interested applicants, alter letter of recommendation requirements for applicants, or organize virtual sub-internships and rotations. Interventions targeted to these students and interventions designed to decrease stress related to the residency application process will be necessary to ensure student wellness. |
| Kjær et al., 2022  ^47^ | February 21 - August 17, 2020 | Denmark | OLBI | Further study is required to ascertain the antecedents of our findings to look at what support would be most beneficial to reduce psychological distress and burnout for medical students. | NR |
| Martinez-Libano et al., 2021^19^ | Nov 2020 | Chile | ECE | More has to be done to study how the transition from face-to-face to online classes affects mental health since such a sudden transition could have harmful consequences for students, especially for those in vulnerable situations. | It is essential that the authorities urgently seek solutions to mitigate the psychological impact of home confinement. Universities have a responsibility to advocate for students, especially for those students who are vulnerable. Fostering resilience is a promising way to mitigate the adverse effects of stressors, prevent burnout and help students thrive after difficult experiences. |
| Moreno-Fernandez et al., 2020^48^ | During confienement period | Spain | MBI-SS | It would be necessary to study burnout differentiating by gender, increasing the population of the study and in different faculties and academic degrees to find indices that allow contrasting these results. Additionally, a longer intervention would be desirable to translate the knowledge of EI into usual behavior, especially after the confinement. | It is also important to design programs aimed at strengthening teaching-learning processes, as well as intervention actions aimed at reducing burnout, especially in students with lower academic results. |
| Muaddi et al., 2023^49^ | Oct – Nov 2020 | Saudi Arabia | MBI-SS | Future studies on burnout syndrome should aim to replicate this study in other medical schools and at different times to determine the generalizability of the findings and to identify factors that may be unique to different populations of medical students. Furthermore, studies can be carried out to explore the effectiveness of various interventions for reducing burnout in medical students and to establish best practices for promoting their well-being. | Developing screening measures and interventional strategies, especially during the transition from pre-clinical to clinical work, to combat burnout and promote and enhance the ability to cope with stressful lifestyles in our future physicians is a paramount priority to ensure their health and professional well-being. Therefore, we propose integrating these strategies into the medical school curriculum as a component of the medical training based on the weight of evidence about burnout in physicians-in-training. |
| Nurhidayati et al., 2021^50^ | Jun 2020 | Indonesia | Burnout scale* | NR | Severity of burnout syndrome can be decreased either personally or by changing the application within the organization in which tasks should be done. In organization, the prevention for burnout could be done by defining clear task statements, beginner participation on orientation program and job training, efficient personal planning related to the department, regulate team gathering which involves suggestion and critic delivery session, and access to paticipative social and society support. An academic advisor should be able to provide counselling and give solutions to students' problems. |
| **Citation** | **Study Period** | **Country** | **Instrument Used** | **Research Gaps** | **Recommendations** |
| Periasamy et al., 2021^51^ | Jun 2021 | India | MBI-SS | We recommend burnout be longitudinally assessed once a year in all medical students to determine the effectiveness of wellness initiatives and make modifications as appropriate. | The implementation of effective wellness initiatives may be sufficient for ameliorating burnout among medical students.. Burnout is a deleterious issue that can negatively impact the healthcare community. Early identification and resolution of the phenomenon can help improve the health outcomes of patients receiving care from medical students and residents progressing through their education. |
| Philip et al., 2021^52^ | Feb 9 - Aug 31, 2020 | India | OLBI | NR | We need to urgently develop initiatives to minimise morbidity and maximise an individual’s well-being and medical career. 1) Emphasising the importance of well-being both in and away from the workplace. 2) Increasing the amount of targeted support around mental health from medical educators/institutions. 3) Stigma reduction and attitude changes that will allow a positive platform on which well-being and mental health optimization and/or treatment can be accessed |
| Ramos et al., 2021^53^ | 3 weeks during the COVID-19 pandemic | Philippines | de novo tool** | NR | Prevention and interventional measures are needed to help students cope with online learning and burnout. Academic resilience is necessary to counteract burnout in terms of the student relationships with their teachers and classmates and satisfaction with their capacity to perform academically |
| Remitha et al., 2020^54^ | Jun - Aug 2020 | Indonesia | Burnout scale*** | Further research is needed to determine other factors that influence the incidence of burnout. | The high burnout prevalence shows the need for a teaching method that does not worsen the workload of medical students during online lectures. |
| Rohmani et al., 2021^55^ | Sep 2020 | Indonesia | MBI-SS | NR | Education providers and managers should make serious efforts to improve students’ academic self-efficacy by involving both lecturers and educational staff to create a supportive academic environment. Innovations in learning should also be made to reduce students’ burnout levels during online learning. |
| Rolland et al., 2022^56^ | May 27 -Jun 27, 2021 | France | MBI-SS & MBI-HSS | NR | NR |
| Ruiz et al., 2022^57^ | Phase 1: 2017  Phase 2: 2020 | Guatemala | MBI-SS | Further exploration of factors such as parental expectations, curricular pressures, relationship, accommodation, and financial difficulties need to be ascertained in order to develop and deliver interventions to reduce rates of burnout. | NR |
|  |  |  |  |  |  |
|  |  |  |  |  |  |
|  |  |  |  |  |  |
|  |  |  |  |  |  |
|  |  |  |  |  |  |
| **Citation** | **Study Period** | **Country** | **Instrument Used** | **Research Gaps** | **Recommendations** |
| Salgado et al., 2021^58^ | Mar 30 – Apr 30, 2020 | Portugal | MBI-SS | NR | Reformulation of teaching methods, creating new methods of assessment (not just exams and/or tests) and promoting dynamic classes to stimulate students’ creativity and motivation (taking, for example, occasional classes outdoors or with music); The reduction in the number of hours of daily and weekly classes;  Provide a wider range of curriculum options so that students can study what they like, and think is appropriate for their vocational pathways; Providing more free workshops to learn how to deal with anxiety, pressure, stress, time management, as well as sessions on emotional intelligence and resilience aimed at the entire academic community; Reduction of waiting lists for psychology appointments; Greater dissemination of mental illness and psychology consultations; Make the exam schedule available at the beginning of the semester, so that students can choose the assessment method in an organized manner and reduce students’ stress and anxiety during the assessment period; Greater coordination by the university’s Rectory to avoid accumulating exams on the same day; Have a lower load of group work and, on the other hand, more individual work, since it was an aspect mentioned by the students; The reduction in the number of students per class in order to promote greater teacher– student interaction; Reformulation of the program of most curricular units, giving more importance to the practical aspect of teaching, the promotion of short-term paid internships and, in general, the preparation of students for the imperfections of the working world (which is the main mission of teaching at university). In the same sense, the courses could be divided into two phases: one held at the university and the other at a company (having access to real problems), with the company paying students for their time, knowledge and availability or, alternatively, paying for their tuition fees (as occurs in Germany). This measure would increase the students’ intrinsic motivation, which, as verified in the present study, is a burnout protection factor; Consideration of student feedback regarding the topics taught and the topics not taught that may be really relevant to them; Creation of an extracurricular physical exercise activity to promote individual and team work to relieve stress and promote interpersonal relationships; Improving the comfort of leisure spaces and increasing the number of social spaces to relieve stress and promote interpersonal relationships; Improving material conditions to increase comfort in classrooms, as material condi- tions have an influence on burnout; Individual and group orientation programs as suggested by the authors; If the success of higher education institutions and their students is related to the well-being of teachers, it is crucial to analyze the predominance of stress and burnout in this profession to understand the problems and some of the causes behind stress and implement measures |
| Seperak-Viera et al., 2021^59^ | July 12 -Aug 9, 2020 | Peru | ECE | NR | NR |
|  |  |  |  |  |  |
|  |  |  |  |  |  |
| **Citation** | **Study Period** | **Country** | **Instrument Used** | **Research Gaps** | **Recommendations** |
| Shreshtha et al., 2021^60^ | Jan 14 -Mar 7, 2021 | Nepal | OLBI-S | A longitudinal study of the same batch of students throughout their 1st to 5th year of study would give a clearer picture of the trend of burnout in different academic years.  Further multicenter prospective studies are required for a better understanding of the prevalence and associated factors of burnout.  It is also recommended to adapt and validate screening tools for use in the Nepali population in the future. | At an individual level, students should be encouraged to incorporate various self-care techniques into their daily lives such as a nutritious diet, regular exercise, restful sleep, making a balance between study and leisure, the practice of self-compassion, and being aware of their emotional needs. Similarly, positive coping and hobbies help to overcome the stressors of medical studies. Some common practices can be mindfulness, yoga, listening to music, reading books, and outdoor games requiring group engagement. Various measures can be taken by medical institutions and governing bodies to create a nurturing, learning environment, teach skills for stress management and promote self-awareness. Adjustment in the curriculum and teaching methods can be made to reduce distress among medical students. A trustworthy and stigma-free environment should be built for medical students and health care professionals. They should also be encouraged to seek professional counseling or therapy when in need. |
| Silistraru et al., 2022^61^ | Dec 2020 -Apr 2021 | Romania | MBI-SS | A longitudinal study could provide more information on burnout scores in the student population, monitored throughout the entire period of study. | Following up on the current research on burnout will allow universities to build a collection of burnout best practices and mental health interventions. |
| Tee et al., 2022^62^ | May – Jul 2021 | Malaysia | CBI | NR | It is important to provide students with a holistic medical education emphasizing positive coping mechanisms and building resilience, on top of competency in the clinical skills. Therefore, medical schools could tackle the stigma attached to mental health problems and the barriers to seeking help by educating faculty staff about the confidentiality policies and procedures, and by monitoring and responding to reports of discrimination due to mental illness. An increased sense of personal accomplishment was found after the implementation of a mentorship program. |
| Toubasi et al., 2022^63^ | May 26 – Sep 25, 2021 | Jordan | MBI-SS | We recommend carrying out further well conducted and high-quality clinical trials to assess the effectiveness of the aforementioned potential interventions in reducing burnout among university students | Institutions are recommended to address the factors that were associated with burnout or its components such as hobbies and hours spent in attending lectures. Necessitates the use of validated tools in screening for burnout to accurately assess burnout. We recommend the implementation of combined methods that proved to be effective in the reduction of burnout among university students, such as mindfulness programs. In addition,. |
| Wang J et al., 2021^64^ | Apr -Jun 2020 | China | ABS | NR | Given the above findings, we suggest that educators pay attention to academic burnout of nursing students in traditional Chinese medicine universities. Measures focused on strengthening nursing students psychological capital and academic engagement are effective for reducing their burnout. |
| Yahya et al., 2021^65^ | Jun 11 – Jul 3, 2020 | Iraq | MBI-SS | NR | Faculty must focus on altering improving the academic and clinical conditions, context, and curricula to reduce unnecessary stresses and create more favorable teaching and clinical practice. As well as that professors need to understand the students’ needs, motivations, and experiences to keep students interested in education and to minimize their burnout rates. |
| Zhang et al., 2021^66^ | Apr 2020 | China | LBS | NR | NR |
|  |  |  |  |  |  |
| **Citation** | **Study Period** | **Country** | **Instrument Used** | **Research Gaps** | **Recommendations** |
| Zis et al., 2021^67^ | Phase 1: Jan 2020  Phase 2: May-20 | Cyprus | MBI-SS | NR | Our findings are useful, particularly for training program directors, as they make it clear that at least for medical students in their last year, medical training should not be virtual. Moreover, our study showed that medical students in their first year of clinical training (in our school, year 4 of medical studies) are significantly stressed and were relieved when clinical training was discontinued. This suggests that the training program could be revised. Reduced hours in the clinical setting in the first clinical year might have a positive effect on reducing the stressors for those students. Moreover, all medical schools should have easily accessible medical student mental health services. Some schools of medicine provide such services through departments of psychiatry or other associated training programs (i.e., psychology). Since this stressful lifestyle often continues through residency training and life as a physician, this is a critical period in which to develop and utilize functional and effective coping strategies |
| Žuljević et al., 2021^68^ | Phase 1: Dec 2019 -Jan 2020  Phase 2: Jun 1-20, 2020 | Croatia | OLBI and CBI-modified  OLBI and CBI-modified | Future research on burnout in medical students could also explore how the COVID-19 pandemic affected female students, as there is a knowledge gap on women in healthcare during COVID-19. Any pandemic-related changes in burnout are probably highly dependent on the context and the study setting, especially since regional differences in burnout already existed before the pandemic | Since the COVID-19 pandemic is expected to continue, well-structured longitudinal studies could provide better insight on rates of burnout in medical students over a longer period as the pandemic fluctuates in intensity. They could also answer the question what quantity of disruptions to teaching can be tolerated by medical students over time and how they perceive them over a longer time period, especially changes to clinical teaching.  To be able to more closely explore the cause of any pandemic-related burnout changes in medical students, future studies should specify in detail the type and amount of changes to the curriculum, for example, the impact on clinical teaching and whether e-learning, if present, was performed in an asynchronous or synchronous format. Studies that will assess how satisfied students are with e-learning would also be relevant, since the effectiveness of different e-learning modal- ities is still unexplored, even though it is one of the major changes the COVID-19 pandemic has brought to medical education. Future studies on burnout could also measure if students experience and anxiety and fear specifically related to COVID-19, especially if they attend clinical classes and have contact with patients, as this could impact their burnout levels. Focus should also be placed on comparing burnout effects between students that have different levels of contact with clinical teaching, as we have assessed in our study. |
| Zuniga et al., 2021^69^ | Apr - May 2020 | Chile | MBI-HSS | Lack of information on the underlying psychosocial mediators and mechanisms. | Findings from this study suggest that a formal multifaceted mindfulness-based self-care program incorporated in the core curriculum can help prevent medical students’ distress and promote their well-being, even amidst the COVID-19 pandemic. |

NR= Not reported; MBI-SS= Maslach Burnout Inventory Student Survey; MBI-HSS= Maslach Burnout Inventory Human Service Survey; aMBI= abbreviated Maslach Burnout Inventory; OLBI= Oldenburg Burnout Inventory; OLBI-S= Oldenburg Burnout Inventory- Student version; CBI= Copenhagen Burnout Inventory ; CBI-modified= Copenhagen Burnout Inventory (modified version); CBI-S= Copenhagen Burnout Inventory-Students; LBQ= Link Burnout Questionnaire; ABS= Academic Burnout Scale; LBS= Learning Burnout Scale; ECE= Emotional Exhaustion Scale; PWS=Physician Worklife Study; BCSQ-12-SS=Burnout clinical subtype questionnaire students survey

*Burnout scale adapted from: ^!^Unknown reference – citation in included study is incorrect.

**^*^based on the Student School Burnout Scale (SSBS) by Aypay (2012), Academic Burnout Questionnaire designed by Breso et al. (2007), CBI (Fiorilli et al., 2015), the Italian version of School Burnout Inventory (Platania et al., 2020), OLBI-S (Campo et al., 2012), ABS developed by Rong et al. (2005) and the School Burnout Inventory developed by Salmera-Aro and Naatanen (2005) (unspecified language)

***Burnout scale: Hubungan antara stres dengan burnout pada mahasiswa fakultas psikologi universitas islam negeri (uin) maulana malik ibrahim malang. Skripsi. Universitas Islam Negeri Maulana Malik Ibrahim

**eText 1: List of excluded studies at full-text screening stage**

(wrong population / wrong outcome / wrong study design / unvalidated questionnaire)

1. Hamza CA, Ewing L, Heath NL, Goldstein AL. When social isolation is nothing new: A longitudinal study psychological distress during COVID-19 among university students with and without preexisting mental health concerns Correction to Hamza et al (2020). Canadian Psychology/Psychologie canadienne. 2021;62(1):31-.

2. Aamir S, Winkel C. The impact of Covid-19 on Saudi Arabian female students: an application of the CES-D depression scale. Journal of Educational and Social Research. 2021;11(1):23-31.

3. Abas IMY, Alejail IIEM, Ali SM. Anxiety among the Sudanese university students during the initial stage of COVID-19 pandemic. Heliyon. 2021;7(3):e06300.

4. Abbas M, Dhane M, Beniey M, Meloche-Dumas L, Eissa M, Guerard-Poirier N, et al. Repercussions of the COVID-19 pandemic on the well-being and training of medical clerks: a pan-Canadian survey. BMC Med Educ. 2020;20(1):385.

5. Abdel Jalil MH, Alsous MM, Hammad EA, Mousa R, Saleh MM, Abu Hammour K. Perceived Public Stress Among Jordanians During the COVID-19 Outbreak. Disaster Med Public. 2022;16(1):240-4.

6. Abdulghani HM, Sattar K, Ahmad T, Akram A. Association of COVID-19 Pandemic with undergraduate Medical Students' Perceived Stress and Coping. Psychol Res Behav Manag. 2020;13:871-81.

7. Abdullah SB, Abdullah A, Haque M. Initial 10 Days into COVID-19 Movement Control Order of Malaysia: How Well Did We Fare? Advances in Human Biology. 2021;11(1):106-12.

8. Abdulrazzaq MM, Adnan MM, Abdulhadi Al-Ani ZT. Psychological stress among dental students at al-iraqia university after corona virus pandemic. Indian Journal of Forensic Medicine and Toxicology. 2020;14(3):2397-401.

9. Abenavoli L, Cinaglia P, Lombardo G, Boffoli E, Scida M, Procopio AC, et al. Anxiety and Gastrointestinal Symptoms Related to COVID-19 during Italian Lockdown. J Clin Med. 2021;10(6):1-12.

10. Abokalawa F, Ahmad SF, Al-Hashel J, Hassan AM, Arabi M. The effects of coronavirus disease 2019 (COVID-19) pandemic on people with epilepsy (PwE): an online survey-based study. Acta Neurol Belg. 2022;122(1):59-66.

11. Abuhmaidan Y, Al-Majali S. The Impact of the Coronavirus Pandemic on Mental Health among Al Ain University Students in Light of Some Demographic Variables. Psychiatria Danubina. 2020;32(3-4):482-90.

12. Aebischer O, Weilenmann S, Gachoud D, Mean M, Spiller TR. Physical and psychological health of medical students involved in the coronavirus disease 2019 response in Switzerland. Swiss Med Wkly. 2020;150:w20418.

13. Agarwal N, Harikar M, Shukla R, Bajpai A. COVID-19 pandemic: a double trouble for Indian adolescents and young adults living with type 1 diabetes. Int J Diabetes Dev Ctries. 2020;40(3):346-52.

14. Agbaria Q, Mokh AA. Coping with Stress During the Coronavirus Outbreak: the Contribution of Big Five Personality Traits and Social Support. Int J Ment Health Addict. 2022;20(3):1854-72.

15. Agberotimi SF, Akinsola OS, Oguntayo R, Olaseni AO. Interactions Between Socioeconomic Status and Mental Health Outcomes in the Nigerian Context Amid COVID-19 Pandemic: A Comparative Study. Frontiers in Psychology. 2020;11.

16. Agius AM, Gatt G, Vento Zahra E, Busuttil A, Gainza-Cirauqui ML, Cortes ARG, et al. Self-reported dental student stressors and experiences during the COVID-19 pandemic. J Dent Educ. 2021;85(2):208-15.

17. Agoramoorthy G. India's outburst of online classes during COVID-19 impacts the mental health of students. Curr Psychol. 2022;41(10):7429-30.

18. Ahammed B, Jahan N, Seddeque A, Hossain MT, Shovo TEA, Khan B, et al. Exploring the association between mental health and subjective sleep quality during the COVID-19 pandemic among Bangladeshi university students. Heliyon. 2021;7(5):e07082-e.

19. Ahmad AR, Murad HR. The Impact of Social Media on Panic During the COVID-19 Pandemic in Iraqi Kurdistan: Online Questionnaire Study. J Med Internet Res. 2020;22(5):e19556.

20. Ahmed GK, Ramadan HKA, Refay SM, Khashbah MA. Comparison of knowledge, attitude, socioeconomic burden, and mental health disorders of COVID-19 pandemic between general population and health care workers in Egypt. Egypt J Neurol Psych. 2021;57(1):25.

21. Ahmed O, Ahmed MZ, Alim S, Khan M, Jobe MC. COVID-19 outbreak in Bangladesh and associated psychological problems: An online survey. Death Stud. 2022;46(5):1080-9.

22. Ahmmed SU, Maria AN. Graduating into a Pandemic: Exploring the Factors Influencing the Anxiety Level of the University Finalists. Shanlax International Journal of Education. 2020;9(1):68-75.

23. Ahuja P, Syal G, Kaur A. Psychological stress: Repercussions of COVID-19 on gender. J Public Aff. 2021;21(4).

24. Akdeniz G, Kavakci M, Gozugok M, Yalcinkaya S, Kucukay A, Sahutogullari B. A Survey of Attitudes, Anxiety Status, and Protective Behaviors of the University Students During the COVID-19 Outbreak in Turkey. Front Psychiatry. 2020;11:695.

25. Akinkugbe AA, Garcia DT, Smith CS, Brickhouse TH, Mosavel M. A descriptive pilot study of the immediate impacts of COVID-19 on dental and dental hygiene students' readiness and wellness. J Dent Educ. 2021;85(3):401-10.

26. Akpinar E. The Effect of Online Learning on Tertiary Level Students&#039; Mental Health during the Covid19 Lockdown. The European Journal of Social &amp; Behavioural Sciences. 2021;30(1):3300-10.

27. Al Dhaheri AS, Bataineh MF, Mohamad MN, Ajab A, Al Marzouqi A, Jarrar AH, et al. Impact of COVID-19 on mental health and quality of life: Is there any effect? A cross-sectional study of the MENA region. Plos One. 2021;16(3):e0249107.

28. Al Eid NA, Arnout BA, Alqahtani MMJ, Fadhel FH, Abdelmotelab AS. The mediating role of religiosity and hope for the effect of self-stigma on psychological well-being among COVID-19 patients. Work. 2021;68(3):525-41.

29. Al-Dwaikat TN, Aldalaykeh M, Ta'an W, Rababa M. The relationship between social networking sites usage and psychological distress among undergraduate students during COVID-19 lockdown. Heliyon. 2020;6(12):e05695.

30. Al-Kumaim NH, Alhazmi AK, Mohammed F, Gazem NA, Shabbir MS, Fazea Y. Exploring the Impact of the COVID-19 Pandemic on University Students' Learning Life: An Integrated Conceptual Motivational Model for Sustainable and Healthy Online Learning. Sustainability. 2021;13(5):1-21.

31. Al-Kumaim NH, Mohammed F, Gazem NA, Fazea Y, Alhazmi AK, Dakkak O. Exploring the Impact of Transformation to Fully Online Learning During COVID-19 on Malaysian University Students’ Academic Life and Performance. International Journal of Interactive Mobile Technologies. 2021;15(5):140-58.

32. Al-Ma'seb HB, Al-Sejari MM. Sociodemographic Variables and Illness Attitudes Toward COVID-19 in Kuwait. Can J Behav Sci. 2021;53(3):365-70.

33. AL-Omiri MK, Alzoubi IA, Al Nazeh AA, Alomiri AK, Maswady MN, Lynch E. COVID-19 and Personality: A Cross-Sectional Multicenter Study of the Relationship Between Personality Factors and COVID-19-Related Impacts, Concerns, and Behaviors. Frontiers in Psychiatry. 2021;12.

34. Al-Qahtani AM, Ibrahim HA, Elgzar WT, Elfeki NK, Shaikh MAK, Shaikh IA, et al. Self-efficacy for taking Preventive Measures against COVID-19 among Undergraduate University Students in Saudi Arabia. J Pure Appl Microbio. 2021;15(1):130-7.

35. Al-Sabbah S, Darwish A, Fares N, Barnes J, Almomani JA. Biopsychosocial factors linked with overall well-being of students and educators during the COVID-19 pandemic. Cogent Psychol. 2021;8(1).

36. Al-Shannaq Y, Mohammad AA, Aldalaykeh M. Depression, coping skills, and quality of life among Jordanian adults during the initial outbreak of COVID-19 pandemic: cross sectional study. Heliyon. 2021;7(4):e06873-e.

37. Al-Sofiani ME, Albunyan S, Alguwaihes AM, Kalyani RR, Golden SH, Alfadda A. Determinants of mental health outcomes among people with and without diabetes during the COVID-19 outbreak in the Arab Gulf Region. J Diabetes. 2021;13(4):339-52.

38. Al-Tammemi AB, Akour A, Alfalah L. Is It Just About Physical Health? An Online Cross-Sectional Study Exploring the Psychological Distress Among University Students in Jordan in the Midst of COVID-19 Pandemic. Front Psychol. 2020;11:562213.

39. Alaloul F, Alomari K, Al Qadire M, Al-Dwaikat T. Public knowledge, attitude, practices, and level of anxiety toward the COVID-19 pandemic among people living in Oman. Nurs Forum. 2021;56(3):596-603.

40. Alam MK, Bin Ali F, Banik R, Yasmin S, Salma N. Assessing the mental health condition of home-confined university level students of Bangladesh due to the COVID-19 pandemic. J Public Health-Heid. 2022;30(7):1685-92.

41. Alan S, Surucu SG, Vurgec BA, Cevik A. An investigation of individuals' health anxiety during the COVID-19 pandemic within the framework of the functional health patterns. Perspectives in Psychiatric Care. 2021;57(3):1103-13.

42. AlAteeq DA, Aljhani S, AlEesa D. Perceived stress among students in virtual classrooms during the COVID-19 outbreak in KSA. J Taibah Univ Med Sc. 2020;15(5):398-403.

43. Alemany-Arrebola I, Rojas-Ruiz G, Granda-Vera J, Mingorance-Estrada AC. Influence of COVID-19 on the Perception of Academic Self-Efficacy, State Anxiety, and Trait Anxiety in College Students. Front Psychol. 2020;11:570017.

44. Alex Siu Fung K, Rebecca MP, Mark JA, Kate N, Kate T, Daniel S, et al. Mental health during the COVID-19 pandemic in two longitudinal UK population cohorts. 2020.

45. Alfawaz H, Yakout SM, Wani K, Aljumah GA, Ansari MGA, Khattak MNK, et al. Dietary Intake and Mental Health among Saudi Adults during COVID-19 Lockdown. International Journal of Environmental Research and Public Health. 2021;18(4).

46. Alfawaz HA, Wani K, Aljumah AA, Aldisi D, Ansari MGA, Yakout SM, et al. Psychological well-being during COVID-19 lockdown: Insights from a Saudi State University's Academic Community. J King Saud Univ Sci. 2021;33(1):101262.

47. Alghamdi AA. Impact of the COVID-19 pandemic on the social and educational aspects of Saudi university students' lives. Plos One. 2021;16(4):e0250026.

48. Ali A, Siddiqui AA, Arshad MS, Iqbal F, Arif TB. Effects of COVID-19 pandemic and lockdown on lifestyle and mental health of students: A retrospective study from Karachi, Pakistan. Ann Med Psychol (Paris). 2022;180(6):S29-S37.

49. Ali H, Yilmaz G, Fareed Z, Shahzad F, Ahmad M. Impact of novel coronavirus (COVID-19) on daily routines and air environment: evidence from Turkey. Air Qual Atmos Health. 2021;14(3):381-7.

50. Ali K, Mufti U, Sharma G, Mufti A. A cross-sectional study to assess the quality of life, depression, anxiety and stress levels after 45 days covid-19 lockdown. International Journal of Current Research and Review. 2020;12(22):108-14.

51. Ali M, Ahsan GU, Khan R, Khan HR, Hossain A. Immediate impact of stay-at-home orders to control COVID-19 transmission on mental well-being in Bangladeshi adults: Patterns, Explanations, and future directions. BMC Res Notes. 2020;13(1):494.

52. Ali SJ, Jayaraj G. Psychosocial impact of lockdown among students. European Journal of Molecular and Clinical Medicine. 2020;7(1):686-96.

53. Alkhamees AA, Aljohani MS, Alghesen MA, Alhabib AT. Psychological Distress in Quarantine Designated Facility During COVID-19 Pandemic in Saudi Arabia. Risk Manag Healthc Policy. 2020;13:3103-20.

54. Alkhamees AA, Alrashed SA, Alzunaydi AA, Almohimeed AS, Aljohani MS. The psychological impact of COVID-19 pandemic on the general population of Saudi Arabia. Compr Psychiatry. 2020;102:152192.

55. Alkwai HM. Graduating from Medical School amid a Pandemic: A Study of Graduates' Mental Health and Concerns. Educ Res Int. 2021;2021.

56. Allen SF, Stevenson J, Lazuras L, Akram U. The role of the COVID-19 pandemic in altered psychological well-being, mental health and sleep: an online cross-sectional study. Psychol Health Med. 2022;27(2):343-51.

57. Allison S, Rebekah LL, Debra R, Jeanette Gowen C. Uncovering the compounding effects of COVID-19 and racism on mental health disparities among biomedical PhD and MD students. 2021.

58. Almazan AN, Chun AS, Perez-Urbano I. The Medical Student Response to the Mental Health Consequences of COVID-19. Acad Psychiatry. 2020;44(6):689-90.

59. Almomani EY, Almomany AM. The Impact of COVID-19 Curfew Restrictions on the University Students' Academic Learning and Mental Health: A Study from Jordan. J Loss Trauma. 2021;26(5):501-3.

60. Almomani EY, Qablan AM, Almomany AM, Atrooz FY. The coping strategies followed by university students to mitigate the COVID-19 quarantine psychological impact. Curr Psychol. 2021;40(11):5772-81.

61. Alonzi S, La Torre A, Silverstein MW. The psychological impact of preexisting mental and physical health conditions during the COVID-19 pandemic. Psychol Trauma. 2020;12(S1):S236-S8.

62. Alqahtani AS, Alrasheed MM, Alqunaibet AM. Public Response, Anxiety and Behaviour during the First Wave of COVID-19 Pandemic in Saudi Arabia. Int J Environ Res Public Health. 2021;18(9).

63. Alqudah A, Al-Smadi A, Oqal M, Qnais EY, Wedyan M, Abu Gneam M, et al. About anxiety levels and anti-anxiety drugs among quarantined undergraduate Jordanian students during COVID-19 pandemic. Int J Clin Pract. 2021;75(7):e14249.

64. Alrasheedy AA, Abdulsalim S, Farooqui M, Alsahali S, Godman B. Knowledge, Attitude and Practice About Coronavirus Disease (COVID-19) Pandemic and Its Psychological Impact on Students and Their Studies: A Cross-Sectional Study Among Pharmacy Students in Saudi Arabia. Risk Manag Healthc P. 2021;14:729-41.

65. Alsairafi Z, Naser AY, Alsaleh FM, Awad A, Jalal Z. Mental Health Status of Healthcare Professionals and Students of Health Sciences Faculties in Kuwait during the COVID-19 Pandemic. Int J Environ Res Public Health. 2021;18(4).

66. Alsalman A, Jahrami H, Mubarak H, Aljabal M, Abdulnabi M, Yusuf A, et al. The Psychological Impact of COVID-19 Pandemic on the population of Bahrain. Acta Biomed. 2020;91(4):e2020131.

67. Alshehri FS, Alatawi Y, Alghamdi BS, Alhifany AA, Alharbi A. Prevalence of post-traumatic stress disorder during the COVID-19 pandemic in Saudi Arabia. Saudi Pharm J. 2020;28(12):1666-73.

68. Alsolais A, Alquwez N, Alotaibi KA, Alqarni AS, Almalki M, Alsolami F, et al. Risk perceptions, fear, depression, anxiety, stress and coping among Saudi nursing students during the COVID-19 pandemic. J Ment Health. 2021;30(2):194-201.

69. Alyami HS, Naser AY, Dahmash EZ, Alyami MH, Alyami MS. Depression and anxiety during the COVID-19 pandemic in Saudi Arabia: A cross-sectional study. Int J Clin Pract. 2021;75(7):e14244.

70. Alyami M, de Albuquerque JV, Krageloh CU, Alyami H, Henning MA. Effects of Fear of COVID-19 on Mental Well-Being and Quality of Life among Saudi Adults: A Path Analysis. Saudi J Med Med Sci. 2021;9(1):24-30.

71. Amar Prashad C, Narayan Sah S, Moumita B, Jamuna TR, Shailesh Y. Impact on Mental Health of students due to restriction caused by COVID-19 pandemic: Cross-sectional study. 2021.

72. Amaral-Prado HM, Borghi F, Mello TMVF, Grassi-Kassisse DM. The impact of confinement in the psychosocial behaviour due COVID-19 among members of a Brazilian university. Int J Soc Psychiatr. 2021;67(6):720-7.

73. Amatori S, Donati Zeppa S, Preti A, Gervasi M, Gobbi E, Ferrini F, et al. Dietary Habits and Psychological States during COVID-19 Home Isolation in Italian College Students: The Role of Physical Exercise. Nutrients. 2020;12(12).

74. Amendola S, von Wyl A, Volken T, Zysset A, Huber M, Dratva J. A Longitudinal Study on Generalized Anxiety Among University Students During the First Wave of the COVID-19 Pandemic in Switzerland. Frontiers in Psychology. 2021;12:643171.

75. Amerio A, Brambilla A, Morganti A, Aguglia A, Bianchi D, Santi F, et al. COVID-19 Lockdown: Housing Built Environment's Effects on Mental Health. Int J Environ Res Public Health. 2020;17(16).

76. Ammar A, Mueller P, Trabelsi K, Chtourou H, Boukhris O, Masmoudi L, et al. Psychological consequences of COVID-19 home confinement: The ECLB-COVID19 multicenter study. Plos One. 2020;15(11):e0240204.

77. Amor AM, Navas P, Verdugo MA, Crespo M. Perceptions of people with intellectual and developmental disabilities about COVID-19 in Spain: a cross-sectional study. J Intell Disabil Res. 2021;65(5):381-96.

78. Andrade EF, Pereira LJ, de Oliveira APL, Orlando DR, Alves DAG, Guilarducci JD, et al. Perceived fear of COVID-19 infection according to sex, age and occupational risk using the Brazilian version of the Fear of COVID-19 Scale. Death Studies. 2022;46(3):533-42.

79. Andrades-Tobar M, García FE, Concha-Ponce P, Valiente C, Lucero C. Predictors of anxiety, depression, and stress symptoms from the COVID-19 outbreak. Revista de Psicopatologia y Psicologia Clinica. 2021;26(1):13-22.

80. Anindyajati G, Wiguna T, Murtani BJ, Christian H, Wigantara NA, Putra AA, et al. Anxiety and Its Associated Factors During the Initial Phase of the COVID-19 Pandemic in Indonesia. Front Psychiatry. 2021;12:634585.

81. Anis Z, Boyu Z, Ehsan H, Vincent S, Henry K. The Relationship between Deteriorating Mental Health Conditions and Longitudinal Behavioral Changes in Google and YouTube Usages among College Students in the United States during COVID-19: Observational Study. 2020.

82. Anonymous. The Impact of COVID-19 on the Well-Being of Division III Student-Athletes. Sport Journal. 2020:N.PAG-N.PAG.

83. Antiporta DA, Cutipe YL, Mendoza M, Celentano DD, Stuart EA, Bruni A. Depressive symptoms among Peruvian adult residents amidst a National Lockdown during the COVID-19 pandemic. Bmc Psychiatry. 2021;21(1).

84. Anyan F, Hjemdal O, Ernstsen L, Havnen A. Change in Physical Activity During the Coronavirus Disease 2019 Lockdown in Norway: The Buffering Effect of Resilience on Mental Health. Front Psychol. 2020;11:598481.

85. Apgar D, Cadmus T. Using Mixed Methods to Assess the Coping and Self-regulation Skills of Undergraduate Social Work Students Impacted by COVID-19. Clin Soc Work J. 2022;50(1):55-66.

86. Ara T, Rahman MM, Hossain MA, Ahmed A. Identifying the Associated Risk Factors of Sleep Disturbance During the COVID-19 Lockdown in Bangladesh: A Web-Based Survey. Front Psychiatry. 2020;11:580268.

87. Arad G, Shamai-Leshem D, Bar-Haim Y. Social Distancing During A COVID-19 Lockdown Contributes to The Maintenance of Social Anxiety: A Natural Experiment. Cognit Ther Res. 2021;45(4):708-14.

88. Araque-Castellanos F, Gonzalez-Gutierrez O, Lopez-Jaimes RJ, Medina-Ortiz O, Nuvan-Hurtado I-L. Psychological well-being and sociodemographic characteristics in university students during quarantine by SARS-COV-2. Archivos Venezolanos de Farmacologia y Terapeutica. 2020;39(8):998-1004.

89. Araujo FJO, de Lima LSA, Cidade PIM, Nobre CB, Neto MLR. Impact Of Sars-Cov-2 And Its Reverberation In Global Higher Education And Mental Health. Psychiatry Res. 2020;288:112977.

90. Arenas DL, Viduani AC, Bassols AMS, Hauck S. Peer support intervention as a tool to address college students' mental health amidst the COVID-19 pandemic. Int J Soc Psychiatr. 2021;67(3):301-2.

91. Arima M, Takamiya Y, Furuta A, Siriratsivawong K, Tsuchiya S, Izumi M. Factors associated with the mental health status of medical students during the COVID-19 pandemic: a cross-sectional study in Japan. Bmj Open. 2020;10(12):e043728.

92. Arora S, Chaudhary P, Singh RK. Impact of coronavirus and online exam anxiety on self-efficacy: the moderating role of coping strategy. Interact Technol Sma. 2021;18(3):475-92.

93. Arruda ESB, Nadielle Silva D, Rodrigo Santos R, Rafael Luiz A. Avaliação dos graus de ansiedade em acadêmicos de uma faculdade privada. Sci med (Porto Alegre, Online). 2020;30(1):35209-.

94. Arslan G. Understanding wellbeing and death obsession of young adults in the context of Coronavirus experiences: Mitigating the effect of mindful awareness. Death Stud. 2022;46(8):1923-32.

95. Arslan G, Allen KA. Exploring the association between coronavirus stress, meaning in life, psychological flexibility, and subjective well-being. Psychol Health Med. 2022;27(4):803-14.

96. Arslan G, Yildirim M. Coronavirus stress, meaningful living, optimism, and depressive symptoms: a study of moderated mediation model. Aust J Psychol. 2021;73(2):113-24.

97. Arslan G, Yildirim M, Aytac M. Subjective vitality and loneliness explain how coronavirus anxiety increases rumination among college students. Death Studies. 2022;46(5):1042-51.

98. Arslan G, Yildirim M, Karatas Z, Kabasakal Z, Kilinc M. Meaningful Living to Promote Complete Mental Health Among University Students in the Context of the COVID-19 Pandemic. Int J Ment Health Ad. 2022;20(2):930-42.

99. Arslan G, Yildirim M, Zangeneh M. Coronavirus Anxiety and Psychological Adjustment in College Students: Exploring the Role of College Belongingness and Social Media Addiction. Int J Ment Health Addict. 2022;20(3):1546-59.

100. Ashraf F, Zareen G, Nusrat A, Arif A, Griffiths MD. Correlates of Psychological Distress Among Pakistani Adults During the COVID-19 Outbreak: Parallel and Serial Mediation Analyses. Frontiers in Psychology. 2021;12:647821.

101. Asiamah N, Opuni FF, Mends-Brew E, Mensah SW, Mensah HK, Quansah F. Short-Term Changes in Behaviors Resulting from COVID-19-Related Social Isolation and Their Influences on Mental Health in Ghana. Community Ment Hlt J. 2021;57(1):79-92.

102. Aslan H, Pekince H. Nursing students' views on the COVID-19 pandemic and their percieved stress levels. Perspectives in Psychiatric Care. 2021;57(2):695-701.

103. Aslan I, Ochnik D, Cinar O. Exploring Perceived Stress among Students in Turkey during the COVID-19 Pandemic. International Journal of Environmental Research and Public Health. 2020;17(23).

104. Ausín B, Castellanos MA, González-Sanguino C, Vakhantseva OV, Almazova OV, Shaigerova LA, et al. The Psychological Impact of Six Weeks of Lockdown as a Consequence of COVID-19 and the Importance of Social Support: A Cross-Cultural Study Comparing Spanish and Russian Populations. Psychology in Russia: State of the Art. 2020;13(4):89-105.

105. Awoke M, Mamo G, Abdu S, Terefe B. Perceived Stress and Coping Strategies Among Undergraduate Health Science Students of Jimma University Amid the COVID-19 Outbreak: Online Cross-Sectional Survey. Frontiers in Psychology. 2021;12:639955.

106. Aylie NS, Mekonen MA, Mekuria RM. The Psychological Impacts of COVID-19 Pandemic Among University Students in Bench-Sheko Zone, South-west Ethiopia: A Community-based Cross-sectional Study. Psychology Research and Behavior Management. 2020;13:813-21.

107. Ayran G, Kose S, Kucukoglu S, Ozdemir AA. The effect of anxiety on nicotine dependence among university students during the COVID-19 pandemic. Perspectives in Psychiatric Care. 2022;58(1):114-23.

108. Bahadir-Yilmaz E, Yuksel A. Evaluation of the psychological problems experienced by university students during the COVID-19 outbreak and suggestions. Perspect Psychiatr Care. 2021;57(2):968-9.

109. Bahcecioglu Turan G, Kose S, Aksoy M. Analysis of nursing students' obsessive and coping behaviors during the COVID-19 pandemic. Perspect Psychiatr Care. 2021;57(4):1628-36.

110. Bahcecioglu Turan G, Ozer Z, Ciftci B. Analysis of anxiety levels and attitudes of nursing students toward the nursing profession during the COVID-19 pandemic. Perspect Psychiatr Care. 2021;57(4):1913-21.

111. Bai W, Xi HT, Zhu Q, Wang Z, Han L, Chen P, et al. Changes in Nursing Students' Career Choices Following the COVID-19 Pandemic in China. Front Psychiatry. 2021;12:657021.

112. Baiano C, Zappullo I, The Lab NG, Conson M. Tendency to Worry and Fear of Mental Health during Italy's COVID-19 Lockdown. Int J Environ Res Public Health. 2020;17(16).

113. Balhara YPS, Kattula D, Singh S, Chukkali S, Bhargava R. Impact of lockdown following COVID-19 on the gaming behavior of college students. Indian J Public Health. 2020;64(Supplement):S172-S6.

114. Bali EB, Tanalp TD, Celebi I. Awareness levels of health technician candidates about the knowledge and prevention ways of the new type coronavirus (COVID-19) pandemic. Flora. 2021;26(1):67-77.

115. Baloch GM, Kamaludin K, Chinna K, Sundarasen S, Nurunnabi M, Khoshaim HB, et al. Coping with COVID-19: The Strategies Adapted by Pakistani Students to Overcome Implications. Int J Environ Res Public Health. 2021;18(4).

116. Baloran ET. Knowledge, Attitudes, Anxiety, and Coping Strategies of Students during COVID-19 Pandemic. J Loss Trauma. 2020;25(8):635-42.

117. Balsamo M, Carlucci L. Italians on the Age of COVID-19: The Self-Reported Depressive Symptoms Through Web-Based Survey. Frontiers in Psychology. 2020;11:569276.

118. Banna MHA, Sayeed A, Kundu S, Christopher E, Hasan MT, Begum MR, et al. The impact of the COVID-19 pandemic on the mental health of the adult population in Bangladesh: a nationwide cross-sectional study. Int J Environ Health Res. 2022;32(4):850-61.

119. Bantjes J, Kazdin AE, Cuijpers P, Breet E, Dunn-Coetzee M, Davids C, et al. A Web-Based Group Cognitive Behavioral Therapy Intervention for Symptoms of Anxiety and Depression Among University Students: Open-Label, Pragmatic Trial. Jmir Ment Health. 2021;8(5):e27400.

120. Bar-Zeev Y, Shauly M, Lee H, Neumark Y. Changes in Smoking Behaviour and Home-Smoking Rules during the Initial COVID-19 Lockdown Period in Israel. Int J Environ Res Public Health. 2021;18(4).

121. Barrea L, Pugliese G, Framondi L, Di Matteo R, Laudisio D, Savastano S, et al. Does Sars-Cov-2 threaten our dreams? Effect of quarantine on sleep quality and body mass index. Journal of Translational Medicine. 2020;18(1).

122. Barrett AM, Hogreve J, Bruggen EC. Coping With Governmental Restrictions: The Relationship Between Stay-at-Home Orders, Resilience, and Functional, Social, Mental, Physical, and Financial Well-Being. Frontiers in Psychology. 2021;11.

123. Barron Millar E, Singhal D, Vijayaraghavan P, Seshadri S, Smith E, Dixon P, et al. Health anxiety, coping mechanisms and COVID 19: An Indian community sample at week 1 of lockdown. Plos One. 2021;16(4):e0250336.

124. Bartos LJ, Funes MJ, Ouellet M, Posadas MP, Krageloh C. Developing Resilience During the COVID-19 Pandemic: Yoga and Mindfulness for the Well-Being of Student Musicians in Spain. Front Psychol. 2021;12:642992.

125. Bartoszek A, Walkowiak D, Bartoszek A, Kardas G. Mental Well-Being (Depression, Loneliness, Insomnia, Daily Life Fatigue) during COVID-19 Related Home-Confinement-A Study from Poland. Int J Environ Res Public Health. 2020;17(20):7417-.

126. Basheti IA, Mhaidat QN, Mhaidat HN. Prevalence of anxiety and depression during COVID-19 pandemic among healthcare students in Jordan and its effect on their learning process: A national survey. Plos One. 2021;16(4):e0249716.

127. Bashir TF, Hassan S, Maqsood A, Khan ZA, Issrani R, Ahmed N, et al. The Psychological Impact Analysis of Novel COVID-19 Pandemic in Health Sciences Students: A Global Survey. Eur J Dent. 2020;14(S 01):S91-S6.

128. Batais MA, Temsah MH, AlGhofili H, AlRuwayshid N, Alsohime F, Almigbal TH, et al. The coronavirus disease of 2019 pandemic-associated stress among medical students in middle east respiratory syndrome-CoV endemic area An observational study. Medicine. 2021;100(3):e23690.

129. Batool-Anwar S, Robbins R, Ali SH, Capasso A, Foreman J, Jones AM, et al. Examining changes in sleep duration associated with the onset of the COVID-19 pandemic: Who is sleeping and who is not? medRxiv.

130. Baumel K, Hamlett M, Wheeler B, Hall D, Randall AK, Mickelson K. Living Through COVID-19: Social Distancing, Computer-Mediated Communication, and Well-Being in Sexual Minority and Heterosexual Adults. J Homosexual. 2021;68(4):673-91.

131. Bawa R, Gabram O, Klatt MD, Blake A, Steinberg B, Westrick A, et al. Embracing Change: A Mindful Medical Center Meets COVID-19. Global Advances In Health and Medicine. 2020;9.

132. Behzadnia B, FatahModares S. Basic Psychological Need-Satisfying Activities during the COVID-19 Outbreak. Appl Psychol Health Well Being. 2020;12(4):1115-39.

133. Belen H. Fear of COVID-19 and Mental Health: The Role of Mindfulness in During Times of Crisis. Int J Ment Health Addict. 2022;20(1):607-18.

134. Ben-Zeev D, Buck B, Meller S, Hudenko WJ, Hallgren KA. Augmenting Evidence-Based Care With a Texting Mobile Interventionist: A Pilot Randomized Controlled Trial. Psychiatr Serv. 2020;71(12):1218-24.

135. Benham G. Stress and sleep in college students prior to and during the COVID-19 pandemic. Stress Health. 2021;37(3):504-15.

136. Berg MB, Lin L. Prevalence and predictors of early COVID-19 behavioral intentions in the United States. Transl Behav Med. 2020;10(4):843-9.

137. Berg-Beckhoff G, Dalgaard Guldager J, Tanggaard Andersen P, Stock C, Smith Jervelund S. What Predicts Adherence to Governmental COVID-19 Measures among Danish Students? Int J Environ Res Public Health.18(4).

138. Besser A, Flett GL, Nepon T, Zeigler-Hill V. Personality, Cognition, and Adaptability to the COVID-19 Pandemic: Associations with Loneliness, Distress, and Positive and Negative Mood States. Int J Ment Health Addict. 2022;20(2):971-95.

139. Bezerra ACV, Silva C, Soares FRG, Silva J. Factors associated with people's behavior in social isolation during the COVID-19 pandemic. Cien Saude Colet. 2020;25(suppl 1):2411-21.

140. Bharti CSCHSRJIA. Is coronavirus lockdown taking a toll on mental health of medical students? A study using WHOQOL-BREF questionnaire. Journal of Family Medicine and Primary Care. 2020;9(10):5261-6.

141. Bhuvaneshwari G, Babu B, Balasuntharam B. A study to assess the level of knowledge and level of anxiety on coronavirus disease 2019 among adults living in Adukkamparai. Drug Invention Today. 2020;13(7):979-82.

142. Biber DD, Melton B, Czech DR. The impact of COVID-19 on college anxiety, optimism, gratitude, and course satisfaction. J Am Coll Health. 2022;70(7):1947-52.

143. Bilges Y, Bilge Y. Investigation of the effects of coronavirus and social isolation on psychological symptoms in terms of psychological resilience and coping styles. Klin Psikiyatr Derg. 2020;23:38-51.

144. Bilgi K, Aytas G, Karatoprak U, Kazancioglu R, Ozcelik S. The Effects of Coronavirus Disease 2019 Outbreak on Medical Students. Front Psychiatry. 2021;12:637946.

145. Birhanu A, Tiki T, Mekuria M, Yilma D, Melese G, Seifu B. COVID-19-Induced Anxiety and Associated Factors Among Urban Residents in West Shewa Zone, Central Ethiopia, 2020. Psychology Research and Behavior Management. 2021;14:99-108.

146. Biswas S, Biswas A. Anxiety level among students of different college and universities in India during lock down in connection to the COVID-19 pandemic. J Public Health-Heid. 2021:1-7.

147. Blake H, Corner J, Cirelli C, Hassard J, Briggs L, Daly JM, et al. Perceptions and Experiences of the University of Nottingham Pilot SARS-CoV-2 Asymptomatic Testing Service: A Mixed-Methods Study. Int J Environ Res Public Health. 2020;18(1).

148. Blake H, Knight H, Jia R, Corner J, Morling JR, Denning C, et al. Students' Views towards Sars-Cov-2 Mass Asymptomatic Testing, Social Distancing and Self-Isolation in a University Setting during the COVID-19 Pandemic: A Qualitative Study. Int J Environ Res Public Health. 2021;18(8).

149. Blasco-Belled A, Tejada-Gallardo C, Torrelles-Nadal C, Alsinet C. The Costs of the COVID-19 on Subjective Well-Being: An Analysis of the Outbreak in Spain. Sustainability. 2020;12(15).

150. Blom V, Lonn A, Ekblom B, Kallings LV, Vaisanen D, Hemmingsson E, et al. Lifestyle Habits and Mental Health in Light of the Two COVID-19 Pandemic Waves in Sweden, 2020. Int J Environ Res Public Health. 2021;18(6).

151. Bogolyubova O, Fernandez ASM, Lopez BT, Portelli P. Traumatic impact of the COVID-19 pandemic in an international sample: Contribution of fatalism to psychological distress and behavior change. Eur J Trauma Dissoc. 2021;5(2).

152. Bohn J, Hogue S. Changing the Game: College Dance Training for Well-Being and Resilience Amidst the COVID-19 Crisis. Health Promot Pract. 2021;22(2):163-6.

153. Bollen Z, Pabst A, Creupelandt C, Fontesse S, Lannoy S, Pinon N, et al. Prior drinking motives predict alcohol consumption during the COVID-19 lockdown: A cross-sectional online survey among Belgian college students. Addict Behav. 2021;115:106772.

154. Bonar EE, Chapman L, McAfee J, Goldstick JE, Bauermeister JA, Carter PM, et al. Perceived impacts of the COVID-19 pandemic on cannabis-using emerging adults. Transl Behav Med. 2021;11(7):1299-309.

155. Bonar EE, Parks MJ, Gunlicks-Stoessel M, Lyden GR, Mehus CJ, Morrell N, et al. Binge drinking before and after a COVID-19 campus closure among first-year college students. Addict Behav. 2021;118:106879.

156. Bonati M, Campi R, Zanetti M, Cartabia M, Scarpellini F, Clavenna A, et al. Psychological distress among Italians during the 2019 coronavirus disease (COVID-19) quarantine. Bmc Psychiatry. 2021;21(1):20.

157. Bonichini S, Tremolada M. Quality of Life and Symptoms of PTSD during the COVID-19 Lockdown in Italy. Int J Environ Res Public Health. 2021;18(8).

158. Borrega-Mouquinho Y, Sanchez-Gomez J, Fuentes-Garcia JP, Collado-Mateo D, Villafaina S. Effects of High-Intensity Interval Training and Moderate-Intensity Training on Stress, Depression, Anxiety, and Resilience in Healthy Adults During Coronavirus Disease 2019 Confinement: A Randomized Controlled Trial. Front Psychol. 2021;12:643069.

159. Bortolon C, Capdevielle D, Dubreucq J, Raffard S. Persecutory ideation and anomalous perceptual experiences in the context of the COVID-19 outbreak in France: what's left one month later? J Psychiatr Res. 2021;134:215-22.

160. Boukrim M, Obtel M, Kasouati J, Achbani A, Razine R. Covid-19 and Confinement: Effect on Weight Load, Physical Activity and Eating Behavior of Higher Education Students in Southern Morocco. Ann Glob Health. 2021;87(1):7.

161. Bourion-Bedes S, Tarquinio C, Batt M, Tarquinio P, Lebreuilly R, Sorsana C, et al. Psychological impact of the COVID-19 outbreak on students in a French region severely affected by the disease: results of the PIMS-CoV 19 study. Psychiatry Res. 2021;295:113559.

162. Bourion-Bédès S, Tarquinio C, Batt M, Tarquinio P, Lebreuilly R, Sorsana C, et al. Stress and associated factors among French university students under the COVID-19 lockdown: The results of the PIMS-CoV 19 study. Journal of Affective Disorders. 2021;283:108-14.

163. Boursier V, Gioia F, Musetti A, Schimmenti A. Facing Loneliness and Anxiety During the COVID-19 Isolation: The Role of Excessive Social Media Use in a Sample of Italian Adults. Frontiers in Psychiatry. 2020;11.

164. Boyraz G, Legros DN, Tigershtrom A. COVID-19 and traumatic stress: The role of perceived vulnerability, COVID-19-related worries, and social isolation. J Anxiety Disord. 2020;76:102307.

165. Bradfield Z, Wynter K, Hauck Y, Vasilevski V, Kuliukas L, Wilson AN, et al. Experiences of receiving and providing maternity care during the COVID-19 pandemic in Australia: A five-cohort cross-sectional comparison. Plos One. 2021;16(3):e0248488.

166. Brailovskaia J, Cosci F, Mansueto G, Miragall M, Herrero R, Banos RM, et al. The association between depression symptoms, psychological burden caused by Covid-19 and physical activity: An investigation in Germany, Italy, Russia, and Spain. Psychiat Res. 2021;295:113596.

167. Brailovskaia J, Margraf J. Predicting adaptive and maladaptive responses to the Coronavirus (COVID-19) outbreak: A prospective longitudinal study. Int J Clin Health Psychol. 2020;20(3):183-91.

168. Brand PLP. COVID-19: a unique learning opportunity if the well-being of learners and frontline workers is adequately supported. Perspectives on Medical Education. 2020;9(3):129-31.

169. Branquinho C, Kelly C, Arevalo LC, Santos A, de Matos MG. "Hey, we also have something to say": A qualitative study of Portuguese adolescents' and young people's experiences under COVID-19. Journal of Community Psychology. 2020;48(8):2740-52.

170. Bressington DT, Cheung TCC, Lam SC, Suen LKP, Fong TKH, Ho HSW, et al. Association Between Depression, Health Beliefs, and Face Mask Use During the COVID-19 Pandemic. Frontiers in Psychiatry. 2020;11.

171. Bridgland VME, Moeck EK, Green DM, Swain TL, Nayda DM, Matson LA, et al. Why the COVID-19 pandemic is a traumatic stressor. Plos One. 2021;16(1):e0240146.

172. Brose A, Blanke ES, Schmiedek F, Kramer AC, Schmidt A, Neubauer AB. Change in mental health symptoms during the COVID-19 pandemic: The role of appraisals and daily life experiences. J Pers. 2021;89(3):468-82.

173. Brouwer KR, Walmsley LA, Parrish EM, McCubbin AK, Welsh JD, Braido CEC, et al. Examining the associations between self-care practices and psychological distress among nursing students during the COVID-19 pandemic. Nurs Educ Today. 2021;100:104864.

174. Brouzos A, Vassilopoulos SP, Baourda VC, Tassi C, Stavrou V, Moschou K, et al. "Staying Home - Feeling Positive": Effectiveness of an on-line positive psychology group intervention during the COVID-19 pandemic. Curr Psychol. 2021:1-13.

175. Browning MHEM, Larson LR, Sharaievska I, Rigolon A, McAnirlin O, Mullenbach L, et al. Psychological impacts from COVID-19 among university students: Risk factors across seven states in the United States. Plos One. 2021;16(1).

176. Bruno G, Panzeri A, Granziol U, Alivernini F, Chirico A, Galli F, et al. The Italian COVID-19 Psychological Research Consortium (IT C19PRC): General Overview and Replication of the UK Study. J Clin Med. 2020;10(1).

177. Brusadelli E, Ferrari L, Benetti M, Bruzzese S, Tonelli GM, Gullo S. Online Supportive Group as social intervention to face COVID lockdown. A qualitative study on psychotherapists, psychology trainees and students, and community people. Res Psychother-Psych. 2020;23(3):279-90.

178. Bu F, Mak HW, Fancourt D. Rates and predictors of uptake of mental health support during the COVID-19 pandemic: an analysis of 26,720 adults in the UK in lockdown. Soc Psychiatry Psychiatr Epidemiol. 2021;56(12):2287-97.

179. Bu F, Steptoe A, Fancourt D. Loneliness during a strict lockdown: Trajectories and predictors during the COVID-19 pandemic in 38,217 United Kingdom adults. Soc Sci Med. 2020;265:113521.

180. Bu F, Steptoe A, Fancourt D. Who is lonely in lockdown? Cross-cohort analyses of predictors of loneliness before and during the COVID-19 pandemic. Public Health. 2020;186:31-4.

181. Buckner JD, Abarno CN, Lewis EM, Zvolensky MJ, Garey L. Increases in distress during stay-at-home mandates During the COVID-19 pandemic: A longitudinal study. Psychiatry Res. 2021;298:113821.

182. Bullard J. The impact of COVID-19 on the well-being of division III student-athletes. Sport J. 2020;21:1-25.

183. Bussone S, Pesca C, Tambelli R, Carola V. Psychological Health Issues Subsequent to SARS-Cov 2 Restrictive Measures: The Role of Parental Bonding and Attachment Style. Front Psychiatry. 2020;11:589444.

184. Byrom N. The challenges of lockdown for early-career researchers. Elife. 2020;9.

185. Caballero-Domínguez CC, Jiménez-Villamizar MP, Campo-Arias A. Suicide risk during the lockdown due to coronavirus disease (COVID-19) in Colombia. Death Studies. 2020.

186. Cai X, Hu XP, Ekumi IO, Wang JC, An YW, Li ZW, et al. Psychological Distress and Its Correlates Among COVID-19 Survivors During Early Convalescence Across Age Groups. Am J Geriat Psychiat. 2020;28(10):1030-9.

187. Calderaro DC, Kahlow BS, Munhoz GA, Dias SEB, Lopes JVZ, Borges AR, et al. Effects of Participating in a Research Project During the COVID-19 Pandemic on Medical Students' Educational Routines and Mental Health: Protocol for a Web-Based Survey Study. JMIR Res Protoc. 2021;10(4):e24617.

188. Cam HH, Top FU, Ayyildiz TK. Impact of the COVID-19 pandemic on mental health and health-related quality of life among university students in Turkey. Curr Psychol. 2022;41(2):1033-42.

189. Camacho-Zuñiga C, Pego L, Escamilla J, Hosseini S. The impact of the COVID-19 pandemic on students’ feelings at high school, undergraduate, and postgraduate levels. Heliyon. 2021;7(3):e06465.

190. Camilleri C, Perry JT, Sammut S. Compulsive Internet Pornography Use and Mental Health: A Cross-Sectional Study in a Sample of University Students in the United States. Front Psychol. 2020;11:613244.

191. Campos J, Campos LA, Bueno JL, Martins BG. Emotions and mood swings of pharmacy students in the context of the coronavirus disease of 2019 pandemic. Curr Pharm Teach Learn. 2021;13(6):635-42.

192. Canas-Lerma AJ, Cuartero-Castaner ME, Mascialino G, Hidalgo-Andrade P. Empathy and COVID-19: Study in Professionals and Students of the Social Health Field in Ecuador. Int J Environ Res Public Health. 2021;18(1).

193. Canli D, Karasar B. Health Anxiety and Emotion Regulation during the Period of COVID-19 Outbreak in Turkey. Psychiatr Danub. 2020;32(3-4):513-20.

194. Cansel N, Ucuz I, Arslan AK, Kayhan Tetik B, Colak C, Melez SNI, et al. Prevalence and predictors of psychological response during immediate COVID-19 pandemic. Int J Clin Pract. 2021;75(5):e13996.

195. Cao W, Fang Z, Hou G, Han M, Xu X, Dong J, et al. The psychological impact of the COVID-19 epidemic on college students in China. Psychiatry Res. 2020;287:112934.

196. Capasso A, Jones AM, Ali SH, Foreman J, Tozan Y, DiClemente RJ. Increased alcohol use during the COVID-19 pandemic: The effect of mental health and age in a cross-sectional sample of social media users in the U.S. Preventive Medicine. 2021;145.

197. Capone V, Caso D, Donizzetti AR, Procentese F. University student mentalwell-being during COVID-19 outbreak: What are the relationships between information seeking, perceived risk and personal resources related to the academic context? Sustainability. 2020;17(12).

198. Carlos Izaias Sartorao F, Wilson Conti de Las Villas R, Ricardo Beauchamp de C, Arlete Aparecida M, Shirlene P, Luiz T, et al. IMPACT OF COVID-19 PANDEMIC ON MENTAL HEALTH OF MEDICAL STUDENTS:A CROSS-SECTIONAL STUDY USING GAD-7 AND PHQ-9 QUESTIONNAIRES. 2020.

199. Carlos von Krakauer H, Sr., Marcella Lima B, Rafaella Dourado L. Distress among Brazilian university students due to the Covid-19 pandemic: survey results and reflections. 2020.

200. Carriedo A, Cecchini JA, Fernandez-Rio J, Mendez-Gimenez A. Resilience and physical activity in people under home isolation due to COVID-19: A preliminary evaluation. Ment Health Phys Act. 2020;19.

201. Casafont C, Fabrellas N, Rivera P, Olive-Ferrer MC, Querol E, Venturas M, et al. Experiences of nursing students as healthcare aid during the COVID-19 pandemic in Spain: A phemonenological research study. Nurs Educ Today. 2021;97:104711.

202. Casali N, Feraco T, Ghisi M, Meneghetti C. “Andrà tutto bene”: Associations Between Character Strengths, Psychological Distress and Self-efficacy During Covid-19 Lockdown. Journal of Happiness Studies. 2020.

203. Castellano-Tejedor C, Torres-Serrano M, Cencerrado A. Psychological impact in the time of COVID-19: A cross-sectional population survey study during confinement. J Health Psychol. 2022;27(4):974-89.

204. Cayo-Rojas CF, Castro-Mena MJ, Agramonte-Rosell RC, Aliaga-Marinas AS, Ladera-Castaneda MI, Cervantes-Ganoza LA, et al. Impact of COVID-19 Mandatory Social Isolation on the Development of Anxiety in Peruvian Dentistry Students: A Logistic Regression Analysis. J Int Soc Prev Community Dent. 2021;11(2):222-9.

205. Cayo-Rojas CF, Castro-Mena MJ, Agramonte-Rosell RLC. Strategies to decrease anxiety in dental students due to social isolation. Revista Cubana de Estomatologia. 2021;58(1):1-3.

206. Cellini N, Canale N, Mioni G, Costa S. Changes in sleep pattern, sense of time and digital media use during COVID-19 lockdown in Italy. J Sleep Res. 2020;29(4):e13074.

207. Cenat JM, Dalexis RD, Guerrier M, Noorishad PG, Derivois D, Bukaka J, et al. Frequency and correlates of anxiety symptoms during the COVID-19 pandemic in low- and middle-income countries: A multinational study. J Psychiatr Res. 2021;132:13-7.

208. Cenat JM, Noorishad PG, Kokou-Kpolou CK, Dalexis RD, Hajizadeh S, Guerrier M, et al. Prevalence and correlates of depression during the COVID-19 pandemic and the major role of stigmatization in low- and middle-income countries: A multinational cross-sectional study. Psychiatry Res. 2021;297:113714.

209. Chakraborty T, Subbiah GK, Damade Y. Psychological Distress during COVID-19 Lockdown among Dental Students and Practitioners in India: A Cross-Sectional Survey. Eur J Dent. 2020;14:S70-S8.

210. Chang J, Yuan Y, Wang D. [Mental health status and its influencing factors among college students during the epidemic of COVID-19]. Nan Fang Yi Ke Da Xue Xue Bao. 2020.

211. Chang MC, Park D. Incidence of Post-Traumatic Stress Disorder After Coronavirus Disease. Healthcare (Basel). 2020;8(4):373-.

212. Chang YK, Hung CL, Timme S, Nosrat S, Chu CH. Exercise Behavior and Mood during the COVID-19 Pandemic in Taiwan: Lessons for the Future. Int J Environ Res Public Health. 2020;17(19):1-17.

213. Chao M, Chen XM, Liu TR, Yang HB, Hall BJ. Psychological distress and state boredom during the COVID-19 outbreak in China: the role of meaning in life and media use. Eur J Psychotraumato. 2020;11(1):1769379.

214. Chao M, Xue DN, Liu TR, Yang HB, Hall BJ. Media use and acute psychological outcomes during COVID-19 outbreak in China. Journal of Anxiety Disorders. 2020;74:102248.

215. Chapman DG, Thamrin C. Scientists in pyjamas: characterising the working arrangements and productivity of Australian medical researchers during the COVID-19 pandemic. Med J Aust. 2020;213(11):516-20.

216. Charles NE, Strong SJ, Burns LC, Bullerjahn MR, Serafine KM. Increased mood disorder symptoms, perceived stress, and alcohol use among college students during the COVID-19 pandemic. Psychiat Res. 2021;296:113706.

217. Chaturvedi K, Vishwakarma DK, Singh N. COVID-19 and its impact on education, social life and mental health of students: A survey. Child Youth Serv Rev. 2021;121:105866.

218. Chauhan VS, Chatterjee K, Chauhan KS, Prakash J, Srivastava K. Impact on anxiety of COVID-19 and lockdown. J Mar Med Soc. 2020;22(3):78-82.

219. Cheema M, Mitrev N, Hall L, Tiongson M, Ahlenstiel G, Kariyawasam V. Depression, anxiety and stress among patients with inflammatory bowel disease during the COVID-19 pandemic: Australian national survey. Bmj Open Gastroenter. 2021;8(1).

220. Cheikh Ismail L, Mohamad MN, Bataineh MF, Ajab A, Al-Marzouqi AM, Jarrar AH, et al. Impact of the Coronavirus Pandemic (COVID-19) Lockdown on Mental Health and Well-Being in the United Arab Emirates. Front Psychiatry. 2021;12:633230.

221. Cheikh Ismail L, Osaili TM, Mohamad MN, Al Marzouqi A, Jarrar AH, Zampelas A, et al. Assessment of eating habits and lifestyle during the coronavirus 2019 pandemic in the Middle East and North Africa region: a cross-sectional study. Br J Nutr. 2021;126(5):757-66.

222. Chen B, Sun J, Feng Y. How Have COVID-19 Isolation Policies Affected Young People's Mental Health? - Evidence From Chinese College Students. Front Psychol. 2020;11:1529.

223. Chen G, Gong J, Qi Z, Zhong S, Su T, Wang J, et al. The Psychological Status of General Population in Hubei Province During the COVID-19 Outbreak: A Cross-Sectional Survey Study. Front Public Health. 2021;9:622762.

224. Chen RN, Liang SW, Peng Y, Li XG, Chen JB, Tang SY, et al. Mental health status and change in living rhythms among college students in China during the COVID-19 pandemic: A large-scale survey. J Psychosom Res. 2020;137.

225. Chen SX, Ng JCK, Hui BPH, Au AKY, Wu WCH, Lam BCP, et al. Dual impacts of coronavirus anxiety on mental health in 35 societies. Sci Rep. 2021;11(1):8925.

226. Chen WL, Song SY, Yap KH. The Unintended Consequences of the Pandemic: The New Normal for College Students in South Korea and Taiwan. Front Public Health. 2021;9:598302.

227. Chen X, Zou Y, Gao H. Role of neighborhood social support in stress coping and psychological wellbeing during the COVID-19 pandemic: Evidence from Hubei, China. Health Place. 2021;69:102532.

228. Cheng C, Wang HY, Ebrahimi OV. Adjustment to a “New Normal:” Coping Flexibility and Mental Health Issues During the COVID-19 Pandemic. Frontiers in Psychiatry. 2021;12.

229. Cheng P, Casement MD, Kalmbach DA, Castelan AC, Drake CL. Digital cognitive behavioral therapy for insomnia promotes later health resilience during the coronavirus disease 19 (COVID-19) pandemic. Sleep. 2021;44(4).

230. Cherak SJ, Rosgen BK, Geddes A, Makuk K, Sudershan S, Peplinksi C, et al. Wellness in medical education: definition and five domains for wellness among medical learners during the COVID-19 pandemic and beyond. Med Educ Online. 2021;26(1):1917488.

231. Cherblanc J, Bergeron-Leclerc C, Maltais D, Cadell S, Gauthier G, Labra O, et al. Predictive Factors of Spiritual Quality of Life during the COVID-19 Pandemic: A Multivariate Analysis. J Relig Health. 2021;60(3):1475-93.

232. Cherdymova EI, Masalimova AR, Khairullina ER, Vasbieva DG, Ismailova NP, Kurbanov RA, et al. Peculiarities of Math Students Adaptation to Temporary Forced Isolation or Quarantine. EURASIA Journal of Mathematics, Science and Technology Education. 2020;16(11).

233. Cheung T, Lam SC, Lee PH, Xiang YT, Yip PSF, International Research Collaboration on C. Global Imperative of Suicidal Ideation in 10 Countries Amid the COVID-19 Pandemic. Front Psychiatry. 2020;11:588781.

234. Chhetri B, Goyal LM, Mittal M, Battineni G. Estimating the prevalence of stress among Indian students during the COVID-19 pandemic: A cross-sectional study from India. J Taibah Univ Med Sc. 2021;16(2):260-7.

235. Chi XL, Becker B, Yu Q, Willeit P, Jiao C, Huang LY, et al. Prevalence and Psychosocial Correlates of Mental Health Outcomes Among Chinese College Students During the Coronavirus Disease (COVID-19) Pandemic. Frontiers in Psychiatry. 2020;11:803.

236. Chih-Chien SY-HTM-CSSH-HL. Effects of Sports Massage on the Physiological and Mental Health of College Students Participating in a 7-Week Intermittent Exercises Program. International Journal of Environmental Research and Public Health. 2021;18(9):5013-.

237. Chitapure T, Bhodaji S, Zanwar M, Malani R. Impact of covid-19 pandemic lockdown on mental and physical health of students: A cross-sectional study. International Journal of Research in Pharmaceutical Sciences. 2020;11:1710-4.

238. Chodkiewicz J, Miniszewska J, Krajewska E, Bilinski P. Mental Health during the Second Wave of the COVID-19 Pandemic-Polish Studies. Int J Environ Res Public Health. 2021;18(7).

239. Choi EPH, Hui BPH, Wan EYF, Kwok JYY, Tam THL, Wu C. COVID-19 and Health-Related Quality of Life: A Community-Based Online Survey in Hong Kong. Int J Environ Res Public Health. 2021;18(6):1-12.

240. Choi S, Hong JY, Kim YJ, Park H. Predicting Psychological Distress Amid the COVID-19 Pandemic by Machine Learning: Discrimination and Coping Mechanisms of Korean Immigrants in the U.S. Int J Environ Res Public Health. 2020;17(17).

241. Chong YY, Chien WT, Cheng HY, Lamnisos D, Lubenko J, Presti G, et al. Patterns of Psychological Responses among the Public during the Early Phase of COVID-19: A Cross-Regional Analysis. International Journal of Environmental Research and Public Health. 2021;18(8).

242. Chung SY, Chou FH. Lessons learned from SARS to COVID-19 in the Taiwanese population. Asian J Psychiatr. 2020;54:102299.

243. Cici R, Yilmazel G. Determination of anxiety levels and perspectives on the nursing profession among candidate nurses with relation to the covid‐19 pandemic. Perspectives in Psychiatric Care. 2020.

244. Cindrich SL, Lansing JE, Brower CS, McDowell CP, Herring MP, Meyer JD. Associations Between Change in Outside Time Pre- and Post-COVID-19 Public Health Restrictions and Mental Health: Brief Research Report. Front Public Health. 2021;9:619129.

245. Clabaugh A, Duque JF, Fields LJ. Academic Stress and Emotional Well-Being in United States College Students Following Onset of the COVID-19 Pandemic. Front Psychol. 2021;12:628787.

246. Clarke C, Mullin M, McGrath D, Farrelly N. University students and study habits. Ir J Psychol Med. 2021:1-10.

247. Cleofas JV, Rocha ICN. Demographic, gadget and internet profiles as determinants of disease and consequence related COVID-19 anxiety among Filipino college students. Educ Inf Technol. 2021;26(6):6771-86.

248. Coakley KE, Lardier DT, Holladay KR, Amorim FT, Mechler H, Zuhl MN. Mental Health Severity Is Associated with Increases in Alcohol Consumption in Young Adult Students during the COVID-19 Pandemic. Alcohol Treat Q. 2021;39(3):328-41.

249. Coakley KE, Le H, Silva SR, Wilks A. Anxiety is associated with appetitive traits in university students during the COVID-19 pandemic. Nutr J. 2021;20(1):45.

250. Coffey CS, MacDonald BV, Shahrvini B, Baxter SL, Lander L. Student Perspectives on Remote Medical Education in Clinical Core Clerkships During the COVID-19 Pandemic. Med Sci Educ. 2020;30(4):1577-84.

251. Cohen AK, Hoyt LT, Dull B. A Descriptive Study of COVID-19-Related Experiences and Perspectives of a National Sample of College Students in Spring 2020. J Adolesc Health. 2020;67(3):369-75.

252. Coley RL, Baum CF. Retracted: Trends in mental health symptoms, service use, and unmet need for services among U.S. adults through the first 9 months of the COVID-19 pandemic. Transl Behav Med. 2021;11(10):1947-56.

253. Collado-Boira EJ, Ruiz-Palomino E, Salas-Media P, Folch-Ayora A, Muriach M, Balino P. "The COVID-19 outbreak"-An empirical phenomenological study on perceptions and psychosocial considerations surrounding the immediate incorporation of final-year Spanish nursing and medical students into the health system. Nurs Educ Today. 2020;92:104504.

254. Collins FE. Measuring COVID-19-related fear and threat in Australian, Indian, and Nepali university students. Pers Individ Dif. 2021;175:110693.

255. Conrad RC, Hahm HC, Koire A, Pinder-Amaker S, Liu CH. College student mental health risks during the COVID-19 pandemic: Implications of campus relocation. J Psychiatr Res. 2021;136:117-26.

256. Copeland WE, McGinnis E, Bai Y, Adams Z, Nardone H, Devadanam V, et al. Impact of COVID-19 Pandemic on College Student Mental Health and Wellness. Journal of the American Academy of Child & Adolescent Psychiatry. 2021;60(1):134-41.e2.

257. Coppola I, Rania N, Parisi R, Lagomarsino F. Spiritual Well-Being and Mental Health During the COVID-19 Pandemic in Italy. Front Psychiatry. 2021;12:626944.

258. Cordoș A-A, Bolboacă SD. Lockdown, Social Media exposure regarding COVID-19 and the relation with self-assessment depression and anxiety. Is the medical staff different? Int J Clin Pract. 2021;75(4):e13933.

259. Cori L, Curzio O, Adorni F, Prinelli F, Noale M, Trevisan C, et al. Fear of COVID-19 for Individuals and Family Members: Indications from the National Cross-Sectional Study of the EPICOVID19 Web-Based Survey. Int J Environ Res Public Health. 2021;18(6).

260. Cornejo LLC, Campos MAJ, Guardia MDV, Rivera ERE. Welfare and Psychological Coping of University Students in a Confined State by Covid-19. Rev Incl. 2020;7:377-98.

261. Corr P. Subjective well-being, self-care, and mental health help-seeking tendencies among DACA students at a large public institution in the mid-Atlantic United States: ProQuest Information & Learning; 2021.

262. Coughenour C, Gakh M, Pharr JR, Bungum T, Jalene S. Changes in Depression and Physical Activity Among College Students on a Diverse Campus After a COVID-19 Stay-at-Home Order. J Commun Health. 2021;46(4):758-66.

263. Coulthard H, Sharps M, Cunliffe L, van den Tol A. Eating in the lockdown during the Covid 19 pandemic; self-reported changes in eating behaviour, and associations with BMI, eating style, coping and health anxiety. Appetite. 2021;161:105082.

264. Courtier N, Brown P, Mundy L, Pope E, Chivers E, Williamson K. Expectations of therapeutic radiography students in Wales about transitioning to practice during the Covid-19 pandemic as registrants on the HCPC temporary register. Radiography. 2021;27(2):316-21.

265. Coyle C, Ghazi H, Georgiou I. The mental health and well-being benefits of exercise during the COVID-19 pandemic: a cross-sectional study of medical students and newly qualified doctors in the UK. Ir J Med Sci. 2021;190(3):925-6.

266. Cueto LJ, Agaton CB. Pandemic and Typhoon: Positive Impacts of a Double Disaster on Mental Health of Female Students in the Philippines. Behav Sci (Basel). 2021;11(5).

267. Cueva MAL, Terrones SAC. Impact of virtual classes on the university students in the context of COVID-19 quarantine: The case of the PUCP. Propos Represent. 2020;8:15-.

268. Curran J. Not quite back to business as usual. Medicus. 2021;61(3):45-.

269. Cuschieri S, Calleja Agius J. Spotlight on the Shift to Remote Anatomical Teaching During Covid-19 Pandemic: Perspectives and Experiences from the University of Malta. Anat Sci Educ. 2020;13(6):671-9.

270. Czeisler ME, Lane RI, Wiley JF, Czeisler CA, Howard ME, Rajaratnam SMW. Follow-up Survey of US Adult Reports of Mental Health, Substance Use, and Suicidal Ideation During the COVID-19 Pandemic, September 2020. Jama Netw Open. 2021;4(2):e2037665.

271. Dadfar M, Mohaghegh F, Sanadgol S, Eslami M. The fear of COVID-19 scale (FCV-19S): A study of Iranian University Students. Mankind Quarterly. 2021;61(3):707-22.

272. Dagnino P, Anguita V, Escobar K, Cifuentes S. Psychological Effects of Social Isolation Due to Quarantine in Chile: An Exploratory Study. Frontiers in Psychiatry. 2020;11.

273. Daly M, Sutin AR, Robinson E. Longitudinal changes in mental health and the COVID-19 pandemic: evidence from the UK Household Longitudinal Study. Psychol Med. 2020:1-10.

274. Daly Z, Slemon A, Richardson CG, Salway T, McAuliffe C, Gadermann AM, et al. Associations between periods of COVID-19 quarantine and mental health in Canada. Psychiat Res. 2021;295.

275. Dang S, Penney LS, Trivedi R, Noel PH, Pugh MJ, Finley E, et al. Caring for Caregivers During COVID-19. J Am Geriatr Soc. 2020;68(10):2197-201.

276. Dangal MR, Bajracharya LS. Students Anxiety Experiences during COVID-19 in Nepal. Kathmandu Univ Med J (KUMJ). 2020;18(70):53-7.

277. Darvishi E, Golestan S, Demehri F, Jamalnia S. A Cross-Sectional Study on Cognitive Errors and Obsessive-Compulsive Disorders among Young People During the Outbreak of Coronavirus Disease 2019. Activitas Nervosa Superior. 2020;62(4):137-42.

278. Davico C, Ghiggia A, Marcotulli D, Ricci F, Amianto F, Vitiello B. Psychological Impact of the COVID-19 Pandemic on Adults and Their Children in Italy. Frontiers in Psychiatry. 2021;12:572997.

279. David ME, Roberts JA. Smartphone Use during the COVID-19 Pandemic: Social Versus Physical Distancing. International Journal of Environmental Research and Public Health. 2021;18(3).

280. Dayton L, Schneider K, Strickland JC, Latkin C. Determinants of worry using the SARS-CoV-19 worry (CoV-Wo) scale among United States residents. Journal of Community Psychology. 2021;49(5):1487-504.

281. De Los Santos JAA, Labrague LJ, Falguera CC. Fear of covid‐19, poor quality of sleep, irritability, and intention to quit school among nursing students: A cross‐sectional study. Perspectives in Psychiatric Care. 2021.

282. De Man J, Buffel V, van de Velde S, Bracke P, Van Hal GF, Wouters E, et al. Disentangling depression in Belgian higher education students amidst the first COVID-19 lockdown (April-May 2020). Arch Public Health. 2021;79(1):3.

283. De Morelos E, de Morelos A, de Nayarit A. Impacto psicológico en estudiantes universitarios mexicanos por confinamiento durante la pandemia por Covid-19. Preprint/Version. 2020;1.

284. Debbarma I, Durai T. Educational disruption: Impact of COVID-19 on students from the Northeast states of India. Child Youth Serv Rev. 2021;120:105769.

285. Debowska A, Horeczy B, Boduszek D, Dolinski D. A repeated cross-sectional survey assessing university students' stress, depression, anxiety, and suicidality in the early stages of the COVID-19 pandemic in Poland. Psychol Med. 2020:1-4.

286. Dębski P, Florek S, Piegza M, Pudlo R, Gorczyca PW. Is it good to be resilient during the COVID‑19 period? The role of ego-resiliency in the intensity of symptoms of anxiety, alcohol use and aggression among Polish people. Int J Occup Med Environ Health. 2021.

287. Defeyter MA, Stretesky PB, Long MA, Furey S, Reynolds C, Porteous D, et al. Mental Well-Being in UK Higher Education During Covid-19: Do Students Trust Universities and the Government? Front Public Health. 2021;9:646916.

288. Delaney RK, Locke A, Pershing ML, Geist C, Clouse E, Precourt Debbink M, et al. Experiences of a Health System's Faculty, Staff, and Trainees' Career Development, Work Culture, and Childcare Needs During the COVID-19 Pandemic. JAMA Netw Open. 2021;4(4):e213997.

289. Deng CH, Wang JQ, Zhu LM, Liu HW, Guo Y, Peng XH, et al. Association of Web-Based Physical Education With Mental Health of College Students in Wuhan During the COVID-19 Outbreak: Cross-Sectional Survey Study. J Med Internet Res. 2020;22(10):e21301.

290. Dhahri AA, Arain SY, Memon AM, Rao A, Medical Education Pakistan collaborator g, Mian MA. "The psychological impact of COVID-19 on medical education of final year students in Pakistan: A cross-sectional study". Ann Med Surg (Lond). 2020;60:445-50.

291. Dhar BK, Ayittey FK, Sarkar SM. Impact of COVID-19 on Psychology among the University Students. Glob Chall. 2020;4(11):2000038.

292. Di Blasi M, Gullo S, Mancinelli E, Freda MF, Esposito G, Gelo OCG, et al. Psychological distress associated with the COVID-19 lockdown: A two-wave network analysis. J Affect Disord. 2021;284:18-26.

293. Di Giuseppe M, Zilcha-Mano S, Prout TA, Perry JC, Orrù G, Conversano C. Psychological Impact of Coronavirus Disease 2019 Among Italians During the First Week of Lockdown. Frontiers in Psychiatry. 2020;11.

294. Di Napoli I, Guidi E, Arcidiacono C, Esposito C, Marta E, Novara C, et al. Italian Community Psychology in the COVID-19 Pandemic: Shared Feelings and Thoughts in the Storytelling of University Students. Front Psychol. 2021;12:571257.

295. Di Renzo L, Gualtieri P, Cinelli G, Bigioni G, Soldati L, Attina A, et al. Psychological Aspects and Eating Habits during COVID-19 Home Confinement: Results of EHLC-COVID-19 Italian Online Survey. Nutrients. 2020;12(7):1-14.

296. Díaz-Jiménez RM, Caravaca-Sánchez F, Martín-Cano MC, De la Fuente-Robles YM. Anxiety levels among social work students during the covid-19 lockdown in spain. Social Work in Health Care. 2020.

297. DiClemente RJ, Capasso A, Ali SH, Jones AM, Foreman J, Tozan Y. Knowledge, beliefs, mental health, substance use, and behaviors related to the COVID-19 pandemic among US adults: a national online survey. J Public Health-Heid. 2022;30(8):2069-79.

298. Dillard AJ, Meier BP. Trait mindfulness is negatively associated with distress related to COVID-19. Personality and Individual Differences. 2021;179:110955-.

299. Dimmock J, Krause AE, Rebar A, Jackson B. Relationships between social interactions, basic psychological needs, and wellbeing during the COVID-19 pandemic. Psychol Health. 2022;37(4):457-69.

300. Ding KL, Yang JZ, Chin MK, Sullivan L, Demirhan G, Violant-Holz V, et al. Mental Health among Adults during the COVID-19 Pandemic Lockdown: A Cross-Sectional Multi-Country Comparison. International Journal of Environmental Research and Public Health. 2021;18(5).

301. Diniz TA, Christofaro DGD, Tebar WR, Cucato GG, Botero JP, Correia MA, et al. Reduction of Physical Activity Levels During the COVID-19 Pandemic Might Negatively Disturb Sleep Pattern. Frontiers in Psychology. 2020;11:586157.

302. Dodd RH, Dadaczynski K, Okan O, McCaffery KJ, Pickles K. Psychological Wellbeing and Academic Experience of University Students in Australia during COVID-19. Int J Environ Res Public Health. 2021;18(3).

303. Doom JR, Seok D, Narayan AJ, Fox KR. Adverse and Benevolent Childhood Experiences Predict Mental Health During the COVID-19 Pandemic. Advers Resil Sci. 2021;2(3):193-204.

304. Dr. Adeel A, Dr. Hania S, Dr. Hyder Ali K, Dr. Suneel P, Areeba Raza K, Dr. Fauziah R. Perceived Risk and Distress related to COVID-19: Comparing Healthcare versus non-Healthcare Workers of Pakistan. 2020.

305. Dragun R, Veček NN, Marendić M, Pribisalić A, Đivić G, Cena H, et al. Have Lifestyle Habits and Psychological Well-Being Changed among Adolescents and Medical Students Due to COVID-19 Lockdown in Croatia? Nutrients. 2021;13(1).

306. Dratva J, Zysset A, Schlatter N, von Wyl A, Huber M, Volken T. Swiss University Students' Risk Perception and General Anxiety during the COVID-19 Pandemic. Int J Environ Res Public Health. 2020;17(20).

307. Drissi N, Alhmoudi A, Al Nuaimi H, Alkhyeli M, Alsalami S, Ouhbi S. Investigating the Impact of COVID-19 Lockdown on the Psychological Health of University Students and Their Attitudes Toward Mobile Mental Health Solutions: Two-Part Questionnaire Study. Jmir Form Res. 2020;4(10):e19876.

308. Dsouza DD, Quadros S, Hyderabadwala ZJ, Mamun MA. Aggregated COVID-19 suicide incidences in India: Fear of COVID-19 infection is the prominent causative factor. Psychiat Res. 2020;290:113145.

309. Du C, Zan MCH, Cho MJ, Fenton JI, Hsiao PY, Hsiao R, et al. Health Behaviors of Higher Education Students from 7 Countries: Poorer Sleep Quality during the COVID-19 Pandemic Predicts Higher Dietary Risk. Clocks Sleep. 2021;3(1):12-30.

310. Du C, Zan MCH, Cho MJ, Fenton JI, Hsiao PY, Hsiao R, et al. The Effects of Sleep Quality and Resilience on Perceived Stress, Dietary Behaviors, and Alcohol Misuse: A Mediation-Moderation Analysis of Higher Education Students from Asia, Europe, and North America during the COVID-19 Pandemic. Nutrients. 2021;13(2).

311. Du C, Zan MCH, Cho MJ, Fenton JI, Hsiao PY, Hsiao R, et al. Increased Resilience Weakens the Relationship between Perceived Stress and Anxiety on Sleep Quality: A Moderated Mediation Analysis of Higher Education Students from 7 Countries. Clocks Sleep. 2020;2(3):334-53.

312. Du J, Mayer G, Hummel S, Oetjen N, Gronewold N, Zafar A, et al. Mental Health Burden in Different Professions During the Final Stage of the COVID-19 Lockdown in China: Cross-sectional Survey Study. J Med Internet Res. 2020;22(12):e24240.

313. Duan H, Yan L, Ding X, Gan Y, Kohn N, Wu J. Impact of the COVID-19 pandemic on mental health in the general Chinese population: Changes, predictors and psychosocial correlates. Psychiatry Res. 2020;293:113396.

314. Duay M, Morgieve M, Niculita-Hirzel H. Sudden Changes and Their Associations with Quality of Life during COVID-19 Lockdown: A Cross-Sectional Study in the French-Speaking Part of Switzerland. International Journal of Environmental Research and Public Health. 2021;18(9).

315. Dubey N, Podder P, Pandey D. Knowledge of COVID-19 and Its Influence on Mindfulness, Cognitive Emotion Regulation and Psychological Flexibility in the Indian Community. Front Psychol. 2020;11:589365.

316. Duong CD. The impact of fear and anxiety of Covid-19 on life satisfaction: Psychological distress and sleep disturbance as mediators. Personality and Individual Differences. 2021;178:110869-.

317. Dur re SMM, Ahmed Z, Mughal Z, Shaikh B, Ahmer A, Sultana R, et al. Impact of COVID-Pandemic on the Mental Health of Medical Students of Sindh Province, Pakistan. Journal of Pharmaceutical Research International. 2021;33(15):56-60.

318. Durak VA, Gunay S, Sigirli D, Akova B, Armagan E. COVID-19 Pandemic and Anxiety Related Factors in Patients Treated in the Emergency Department. Signa Vitae. 2020;16(2):167-74.

319. Duran S, Erkin O. Psychologic distress and sleep quality among adults in Turkey during the COVID-19 pandemic. Prog Neuro-Psychoph. 2021;107:110254.

320. Dutheil F, Mondillon L, Navel V. PTSD as the second tsunami of the SARS-Cov2 pandemic. Psychological Medicine. 2020.

321. Dwivedi D, Kaur N, hi A, Shukla S, Tripathi S. Perception of stress among medical undergraduate during coronavirus disease-19 pandemic on exposure to online teaching. National Journal of Physiology, Pharmacy and Pharmacology. 2020;10(8):657-62.

322. Dzhambov AM, Lercher P, Browning MHEM, Stoyanov D, Petrova N, Novakov S, et al. Does greenery experienced indoors and outdoors provide an escape and support mental health during the COVID-19 quarantine? Environmental Research. 2021;196:110420.

323. Džuka J, Klučárová Z, Babinčák P. Covid-19 in slovakia: Economic, social and psychological factors of subjective well-being and depressive symptoms during a pandemic. Ceskoslovenska Psychologie. 2021;65(2):125-45.

324. Ebrahimi HK, Salami S, Jafarnejad S, Dezfouli SMM, Delavar MA, Esmaeilian S. Relationship between anxiety and resilience of coronavirus (COVID-19) in pediatric and emergency assistants of Iran Medical Science University, 2021. Pakistan Journal of Medical & Health Sciences. 2021;15(3):984-91.

325. Eden AL, Johnson BK, Reinecke L, Grady SM. Media for Coping During COVID-19 Social Distancing: Stress, Anxiety, and Psychological Well-Being. Front Psychol. 2020;11:577639.

326. El Keshky MES, Alsabban AM, Basyouni SS. The psychological and social impacts on personal stress for residents quarantined for COVID-19 in Saudi Arabia. Arch Psychiatr Nurs. 2021;35(3):311-6.

327. El Morr C, Ritvo P, Ahmad F, Moineddin R. Effectiveness of an 8-Week Web-Based Mindfulness Virtual Community Intervention for University Students on Symptoms of Stress, Anxiety, and Depression: Randomized Controlled Trial. JMIR Ment Health. 2020;7(7):e18595.

328. El-Monshed AH, El-Adl AA, Ali AS, Loutfy A. University students under lockdown, the psychosocial effects and coping strategies during COVID-19 pandemic: A cross sectional study in Egypt. J Am Coll Health. 2022;70(3):679-90.

329. Ela MZ, Shohel TA, Shovo TE, Khan L, Jahan N, Hossain MT, et al. Prolonged lockdown and academic uncertainties in Bangladesh: A qualitative investigation during the COVID-19 pandemic. Heliyon. 2021;7(2):e06263.

330. Elbogen EB, Lanier M, Blakey SM, Wagner HR, Tsai J. Suicidal ideation and thoughts of self-harm during the COVID-19 pandemic: The role of COVID-19-related stress, social isolation, and financial strain. Depress Anxiety. 2021.

331. Elemo AS, Ahmed AH, Kara E, Zerkeshi MK. The Fear of COVID-19 and Flourishing: Assessing the Mediating Role of Sense of Control in International Students. Int J Ment Health Addict. 2022;20(4):2408-18.

332. Elhadi M, Alsoufi A, Msherghi A, Alshareea E, Ashini A, Nagib T, et al. Psychological Health, Sleep Quality, Behavior, and Internet Use Among People During the COVID-19 Pandemic: A Cross-Sectional Study. Frontiers in Psychiatry. 2021;12.

333. Elhadi M, Buzreg A, Bouhuwaish A, Khaled A, Alhadi A, Msherghi A, et al. Psychological Impact of the Civil War and COVID-19 on Libyan Medical Students: A Cross-Sectional Study. Front Psychol. 2020;11:570435.

334. Elhai JD, McKay D, Yang HB, Minaya C, Montag C, Asmundson GJG. Health anxiety related to problematic smartphone use and gaming disorder severity during COVID-19: Fear of missing out as a mediator. Hum Behav Emerg Tech. 2021;3(1):137-46.

335. Elhaty IA, Elhadary T. A pre-assessment of the educational programs intended to be applied by Turkish universities in light of the continuing spread of COVID-19. European Journal of Molecular and Clinical Medicine. 2020;7(6):1-20.

336. Elmer T, Mepham K, Stadtfeld C. Students under lockdown: Comparisons of students' social networks and mental health before and during the COVID-19 crisis in Switzerland. Plos One. 2020;15(7).

337. Elsalem L, Al-Azzam N, Jum'ah AA, Obeidat N, Sindiani AM, Kheirallah KA. Stress and behavioral changes with remote E-exams during the Covid-19 pandemic: A cross-sectional study among undergraduates of medical sciences. Ann Med Surg. 2020;60:271-9.

338. Elsharkawy NB, Abdelaziz EM. Levels of fear and uncertainty regarding the spread of coronavirus disease (COVID-19) among university students. Perspectives in Psychiatric Care. 2021;57(3):1356-64.

339. Enyew Getaneh MBSWMSANYM. The Psychological Impact of COVID-19 Pandemic on Graduating Class Students at the University of Gondar, Northwest Ethiopia. Psychology Research and Behavior Management. 2021;14:109-22.

340. Ersin F, Kartal M. The determination of the perceived stress levels and health-protective behaviors of nursing students during the COVID-19 pandemic. Perspect Psychiatr Care. 2020;57(2):929-35.

341. Eryilmaz A, Basal A. Student and Teacher Perspectives: Developing the Scale of Coping Strategies for Pessimism and Subjective Well-Being Model Based on Coping Strategies for COVID-19 and Goal Striving. International Online Journal of Education and Teaching. 2021;8(1):546-63.

342. Escolà-Gascón Á, Marín FX, Rusiñol J, Gallifa J. Evidence of the psychological effects of pseudoscientific information about COVID-19 on rural and urban populations. Psychiatry Res. 2021;295:113628.

343. Essadek A, Rabeyron T. Mental health of French students during the Covid-19 pandemic. J Affect Disord. 2020;277:392-3.

344. Essangri H, Sabir M, Benkabbou A, Majbar MA, Amrani L, Ghannam A, et al. Predictive Factors for Impaired Mental Health among Medical Students during the Early Stage of the COVID-19 Pandemic in Morocco. Am J Trop Med Hyg. 2021;104(1):95-102.

345. Esteves CS, de Oliveira CR, Argimon IIL. Social Distancing: Prevalence of Depressive, Anxiety, and Stress Symptoms Among Brazilian Students During the COVID-19 Pandemic. Front Public Health. 2020;8:589966.

346. Evans S, Alkan E, Bhangoo JK, Tenenbaum H, Ng-Knight T. Effects of the COVID-19 lockdown on mental health, wellbeing, sleep, and alcohol use in a UK student sample. Psychiat Res. 2021;298:113819.

347. Eweida RS, Rashwan ZI, Desoky GM, Khonji LM. Mental strain and changes in psychological health hub among intern-nursing students at pediatric and medical-surgical units amid ambience of COVID-19 pandemic: A comprehensive survey. Nurse Educ Pract. 2020;49:102915.

348. Fabelo-Roche JR, Iglesias-More S, Gomez-Garcia AM. Persons with Substance Abuse Disorders and Other Addictions: Coping with the COVID-19 Pandemic. MEDICC Rev. 2021;23(2):55.

349. Faez M, Hadi J, Abdalqader M, Assem H, Ads HO, Ghazi HF. Impact of lockdown due to covid-19 on mental health among students in private university at selangor. European Journal of Molecular and Clinical Medicine. 2020;7(11):508-17.

350. Faghankhani M, Sodagari F, Shokrani M, Baradaran HR, Adabi A, Zabihi M, et al. Perceived Stress among Iranians during COVID-19 Pandemic; Stressors and Coping Mechanisms: A Mixed-methods Approach: Stress percu chez les Iraniens durant la pandemie de la COVID-19; stresseurs et mecanismes d'adaptation: Une approche de methodes mixtes. Can J Psychiat. 2021:7067437211004881.

351. Faisal RA, Jobe MC, Ahmed O, Sharker T. Mental Health Status, Anxiety, and Depression Levels of Bangladeshi University Students During the COVID-19 Pandemic. Int J Ment Health Addict. 2022;20(3):1500-15.

352. Fakhar EAKM, Bhutta ZA, Shabbir S, Akhtar M. Psychosocial impact of COVID-19 outbreak on international students living in Hubei province, China. Travel Med Infect Dis. 2020;37:101712.

353. Fan J, Smith AP. Information Overload, Wellbeing and COVID-19: A Survey in China. Behav Sci (Basel). 2021;11(5).

354. Fan J-qYJ-j. Role of solution-focused intervention in improving mood of college students during the coronavirus disease 2019 pandemic-A study in a university of Hangzhou. Huanjing yu Zhiye Yixue = Journal of Environmental &amp; Occupational Medicine. 2020;37(9):858-.

355. Fanaj N, Mustafa S. Depression Measured by PHQ-9 in Kosovo during the COVID-19 Outbreak: An Online Survey. Psychiatr Danub. 2021;33(1):95-100.

356. Fanari ASC. Longitudinal effects of U.S. students&#039; reentry shock on psychological health after returning home during the COVID-19 global pandemic. International Journal of Intercultural Relations. 2021;82:298-310.

357. Fathi A, Sadeghi S, Rad AAM, Rahnmo SS, Rostami H, Abdolmohammadi K. The Role of Cyberspace Use on Lifestyle Promoting Health and Coronary Anxiety in Young People. Iran J Psychiat Clin. 2020;26(3):332-46.

358. Fathimahhayati LD, Pawitra TA, Tambunan W. Analisis Ergonomi Pada Perkuliahan Daring Menggunakan Smartphone Selama Masa Pandemi COVID-19: Studi Kasus Mahasiswa Prodi Teknik Industri Universitas Mulawarman. Operations Excellence: Journal Of Applied Industrial Engineering. 2020;12(3):308-17.

359. Faulkner J, O'Brien WJ, McGrane B, Wadsworth D, Batten J, Askew CD, et al. Physical activity, mental health and well-being of adults during initial COVID-19 containment strategies: A multi-country cross-sectional analysis. J Sci Med Sport. 2021;24(4):320-6.

360. Favieri F, Forte G, Tambelli R, Casagrande M. The Italians in the Time of Coronavirus: Psychosocial Aspects of the Unexpected COVID-19 Pandemic. Front Psychiatry. 2021;12:551924.

361. Fawaz M, Al Nakhal M, Itani M. COVID-19 quarantine stressors and management among Lebanese students: a qualitative study. Curr Psychol. 2021:1-8.

362. Fawaz M, Samaha A. COVID-19 quarantine: Post-traumatic stress symptomatology among Lebanese citizens. Int J Soc Psychiatr. 2020;66(7):666-74.

363. Fawaz M, Samaha A. E-learning: Depression, anxiety, and stress symptomatology among Lebanese university students during COVID-19 quarantine. Nurs Forum. 2021;56(1):52-7.

364. Fedorenko EJ, Kibbey MM, Contrada RJ, Farris SG. Psychosocial predictors of virus and social distancing fears in undergraduate students living in a US COVID-19 "hotspot". Cogn Behav Ther. 2021;50(3):217-33.

365. Fekih-Romdhane F, Dissem N, Cheour M. How did Tunisian university students cope with fear of COVID-19? A comparison across schizotypy features. Pers Individ Dif. 2021;178:110872.

366. Feng SY, Zhang QC, Ho SMY. Fear and anxiety about COVID-19 among local and overseas Chinese university students. Health Soc Care Comm. 2021;29(6):E249-E58.

367. Feng Y, Zhang Y. Evaluation and Analysis of Mental Health Level of College Students With Financial Difficulties Under the Background of COVID-19. Front Psychol. 2021;12:649195.

368. Feng Y, Zong M, Yang ZZ, Gu W, Dong D, Qiao ZH. When altruists cannot help: the influence of altruism on the mental health of university students during the COVID-19 pandemic. Globalization Health. 2020;16(1):61.

369. Fernández Cruz M, Álvarez Rodríguez J, Ávalos Ruiz I, Cuevas López M, de Barros Camargo C, Díaz Rosas F, et al. Evaluation of the Emotional and Cognitive Regulation of Young People in a Lockdown Situation Due to the Covid-19 Pandemic. Front Psychol. 2020;11:565503.

370. Fernández RS, Crivelli L, Guimet NM, Allegri RF, Pedreira ME. Psychological distress associated with COVID-19 quarantine: Latent profile analysis, outcome prediction and mediation analysis. Journal of Affective Disorders. 2020;277:75-84.

371. Fernández-Castillo A. State-Anxiety and Academic Burnout Regarding University Access Selective Examinations in Spain During and After the COVID-19 Lockdown. Front Psychol. 2021;12:621863.

372. Ferreira LC, Amorim RS, Melo Campos FM, Cipolotti R. Mental health and illness of medical students and newly graduated doctors during the pandemic of SARS-Cov-2/COVID-19. Plos One. 2021;16(5):e0251525.

373. Ferreira LN, Pereira LN, da Fe Bras M, Ilchuk K. Quality of life under the COVID-19 quarantine. Qual Life Res. 2021;30(5):1389-405.

374. Feter N, Caputo EL, Doring IR, Leite JS, Cassuriaga J, Reichert FF, et al. Sharp increase in depression and anxiety among Brazilian adults during the COVID-19 pandemic: findings from the PAMPA cohort. Public Health. 2021;190:101-7.

375. Fidanci I, Aksoy H, Yengil Taci D, Ayhan Baser D, Cankurtaran M. Evaluation of the effect of the Covid-19 pandemic on smoking addiction levels. Int J Clin Pract. 2021;75(5):e14012.

376. Findyartini A, Anggraeni D, Husin JM, Greviana N. Exploring medical students' professional identity formation through written reflections during the COVID-19 pandemic. Journal of Public Health Research. 2020;9:4-10.

377. Fiorillo A, Sampogna G, Giallonardo V, Del Vecchio V, Luciano M, Albert U, et al. Effects of the lockdown on the mental health of the general population during the COVID-19 pandemic in Italy: Results from the COMET collaborative network. Eur Psychiatry. 2020;63(1):e87.

378. Firkey MK, Sheinfil AZ, Woolf-King SE. Substance use, sexual behavior, and general well-being of U.S. college students during the COVID-19 pandemic: A brief report. J Am Coll Health. 2021:1-7.

379. First JM, Shin H, Ranjit YS, Houston JB. COVID-19 Stress and Depression: Examining Social Media, Traditional Media, and Interpersonal Communication. Journal of Loss and Trauma. 2021;26(2):101-15.

380. Fish JN, McInroy LB, Paceley MS, Williams ND, Henderson S, Levine DS, et al. "I'm Kinda Stuck at Home With Unsupportive Parents Right Now": LGBTQ Youths' Experiences With COVID-19 and the Importance of Online Support. J Adolescent Health. 2020;67(3):450-2.

381. Fitzgerald A, Konrad S. Transition in learning during COVID-19: Student nurse anxiety, stress, and resource support. Nurs Forum. 2021;56(2):298-304.

382. Fitzpatrick KM, Drawve G, Harris C. Facing new fears during the COVID-19 pandemic: The State of America's mental health. J Anxiety Disord. 2020;75:102291.

383. Flaudias V, Iceta S, Zerhouni O, Rodgers RF, Billieux J, Llorca PM, et al. COVID-19 pandemic lockdown and problematic eating behaviors in a student population. J Behav Addict. 2020;9(3):826-35.

384. Flaudias V, Zerhouni O, Pereira B, Cherpitel CJ, Boudesseul J, de Chazeron I, et al. The Early Impact of the COVID-19 Lockdown on Stress and Addictive Behaviors in an Alcohol-Consuming Student Population in France. Front Psychiatry. 2021;12:628631.

385. Fong BF, Wong MCS, Law VTS, Lo MF, Ng TKC, Yee HHL, et al. Relationships between Physical and Social Behavioural Changes and the Mental Status of Homebound Residents in Hong Kong during the COVID-19 Pandemic. International Journal of Environmental Research and Public Health. 2020;17(18).

386. Ford MB. Social distancing during the COVID-19 pandemic as a predictor of daily psychological, social, and health-related outcomes. J Gen Psychol. 2021;148(3):249-71.

387. Ford T, John A, Gunnell D. Mental health of children and young people during pandemic. BMJ. 2021;372:n614.

388. Fornili M, Petri D, Berrocal C, Fiorentino G, Ricceri F, Macciotta A, et al. Psychological distress in the academic population and its association with socio-demographic and lifestyle characteristics during COVID-19 pandemic lockdown: Results from a large multicenter Italian study. Plos One. 2021;16(3):e0248370.

389. Forster M, Rogers C, Sussman SY, Yu S, Rahman T, Zeledon H, et al. Adverse childhood experiences and problematic smartphone use among college students: Findings from a pilot study. Addict Behav. 2021;117.

390. Fortgang RG, Wang SB, Millner AJ, Reid-Russell A, Beukenhorst AL, Kleiman EM, et al. Increase in Suicidal Thinking During COVID-19. Clin Psychol Sci. 2021;9(3):482-8.

391. Fountoulakis KN, Exadactylos A, Anastasiadis K, Papaioannou N, Javed A. The international initiatives of the collaboration between the Aristotle University of Thessaloniki School of Medicine, the Panhellenic Medical Association and the World Psychiatric Association, concerning mental health during the COVID-19 outbreak. Psychiatriki. 2020;31(4):289-92.

392. Frontini R, Rebelo-Goncalves R, Amaro N, Salvador R, Matos R, Morouco P, et al. The Relationship Between Anxiety Levels, Sleep, and Physical Activity During COVID-19 Lockdown: An Exploratory Study. Frontiers in Psychology. 2021;12.

393. Fruehwirth JC, Biswas S, Perreira KM. The Covid-19 pandemic and mental health of first-year college students: Examining the effect of Covid-19 stressors using longitudinal data. Plos One. 2021;16(3):e0247999.

394. Fu W, Wang C, Zou L, Guo Y, Lu Z, Yan S, et al. Psychological health, sleep quality, and coping styles to stress facing the COVID-19 in Wuhan, China. Transl Psychiatry. 2020;10(1):225.

395. Fu W, Yan S, Zong Q, Anderson-Luxford D, Song X, Lv Z, et al. Mental health of college students during the COVID-19 epidemic in China. J Affect Disord. 2021;280(Pt A):7-10.

396. Fuentes A, Caballero J, Ip E, Owens R, Jacobs R. Coping, resilience, and emotional wellbeing in pharmacy students during the COVID-19 pandemic. JACCP Journal of the American College of Clinical Pharmacy. 2020;3(8):1637.

397. Fullana MA, Hidalgo-Mazzei D, Vieta E, Radua J. Coping behaviors associated with decreased anxiety and depressive symptoms during the COVID-19 pandemic and lockdown. J Affect Disord. 2020;275:80-1.

398. Fuse-Nagase Y, Kuroda T, Watanabe J. Mental health of university freshmen in Japan during the COVID-19 pandemic: Screening with Kessler psychological distress scale (K6). Asian J Psychiatr. 2020;54:102407.

399. Gabrielli S, Rizzi S, Bassi G, Carbone S, Maimone R, Marchesoni M, et al. Engagement and Effectiveness of a Healthy-Coping Intervention via Chatbot for University Students During the COVID-19 Pandemic: Mixed Methods Proof-of-Concept Study. JMIR Mhealth Uhealth. 2021;9(5):e27965.

400. Gacek M, Krzywoszanski L. Symptoms of Anxiety and Depression in Students With Developmental Disabilities During COVID-19 Lockdown in Poland. Front Psychiatry. 2021;12:576867.

401. Gaeta ML, Gaeta L, Rodriguez MDS. The Impact of COVID-19 Home Confinement on Mexican University Students: Emotions, Coping Strategies, and Self-Regulated Learning. Front Psychol. 2021;12:642823.

402. Gaiha SM, Lempert LK, Halpern-Felsher B. Underage Youth and Young Adult e-Cigarette Use and Access Before and During the Coronavirus Disease 2019 Pandemic. Jama Netw Open. 2020;3(12):e2027572.

403. Galic M, Mustapic L, Simunic A, Sic L, Cipolletta S. COVID-19 Related Knowledge and Mental Health: Case of Croatia. Front Psychol. 2020;11:567368.

404. Galindo-Vazquez O, Ramirez-Orozco M, Costas-Muniz R, Mendoza-Contreras LA, Calderillo-Ruiz G, Meneses-Garcia A. Symptoms of anxiety, depression and self-care behaviors during the COVID-19 pandemic in the general population. Gac Med Mex. 2020;156(4):298-305.

405. Gallego-Gomez JI, Campillo-Cano M, Carrion-Martinez A, Balanza S, Rodriguez-Gonzalez-Moro MT, Simonelli-Munoz AJ, et al. The COVID-19 Pandemic and Its Impact on Homebound Nursing Students. Int J Environ Res Public Health. 2020;17(20).

406. Galvão DdSRNFCdCCSVRTdNFAA. Psychosocial aspects of nursing students during the COVID-19 Pandemic. Enferm foco (Brasília). 2020;11(2):143-7.

407. Galvin J, Richards G, Smith AP. A Longitudinal Cohort Study Investigating Inadequate Preparation and Death and Dying in Nursing Students: Implications for the Aftermath of the COVID-19 Pandemic. Front Psychol. 2020;11:2206.

408. Ganson KT, Tsai AC, Weiser SD, Benabou SE, Nagata JM. Job Insecurity and Symptoms of Anxiety and Depression Among U.S. Young Adults During COVID-19. J Adolesc Health. 2021;68(1):53-6.

409. Gao C, Scullin MK. Sleep health early in the coronavirus disease 2019 (COVID-19) outbreak in the United States: integrating longitudinal, cross-sectional, and retrospective recall data. Sleep Med. 2020;73:1-10.

410. Garboczy S, Szeman-Nagy A, Ahmad MS, Harsanyi S, Ocsenas D, Rekenyi V, et al. Health anxiety, perceived stress, and coping styles in the shadow of the COVID-19. BMC Psychol. 2021;9(1):53.

411. Garcia DT, Akinkugbe AA, Mosavel M, Smith CS, Brickhouse TH. COVID-19 and Dental and Dental Hygiene Students' Career Plans. JDR Clin Trans Res. 2021;6(2):153-60.

412. Garcia MRT, Tarazona ZEM, Natividad PM. Disorders of the emotions as a consequence of COVID-19 and the confinement in university of the different schools of the Hermilio Valdizan National University. Revista de Comunicacion y Salud. 2020;10(2):343-54.

413. Garcia-Alvarez L, de la Fuente-Tomas L, Garcia-Portilla MP, Saiz PA, Lacasa CM, Dal Santo F, et al. Early psychological impact of the 2019 coronavirus disease (COVID-19) pandemic and lockdown in a large Spanish sample. J Glob Health. 2020;10(2):020505.

414. García-González J, Ruqiong W, Alarcon-Rodriguez R, Requena-Mullor M, Ding C, Ventura-Mir, et al. Analysis of Anxiety Levels of Nursing Students Because of e-Learning during the COVID-19 Pandemic. Healthcare (Basel). 2021;9(3).

415. Garvey AM, Garcia IJ, Franco SHO, Fernandez CM. The Psychological Impact of Strict and Prolonged Confinement on Business Students during the COVID-19 Pandemic at a Spanish University. International Journal of Environmental Research and Public Health. 2021;18(4).

416. Gaş S, Ekşi Özsoy H, Cesur Aydın K. The association between sleep quality, depression, anxiety and stress levels, and temporomandibular joint disorders among Turkish dental students during the COVID-19 pandemic. Cranio. 2021:1-6.

417. Gaspar T, Paiva T, Matos MG. Impact of Covid-19 in Global Health and Psychosocial Risks at Work. J Occup Environ Med. 2021;63(7):581-7.

418. Gato J, Barrientos J, Tasker F, Miscioscia M, Cerqueira-Santos E, Malmquist A, et al. Psychosocial Effects of the COVID-19 Pandemic and Mental Health among LGBTQ plus Young Adults: A Cross-Cultural Comparison across Six Nations. J Homosexual. 2021;68(4):612-30.

419. Gautam S, Imteyaz SP, Alam MI. COVID-19 Pandemic: Assessment of Stress and Perception of E-Learning amongst First Year Undergraduate Medical Students. Journal of Clinical and Diagnostic Research. 2021;15(3):JC01-JC4.

420. Gavurova B, Ivankova V, Rigelsky M. Relationships between Perceived Stress, Depression and Alcohol Use Disorders in University Students during the COVID-19 Pandemic: A Socio-Economic Dimension. International Journal of Environmental Research and Public Health. 2020;17(23).

421. Ge FF, Zhang D, Wu LH, Mu HW. Predicting Psychological State Among Chinese Undergraduate Students in the COVID-19 Epidemic: A Longitudinal Study Using a Machine Learning. Neuropsych Dis Treat. 2020;16:2111-8.

422. Ge FF, Zheng AN, Wan MT, Luo G, Zhang J. Psychological State Among the General Chinese Population Before and During the COVID-19 Epidemic: A Network Analysis. Frontiers in Psychiatry. 2021;12.

423. Gecaite-Stonciene J, Saudargiene A, Pranckeviciene A, Liaugaudaite V, Griskova-Bulanova I, Simkute D, et al. Impulsivity Mediates Associations Between Problematic Internet Use, Anxiety, and Depressive Symptoms in Students: A Cross-Sectional COVID-19 Study. Frontiers in Psychiatry. 2021;12:634464.

424. Generali L, Iani C, Macaluso GM, Montebugnoli L, Siciliani G, Consolo U. The perceived impact of the COVID-19 pandemic on dental undergraduate students in the Italian region of Emilia-Romagna. Eur J Dent Educ. 2021;25(3):621-33.

425. George G, Thomas MR. Quarantined effects and strategies of college students – COVID-19. Asian Education and Development Studies. 2020.

426. Georgiou I, Hounat A, Park JJ, Gillespie C, Bandyopadhyay S, Saunders KEA. The Factors That Influenced Medical Students' Decision to Work Within the NHS During the COVID-19 Pandemic-A National, Cross-sectional Study. J Occup Environ Med. 2021;63(4):296-301.

427. Georgiou N, Delfabbro P, Balzan R. COVID-19-related conspiracy beliefs and their relationship with perceived stress and pre-existing conspiracy beliefs. Personality and Individual Differences. 2020;166:110201.

428. Germani A, Buratta L, Delvecchio E, Gizzi G, Mazzeschi C. Anxiety Severity, Perceived Risk of COVID-19 and Individual Functioning in Emerging Adults Facing the Pandemic. Front Psychol. 2020;11:567505.

429. Germani A, Buratta L, Delvecchio E, Mazzeschi C. Emerging Adults and COVID-19: The Role of Individualism-Collectivism on Perceived Risks and Psychological Maladjustment. Int J Environ Res Public Health. 2020;17(10).

430. Gh, our R, Ghanayem R, Alkhanafsa F, Alsharif A, Asfour H, et al. Double Burden of COVID-19 Pandemic and Military Occupation: Mental Health Among a Palestinian University Community in the West Bank. Ann Glob Health.86(1):131.

431. Ghaderi E, Mahmoodi H, Saqqezi PS, Gheshlagh RG, Moradi G, Shokri A, et al. Knowledge, attitudes, practices and fear of COVID-19 among Iranians: A quick online survey. Health Soc Care Comm. 2022;30(3):1154-62.

432. Ghafari R, Mirghafourvand M, Rouhi M, Osouli Tabrizi S. Mental health and its relationship with social support in Iranian students during the COVID-19 pandemic. BMC Psychol. 2021;9(1):81.

433. Ghazawy ER, Ewis AA, Mahfouz EM, Khalil DM, Arafa A, Mohammed Z, et al. Psychological impacts of COVID-19 pandemic on the university students in Egypt. Health Promot Int. 2021;36(4):1116-25.

434. Giardina A, Di Blasi M, Schimmenti A, King DL, Starcevic V, Billieux J. Online Gaming and Prolonged Self-Isolation: Evidence from Italian Gamers During the Covid-19 Outbreak. Clin Neuropsychiatry. 2021;18(1):65-74.

435. Giusti L, Salza A, Mammarella S, Bianco D, Ussorio D, Casacchia M, et al. #Everything Will Be Fine. Duration of Home Confinement and "All-or-Nothing" Cognitive Thinking Style as Predictors of Traumatic Distress in Young University Students on a Digital Platform During the COVID-19 Italian Lockdown. Front Psychiatry. 2020;11:574812.

436. Gloster AT, Lamnisos D, Lubenko J, Presti G, Squatrito V, Constantinou M, et al. Impact of COVID-19 pandemic on mental health: An international study. Plos One. 2020;15(12):e0244809.

437. Glowacz F, Schmits E. Psychological distress during the COVID-19 lockdown: The young adults most at risk. Psychiat Res. 2020;293:113486.

438. Göl İ, Erkin Ö. Mental status of nursing students assessed using the general health questionnaire during the COVID-19 pandemic in Turkey. Perspect Psychiatr Care. 2021.

439. Gomez-Salgado J, Andres-Villas M, Dominguez-Salas S, Diaz-Milanes D, Ruiz-Frutos C. Related Health Factors of Psychological Distress During the COVID-19 Pandemic in Spain. Int J Environ Res Public Health. 2020;17(11).

440. Gonzales G, de Mola EL, Gavulic KA, McKay T, Purcell C. Mental Health Needs Among Lesbian, Gay, Bisexual, and Transgender College Students During the COVID-19 Pandemic. J Adolescent Health. 2020;67(5):645-8.

441. Gonzalez M, Epperson AE, Halpern-Felsher B, Halliday DM, Song AV. Smokers Are More Likely to Smoke More after the COVID-19 California Lockdown Order. Int J Environ Res Public Health. 2021;18(5).

442. González-García M, Álvarez JC, Pérez EZ, Fern, ez-Carriba S, López JG. Feasibility of a Brief Online Mindfulness and Compassion-Based Intervention to Promote Mental Health Among University Students During the COVID-19 Pandemic. Mindfulness (N Y). 2021:1-11.

443. Gonzalez-Lopez OR, Buenadicha-Mateos M, Sanchez-Hernandez MI. Overwhelmed by Technostress? Sensitive Archetypes and Effects in Times of Forced Digitalization. Int J Environ Res Public Health. 2021;18(8).

444. Gonzalez-Sanguino C, Ausin B, Castellanos MA, Saiz J, Munoz M. Mental health consequences of the Covid-19 outbreak in Spain. A longitudinal study of the alarm situation and return to the new normality. Prog Neuropsychopharmacol Biol Psychiatry. 2021;107:110219.

445. Goodman JH, Luberto CM, Wang A, Halvorson B, Haramati A. Stress and Coping Among Health Professions Students During COVID-19: A Perspective on the Benefits of Mindfulness. Global Advances In Health and Medicine. 2020;9.

446. Goodwin R, Hou WK, Sun S, Ben-Ezra M. Psychological and behavioural responses to COVID-19: a China-Britain comparison. J Epidemiol Community Health. 2021;75(2):189-92.

447. Gori A, Topino E, Craparo G, Grotto RL, Caretti V. An empirical model for understanding the threat responses at the time of COVID-19. Mediterr J Clin Psyc. 2021;9(1):1-18.

448. Gori A, Topino E, Di Fabio A. The protective role of life satisfaction, coping strategies and defense mechanisms on perceived stress due to COVID-19 emergency: A chained mediation model. Plos One. 2020;15(11):e0242402.

449. Goularte JF, Serafim SD, Colombo R, Hogg B, Caldieraro MA, Rosa AR. COVID-19 and mental health in Brazil: Psychiatric symptoms in the general population. J Psychiatr Res. 2021;132:32-7.

450. Grande RAN, Berdida DJE, Villagracia HN, Cornejo LTO, Villacorte LM, Borja MVF. Association Between Perceived Resilience and Mental Well-Being of Saudi Nursing Students During COVID-19 Pandemic: A Cross-Sectional Study. Journal of Holistic Nursing. 2021;39(4):314-24.

451. Grande RAN, Butcon VER, Indonto MCL, Villacorte LM, Berdida DJE. Quality of life of nursing internship students in Saudi Arabia during the COVID-19 pandemic: A cross-sectional study. International Journal of Africa Nursing Sciences. 2021;14:100301.

452. Graupensperger S, Benson AJ, Kilmer JR, Evans MB. Social (Un)distancing: Teammate Interactions, Athletic Identity, and Mental Health of Student-Athletes During the COVID-19 Pandemic. J Adolesc Health. 2020;67(5):662-70.

453. Gritsenko V, Skugarevsky O, Konstantinov V, Khamenka N, Marinova T, Reznik A, et al. COVID 19 Fear, Stress, Anxiety, and Substance Use Among Russian and Belarusian University Students. Int J Ment Health Ad. 2021;19(6):2362-8.

454. Guan J, Wu CP, Wei DD, Xu QQ, Wang J, Lin HL, et al. Prevalence and Factors for Anxiety during the COVID-19 Pandemic among College Students in China. International Journal of Environmental Research and Public Health. 2021;18(9):4974-.

455. Guidotti Breting LM, Towns SJ, Butts AM, Brett BL, Leaffer EB, Whiteside DM. 2020 COVID-19 American Academy of Clinical Neuropsychology (AACN) Student Affairs Committee survey of neuropsychology trainees. Clin Neuropsychol. 2020;34(7-8):1284-313.

456. Guillasper JN, Oducado RMF, Soriano GP. Protective role of resilience on COVID-19 impact on the quality of life of nursing students in the Philippines. Belitung Nurs J. 2021;7(1):43-9.

457. Gul Muhammad BSSCKNMKKHBKSFAHAA. COVID-19: exploring impacts of the pandemic and lockdown on mental health of Pakistani students. Peerj. 2021.

458. Gundogan S. The mediator role of the fear of COVID-19 in the relationship between psychological resilience and life satisfaction. Curr Psychol. 2021;40(12):6291-9.

459. Guner H. Examining of the Emotional Mood about Their Online Education of First-Year Students Beginning Their University Education with Distance Education Because of COVID-19. Higher Education Studies. 2021;11(1):148-59.

460. Gungor A, Karaman MA, Sari HI, Colak TS. Investigating the Factors Related to Coronavirus Disease 2019 (COVID-19) on Undergraduate Students&apos; Interests in Coursework. International Journal of Psychology and Educational Studies. 2020;7(3):1-13.

461. Guo AA, Crum MA, Fowler LA. Assessing the Psychological Impacts of COVID-19 in Undergraduate Medical Students. Int J Environ Res Public Health. 2021;18(6).

462. Guo J, Feng XL, Wang XH, van IMH. Coping with COVID-19: Exposure to COVID-19 and Negative Impact on Livelihood Predict Elevated Mental Health Problems in Chinese Adults. Int J Environ Res Public Health. 2020;17(11):1-18.

463. Gupta P, Anupama BK, Ramakrishna K. Prevalence of Depression and Anxiety Among Medical Students and House Staff During the COVID-19 Health-Care Crisis. Acad Psychiatr. 2021;45(5):575-80.

464. Gupta R, Agrawal R. Are the concerns destroying mental health of college students?: A qualitative analysis portraying experiences amidst COVID-19 ambiguities. Anal Soc Issues Public Policy. 2021;21(1):621-39.

465. Gurvich C, Thomas N, Thomas EH, Hudaib AR, Sood L, Fabiatos K, et al. Coping styles and mental health in response to societal changes during the COVID-19 pandemic. Int J Soc Psychiatry. 2021;67(5):540-9.

466. Guse J, Heinen I, Kurre J, Mohr S, Bergelt C. Perception of the study situation and mental burden during the COVID-19 pandemic among undergraduate medical students with and without mentoring. Gms J Med Edu. 2020;37(7):Doc72.

467. Guzman-Munoz E, Concha-Cisternas Y, Onate-Barahona A, Lira-Cea C, Cigarroa-Cuevas I, Mendez-Rebolledo G, et al. [Factors associated with low quality of life in Chilean adults during the COVID-19 quarantine]. Rev Med Chil. 2020;148(12):1759-66.

468. Haesebaert F, Haesebaert J, Zante E, Franck N. Who maintains good mental health in a locked-down country? A French nationwide online survey of 11,391 participants. Health Place. 2020;66:102440.

469. Haft SL, Zhou Q. An outbreak of xenophobia: Perceived discrimination and anxiety in Chinese American college students before and during the COVID-19 pandemic. Int J Psychol. 2021;56(4):522-31.

470. Haider AS, Al-Salman S. Dataset of Jordanian university students' psychological health impacted by using e-learning tools during COVID-19. Data Brief. 2020;32:106104.

471. Hajduk M, Dancik D, Januska J, Svetsky V, Strakova A, Turcek M, et al. Psychotic experiences in student population during the COVID-19 pandemic. Schizophr Res. 2020;222:520-1.

472. Hakami Z, Khanagar SB, Vishwanathaiah S, Hakami A, Bokhari AM, Jabali AH, et al. Psychological impact of the coronavirus disease 2019 (COVID-19) pandemic on dental students: A nationwide study. J Dent Educ. 2021;85(4):494-503.

473. Haliwa I, Spalding R, Smith K, Chappell A, Strough J. Risk and protective factors for college students' psychological health during the COVID-19 pandemic. J Am Coll Health. 2021:1-5.

474. Hall SS, Zygmunt E. “I Hate It Here”: Mental Health Changes of College Students Living With Parents During the COVID-19 Quarantine. Emerging Adulthood. 2021:21676968211000494-.

475. Hall SS, Zygmunt E. Dislocated College Students and the Pandemic: Back Home Under Extraordinary Circumstances. Fam Relat. 2021;70(3):689-704.

476. Halperin SJ, Henderson MN, Prenner S, Grauer JN. Prevalence of Anxiety and Depression Among Medical Students During the Covid-19 Pandemic: A Cross-Sectional Study. J Med Educ Curric De. 2021;8:1-7.

477. Hamad SA, Abdallah YN, Eman Zmaily D, Mohammed HA, Musfer SA. Depression and anxiety during 2019 coronavirus disease pandemic in Saudi Arabia: a cross-sectional study. 2020.

478. Hamaideh SH, Al-Modallal H, Tanash M, Hamdan-Mansour A. Depression, anxiety and stress among undergraduate students during COVID-19 outbreak and "home-quarantine". Nurs Open. 2022;9(2):1423-31.

479. Hammad MA, Alqarni TM. Psychosocial effects of social media on the Saudi society during the Coronavirus Disease 2019 pandemic: A cross-sectional study. Plos One. 2021;16(3):e0248811.

480. Hamza CA, Ewing L, Heath NL, Goldstein AL. When social isolation is nothing new: A longitudinal study on psychological distress during COVID-19 among university students with and without preexisting mental health concerns. Canadian Psychology/Psychologie canadienne. 2021;62(1):20-30.

481. Han T, Ma W, Gong H, Hu Y, Zhang Y, Zhang C, et al. Investigation and analysis of negative emotion among university students during home quarantine of COVID-19. Journal of Xi'an Jiaotong University (Medical Sciences). 2021;42(1):132-6.

482. Han W, Xu L, Niu A, Jing Y, Qin W, Zhang J, et al. Online-Based Survey on College Students' Anxiety During COVID-19 Outbreak. Psychol Res Behav Manag. 2021;14:385-92.

483. Hao F, Tan W, Jiang L, Zhang L, Zhao X, Zou Y, et al. Do psychiatric patients experience more psychiatric symptoms during COVID-19 pandemic and lockdown? A case-control study with service and research implications for immunopsychiatry. Brain, Behavior, and Immunity. 2020;87:100-6.

484. Harries AJ, Lee C, Jones L, Rodriguez RM, Davis JA, Boysen-Osborn M, et al. Effects of the COVID-19 pandemic on medical students: a multicenter quantitative study. BMC Med Educ. 2021;21(1):14.

485. Harris SM, Sandal GM. COVID-19 and psychological distress in Norway: The role of trust in the healthcare system. Scand J Public Health. 2021;49(1):96-103.

486. Hassnain S, Omar N. How COVID-19 is Affecting Apprentices. Biomedica. 2020;36:251-5.

487. Hathaway ED, Peyer KL, Doyle KA. A first look at perceived stress in southeastern university students during the COVID-19 pandemic. J Am Coll Health. 2021:1-4.

488. Hay D, Jamal MS, Al-Tawil K, Petohazi A, Gulli V, Bednarczuk NF, et al. The effect of the COVID-19 pandemic on mental health associated trauma, admissions and fractures at a London major trauma centre. Ann R Coll Surg Engl. 2021;103(2):114-9.

489. Hayat K, Haq M, Wang WH, Khan FU, Rehman AU, Rasool M, et al. Impact of the COVID-19 outbreak on mental health status and associated factors among general population: a cross-sectional study from Pakistan. Psychol Health Med. 2022;27(1):54-68.

490. He L, Wei D, Yang F, Zhang J, Cheng W, Feng J, et al. Functional Connectome Prediction of Anxiety Related to the COVID-19 Pandemic. Am J Psychiatry. 2021;178(6):530-40.

491. He Q, Fan BF, Xie B, Liao YH, Han X, Chen Y, et al. Mental health conditions among the general population, healthcare workers and quarantined population during the coronavirus disease 2019 (COVID-19) pandemic. Psychol Health Med. 2022;27(1):186-98.

492. Helena Silveira S, Mariana Gonzalez C, Valesca Doro D, Mateus Luz L, owski, Tiago Neunenfeld M, et al. Depression and anxiety among the University community during the Covid-19 pandemic: a study in Southern Brazil. 2021.

493. Herle M, Smith AD, Bu F, Steptoe A, Fancourt D. Trajectories of eating behavior during COVID-19 lockdown: Longitudinal analyses of 22,374 adults. Clin Nutr ESPEN. 2021;42:158-65.

494. Hernández-López M, Cepeda-Benito A, Díaz-Pavón P, Rodríguez-Valverde M. Psychological inflexibility and mental health symptoms during the COVID-19 lockdown in Spain: A longitudinal study. Journal of Contextual Behavioral Science. 2021;19:42-9.

495. Hidalgo MD, Balluerka N, Gorostiaga A, Espada JP, Santed MA, Padilla JL, et al. The Psychological Consequences of COVID-19 and Lockdown in the Spanish Population: An Exploratory Sequential Design. Int J Environ Res Public Health. 2020;17(22).

496. Hoffart A, Johnson SU, Ebrahimi OV. Loneliness and Social Distancing During the COVID-19 Pandemic: Risk Factors and Associations With Psychopathology. Front Psychiatry. 2020;11:589127.

497. Hong W, Liu RD, Ding Y, Fu X, Zhen R, Sheng X. Social Media Exposure and College Students' Mental Health During the Outbreak of COVID-19: The Mediating Role of Rumination and the Moderating Role of Mindfulness. Cyberpsychol Behav Soc Netw. 2021;24(4):282-7.

498. Hood B, Jelbert S, Santos LR. Benefits of a psychoeducational happiness course on university student mental well-being both before and during a COVID-19 lockdown. Health Psychol Open. 2021;8(1):2055102921999291.

499. Hoorelbeke K, Sun X, Koster EHW, Dai Q. Connecting the dots: A network approach to post-traumatic stress symptoms in Chinese healthcare workers during the peak of the Coronavirus Disease 2019 outbreak. Stress Health. 2021;37(4):692-705.

500. Horita R, Nishio A, Yamamoto M. The effect of remote learning on the mental health of first year university students in Japan. Psychiatry Res. 2021;295:113561.

501. Hossain MJ, Hridoy A, Rahman SMA, Ahmmed F. Major Depressive and Generalized Anxiety Disorders Among University Students During the Second Wave of COVID-19 Outbreak in Bangladesh. Asia-Pac J Public He. 2021;33(5):676-8.

502. Hossain MT, Ahammed B, Chanda SK, Jahan N, Ela MZ, Islam MN. Social and electronic media exposure and generalized anxiety disorder among people during COVID-19 outbreak in Bangladesh: A preliminary observation. Plos One. 2020;15(9).

503. Hossain SFA, Nurunnabi M, Sundarasen S, Chinna K, Kamaludin K, Baloch GM, et al. Socio-psychological impact on Bangladeshi students during COVID-19. Journal of Public Health Research. 2020;9:38-44.

504. Hou FS, Bi FY, Jiao R, Luo D, Song KX. Gender differences of depression and anxiety among social media users during the COVID-19 outbreak in China:a cross-sectional study. Bmc Public Health. 2020;20(1):1648.

505. Hou J, Yu Q, Lan X. COVID-19 Infection Risk and Depressive Symptoms Among Young Adults During Quarantine: The Moderating Role of Grit and Social Support. Front Psychol. 2020;11:577942.

506. Hou WK, Lee TM, Liang L, Li TW, Liu H, Tong H, et al. Psychiatric symptoms and behavioral adjustment during the COVID-19 pandemic: evidence from two population-representative cohorts. Transl Psychiatry. 2021;11(1):174.

507. Hou WK, Tong H, Liang L, Li TW, Liu H, Ben-Ezra M, et al. Probable anxiety and components of psychological resilience amid COVID-19: A population-based study. J Affect Disord. 2021;282:594-601.

508. Hoyt LT, Cohen AK, Dull B, Maker Castro E, Yazdani N. "Constant Stress Has Become the New Normal": Stress and Anxiety Inequalities Among U.S. College Students in the Time of COVID-19. J Adolesc Health. 2021;68(2):270-6.

509. Hsu CH, Lin HH, Wang CC, Jhang S. How to defend covid-19 in taiwan? Talk about people’s disease awareness, attitudes, behaviors and the impact of physical and mental health. International Journal of Environmental Research and Public Health. 2020;17(13):1-23.

510. Hu Q, Umeda M. Stress, Anxiety, and Depression for Chinese Residents in Japan during the COVID-19 Pandemic. Int J Environ Res Public Health. 2021;18(9).

511. Huang L, Kern ML, Oades LG. Strengthening University Student Wellbeing: Language and Perceptions of Chinese International Students. Int J Environ Res Public Health. 2020;17(15):1-18.

512. Huang L, Lei W, Xu F, Liu H, Yu L. Emotional responses and coping strategies in nurses and nursing students during Covid-19 outbreak: A comparative study. Plos One. 2020;15(8):e0237303.

513. Huang Y, Zhao N. Generalized anxiety disorder, depressive symptoms and sleep quality during COVID-19 outbreak in China: a web-based cross-sectional survey. Psychiatry Res. 2020;288:112954.

514. Huang Y, Zhao N. Chinese mental health burden during the COVID-19 pandemic. Asian Journal of Psychiatry. 2020;51.

515. Huang Y, Zhao N. Mental health burden for the public affected by the COVID-19 outbreak in China: Who will be the high-risk group? Psychology, Health and Medicine. 2021;26(1):23-34.

516. Huckins JF, daSilva AW, Wang W, Hedlund E, Rogers C, Nepal SK, et al. Mental Health and Behavior of College Students During the Early Phases of the COVID-19 Pandemic: Longitudinal Smartphone and Ecological Momentary Assessment Study. J Med Internet Res. 2020;22(6):e20185.

517. Huma, Sohail MK, Akhtar N, Muhammad D, Afzal H, Mufti MR, et al. Analyzing COVID-2019 Impact on Mental Health Through Social Media Forum. Cmc-Comput Mater Con. 2021;67(3):3737-48.

518. Hung M, Licari FW, Hon ES, Lauren E, Su S, Birmingham WC, et al. In an era of uncertainty: Impact of COVID-19 on dental education. J Dent Educ. 2021;85(2):148-56.

519. Hunt C, Gibson GC, Vander Horst A, Cleveland KA, Wawrosch C, Granot M, et al. Gender Diverse College Students Exhibit Higher Psychological Distress Than Male and Female Peers During the Novel Coronavirus (COVID-19) Pandemic. Psychol Sex Orientat. 2021;8(2):238-44.

520. Husain W, Ashkanani F. Does COVID-19 change dietary habits and lifestyle behaviours in Kuwait: a community-based cross-sectional study. Environ Health Prev. 2020;25(1).

521. Husky MM, Kovess-Masfety V, Gobin-Bourdet C, Swendsen J. Prior depression predicts greater stress during Covid-19 mandatory lockdown among college students in France. Compr Psychiat. 2021;107:152234.

522. Husky MM, Kovess-Masfety V, Swendsen JD. Stress and anxiety among university students in France during Covid-19 mandatory confinement. Compr Psychiat. 2020;102:152191.

523. Hussien RM, Shahin MAH. Coronavirus disease-19 quarantine experience in the middle east region: Emotional status, health patterns, and self-efficacy survey. Open Access Macedonian Journal of Medical Sciences. 2020;8:330-45.

524. Idoiaga Mondragon N, Berasategi Sancho N, Eiguren Munitis A, Dosil Santamaria M. Exploring the social and emotional representations used by students from the University of the Basque Country to face the first outbreak of COVID-19 pandemic. Health Educ Res. 2021;36(2):159-69.

525. Idowu A, Olawuyi DA, Nwadioke CO. Impacts of Covid-19 Pandemic on the Psychological Well Being of Students in a Nigerian University. J Med Surg Res. 2020;7(1):798-806.

526. Ihm L, Zhang H, van Vijfeijken A, Waugh MG. Impacts of the Covid-19 pandemic on the health of university students. Int J Health Plann Manage. 2021;36(3):618-27.

527. Ikizer G, Karanci AN, Gul E, Dilekler I. Post-traumatic stress, growth, and depreciation during the COVID-19 pandemic: evidence from Turkey. Eur J Psychotraumatol. 2021;12(1):1872966.

528. Ilango S, Jayakumar S, Kumar KS, Kumar Madhan S, Ardhanaari M, Gunapriya R. Impact of COVID-19 outbreak on the mental health status of undergraduate medical students in a COVID-19 treating medical college: a prospective longitudinal study. Peerj. 2020.

529. Imran N, Masood HMU, Ayub M, Gondal KM. Psychological impact of COVID-19 pandemic on postgraduate trainees: a cross-sectional survey. Postgrad Med J. 2021;97(1152):632-7.

530. Imtiaz A, Khan NM, Hossain MA. COVID-19 in Bangladesh: measuring differences in individual precautionary behaviors among young adults. J Public Health-Heid. 2022;30(6):1473-84.

531. Inbar L, Shinan-Altman S. Emotional reactions and subjective health status during the COVID-19 pandemic in Israel: the mediating role of perceived susceptibility. Psychol Health Med. 2021;26(1):75-84.

532. Iob E, Frank P, Steptoe A, Fancourt D. Levels of Severity of Depressive Symptoms Among At-Risk Groups in the UK During the COVID-19 Pandemic. Jama Netw Open. 2020;3(10):e2026064.

533. Islam MA, Barna SD, Raihan H, Khan MNA, Hossain MT. Depression and anxiety among university students during the COVID-19 pandemic in Bangladesh: A web-based cross-sectional survey. Plos One. 2020;15(8):e0238162.

534. Islam MS, Ferdous MZ, Potenza MN. Panic and generalized anxiety during the COVID-19 pandemic among Bangladeshi people: An online pilot survey early in the outbreak. Journal of Affective Disorders. 2020;276:30-7.

535. Islam MS, Potenza MN, van Os J. Posttraumatic stress disorder during the COVID-19 pandemic: Upcoming challenges in Bangladesh and preventive strategies. Int J Soc Psychiatry. 2021;67(2):205-6.

536. Islam MS, Sujan MSH, Tasnim R, Ferdous MZ, Masud JHB, Kundu S, et al. Problematic internet use among young and adult population in Bangladesh: Correlates with lifestyle and online activities during the COVID-19 pandemic. Addict Behav Rep. 2020;12:100311.

537. Islam MS, Sujan MSH, Tasnim R, Mohona RA, Ferdous MZ, Kamruzzaman S, et al. Problematic Smartphone and Social Media Use Among Bangladeshi College and University Students Amid COVID-19: The Role of Psychological Well-Being and Pandemic Related Factors. Frontiers in Psychiatry. 2021;12:647386.

538. Islam MS, Sujan MSH, Tasnim R, Sikder MT, Potenza MN, van Os J. Psychological responses during the COVID-19 outbreak among university students in Bangladesh. Plos One. 2020;15(12):e0245083.

539. Islam SMD, Bodrud-Doza M, Khan RM, Haque MA, Mamun MA. Exploring COVID-19 stress and its factors in Bangladesh: A perception-based study. Heliyon. 2020;6(7):e04399.

540. Jackson KM, Merrill JE, Stevens AK, Hayes KL, White HR. Changes in Alcohol Use and Drinking Context due to the COVID-19 Pandemic: A Multimethod Study of College Student Drinkers. Alcohol Clin Exp Res. 2021;45(4):752-64.

541. Jacob L, Smith L, Armstrong NC, Yakkundi A, Barnett Y, Butler L, et al. Alcohol use and mental health during COVID-19 lockdown: A cross-sectional study in a sample of UK adults. Drug and Alcohol Dependence. 2021;219.

542. Jacobs R, Lanspa M, Kane M, Caballero J. Predictors of emotional wellbeing in osteopathic medical students in a COVID-19 world. J Osteopath Med. 2021;121(5):455-61.

543. Jahan I, Ullah I, Griffiths MD, Mamun MA. Covid‐19 suicide and its causative factors among the healthcare professionals: Case study evidence from press reports. Perspectives in Psychiatric Care. 2021.

544. Jane-Llopis E, Anderson P, Segura L, Zabaleta E, Munoz R, Ruiz G, et al. Mental ill-health during COVID-19 confinement. Bmc Psychiatry. 2021;21(1):194.

545. Jarego M, Pimenta F, Pais-Ribeiro J, Costa RM, Patrão I, Coelho L, et al. Do coping responses predict better/poorer mental health in Portuguese adults during Portugal's national lockdown associated with the COVID-19? Personality and Individual Differences. 2021;175.

546. Jarid G, Sharron Xuanren W, Rubi AGO, Hern M, ez S. Mental health of undocumented college students during the COVID-19 pandemic. 2020.

547. Jassim G, Jameel M, Brennan E, Yusuf M, Hasan N, Alwatani Y. Psychological Impact of COVID-19, Isolation, and Quarantine: A Cross-Sectional Study. Neuropsych Dis Treat. 2021;17:1413-21.

548. Jeong HJ, Kim S, Lee J. Mental Health, Life Satisfaction, Supportive Parent Communication, and Help-seeking Sources in the wake of COVID-19: First-generation College Students (FGCS) Vs. Non-first-generation College Students (non-FGCS). Journal of College Student Psychotherapy. 2021.

549. Jezzini-Martinez S, Quiroga-Garza A, Jacobo-Baca G, Guzman-Lopez S, Salinas-Alvarez Y, Martinez-Garza J, et al. COVID-19 Causing Burnout Among Medical Students. Faseb J. 2021;35.

550. Ji GJ, Wei WJ, Yue KC, Li H, Shi LJ, Ma JD, et al. Effects of the COVID-19 Pandemic on Obsessive-Compulsive Symptoms Among University Students: Prospective Cohort Survey Study. J Med Internet Res. 2020;22(9):e21915.

551. Jia Y, Qi Y, Bai L, Han Y, Xie Z, Ge J. Knowledge-attitude-practice and psychological status of college students during the early stage of COVID-19 outbreak in China: a cross-sectional study. Bmj Open. 2021;11(2):e045034.

552. Jiang D. Perceived Stress and Daily Well-Being During the COVID-19 Outbreak: The Moderating Role of Age. Front Psychol. 2020;11:571873.

553. Jiang HJ, Nan J, Lv ZY, Yang J. Psychological impacts of the COVID-19 epidemic on Chinese people: Exposure, post-traumatic stress symptom, and emotion regulation. Asian Pac J Trop Med. 2020;13(6):252-9.

554. Jiang R. Knowledge, attitudes and mental health of university students during the COVID-19 pandemic in China. Child Youth Serv Rev. 2020;119:105494.

555. Jiang R-cLA-m. Mental health status and its influencing factors of college students in Anhui Province during the coronavirus disease 2019 pandemic. Huanjing yu Zhiye Yixue = Journal of Environmental &amp; Occupational Medicine. 2020;37(9):867-.

556. Jiang W, Liu X, Zhang J, Feng Z. Mental health status of Chinese residents during the COVID-19 epidemic. Bmc Psychiatry. 2020;20(1):580.

557. Jiang W, Ren Z, Yu L, Tan Y, Shi C. A Network Analysis of Post-traumatic Stress Disorder Symptoms and Correlates During the COVID-19 Pandemic. Front Psychiatry. 2020;11:568037.

558. Jiang Y. Problematic Social Media Usage and Anxiety Among University Students During the COVID-19 Pandemic: The Mediating Role of Psychological Capital and the Moderating Role of Academic Burnout. Frontiers in Psychology. 2021;12:612007.

559. Jimenez O, Sanchez-Sanchez LC, Garcia-Montes JM. Psychological Impact of COVID-19 Confinement and Its Relationship with Meditation. Int J Environ Res Public Health. 2020;17(18).

560. Jin LY, Hao ZJ, Huang JZ, Akram HR, Saeed MF, Ma HB. Depression and anxiety symptoms are associated with problematic smartphone use under the COVID-19 epidemic: The mediation models. Child Youth Serv Rev. 2021;121.

561. Jin Z, Zhao KB, Xia YY, Chen RJ, Yu H, Tamutana TT, et al. Relationship Between Psychological Responses and the Appraisal of Risk Communication During the Early Phase of the COVID-19 Pandemic: A Two-Wave Study of Community Residents in China. Front Public Health. 2020;8:550220.

562. Jindal V, Mittal S, Kaur T, Bansal AS, Kaur P, Kaur G, et al. Knowledge, anxiety and the use of hydroxychloroquine prophylaxis among health care students and professionals regarding COVID-19 pandemic. Adv Respir Med. 2020;88(6):520-30.

563. Jing X, Yu J, Yu Z, Xinyi G, Wenjing M, Bo Z, et al. The Impact of Psychology Interventions on Changing Mental Health Status and Sleep Quality in University Students during the COVID-19 Pandemic. 2020.

564. Johansson F, Cote P, Hogg-Johnson S, Rudman A, Holm LW, Grotle M, et al. Depression, anxiety and stress among Swedish university students before and during six months of the COVID-19 pandemic: A cohort study. Scand J Public Healt. 2021;49(7):741-9.

565. John RR, R PJ. Impact of Lockdown on the Attitude of University Students in South India-A Cross-Sectional Observational Study. J Maxillofac Oral Surg. 2021:1-8.

566. Jones HE, Manze M, Ngo V, Lamberson P, Freudenberg N. The Impact of the COVID-19 Pandemic on College Students' Health and Financial Stability in New York City: Findings from a Population-Based Sample of City University of New York (CUNY) Students. J Urban Health. 2021;98(2):187-96.

567. Joseph R, Lucca JM, Alshayban D, Alshehry YA. The immediate psychological response of the general population in Saudi Arabia during COVID-19 pandemic: A cross-sectional study. J Infect Public Health. 2021;14(2):276-83.

568. Joshi A, Kaushik V, Vats N, Kour S. Covid-19 pandemic: Pathological, socioeconomical and psychological impact on life, and possibilities of treatment. International Journal of Pharmaceutical Research. 2021;13(2):2724-38.

569. Juchnowicz D, Baj J, Forma A, Karakula K, Sitarz R, Bogucki J, et al. The Outbreak of SARS-CoV-2 Pandemic and the Well-Being of Polish Students: The Risk Factors of the Emotional Distress during COVID-19 Lockdown. J Clin Med. 2021;10(5):1-22.

570. Julie A, aux, Ilaria M, Melissa M, Nathalie T, Mathilde P, et al. Higher risk of mental health deterioration during the Covid-19 lockdown among students rather than non-students. The French Confins study. 2020.

571. Jungmann SM, Witthöft M. Health anxiety, cyberchondria, and coping in the current COVID-19 pandemic: Which factors are related to coronavirus anxiety? Journal of Anxiety Disorders. 2020;73.

572. Kadam P, Jabade M, Ligade T. A study to assess the student's anxiety level about examination during lock down in selected colleges of pune city. Indian Journal of Forensic Medicine and Toxicology. 2020;14(4):3723-5.

573. Kaisar MT, Chowdhury SY. Foreign Language Virtual Class Room: Anxiety Creator or Healer? English Language Teaching. 2020;13(11):130-9.

574. Kalkan Uğurlu Y, Mataracı Değirmenci D, Durgun H, Gök Uğur H. The examination of the relationship between nursing students' depression, anxiety and stress levels and restrictive, emotional, and external eating behaviors in COVID-19 social isolation process. Perspect Psychiatr Care. 2021;57(2):507-16.

575. Kalok A, Sharip S, Abdul Hafizz AM, Zainuddin ZM, Shafiee MN. The Psychological Impact of Movement Restriction during the COVID-19 Outbreak on Clinical Undergraduates: A Cross-Sectional Study. Int J Environ Res Public Health. 2020;17(22).

576. Kamaludin K, Chinna K, Sundarasen S, Khoshaim HB, Nurunnabi M, Baloch GM, et al. Coping with COVID-19 and movement control order (MCO): experiences of university students in Malaysia. Heliyon. 2020;6(11):e05339.

577. Kandel S, Lamsal M, Yadav SA, Bhandari D, Adhikari G, Poudel S, et al. Lifestyle, behavior, perception and practices of Nepalese during lockdown due to COVID-19 pandemic. JNMA J Nepal Med Assoc. 2020;58(229):690-5.

578. Kantor BN, Kantor J. Mental Health Outcomes and Associations During the COVID-19 Pandemic: A Cross-Sectional Population-Based Study in the United States. Front Psychiatry. 2020;11:569083.

579. Kaparounaki CK, Patsali ME, Mousa DV, Papadopoulou EVK, Papadopoulou KKK, Fountoulakis KN. University students' mental health amidst the COVID-19 quarantine in Greece. Psychiatry Res. 2020;290:113111.

580. Kapasia N, Paul P, Roy A, Saha J, Zaveri A, Mallick R, et al. Impact of lockdown on learning status of undergraduate and postgraduate students during COVID-19 pandemic in West Bengal, India. Child Youth Serv Rev. 2020;116:105194.

581. Kar N, Kar B, Kar S. Stress and coping during COVID-19 pandemic: Result of an online survey. Psychiatry Res. 2021;295:113598.

582. Karageorghis CI, Bird JM, Hutchinson JC, Hamer M, Delevoye-Turrell YN, Guerin SMR, et al. Physical activity and mental well-being under COVID-19 lockdown: a cross-sectional multination study. Bmc Public Health. 2021;21(1):988.

583. Karahan Yilmaz S, Eskici G. Evaluation of emotional (depression) and behavioural (nutritional, physical activity and sleep) status of Turkish adults during the COVID-19 pandemic period. Public Health Nutr. 2021;24(5):942-9.

584. Karasar B, Canli D. Psychological Resilience and Depression during the Covid-19 Pandemic in Turkey. Psychiatr Danub. 2020;32(2):273-9.

585. Karasmanaki E, Tsantopoulos G. Impacts of social distancing during COVID-19 pandemic on the daily life of forestry students. Child Youth Serv Rev. 2021;120:105781.

586. Kassim MAM, Pang NTP, Mohamed NH, Kamu A, Ho CM, Ayu F, et al. Relationship Between Fear of COVID-19, Psychopathology and Sociodemographic Variables in Malaysian Population. Int J Ment Health Ad. 2022;20(3):1303-10.

587. Kassir G, El Hayek S, Zalzale H, Orsolini L, Bizri M. Psychological distress experienced by self-quarantined undergraduate university students in Lebanon during the COVID-19 outbreak. Int J Psychiatry Clin Pract. 2021;25(2):172-9.

588. Kate Mc I, Pauline L, Patrick D, Henry W, Judith MV, Anil PSO, et al. The Lifelines COVID-19 Cohort: a questionnaire-based study to investigate COVID-19 infection and its health and societal impacts in a Dutch population-based cohort. 2020.

589. Kaufman-Shriqui V, Navarro DA, Raz O, Boaz M. Multinational dietary changes and anxiety during the coronavirus pandemic-findings from Israel. Isr J Health Policy Res. 2021;10(1):28.

590. Kavaklı M, Ak M, Uğuz F, Türkmen OO. The mediating role of self-compassion in the relationship between perceived COVID-19 threat and death anxiety. Klinik Psikiyatri Dergisi. 2020;23:15-23.

591. Kecojevic A, Basch CH, Sullivan M, Davi NK. The impact of the COVID-19 epidemic on mental health of undergraduate students in New Jersey, cross-sectional study. Plos One. 2020;15(9):e0239696.

592. Kehok SILMRIK. Exploring the Emotions of Single International Students in Hong Kong Facing the COVID-19 Pandemic. Journal of International Students. 2020;10:91-107.

593. Keyserlingk L, Yamaguchi‐Pedroza K, Arum R, Eccles JS. Stress of university students before and after campus closure in response to covid‐19. Journal of Community Psychology. 2021.

594. Keyworth C, Epton T, Byrne-Davis L, Leather JZ, Armitage CJ. What challenges do UK adults face when adhering to COVID-19-related instructions? Cross-sectional survey in a representative sample. Prev Med. 2021;147:106458.

595. Khademian F, Delavari S, Koohjani Z, Khademian Z. An investigation of depression, anxiety, and stress and its relating factors during COVID-19 pandemic in Iran. Bmc Public Health. 2021;21(1):275.

596. Khalid A, Younas MW, Khan H, Khan MS, Malik AR, Butt AUA, et al. Relationship between knowledge on COVID-19 and psychological distress among students living in quarantine: an email survey. Aims Public Health. 2021;8(1):90-9.

597. Khan AH, Sultana MS, Hossain S, Hasan MT, Ahmed HU, Sikder MT. The impact of COVID-19 pandemic on mental health & wellbeing among home-quarantined Bangladeshi students: A cross-sectional pilot study. Journal of Affective Disorders. 2020;277:121-8.

598. Kharma MY, Koussa B, Aldwaik A, Yaseen J, Alamari S, Alras H, et al. Assessment of Anxiety and Stress among Dental Students to Return to Training in Dental College in COVID-19 Era. Eur J Dent. 2020;14:S86-s90.

599. Khatatbeh M, Khasawneh A, Hussein H, Altahat O, Alhalaiqa F. Psychological Impact of COVID-19 Pandemic Among the General Population in Jordan. Frontiers in Psychiatry. 2021;12.

600. Khattar A, Jain PR, Quadri SMK. Effects of the Disastrous Pandemic COVID 19 on Learning Styles, Activities and Mental Health of Young Indian Students - A Machine Learning Approach. Proceedings of the International Conference on Intelligent Computing and Control Systems (Iciccs 2020). 2020:1190-5.

601. Kheirallah K, Bloukh S, Khasawneh W, Alsulaiman J, Khassawneh A, Al-Mistarehi AH, et al. Medical students' relative immunity, or lack thereof, against COVID-19 emotional distress and psychological challenges; a descriptive study from Jordan. F1000Res. 2021;10:297.

602. Khodabakhshi-Koolaee A. Living in home quarantine: Analyzing psychological experiences college Student's in COVID-19. Journal of Military Medicine. 2020;22(2):130-8.

603. Khoshaim HB, Al-Sukayt A, Chinna K, Nurunnabi M, Sundarasen S, Kamaludin K, et al. Anxiety Level of University Students During COVID-19 in Saudi Arabia. Front Psychiatry. 2020;11:579750.

604. Khoshaim HB, Al-Sukayt A, Nurunnabi M, Sundarasen S, Kamaludin K, Chinna K, et al. How students in the Kingdom of Saudi Arabia are coping with COVID-pandemic 19. Journal of Public Health Research. 2020;9:17-23.

605. Khurram R, Razzak DA, Ahmad W, Qamar A, Mumtaz T, Sameed QU. Mental Distress after 1st Wave of COVID-19 among Medical Students. Journal of Pharmaceutical Research International. 2020;32(44):7-12.

606. Kibbey MM, Fedorenko EJ, Farris SG. Anxiety, depression, and health anxiety in undergraduate students living in initial US outbreak "hotspot" during COVID-19 pandemic. Cogn Behav Therapy. 2021;50(5):409-21.

607. Kilani HA, Bataineh MF, Al-Nawayseh A, Atiyat K, Obeid O, Abu-Hilal MM, et al. Healthy lifestyle behaviors are major predictors of mental wellbeing during COVID-19 pandemic confinement: A study on adult Arabs in higher educational institutions. Plos One. 2020;15(12):e0243524.

608. Kilius E, Abbas NH, McKinnon L, Samson DR. Pandemic Nightmares: COVID-19 Lockdown Associated With Increased Aggression in Female University Students' Dreams. Front Psychol. 2021;12:644636.

609. Kim JH, Shim Y, Choi I, Choi E. The Role of Coping Strategies in Maintaining Well-Being During the COVID-19 Outbreak in South Korea. Soc Psychol Pers Sci. 2022;13(1):320-32.

610. Kim SC, Sloan C, Montejano A, Quiban C. Impacts of Coping Mechanisms on Nursing Students' Mental Health during COVID-19 Lockdown: A Cross-Sectional Survey. Nurs Rep. 2021;11(1):36-44.

611. Kim YJ. The Effect of Tele-acupressure Self-practice for Mental Health and Wellbeing in the Community During COVID-19. Curr Psychiat Res Re. 2020;16(4):267-74.

612. Kim YJ, Cho JH. Correlation between Preventive Health Behaviors and Psycho-Social Health Based on the Leisure Activities of South Koreans in the COVID-19 Crisis. International Journal of Environmental Research and Public Health. 2020;17(11).

613. Kim YJ, Kim ES. Relationship between Phobic Anxiety in Work and Leisure Activity Situations, and Optimistic Bias Associated with COVID-19 among South Koreans. International Journal of Environmental Research and Public Health. 2020;17(22).

614. Kira IA, Shuwiekh HAM, Alhuwailah A, Ashby JS, Sous MSF, Baali SBA, et al. The Effects of COVID-19 and Collective Identity Trauma (Intersectional Discrimination) on Social Status and Well-Being. Traumatology. 2021;27(1):29-39.

615. Kiran Fatima BSS. IMPACT ON MENTAL HEALTH OF UNDERGRADUATES AND THE WAYS TO COPE STRESS DURING COVID-19 PANDEMIC. Pakistan Armed Forces Medical Journal. 2020(5):1453-.

616. Kirmizi M, Yalcinkaya G, Sengul YS. Gender differences in health anxiety and musculoskeletal symptoms during the COVID-19 pandemic. J Back Musculoskelet Rehabil. 2021;34(2):161-7.

617. Klaassen H, Ashida S, Comnick CL, Xie XJ, Smith BM, Tabrizi M, et al. COVID-19 pandemic and its impact on dental students: A multi-institutional survey. J Dent Educ. 2021;85(7):1280-6.

618. Kleiman EM, Yeager AL, Grove JL, Kellerman JK, Kim JS. Real-time Mental Health Impact of the COVID-19 Pandemic on College Students: Ecological Momentary Assessment Study. JMIR Ment Health. 2020;7(12):e24815.

619. Knowles KA, Olatunji BO. Anxiety and safety behavior usage during the COVID-19 pandemic: The prospective role of contamination fear. J Anxiety Disord. 2021;77:102323.

620. Kocak O, Kocak OE, Younis MZ. The Psychological Consequences of COVID-19 Fear and the Moderator Effects of Individuals' Underlying Illness and Witnessing Infected Friends and Family. Int J Environ Res Public Health. 2021;18(4):1-15.

621. Kochhar AS, Bhasin R, Kochhar GK, Dadlani H, Mehta VV, Kaur R, et al. Lockdown of 1.3 billion people in India during Covid-19 pandemic: A survey of its impact on mental health. Asian J Psychiatr. 2020;54:102213.

622. Kochuvilayil T, Fernandez RS, Moxham LJ, Lord H, Alomari A, Hunt L, et al. COVID-19: Knowledge, anxiety, academic concerns and preventative behaviours among Australian and Indian undergraduate nursing students: A cross-sectional study. J Clin Nurs. 2021;30(5-6):882-91.

623. Kohls E, Baldofski S, Moeller R, Klemm SL, Rummel-Kluge C. Mental Health, Social and Emotional Well-Being, and Perceived Burdens of University Students During COVID-19 Pandemic Lockdown in Germany. Front Psychiatry. 2021;12:643957.

624. Kokturk Dalcali B, Durgun H, Tas AS. Anxiety levels and sleep quality in nursing students during the COVID-19 pandemic. Perspect Psychiatr Care. 2021;57(4):1999-2005.

625. Kolodziejczyk A, Misiak B, Szczesniak D, Maciaszek J, Ciulkowicz M, Luc D, et al. Coping Styles, Mental Health, and the COVID-19 Quarantine: A Nationwide Survey in Poland. Front Psychiatry. 2021;12:625355.

626. Kornilaki EN. The psychological effect of COVID-19 quarantine on Greek young adults: Risk factors and the protective role of daily routine and altruism. Int J Psychol. 2022;57(1):33-42.

627. Kostic J, Zikic O, Dordevic V, Krivokapic Z. Perceived stress among university students in south-east Serbia during the COVID-19 outbreak. Ann Gen Psychiatr. 2021;20(1):25.

628. Kotera Y, Cockerill V, Chircop JGE, Forman D. Mental health shame, self-compassion and sleep in UK nursing students: Complete mediation of self-compassion in sleep and mental health. Nurs Open. 2021;8(3):1325-35.

629. Kowal M, Coll-Martín T, Ikizer G, Rasmussen J, Eichel K, Studzińska A, et al. Who is the Most Stressed During the COVID-19 Pandemic? Data From 26 Countries and Areas. Appl Psychol Health Well Being. 2020;12(4):946-66.

630. Krendl AC. Changes in stress predict worse mental health outcomes for college students than does loneliness; evidence from the COVID-19 pandemic. J Am Coll Health. 2021:1-4.

631. Kshipra M, Disha K, Manjusha P. COVID-19 and Mental Health: A Study of its Impact on Students. 2020.

632. Kuman Tuncel O, Tasbakan SE, Gokengin D, Erdem HA, Yamazhan T, Sipahi OR, et al. The deep impact of the COVID-19 pandemic on medical students: An online cross-sectional study evaluating Turkish students' anxiety. Int J Clin Pract. 2021;75(6):e14139.

633. Kumari P, Gupta P, Piyoosh AK, Tyagi B, Kumar P. COVID 19 : Impact on mental health of graduating and post graduating students. J Stat Manag Syst. 2021;24(1):67-79.

634. Kurcer MA, Erdogan Z, Cakir Kardes V. The effect of the COVID-19 pandemic on health anxiety and cyberchondria levels of university students. Perspect Psychiatr Care. 2022;58(1):132-40.

635. Kuru Alici N, Ozturk Copur E. Anxiety and fear of covid‐19 among nursing students during the covid‐19 pandemic: A descriptive correlation study. Perspectives in Psychiatric Care. 2021.

636. Labrague LJ, Ballad CA. Lockdown fatigue among college students during the COVID-19 pandemic: Predictive role of personal resilience, coping behaviors, and health. Perspectives in Psychiatric Care. 2021;57(4):1905-12.

637. Labrague LJ, De los Santos JAA, Falguera CC. Social and emotional loneliness among college students during the COVID-19 pandemic: The predictive role of coping behaviors, social support, and personal resilience. Perspectives in Psychiatric Care. 2021;57(4):1578-84.

638. Lade K, Chib S, Karangutkar S, Jha RK. Impact of COVID-19 on mental health of management students. European Journal of Molecular and Clinical Medicine. 2021;8(1):275-86.

639. Laher S, Bain K, Bemath N, de Andrade V, Hassem T. Undergraduate psychology student experiences during COVID-19: challenges encountered and lessons learnt. S Afr J Psychol. 2021;51(2):215-28.

640. Lahiri A, Jha SS, Acharya R, Dey A, Chakraborty A. Has loneliness and poor resilient coping influenced the magnitude of psychological distress among apparently healthy Indian adults during the lockdown? Evidence from a rapid online nation-wide cross-sectional survey. Plos One. 2021;16(1).

641. Lahtinen O, Aaltonen J, Kaakinen J, Franklin L, Hyona J. The effects of app-based mindfulness practice on the well-being of university students and staff. Curr Psychol. 2021:1-10.

642. Lai AY, Lee L, Wang MP, Feng Y, Lai TT, Ho LM, et al. Mental Health Impacts of the COVID-19 Pandemic on International University Students, Related Stressors, and Coping Strategies. Front Psychiatry. 2020;11:584240.

643. Lal A, Sanaullah A, MK MS, Ahmed N, Maqsood A, Ahmed N. Psychological Distress among Adults in Home Confinement in the Midst of COVID-19 Outbreak. Eur J Dent. 2020;14:S27-s33.

644. Lancaster M, Arango E. Health and Emotional Well-Being of Urban University Students in the Era of COVID-19. Traumatology. 2021;27(1):107-17.

645. Lancaster RJ, Schmitt C, Debish M. A qualitative examination of graduating nurses' response to the Covid-19 pandemic. Nurs Ethics. 2021;28(7-8):1337-47.

646. Landa-Blanco M, Landa-Blanco A, Mejía-Suazo CJ, Martínez-Martínez CA. Coronavirus awareness and mental health: clinical symptoms and attitudes toward seeking professional psychological help. Frontiers in Psychology. 2021;12:549644.

647. Landaeta-Díaz L, González-Medina G, Agüero SD. Anxiety, anhedonia and food consumption during the COVID-19 quarantine in Chile. Appetite. 2021;164:105259.

648. Lardone A, Sorrentino P, Giancamilli F, Palombi T, Simper T, Mandolesi L, et al. Psychosocial variables and quality of life during the COVID-19 lockdown: a correlational study on a convenience sample of young Italians. Peerj. 2020;8:e10611.

649. Laslo-Roth R, Bareket-Bojmel L, Margalit M. Loneliness experience during distance learning among college students with ADHD: the mediating role of perceived support and hope. Eur J Spec Needs Edu. 2022;37(2):220-34.

650. Lathabhavan R, Vispute S. Examining the Mediating Effects of Stress on Fear of COVID-19 and Well-being Using Structural Equation Modeling. Int J Ment Health Ad. 2021.

651. Lathika AR, Soman B. COVID-19 on youth mental health. Med J Armed Forces India. 2021;77(1):111-2.

652. Lavender DM, Dekker AP, Tambe AA. Rising to the challenge: medical students as Doctors' Assistants; an evaluation of a new clinical role. J Adv Med Educ Prof. 2021;9(1):26-33.

653. Lawal AM, Alhassan EO, Mogaji HO, Odoh IM, Essien EA. Differential effect of gender, marital status, religion, ethnicity, education and employment status on mental health during COVID-19 lockdown in Nigeria. Psychol Health Med. 2022;27(1):1-12.

654. Lawrence SA, Garcia J, Stewart C, Rodriguez C. The mental and behavioral health impact of COVID-19 stay at home orders on social work students. Soc Work Educ. 2022;41(4):707-21.

655. Le Vigouroux S, Goncalves A, Charbonnier E. The Psychological Vulnerability of French University Students to the COVID-19 Confinement. Health Educ Behav. 2021;48(2):123-31.

656. Lechner WV, Laurene KR, Patel S, Anderson M, Grega C, Kenne DR. Changes in alcohol use as a function of psychological distress and social support following COVID-19 related University closings. Addict Behav. 2020;110:106527.

657. Lechner WV, Sidhu NK, Jin JT, Kittaneh AA, Laurene KR, Kenne DR. Increases in Risky Drinking During the COVID-19 Pandemic Assessed via Longitudinal Cohort Design: Associations With Racial Tensions, Financial Distress, Psychological Distress and Virus-Related Fears. Alcohol Alcohol. 2021;56(6):702-7.

658. Lee HS, Dean D, Baxter T, Griffith T, Park S. Deterioration of mental health despite successful control of the COVID-19 pandemic in South Korea. Psychiat Res. 2021;295:113570.

659. Lee J, Jeong HJ, Kim S. Stress, Anxiety, and Depression Among Undergraduate Students during the COVID-19 Pandemic and their Use of Mental Health Services. Innov High Educ. 2021;46(5):519-38.

660. Lee SJ, Natour AK, Geevarghese SK. Fireside Chats: A Novel Wellness Initiative for Medical Students in the COVID-19 Era. Am Surg. 2022;88(7):1703-7.

661. Lee Y, Yang BX, Liu Q, Luo D, Kang LJ, Yang F, et al. Synergistic effect of social media use and psychological distress on depression in China during the COVID-19 epidemic. Psychiatry and Clinical Neurosciences. 2020;74(10):552-3.

662. Lee YC, Wu WL, Lee CK. How COVID-19 Triggers Our Herding Behavior? Risk Perception, State Anxiety, and Trust. Front Public Health. 2021;9:587439.

663. Lee YR, Lee JY, Park IH, Kim M, Jhon M, Kim JW, et al. The Relationships among Media Usage Regarding COVID-19, Knowledge about Infection, and Anxiety: Structural Model Analysis. J Korean Med Sci. 2020;35(48):e426.

664. Lei L, Huang X, Zhang S, Yang J, Yang L, Xu M. Comparison of Prevalence and Associated Factors of Anxiety and Depression Among People Affected by versus People Unaffected by Quarantine During the COVID-19 Epidemic in Southwestern China. Med Sci Monit. 2020;26:e924609.

665. Leiros-Rodriguez R, Rodriguez-Nogueira O, Pinto-Carral A, Alvarez-Alvarez MJ, Galan-Martin MA, Montero-Cuadrado F, et al. Musculoskeletal Pain and Non-Classroom Teaching in Times of the COVID-19 Pandemic: Analysis of the Impact on Students from Two Spanish Universities. J Clin Med. 2020;9(12).

666. Levkovich I, Shinan-Altman S. Impact of the COVID-19 pandemic on stress and emotional reactions in Israel: a mixed-methods study. Int Health. 2021;13(4):358-66.

667. Li D, Zou L, Zhang Z, Zhang P, Zhang J, Fu W, et al. The Psychological Effect of COVID-19 on Home-Quarantined Nursing Students in China. Front Psychiatry. 2021;12:652296.

668. Li H, Zheng L, Le H, Zhuo LJ, Wu Q, Ma GQ, et al. The Mediating Role of Internalized Stigma and Shame on the Relationship between COVID-19 Related Discrimination and Mental Health Outcomes among Back-to-School Students in Wuhan. International Journal of Environmental Research and Public Health. 2020;17(24).

669. Li HY, Cao H, Leung DYP, Mak YW. The Psychological Impacts of a COVID-19 Outbreak on College Students in China: A Longitudinal Study. Int J Environ Res Public Health. 2020;17(11).

670. Li JB, Yang A, Dou K, Cheung RYM. Self-Control Moderates the Association Between Perceived Severity of Coronavirus Disease 2019 (COVID-19) and Mental Health Problems Among the Chinese Public. International Journal of Environmental Research and Public Health. 2020;17(13).

671. Li L, Liu G, Xu W, Zhang Y, He M. Effects of Internet Hospital Consultations on Psychological Burdens and Disease Knowledge During the Early Outbreak of COVID-19 in China: Cross-Sectional Survey Study. J Med Internet Res. 2020;22(8):e19551.

672. Li M, Liu L, Yang Y, Wang Y, Yang X, Wu H. Psychological Impact of Health Risk Communication and Social Media on College Students During the COVID-19 Pandemic: Cross-Sectional Study. J Med Internet Res. 2020;22(11):e20656.

673. Li Q. Psychosocial and coping responses toward 2019 coronavirus diseases (COVID-19): a cross-sectional study within the Chinese general population. Qjm-Int J Med. 2020;113(10):731-8.

674. Li TMH, Leung CSY. Exploring student mental health and intention to use online counseling in Hong Kong during the covid‐19 pandemic. Psychiatry and Clinical Neurosciences. 2020;74(10):564-5.

675. Li WW, Yu HZ, Miller DJ, Yang F, Rouen C. Novelty Seeking and Mental Health in Chinese University Students Before, During, and After the COVID-19 Pandemic Lockdown: A Longitudinal Study. Frontiers in Psychology. 2020;11:600739.

676. Li X, Fu P, Fan C, Zhu M, Li M. COVID-19 Stress and Mental Health of Students in Locked-Down Colleges. Int J Environ Res Public Health. 2021;18(2).

677. Li X, Lv S, Liu L, Chen R, Chen J, Liang S, et al. COVID-19 in Guangdong: Immediate Perceptions and Psychological Impact on 304,167 College Students. Front Psychol. 2020;11:2024.

678. Li X, Wu H, Meng F, Li L, Wang Y, Zhou M. Relations of COVID-19-Related Stressors and Social Support With Chinese College Students' Psychological Response During the COVID-19 Pandemic. Front Psychiatry. 2020;11:551315.

679. Li XJ, Lv QY, Tang WJ, Deng W, Zhao LS, Meng YJ, et al. Psychological stresses among Chinese university students during the COVID-19 epidemic: The effect of early life adversity on emotional distress. Journal of Affective Disorders. 2021;282:33-8.

680. Li XL, Yu HG, Yang WQ, Mo QH, Yang ZG, Wen SS, et al. Depression and Anxiety Among Quarantined People, Community Workers, Medical Staff, and General Population in the Early Stage of COVID-19 Epidemic. Frontiers in Psychology. 2021;12.

681. Li Y, Qin L, Shi Y, Han J. The Psychological Symptoms of College Student in China during the Lockdown of COVID-19 Epidemic. Healthcare (Basel). 2021;9(4).

682. Li Y, Wang Y, Jiang J, Valdimarsdóttir UA, Fall K, Fang F, et al. Psychological distress among health professional students during the COVID-19 outbreak. Psychol Med. 2021:1-3.

683. Li Y, Zhao J, Ma Z, McReynolds LS, Lin D, Chen Z, et al. Mental health among college students during the covid-19 pandemic in china: A 2-wave longitudinal survey. Journal of Affective Disorders. 2020.

684. Liang L, Gao T, Ren H, Cao R, Qin Z, Hu Y, et al. Post-traumatic stress disorder and psychological distress in Chinese youths following the COVID-19 emergency. J Health Psychol. 2020;25(9):1164-75.

685. Liang L, Ren H, Cao R, Hu Y, Qin Z, Li C, et al. The Effect of COVID-19 on Youth Mental Health. Psychiatr Q. 2020;91(3):841-52.

686. Liang S-W, Chen R-N, Liu L-L, Li X-G, Chen J-B, Tang S-Y, et al. The psychological impact of the COVID-19 epidemic on Guangdong college students: The difference between seeking and not seeking psychological help. Frontiers in Psychology. 2020;11.

687. Ligus K, Fritzson E, Hennessy EA, Acabchuk RL, Bellizzi K. Disruptions in the management and care of university students with preexisting mental health conditions during the COVID-19 pandemic. Transl Behav Med. 2021;11(3):802-7.

688. Lin D, Friedman DB, Qiao S, Tam CC, Li X, Li X. Information uncertainty: a correlate for acute stress disorder during the COVID-19 outbreak in China. Bmc Public Health. 2020;20(1):1867.

689. Lin JY, Guo TY, Becker B, Yu Q, Chen ST, Brendon S, et al. Depression is Associated with Moderate-Intensity Physical Activity Among College Students During the COVID-19 Pandemic: Differs by Activity Level, Gender and Gender Role. Psychology Research and Behavior Management. 2020;13:1123-34.

690. Lin L. Longitudinal associations of meaning in life and psychosocial adjustment to the COVID-19 outbreak in China. Br J Health Psychol. 2021;26(2):525-34.

691. Lin LY, Wang J, Ou-Yang XY, Miao Q, Chen R, Liang FX, et al. The immediate impact of the 2019 novel coronavirus (COVID-19) outbreak on subjective sleep status. Sleep Med. 2021;77:348-54.

692. Lin Y, Liu S, Li S, Zuo H, Zhang B. Relationships between the changes in sleep patterns and sleep quality among Chinese people during the 2019 coronavirus disease outbreak. Sleep Medicine. 2021.

693. Lin YL, Hu ZJ, Alias H, Wong LP. Influence of Mass and Social Media on Psychobehavioral Responses Among Medical Students During the Downward Trend of COVID-19 in Fujian, China: Cross-Sectional Study. J Med Internet Res. 2020;22(7):e19982.

694. Lindau ST, Makelarski JA, Boyd K, Doyle KE, Haider S, Kumar S, et al. Change in Health-Related Socioeconomic Risk Factors and Mental Health During the Early Phase of the COVID-19 Pandemic: A National Survey of U.S. Women. J Womens Health (Larchmt). 2021;30(4):502-13.

695. Lindemann IL, Simonetti AB, Amaral CPd, Riffel RT, Simon TT, Stobbe JC, et al. Perception of fear of being infected by the new coronavirus. J bras psiquiatr. 2021;70(1):3-11.

696. Lindinger-Sternart S, Kaur V, Widyaningsih Y, Patel AK. COVID-19 phobia across the world: Impact of resilience on COVID-19 phobia in different nations. Couns Psychother Res. 2021;21(2):290-302.

697. Lingawi HS, Afifi IK. COVID-19 Associated Stress Among Dental Students. Open Dent J. 2020;14(1):554-62.

698. Lippke S, Fischer MA, Ratz T. Physical Activity, Loneliness, and Meaning of Friendship in Young Individuals - A Mixed-Methods Investigation Prior to and During the COVID-19 Pandemic With Three Cross-Sectional Studies. Front Psychol. 2021;12:617267.

699. Lischer S, Safi N, Dickson C. Remote learning and students' mental health during the Covid-19 pandemic: A mixed-method enquiry. Prospects (Paris). 2021:1-11.

700. Liu C, Lee YC, Lin YL, Yang SY. Factors associated with anxiety and quality of life of the Wuhan populace during the COVID-19 pandemic. Stress Health. 2021;37(5):887-97.

701. Liu C, Liu D, Huang N, Fu M, Ahmed JF, Zhang Y, et al. The Combined Impact of Gender and Age on Post-traumatic Stress Symptoms, Depression, and Insomnia During COVID-19 Outbreak in China. Front Public Health. 2020;8:620023.

702. Liu J, Fan W, Makamure J, Zheng C, Wang J, Zhu Q. Online Mental Health Survey in a Medical College in China During the COVID-19 Outbreak. Frontiers in Psychiatry. 2020;11:459.

703. Liu S, Heinzel S, Haucke MN, Heinz A. Increased Psychological Distress, Loneliness, and Unemployment in the Spread of COVID-19 over 6 Months in Germany. Medicina (Kaunas). 2021;57(1).

704. Liu S, Lithopoulos A, Zhang CQ, Garcia-Barrera MA, Rhodes RE. Personality and perceived stress during COVID-19 pandemic: Testing the mediating role of perceived threat and efficacy. Personality and Individual Differences. 2021;168:110351.

705. Liu S, Xi HT, Zhu QQ, Ji MM, Zhang HY, Yang BX, et al. The prevalence of fatigue among Chinese nursing students in post-COVID-19 era. Peerj. 2021;9:e11154.

706. Liu SY, Liu Y, Liu Y. Somatic symptoms and concern regarding COVID-19 among Chinese college and primary school students: A cross-sectional survey. Psychiat Res. 2020;289:113070.

707. Liu Y, Li P, Lv Y, Hou X, Rao Q, Tan J, et al. Public awareness and anxiety during COVID-19 epidemic in China: A cross-sectional study. Compr Psychiatry. 2021;107:152235.

708. Lo Coco G, Gentile A, Bosnar K, Milovanovic I, Bianco A, Drid P, et al. A Cross-Country Examination on the Fear of COVID-19 and the Sense of Loneliness during the First Wave of COVID-19 Outbreak. Int J Environ Res Public Health. 2021;18(5).

709. Lo Moro G, Soneson E, Jones PB, Galante J. Establishing a Theory-Based Multi-Level Approach for Primary Prevention of Mental Disorders in Young People. Int J Environ Res Public Health. 2020;17(24).

710. Loda T, Loffler T, Erschens R, Zipfel S, Herrmann-Werner A. Medical education in times of COVID-19: German students' expectations - A cross-sectional study. Plos One. 2020;15(11):e0241660.

711. Long Y, Quan FY, Zheng Y. Effects of Bicultural Identity Integration and National Identity on COVID-19-Related Anxiety Among Ethnic Minority College Students: The Mediation Role of Power Values. Psychology Research and Behavior Management. 2021;14:239-49.

712. Lopes B, Bortolon C, Jaspal R. Paranoia, hallucinations and compulsive buying during the early phase of the COVID-19 outbreak in the United Kingdom: A preliminary experimental study. Psychiatry Res. 2020;293:113455.

713. López Steinmetz LC, Fong SB, Godoy JC. Longitudinal evidence on mental health changes of college students with and without mental disorder background during the Argentina's lengthy mandatory quarantine. Prog Neuropsychopharmacol Biol Psychiatry. 2021;110:110308.

714. Lopez-Castro T, Brandt L, Anthonipillai NJ, Espinosa A, Melara R. Experiences, impacts and mental health functioning during a COVID-19 outbreak and lockdown: Data from a diverse New York City sample of college students. Plos One. 2021;16(4):e0249768.

715. Lopez-Moreno M, Lopez MTI, Miguel M, Garces-Rimon M. Physical and Psychological Effects Related to Food Habits and Lifestyle Changes Derived from Covid-19 Home Confinement in the Spanish Population. Nutrients. 2020;12(11):1-17.

716. Lu PX, Li X, Lu L, Zhang Y. The psychological states of people after Wuhan eased the lockdown. Plos One. 2020;15(11):e0241173.

717. Lu WH, Ko NY, Chang YP, Yen CF, Wang PW. The Coronavirus Disease 2019 Pandemic in Taiwan: An Online Survey on Worry and Anxiety and Associated Factors. Int J Environ Res Public Health. 2020;17(21):1-13.

718. Lucia Yetunde O. Psychological state and family functioning of University of Ibadan students during the COVID-19 lockdown. 2020.

719. Lukács A. Mental well-being of university students in social isolation. European Journal of Health Psychology. 2021;28(1):22-9.

720. Lyons Z, Wilcox H, Leung L, Dearsley O. COVID-19 and the mental well-being of Australian medical students: impact, concerns and coping strategies used. Australas Psychiatry. 2020;28(6):649-52.

721. Ma H, Miller C. Trapped in a Double Bind: Chinese Overseas Student Anxiety during the COVID-19 Pandemic. Health Commun. 2021;36(13):1598-605.

722. Ma Z, Zhao J, Li Y, Chen D, Wang T, Zhang Z, et al. Mental health problems and correlates among 746 217 college students during the coronavirus disease 2019 outbreak in China. Epidemiol Psych Sci. 2020;29:e181.

723. Maarefvand M, Hosseinzadeh S, Farmani O, Safarabadi Farahani A, Khubchandani J. Coronavirus Outbreak and Stress in Iranians. International Journal of Environmental Research and Public Health [Internet]. 2020; 17(12).

724. Macaraan MC. Mental health and legal education in the time of pandemic. J Public Health (Oxf). 2021;43(3):e525-e6.

725. Mack DL, DaSilva AW, Rogers C, Hedlund E, Murphy EI, Vojdanovski V, et al. Mental Health and Behavior of College Students During the COVID-19 Pandemic: Longitudinal Mobile Smartphone and Ecological Momentary Assessment Study, Part II. J Med Internet Res. 2021;23(6):e28892.

726. Mahamid FA, Bdier D. The Association Between Positive Religious Coping, Perceived Stress, and Depressive Symptoms During the Spread of Coronavirus (COVID-19) Among a Sample of Adults in Palestine: Across Sectional Study. J Relig Health. 2021;60(1):34-49.

727. Mahat N, Zubaidi SA, Soe HHK, Nah SA. Paediatric surgical response to an 'adult' COVID-19 pandemic. Med J Malaysia. 2021;76(3):284-90.

728. Maher JP, Hevel DJ, Reifsteck EJ, Drollette ES. Physical activity is positively associated with college students&#039; positive affect regardless of stressful life events during the COVID-19 pandemic. Psychology of Sport and Exercise. 2020:101826-.

729. Mahmood QK, Jafree SR, Sohail MM, Akram MB. A Cross-Sectional Survey of Pakistani Muslims Coping with Health Anxiety through Religiosity during the COVID-19 Pandemic. J Relig Health. 2021;60(3):1462-74.

730. Mahmood Z, Saleem S, Subhan S, Jabeen A. Psychosocial Reactions of Pakistani Students Towards COVID-19: A Prevalence Study. Pak J Med Sci. 2021;37(2):456-60.

731. Mahmoudi H, Saffari M, Movahedi M, Sanaeinasab H, Rashidi-Jahan H, Pourgholami M, et al. A mediating role for mental health in associations between COVID-19-related self-stigma, PTSD, quality of life, and insomnia among patients recovered from COVID-19. Brain Behav. 2021;11(5):e02138.

732. Mahmud Z, Rahim RA, Abidin AWZ, Abdullah NNN. Mental and Emotional Wellbeing During the COVID-19 Pandemic: The Unprecedented Malaysian Experience. Curr Psychiat Res Re. 2020;16(4):244-56.

733. Maia BR, Dias PC. Anxiety, depression and stress in university students: the impact of COVID-19. Estud Psicol (Campinas, Online). 2020;37:e200067-e.

734. Mair TS, Mountford DR, Radley R, Lockett E, Parkin TD. Mental wellbeing of equine veterinary surgeons, veterinary nurses and veterinary students during the COVID‐19 pandemic. Equine Veterinary Education. 2020:eve.13399-eve.

735. Majeed S, Schwaiger EM, Nazim A, Samuel IS. The Psychological Impact of COVID-19 Among Pakistani Adults in Lahore. Front Public Health. 2021;9:578366.

736. Majumdar P, Biswas A, Sahu S. COVID-19 pandemic and lockdown: cause of sleep disruption, depression, somatic pain, and increased screen exposure of office workers and students of India. Chronobiol Int. 2020;37(8):1191-200.

737. Majumder MAA, Cohall D, Ojeh N, Campbell MH, Adams OP, Sa B, et al. Assessing online learning readiness and perceived stress among first year medical students during COVID-19 pandemic: a multi-country study. Can Med Educ J. 2021;12(2):e131-e3.

738. Maldonato NM, Bottone M, Chiodi A, Continisio GI, De Falco A, Duval M, et al. A Mental Health First Aid Service in an Italian University Public Hospital during the Coronavirus Disease 2019 Outbreak. Sustainability. 2020;12(10).

739. Malesza M, Kaczmarek MC. Predictors of anxiety during the COVID-19 pandemic in Poland. Pers Individ Dif. 2021;170:110419.

740. Mamun MA, Akter T, Zohra F, Sakib N, Bhuiyan AKMI, Banik PC, et al. Prevalence and risk factors of COVID-19 suicidal behavior in Bangladeshi population: are healthcare professionals at greater risk? Heliyon. 2020;6(10).

741. Mamun MA, Sakib N, Gozal D, Bhuiyan AI, Hossain S, Bodrud-Doza M, et al. The COVID-19 pandemic and serious psychological consequences in Bangladesh: A population-based nationwide study. J Affect Disord. 2021;279:462-72.

742. Mangalam K, Adarsh S, Akshay A, Yamini S, Jyoti S, Megha Y, et al. Impact of covid-19 on stress in collegiate student. Medico-Legal Update. 2020;20(4):286-9.

743. Manis M, DeHart R, Kyle J, Bailey R. Incorporating a wellness initiative into a general medicine advanced pharmacy practice experience. JACCP Journal of the American College of Clinical Pharmacy. 2020;3(8):1559.

744. Manjareeka M, Pathak M. COVID-19 lockdown anxieties: Is student a vulnerable group? Journal of Indian Association for Child and Adolescent Mental Health. 2020;17(1):72-80.

745. Marchetti D, Fontanesi L, Mazza C, Di Giandomenico S, Roma P, Verrocchio MC. Parenting-Related Exhaustion During the Italian COVID-19 Lockdown. J Pediatr Psychol. 2020;45(10):1114-23.

746. Marelli S, Castelnuovo A, Somma A, Castronovo V, Mombelli S, Bottoni D, et al. Impact of COVID-19 lockdown on sleep quality in university students and administration staff. J Neurol. 2021;268(1):8-15.

747. María del Carmen PF, María del Mar MJ, Martínez ÁM, Gázquez Linares JJ. Threat of COVID-19 and emotional state during quarantine: Positive and negative affect as mediators in a cross-sectional study of the Spanish population. Plos One. 2020;15(6).

748. Marques G, Drissi N, Diez ID, de Abajo BS, Ouhbi S. Impact of COVID-19 on the psychological health of university students in Spain and their attitudes toward Mobile mental health solutions. Int J Med Inform. 2021;147:104369.

749. Marschalko EE, Kotta I, Kalcza-Janosi K, Szabo K, Jancso-Farcas S. Psychological Predictors of COVID-19 Prevention Behavior in Hungarian Women Across Different Generations. Front Psychol. 2021;12:596543.

750. Martin-Delgado L, Goni-Fuste B, Alfonso-Arias C, De Juan M, Wennberg L, Rodriguez E, et al. Nursing students on the frontline: Impact and personal and professional gains of joining the health care workforce during the COVID-19 pandemic in Spain. J Prof Nurs. 2021;37(3):588-97.

751. Martinez L, Valencia I, Trofimoff V. Subjective wellbeing and mental health during the COVID-19 pandemic: Data from three population groups in Colombia. Data Brief. 2020;32:106287.

752. Martinez-Marti ML, Theirs CI, Pascual D, Corradi G. Character Strengths Predict an Increase in Mental Health and Subjective Well-Being Over a One-Month Period During the COVID-19 Pandemic Lockdown. Front Psychol. 2020;11:584567.

753. Marzo RR, Ismail Z, Htay MNN, Bahari R, Ismail R, Villanueva EQ, et al. Psychological distress during pandemic Covid-19 among adult general population: Result across 13 countries. Clin Epidemiol Glob. 2021;10:100708.

754. Masha'al D, Rababa M, Shahrour G. Distance Learning-Related Stress Among Undergraduate Nursing Students During the COVID-19 Pandemic. J Nurs Educ. 2020;59(12):666-+.

755. Matos M, McEwan K, Kanovsky M, Halamova J, Steindl SR, Ferreira N, et al. Fears of compassion magnify the harmful effects of threat of COVID-19 on mental health and social safeness across 21 countries. Clin Psychol Psychother. 2021;28(6):1317-33.

756. Matsungo TM, Chopera P. Effect of the COVID-19-induced lockdown on nutrition, health and lifestyle patterns among adults in Zimbabwe. BMJ Nutr Prev Health. 2020;3(2):205-12.

757. Mautong H, Gallardo-Rumbea JA, Alvarado-Villa GE, Fernandez-Cadena JC, Andrade-Molina D, Orellana-Roman CE, et al. Assessment of depression, anxiety and stress levels in the Ecuadorian general population during social isolation due to the COVID-19 outbreak: a cross-sectional study. Bmc Psychiatry. 2021;21(1).

758. Mazza M, Attanasio M, Pino MC, Masedu F, Tiberti S, Sarlo M, et al. Moral Decision-Making, Stress, and Social Cognition in Frontline Workers vs. Population Groups During the COVID-19 Pandemic: An Explorative Study. Front Psychol. 2020;11:588159.

759. Mazzarella C, Spina A, Dallio M, Gravina AG, Romeo M, M DIM, et al. The analysis of alcohol consumption during the severe acute respiratory syndrome coronavirus 2 Italian lockdown. Minerva Med. 2021.

760. Mboua PC, Siakam C, Nguepy Keubo FR. [Trauma and resilience associated with the COVID-19 pandemic in the cities of Bafoussam and Dschang in Cameroon]. Ann Med Psychol (Paris). 2021;179(9):812-7.

761. Mboua PCSCMNL. Impact of the resumption of classes on the mental health of students of the Faculty of Letters and Social Sciences of the University of Dschang, in the context of Covid 19. Journal of Affective Disorders Reports. 2021;5:100147-.

762. Mckay D, Yang HB, Elhai J, Asmundson GJG. Anxiety regarding contracting COVID-19 related to interoceptive anxiety sensations: The moderating role of disgust propensity and sensitivity. Journal of Anxiety Disorders. 2020;73:102233.

763. McQuade BM, Jarrett JB. Do you mind? A mindfulness practice curriculum for the pharmacy workforce. JACCP Journal of the American College of Clinical Pharmacy. 2020;3(8):1616.

764. Mechili EA, Saliaj A, Kamberi F, Girvalaki C, Peto E, Patelarou AE, et al. Is the mental health of young students and their family members affected during the quarantine period? Evidence from the covid‐19 pandemic in albania. Journal of Psychiatric and Mental Health Nursing. 2020.

765. Meda N, Pardini S, Slongo I, Bodini L, Zordan MA, Rigobello P, et al. Students' mental health problems before, during, and after COVID-19 lockdown in Italy. J Psychiatr Res. 2021;134:69-77.

766. Medeiros MS, Barreto DMS, Sampaio R, Alves BCFdB, Albino DCM, Fern, et al. Art as a Coping Strategy During the Pandemic. Rev bras educ méd. 2020;44:e130-e.

767. Medeiros RA, Vieira DL, Silva E, Rezende L, Santos RWD, Tabata LF. Prevalence of symptoms of temporomandibular disorders, oral behaviors, anxiety, and depression in Dentistry students during the period of social isolation due to COVID-19. J Appl Oral Sci. 2020;28:e20200445.

768. Megalakaki O, Kokou-Kpolou CK, Vaudé J, Park S, Iorfa SK, Cénat JM, et al. Does peritraumatic distress predict PTSD, depression and anxiety symptoms during and after COVID-19 lockdown in France? A prospective longitudinal study. J Psychiatr Res. 2021;137:81-8.

769. Megreya AM, Latzman RD, Al-Ahmadi AM, Al-Dosari NF. The COVID-19-Related Lockdown in Qatar: Associations Among Demographics, Social Distancing, Mood Changes, and Quality of Life. Int J Ment Health Ad. 2021.

770. Meira CM, Jr., Meneguelli KS, Leopoldo MPG, Florindo AA. Anxiety and Leisure-Domain Physical Activity Frequency, Duration, and Intensity During Covid-19 Pandemic. Front Psychol. 2020;11:603770.

771. Mejia CR, Ticona D, Rodriguez-Alarcon JF, Campos-Urbina AM, Catay-Medina JB, Porta-Quinto T, et al. The Media and their Informative Role in the Face of the Coronavirus Disease 2019 (COVID-19): Validation of Fear Perception and Magnitude of the Issue (MED-COVID-19). Electron J Gen Med. 2020;17(6).

772. Melcher J, Lavoie J, Hays R, D'Mello R, Rauseo-Ricupero N, Camacho E, et al. Digital phenotyping of student mental health during COVID-19: an observational study of 100 college students. J Am Coll Health. 2021:1-13.

773. Menacho-Vargas I, Supo-Condori F, Cavero-Ayvar HN, Caloretti-Castillo MJ, Lazo-Tafur YJ, Guerrero-Rojas JJ. Family Climate and Resilience in Peruvian Students in Times of Global Pandemic by Covid-19. Turismo. 2021:9-.

774. Meng N, Liu Z, Wang Y, Feng Y, Liu Q, Huang JQ, et al. Beyond Sociodemographic and COVID-19-Related Factors: The Association Between the Need for Psychological and Information Support from School and Anxiety and Depression. Med Sci Monitor. 2021;27:e929280.

775. Meng Y, Zhu M-Q, Liu N-Q, Liu W-Z, Wu L-L. Mental health status of college students under regular prevention and control of coronavirus disease 2019 epidemic. Academic Journal of Second Military Medical University. 2020;41(9):958-65.

776. Meo SA, Abukhalaf AA, Alomar AA, Sattar K, Klonoff DC. COVID-19 Pandemic: Impact of Quarantine on Medical Students' Mental Wellbeing and Learning Behaviors. Pak J Med Sci. 2020;36(COVID19-S4):S43-S8.

777. Mesa-Cano IC, Ochoa-Yumbla CDC, Abad-Martinez NI, Ramirez-Coronel AA, Martinez-Suarez PC, Pogyo-Morocho GL, et al. Psychometric assessment and nursing intervention in fear of covid-19. Project crimea. Archivos Venezolanos de Farmacologia y Terapeutica. 2020;39(5):660-6.

778. Meyer J, McDowell C, Lansing J, Brower C, Smith L, Tully M, et al. Changes in Physical Activity and Sedentary Behavior in Response to COVID-19 and Their Associations with Mental Health in 3052 US Adults. Int J Environ Res Public Health. 2020;17(18).

779. Mihatsch L, von der Linde M, Knolle F, Luchting B, Dimitriadis K, Heyn J. Survey of German medical students during the COVID-19 pandemic: attitudes toward volunteering versus compulsory service and associated factors. J Med Ethics. 2022;48(9):630-6.

780. Mikhailov A, Burlakova M. A Provincial University at the Times of the World Pandemic: Preservation or Development? International Dialogues on Education: Past and Present. 2020;7:19-21.

781. Miller I, Jensen K. Introduction of Mindfulness in an Online Engineering Core Course during the COVID-19 Pandemic. Advances in Engineering Education. 2020;8(4).

782. Mirhosseini S, Dadgari A, Basirinezhad MH, Mohammadpourhodki R, Ebrahimi H. The proportion of death anxiety and its related factors during the COVID-19 pandemic in the Iranian population. Fam Med Prim Care Re. 2021;23(1):36-40.

783. Misca G, Thornton G. Navigating the Same Storm but Not in the Same Boat: Mental Health Vulnerability and Coping in Women University Students During the First COVID-19 Lockdown in the UK. Front Psychol. 2021;12:648533.

784. Moayed MS, Vahedian-Azimi A, Mirmomeni G, Rahimi-Bashar F, Goharimoghadam K, Pourhoseingholi MA, et al. Coronavirus (COVID-19)-Associated Psychological Distress Among Medical Students in Iran. Adv Exp Med Biol. 2021;1321:245-51.

785. Mohammadpour M, Ghorbani V, Khoramnia S, Ahmadi SM, Ghvami M, Maleki M. Anxiety, Self-Compassion, Gender Differences and COVID-19: Predicting Self-Care Behaviors and Fear of COVID-19 Based on Anxiety and Self-Compassion with an Emphasis on Gender Differences. Iran J Psychiatry. 2020;15(3):213-9.

786. Mohammadzadeh F, Noghabi AD, Khosravan S, Bazeli J, Armanmehr V, Paykani T. Anxiety Severity Levels and Coping Strategies during the COVID-19 Pandemic among People Aged 15 Years and Above in Gonabad, Iran. Arch Iran Med. 2020;23(9):633-8.

787. Mohr CD, Umemoto SK, Rounds TW, Bouleh P, Arpin SN. Drinking to Cope in the COVID-19 Era: An Investigation Among College Students. J Stud Alcohol Drugs. 2021;82(2):178-87.

788. Molero Jurado MDM, Perez-Fuentes MDC, Fernandez-Martinez E, Martos Martinez A, Gazquez Linares JJ. Coping Strategies in the Spanish Population: The Role in Consequences of COVID-19 on Mental Health. Front Psychiatry. 2021;12:606621.

789. Molock SD, Parchem B. The impact of COVID-19 on college students from communities of color. J Am Coll Health. 2021:1-7.

790. Mongkhon P, Ruengorn C, Awiphan R, Thavorn K, Hutton B, Wongpakaran N, et al. Exposure to COVID-19-Related Information and its Association With Mental Health Problems in Thailand: Nationwide, Cross-sectional Survey Study. J Med Internet Res. 2021;23(2):e25363.

791. Morales-Rodríguez FM. Fear, Stress, Resilience and Coping Strategies during COVID-19 in Spanish University Students. Sustainability. 2021;13(11):5824-.

792. Moreira PS, Ferreira S, Couto B, Machado-Sousa M, Fernandez M, Raposo-Lima C, et al. Protective Elements of Mental Health Status during the COVID-19 Outbreak in the Portuguese Population. International Journal of Environmental Research and Public Health. 2021;18(4):1-11.

793. Moreland CJ, Paludneviciene R, Park JH, McKee M, Kushalnagar P. Deaf adults at higher risk for severe illness: COVID-19 information preference and perceived health consequences. Patient Education and Counseling. 2021;104(11):2830-3.

794. Morganti A, Brambilla A, Amerio A, Aguglia A, Odone A, Costanza A, et al. COVID-19 lockdown: housing built environment’s effects on mental health. European Journal of Public Health. 2020;30.

795. Moriarty T, Bourbeau K, Fontana F, McNamara S, da Silva MP. The Relationship between Psychological Stress and Healthy Lifestyle Behaviors during COVID-19 among Students in a US Midwest University. International Journal of Environmental Research and Public Health. 2021;18(9).

796. Mosanya M. Buffering Academic Stress during the COVID-19 Pandemic Related Social Isolation: Grit and Growth Mindset as Protective Factors against the Impact of Loneliness. Int J Appl Posit Psychol. 2021:1-16.

797. Mouloud K, Krine N. COVID-19 and the mental health of physical education students before and during lockdown. Viref-Rev Educ Fis. 2020;9(4):73-80.

798. Mousavi SA, Hooshyari Z, Ahmadi A. The Most Stressful Events during the COVID-19 Epidemic. Iran J Psychiatry. 2020;15(3):220-7.

799. Mousoulidou M, Siakalli M, Christodoulou A, Argyrides M. The Impact of the COVID-19 Pandemic on Mental Health: Evidence from Cyprus. Int J Environ Res Public Health. 2021;18(8).

800. Mridul, Bisht B, ana, Sharma D, Kaur N. Online classes during covid-19 pandemic: Anxiety, stress & depression among university students. Indian Journal of Forensic Medicine and Toxicology. 2021;15(1):186-9.

801. Mrklas K, Shalaby R, Hrabok M, Gusnowski A, Vuong W, Surood S, et al. Prevalence of Perceived Stress, Anxiety, Depression, and Obsessive-Compulsive Symptoms in Health Care Workers and Other Workers in Alberta During the COVID-19 Pandemic: Cross-Sectional Survey. JMIR Ment Health. 2020;7(9):e22408.

802. Mueller JT, McConnell K, Burow PB, Pofahl K, Merdjanoff AA, Farrell J. Impacts of the COVID-19 pandemic on rural America. Proc Natl Acad Sci U S A. 2021;118(1).

803. Muhamad AB, Pang NTP, Salvaraji L, Rahim SSSA, Jeffree MS, Omar A. Retrospective Analysis of Psychological Factors in COVID-19 Outbreak Among Isolated and Quarantined Agricultural Students in a Borneo University. Frontiers in Psychiatry. 2021;12:558591.

804. Muhammad Alfareed Zafar S, Junaid Tahir M, Malik M, Irfan Malik M, Kamal Akhtar F, Ghazala R. Awareness, anxiety, and depression in healthcare professionals, medical students, and general population of Pakistan during COVID-19 Pandemic: A cross sectional online survey. Med J Islam Repub Iran. 2020;34:131.

805. Muhammad I, Faizah S, Vincent H, Waqar A, Rosmaiza G. The psychological impact of coronavirus on university students and its socio-economic determinants in Malaysia. 2020.

806. Muhammad S, Noman A, Zia Ul M, Tahir Mehmood K, Naureen S, Khalid H, et al. Psychological Impact of COVID-19 on Pakistani University Students and How They Are Coping. 2020.

807. Mukherjee M, Maity C. Influence of media engagement on the post traumatic stress disorder in context of the COVID-19 pandemic: an empirical reflection from India. Journal of Human Behavior in the Social Environment. 2021;31(1-4):409-24.

808. Mukherjee M, Maity C, Chatterjee S. Media use pattern as an indicator of mental health in the COVID-19 pandemic: Dataset from India. Data Brief. 2021;34:106722.

809. Muley PP, Meshram KA, Muley PA. Effect of COVID-19 lockdown on perceived stress scale in medical students. Indian Journal of Forensic Medicine and Toxicology. 2020;14(4):6426-9.

810. Munk AJL, Schmidt NM, Alexander N, Henkel K, Hennig J. Covid-19-Beyond virology: Potentials for maintaining mental health during lockdown. Plos One. 2020;15(8):e0236688.

811. Munsell SE, O'Malley L, Mackey C. Coping with COVID. Educational Research: Theory and Practice. 2020;31(3):101-9.

812. Murphy L, Eduljee NB, Croteau K. College Student Transition to Synchronous Virtual Classes during the COVID-19 Pandemic in Northeastern United States. Pedagogical Research. 2020;5(4).

813. Mushquash AR, Grassia E. Coping during COVID-19: examining student stress and depressive symptoms. J Am Coll Health. 2021:1-4.

814. Mustafa RM, Alrabadi NN, Alshali RZ, Khader YS, Ahmad DM. Knowledge, Attitude, Behavior, and Stress Related to COVID-19 among Undergraduate Health Care Students in Jordan. Eur J Dent. 2020;14:S50-s5.

815. Nagasu M, Muto K, Yamamoto I. Impacts of anxiety and socioeconomic factors on mental health in the early phases of the COVID-19 pandemic in the general population in Japan: A web-based survey. Plos One. 2021;16(3):e0247705.

816. Nagata JM, Ganson KT, Whittle HJ, Chu J, Harris OO, Tsai AC, et al. Food Insufficiency and Mental Health in the U.S. During the COVID-19 Pandemic. Am J Prev Med. 2021;60(4):453-61.

817. Naidoo P, Cartwright D. Where to from here? Contemplating the impact of covid-19 on south african students and student counseling services in higher education. Journal of College Student Psychotherapy. 2020.

818. Nakhostin-Ansari A, Aghajani F, Khonji M, Aghajani R, Sherafati A, Shahmansouri N. Depression and anxiety among iranian medical students during COVID-19 pandemic. Iranian Journal of Psychiatry. 2020;15(3):228-35.

819. Nanjundaswamy MH, Pathak H, Chaturvedi SK. Perceived stress and anxiety during COVID-19 among psychiatry trainees. Asian Journal of Psychiatry. 2020;54:102282.

820. Naser AY, Dahmash EZ, Al-Rousan R, Alwafi H, Alrawashdeh HM, Ghoul I, et al. Mental health status of the general population, healthcare professionals, and university students during 2019 coronavirus disease outbreak in Jordan: A cross-sectional study. Brain Behav. 2020;10(8):e01730.

821. Nazar W, Leszkowicz J, Pienkowska A, Brzezinski M, Szlagatys-Sidorkiewicz A, Plata-Nazar K. Before-and-after online community survey on knowledge and perception of COVID-19 pandemic. BMC Infect Dis. 2020;20(1):861.

822. Ngussa BM, Fitriyah FK, Diningrat SWM. Correlation between Facebook Use, Mental Health and Learning Engagement: A Case of Universities in Surabaya City, Indonesia. Turk Online J Distan. 2021;22(1):229-45.

823. Ni J, Wang F, Liu Y, Wu M, Jiang Y, Zhou Y, et al. Psychological Impact of the COVID-19 Pandemic on Chinese Health Care Workers: Cross-Sectional Survey Study. JMIR Ment Health. 2021;8(1):e23125.

824. Nicola M, Susanna P, Irene S, Luca B, Paolo R, Francesco V, et al. COVID-19 and depressive symptoms in students before and during lockdown. 2020.

825. Nie X, Feng K, Wang S, Li Y. Factors Influencing Public Panic During the COVID-19 Pandemic. Front Psychol. 2021;12:576301.

826. Niedzwiedz CL, Green MJ, Benzeval M, Campbell D, Craig P, Demou E, et al. Mental health and health behaviours before and during the initial phase of the COVID-19 lockdown: longitudinal analyses of the UK Household Longitudinal Study. J Epidemiol Commun H. 2021;75(3):224-31.

827. Nihmath Nisha S, Francis YM, Balaji K, Raghunath G, Kumaresan M. A survey on anxiety and depression level among South Indian medical students during the COVID 19 pandemic. International Journal of Research in Pharmaceutical Sciences. 2020;11:779-86.

828. Nino M, Harris C, Drawve G, Fitzpatrick KM. Race and ethnicity, gender, and age on perceived threats and fear of COVID-19: Evidence from two national data sources. SSM Popul Health. 2021;13:100717.

829. Nishimura Y, Ochi K, Tokumasu K, Obika M, Hagiya H, Kataoka H, et al. Impact of the COVID-19 Pandemic on the Psychological Distress of Medical Students in Japan: Cross-sectional Survey Study. J Med Internet Res. 2021;23(2):e25232.

830. Nomura K, Minamizono S, Maeda E, Kim R, Iwata T, Hirayama J, et al. Cross-sectional survey of depressive symptoms and suicide-related ideation at a Japanese national university during the COVID-19 stay-home order. Environ Health Prev. 2021;26(1):30.

831. Norton EJ, Georgiou I, Fung A, Nazari A, Bandyopadhyay S, Saunders KEA. Personal protective equipment and infection prevention and control: a national survey of UK medical students and interim foundation doctors during the COVID-19 pandemic. J Public Health (Oxf). 2021;43(1):67-75.

832. Novacek DM, Hampton-Anderson JN, Ebor MT, Loeb TB, Wyatt GE. Mental health ramifications of the COVID-19 pandemic for Black Americans: Clinical and research recommendations. Psychol Trauma. 2020;12(5):449-51.

833. Nurunnabi M, Almusharraf N, Aldeghaither D. Mental health and well-being during the COVID-19 pandemic in higher education: Evidence from G20 countries. J Public Health Res. 2020;9(Suppl 1):2010.

834. Nurunnabi M, Hossain S, Chinna K, Sundarasen S, Khoshaim HB, Kamaludin K, et al. Coping strategies of students for anxiety during the COVID-19 pandemic in China: a cross-sectional study. F1000Res. 2020;9:1115.

835. O'Byrne L, Gavin B, Adamis D, Lim YX, McNicholas F. Levels of stress in medical students due to COVID-19. J Med Ethics. 2021;47(6):383-8.

836. Öcal A, Cvetković VM, Baytiyeh H, Tedim FMS, Zečević M. Public reactions to the disaster COVID-19: a comparative study in Italy, Lebanon, Portugal, and Serbia. Geomatics, Natural Hazards and Risk. 2020;11(1):1864-85.

837. Odriozola-González P, Planchuelo-Gómez Á, Irurtia MJ, de Luis-García R. Psychological effects of the COVID-19 outbreak and lockdown among students and workers of a Spanish university. Psychiatry Res. 2020;290:113108.

838. Olah AR, Ford TE. Humor styles predict emotional and behavioral responses to COVID-19. Humor. 2021;34(2):177-99.

839. Olaimat AN, Aolymat I, Elsahoryi N, Shahbaz HM, Holley RA. Attitudes, Anxiety, and Behavioral Practices Regarding COVID-19 among University Students in Jordan: A Cross-Sectional Study. Am J Trop Med Hyg. 2020;103(3):1177-83.

840. Olmos-Gómez MDC. Sex and Careers of University Students in Educational Practices as Factors of Individual Differences in Learning Environment and Psychological Factors during COVID-19. Int J Environ Res Public Health. 2020;17(14).

841. Olson R, Fryz R, Essemiah J, Crawford M, King A, Fateye B. Mental health impacts of COVID-19 lockdown on US college students: Results of a photoelicitation project. J Am Coll Health. 2021:1-11.

842. Oriane A, Sonja W, David G, Marie M, Spiller Tobias R. Physical and psychological health of medical students involved in the COVID-19 response in Switzerland. Swiss Medical Weekly. 2020;150(49).

843. Ouyang H, Wen J, Gu WY, Shen HY, Song K. Evaluation of the effect of the COVID-19 pandemic on clinical characteristics and psychological status in internet consultation respondents. Sci Progress-Uk. 2021;104(2):368504211014696.

844. Owais Nasim HM, Qamar L, Syed AUY, Saleem S, Khalid A, Javaid Z, et al. Real-time measurement of psychological impact due to E- Learning; among the undergraduate dental students during Covid-19. European Journal of Molecular and Clinical Medicine. 2021;8(1):1242-51.

845. Owen AJ, Tran T, Hammarberg K, Kirkman M, Fisher J, Group C-RIR. Poor appetite and overeating reported by adults in Australia during the coronavirus-19 disease pandemic: a population-based study. Public Health Nutr. 2021;24(2):275-81.

846. Oyetunji TP, Ogunmola OA, Oyelakin TT, Olorunsogbon OF, Ajayi FO. COVID-19-related risk perception, anxiety and protective behaviours among Nigerian adults: a cross-sectional study. J Public Health-Heid. 2021:1-9.

847. Ozamiz-Etxebarria N, Santa María MD, Munitis AE, Gorrotxategi MP. Reduction of COVID-19 Anxiety Levels Through Relaxation Techniques: A Study Carried Out in Northern Spain on a Sample of Young University Students. Front Psychol. 2020;11:2038.

848. Ozdemir S, Ng S, Chaudhry I, Finkelstein EA. Adoption of Preventive Behaviour Strategies and Public Perceptions About COVID-19 in Singapore. Int J Health Policy. 2022;11(5):579-91.

849. Ozdin S, Bayrak Ozdin S. Levels and predictors of anxiety, depression and health anxiety during COVID-19 pandemic in Turkish society: The importance of gender. Int J Soc Psychiatry. 2020;66(5):504-11.

850. Ozga D, Krupa S, Medrzycka-Dabrowska W, Knap M, Witt P. Search for factors of anxiety incidence in midwifery students during the COVID-19 pandemic. Perspect Psychiatr Care. 2021;57(4):2037-8.

851. Ozmen S, Ozkan O, Ozer O, Yanardag MZ. Investigation of COVID-19 Fear, Well-Being and Life Satisfaction in Turkish Society. Soc Work Public Health. 2021;36(2):164-77.

852. Padron I, Fraga I, Vieitez L, Montes C, Romero E. A Study on the Psychological Wound of COVID-19 in University Students. Front Psychol. 2021;12:589927.

853. Paico NIH, Sebastian SD, Vergaray JM, Arellano EGR, Navarro ER. Anxiety and aggressiveness in Peruvian postgraduate students in COVID-19 context. European Journal of Molecular and Clinical Medicine. 2021;8(3):713-26.

854. Pakenham KI, Landi G, Boccolini G, Furlani A, Grandi S, Tossani E. The moderating roles of psychological flexibility and inflexibility on the mental health impacts of COVID-19 pandemic and lockdown in Italy. J Contextual Behav Sci. 2020;17:109-18.

855. Palgi Y, Shrira A, Ring L, Bodner E, Avidor S, Bergman Y, et al. The loneliness pandemic: Loneliness and other concomitants of depression, anxiety and their comorbidity during the COVID-19 outbreak. Journal of Affective Disorders. 2020;275:109-11.

856. Palmer TJ, Chisholm LJ, Rolf CG, Morris CR. Deliberate practice and self-recorded demonstration of skill proficiency: One baccalaureate nursing school's response to the COVID-19 pandemic. Nurse Educ Pract. 2021;53:103071.

857. Palvolgyi A, Makai A, Premusz V, Trpkovici M, Acs P, Betlehem J, et al. A Preliminary Study on the Effect of the Covid-19 Pandemic on Sporting Behavior, Mindfulness and Well-Being. Health Probl Civiliz. 2020;14(3):157-64.

858. Panatik SA, Wan Mohd Yunus WMA, Badri SKZ, Mukhtar F. The Unprecedented Movement Control Order (Lockdown) and Factors Associated With the Negative Emotional Symptoms, Happiness, and Work-Life Balance of Malaysian University Students During the Coronavirus Disease (COVID-19) Pandemic. Frontiers in Psychiatry. 2020;11:566221.

859. Pandey D, Bansal S, Goyal S, Garg A, Sethi N, Pothiyill DI, et al. Psychological impact of mass quarantine on population during pandemics—The COVID-19 Lock-Down (COLD) study. Plos One. 2020;15(10):e0240501.

860. Pandey U, Corbett G, Mohan S, Reagu S, Kumar S, Farrell T, et al. Anxiety, Depression and Behavioural Changes in Junior Doctors and Medical Students Associated with the Coronavirus Pandemic: A Cross-Sectional Survey. The Journal of Obstetrics and Gynecology of India. 2021;71(1):33-7.

861. Pandita S, Mishra HG, Chib S. Psychological impact of covid-19 crises on students through the lens of Stimulus-Organism-Response (SOR) model. Child Youth Serv Rev. 2021;120:105783.

862. Pandya A, Lodha P. Mental Health of College Students Amidst COVID-19: Implications for Reopening of Colleges and Universities. Indian Journal of Psychological Medicine. 2021;43(3):274-5.

863. Papadopoulou A, Efstathiou V, Yotsidi V, Pomini V, Michopoulos I, Markopoulou E, et al. Suicidal ideation during COVID-19 lockdown in Greece: Prevalence in the community, risk and protective factors. Psychiatry Res. 2021;297:113713.

864. Park KH, Kim AR, Yang MA, Lim SJ, Park JH. Impact of the COVID-19 pandemic on the lifestyle, mental health, and quality of life of adults in South Korea. Plos One. 2021;16(2):e0247970.

865. Parola A, Rossi A, Tessitore F, Troisi G, Mannarini S. Mental Health Through the COVID-19 Quarantine: A Growth Curve Analysis on Italian Young Adults. Frontiers in Psychology. 2020;11:567484.

866. Parra RMR. Depression and Meaning of Life in University Students in Times of Pandemic. Int J Educ Psychol. 2020;9(3):223-42.

867. Parrado-Gonzalez A, Leon-Jariego JC. [Covid-19: factors associated with emotional distress and psychological morbidity in spanish population.]. Rev Esp Salud Publica. 2020;94.

868. Parthiban BVRMGRRC. Psychological impact of COVID 19 on the amount of perceived stress among college students studying across various streams in India during the period of lockdown. Journal of Evolution of Medical and Dental Sciences. 2020;9(39):2889-93.

869. Pasiga BD. Relationship knowledge transmission of covid-19 and fear of dental care during pandemic in South Sulawesi, Indonesia. Pesquisa Brasileira em Odontopediatria e Clinica Integrada. 2020;21:1-12.

870. Passavanti M, Argentieri A, Barbieri DM, Lou B, Wijayaratna K, Foroutan Mirhosseini AS, et al. The psychological impact of COVID-19 and restrictive measures in the world. J Affect Disord. 2021;283:36-51.

871. Passos L, Prazeres F, Teixeira A, Martins C. Impact on Mental Health Due to COVID-19 Pandemic: Cross-Sectional Study in Portugal and Brazil. International Journal of Environmental Research and Public Health. 2020;17(18).

872. Patelarou A, Mechili EA, Galanis P, Zografakis-Sfakianakis M, Konstantinidis T, Saliaj A, et al. Nursing students, mental health status during COVID-19 quarantine: evidence from three European countries. J Ment Health. 2021;30(2):164-9.

873. Pathoulas JT, Olson SJ, Idnani A, Farah RS, Hordinsky MK, Widge AS. Cross-sectional survey examining skin picking and hair pulling disorders during the COVID-19 pandemic. J Am Acad Dermatol. 2021;84(3):771-3.

874. Patias ND, Von Hohendorff J, Cozzer AJ, Flores PA, Scorsolini-Comin F. Mental Health and Coping Strategies in Undergraduate and Graduate Students During COVID-19 Pandemic. Trends in Psychology. 2021.

875. Patsali ME, Mousa DV, Papadopoulou EVK, Papadopoulou KKK, Kaparounaki CK, Diakogiannis I, et al. University students' changes in mental health status and determinants of behavior during the COVID-19 lockdown in Greece. Psychiatry Res. 2020;292:113298.

876. Paulino M, Dumas-Diniz R, Brissos S, Brites R, Alho L, Simoes MR, et al. COVID-19 in Portugal: exploring the immediate psychological impact on the general population. Psychol Health Med. 2021;26(1):44-55.

877. Paulo Afonso M, Am, a S, Ana Lara Navarrete F, ez, Barbara Neiva P, et al. Evaluation of Depression, Anxiety and Sleep Quality in the Brazilian Population During Social Isolation Due to the New Coronavirus (SARS-CoV-2) pandemic: the DEGAS-CoV Study. 2021.

878. Pavan G, Ponnala VR, Veeri RB, Kumar R, Murti K, Dhingra S, et al. Covid-19 impact on the mental health of indian pharmacy students: An online survey. International Journal of Pharmaceutical Research. 2021;13(2):3022-7.

879. Pawlak KM, Kral J, Khan R, Amin S, Bilal M, Lui RN, et al. Impact of COVID-19 on endoscopy trainees: an international survey. Gastrointest Endosc. 2020;92(4):925-35.

880. Pedrozo-Pupo JC, Pedrozo-Cortes MJ, Campo-Arias A. Perceived stress associated with COVID-19 epidemic in Colombia: an online survey. Cad Saude Publica. 2020;36(5):e00090520.

881. Pelaccia T, Sibilia J, Fels É, Gauer L, Mus, a A, et al. And if we had to do it all over again, would we send medical students to the emergency departments during a pandemic? Lessons learned from the COVID-19 outbreak. Intern Emerg Med. 2021:1-8.

882. Pellerin N, Raufaste E. Psychological Resources Protect Well-Being During the COVID-19 Pandemic: A Longitudinal Study During the French Lockdown. Front Psychol. 2020;11:590276.

883. Pellert M, Lasser J, Metzler H, Garcia D. Dashboard of Sentiment in Austrian Social Media During COVID-19. Front Big Data. 2020;3:32.

884. Peng S, Yang XY, Yang T, Zhang W, Cottrell RR. Uncertainty Stress, and Its Impact on Disease Fear and Prevention Behavior during the COVID-19 Epidemic in China: A Panel Study. Am J Health Behav. 2021;45(2):334-41.

885. Peng W, Berry EM. Coping with the Challenges of COVID-19 Using the Sociotype Framework: A Rehearsal for the Next Pandemic. Rambam Maimonides Me. 2021;12(1).

886. Pereira MBC, Am e, Villela aVABCNBAdSTCRPMFPCPTRSRINP. Stability in Mental Health Among Medical Students During COVID-19 Quarantine: A 3-Year Prospective Study (preprint). 2021.

887. Pérez Abreu MRGTJJTVOITAEBAED. Psychological disorders in medicine students during the active investigation of COVID-19. Medisan. 2020;24(4):537-48.

888. Perez-Carbonell L, Meurling IJ, Wassermann D, Gnoni V, Leschziner G, Weighall A, et al. Impact of the novel coronavirus (COVID-19) pandemic on sleep. J Thorac Dis. 2020;12:S163-S75.

889. Perez-Dominguez F, Polanco-Ilabaca F, Pinto-Toledo F, Michaeli D, Achiardi J, Santana V, et al. Lifestyle Changes Among Medical Students During COVID-19 Pandemic: A Multicenter Study Across Nine Countries. Health Educ Behav. 2021;48(4):446-54.

890. Petillion RJ, McNeil WS. Student Experiences of Emergency Remote Teaching: Impacts of Instructor Practice on Student Learning, Engagement, and Well-Being. J Chem Educ. 2020;97(9):2486-93.

891. Pfund GN, Hill PL, Harriger J. Video chatting and appearance satisfaction during COVID-19: Appearance comparisons and self-objectification as moderators. Int J Eat Disord. 2020;53(12):2038-43.

892. Pham KM, Pham LV, Phan DT, Tran TV, Nguyen HC, Nguyen MH, et al. Healthy Dietary Intake Behavior Potentially Modifies the Negative Effect of COVID-19 Lockdown on Depression: A Hospital and Health Center Survey. Front Nutr. 2020;7:581043.

893. Pieh C, Budimir S, Delgadillo J, Barkham M, Fontaine JRJ, Probst T. Mental Health During COVID-19 Lockdown in the United Kingdom. Psychosom Med. 2021;83(4):328-37.

894. Pieh C, Budimir S, Probst T. The effect of age, gender, income, work, and physical activity on mental health during coronavirus disease (COVID-19) lockdown in Austria. J Psychosom Res. 2020;136:110186.

895. Pierce M, McManus S, Hope H, Hotopf M, Ford T, Hatch SL, et al. Mental health responses to the COVID-19 pandemic: a latent class trajectory analysis using longitudinal UK data. Lancet Psychiat. 2021;8(7):610-9.

896. Piltch-Loeb R, Merdjanoff A, Meltzer G. Anticipated mental health consequences of COVID-19 in a nationally-representative sample: Context, coverage, and economic consequences. Prev Med. 2021;145:106441.

897. Pinchoff J, Santhya KG, White C, Rampal S, Acharya R, Ngo TD. Gender specific differences in COVID-19 knowledge, behavior and health effects among adolescents and young adults in Uttar Pradesh and Bihar, India. Plos One. 2020;15(12):e0244053.

898. Pirnia B, Dezhakam H, Pirnia K, Malekanmehr P, Soleimani AA, Zahiroddin A, et al. COVID-19 pandemic and addiction: Current problems in Iran. Asian Journal of Psychiatry. 2020;54:102313.

899. Plath AM. Wellness and self-care of counselor education and supervision doctoral students: ProQuest Information & Learning; 2021.

900. Pradhan M, Chettri A, Maheshwari S. Fear of death in the shadow of COVID-19: The mediating role of perceived stress in the relationship between neuroticism and death anxiety. Death Studies. 2022;46(5):1106-10.

901. Pramukti I, Strong C, Sitthimongkol Y, Setiawan A, Pandin MGR, Yen CF, et al. Anxiety and Suicidal Thoughts During the COVID-19 Pandemic: Cross-Country Comparative Study Among Indonesian, Taiwanese, and Thai University Students. J Med Internet Res. 2020;22(12):e24487.

902. Pratheebha C, Gayatri Devi R, Jayaraj G. Knowledge and awareness of COVID 19 and its impact on mental health. International Journal of Research in Pharmaceutical Sciences. 2020;11:807-15.

903. Pretorius TL. Depression among health care students in the time of COVID-19: the mediating role of resilience in the hopelessness-depression relationship. S Afr J Psychol. 2021;51(2):269-78.

904. Prowse R, Sherratt F, Abizaid A, Gabrys RL, Hellemans KGC, Patterson ZR, et al. Coping With the COVID-19 Pandemic: Examining Gender Differences in Stress and Mental Health Among University Students. Frontiers in Psychiatry. 2021;12:650759.

905. Pruitt LD, McIntosh LS, Reger G. Suicide Safety Planning During a Pandemic: The Implications of COVID-19 on Coping with a Crisis. Suicide Life Threat Behav. 2020;50(3):741-9.

906. Puccinelli PJ, Costa TS, Seffrin A, de Lira CAB, Vancini RL, Knechtle B, et al. Physical Activity Levels and Mental Health during the COVID-19 Pandemic: Preliminary Results of a Comparative Study between Convenience Samples from Brazil and Switzerland. Medicina-Lithuania. 2021;57(1).

907. Puccinelli PJ, da Costa TS, Seffrin A, de Lira CAB, Vancini RL, Nikolaidis PT, et al. Reduced level of physical activity during COVID-19 pandemic is associated with depression and anxiety levels: an internet-based survey. Bmc Public Health. 2021;21(1):425.

908. Qanash S, Al-Husayni F, Alemam S, Alqublan L, Alwafi E, Mufti HN, et al. Psychological Effects on Health Science Students After Implementation of COVID-19 Quarantine and Distance Learning in Saudi Arabia. Cureus. 2020;12(11):e11767.

909. Qi H, Liu R, Chen X, Yuan XF, Li YQ, Huang HH, et al. Prevalence of anxiety and associated factors for Chinese adolescents during the COVID-19 outbreak. Psychiatry Clin Neurosci. 2020;74(10):555-7.

910. Qian M, Wu Q, Wu P, Hou Z, Liang Y, Cowling BJ, et al. Anxiety levels, precautionary behaviours and public perceptions during the early phase of the COVID-19 outbreak in China: a population-based cross-sectional survey. Bmj Open. 2020;10(10):e040910.

911. Qin F, Song Y, Nassis GP, Zhao L, Dong Y, Zhao C, et al. Physical Activity, Screen Time, and Emotional Well-Being during the 2019 Novel Coronavirus Outbreak in China. Int J Environ Res Public Health. 2020;17(14).

912. Quintiliani L, Sisto A, Vicinanza F, Curcio G, Tambone V. Resilience and psychological impact on Italian university students during COVID-19 pandemic. Distance learning and health. Psychol Health Med. 2022;27(1):69-80.

913. Quittkat HL, Dusing R, Holtmann FJ, Buhlmann U, Svaldi J, Vocks S. Perceived Impact of Covid-19 Across Different Mental Disorders: A Study on Disorder-Specific Symptoms, Psychosocial Stress and Behavior. Front Psychol. 2020;11:586246.

914. Radchikova NP, Odintsova MA. Assessment of the COVID-19 pandemic situation: Data from two countries with different security measures taken by authorities (Belarus and Russia). Data Brief. 2021;35:106917.

915. Rahali K, Abidli Z, Khohmimidi A, Elhamzaoui M, Seghiri R, Jabari K, et al. Ibn Tofail'sUniversity students' satisfaction evaluation towards distance learning and its impacts on the students' mental health during the Covid 19 Confinement. Bangladesh J Med Sci. 2020;19(19):S51-S7.

916. Rahman ME, Al Zubayer A, Bhuiyan MRA, Jobe MC, Khan MKA. Suicidal behaviors and suicide risk among Bangladeshi people during the COVID-19 pandemic: An online cross-sectional survey. Heliyon. 2021;7(2).

917. Rahman MM, Khan SJ, Sakib MS, Chakma S, Procheta NF, Al Mamun Z, et al. Assessing the psychological condition among general people of Bangladesh during COVID-19 pandemic. Journal of Human Behavior in the Social Environment. 2021;31(1-4):449-63.

918. Rainford LA, Zanardo M, Buissink C, Decoster R, Hennessy W, Knapp K, et al. The impact of COVID-19 upon student radiographers and clinical training. Radiography. 2021;27(2):464-74.

919. Rajab MH, Gazal AM, Alkattan K. Challenges to Online Medical Education During the COVID-19 Pandemic. Cureus. 2020;12(7):e8966.

920. Rajkumar RP. Sleep, physical activity and mental health during the COVID-19 pandemic: complexities and opportunities for intervention. Sleep Med. 2021;77:307-8.

921. Rakhmanov O, Dane S. Knowledge and Anxiety Levels of African University Students Against COVID-19 During the Pandemic Outbreak by an Online Survey. J Res Med Dent Sci. 2020;8(3):53-6.

922. Ramírez-Coronel AA, Cárdenas-Castillo PF, Martínez-Suárez PC, Yambay-Bautista XR, Mesa-Cano IC, Minchala-Urgilés RE, et al. Psychological impact of covid-19 confinement towards a new anxiety-depressive clinimetric construct in adult women of azogues. Archivos Venezolanos de Farmacologia y Terapeutica. 2020;39(8):923-34.

923. Ramos-Vera C. The dynamic network relationships of obsession and death from COVID-19 anxiety among Peruvian university students during the second quarantine. Rev Colomb Psiquiatr (Engl Ed). 2021.

924. Ramyarathidevi M, Mathiazhakan U, Tamilselvi S, Priya V, Arifa Bee S, Kanniammal C. Assessment of level of academic stress and stress coping style on impact of lockdown COVID 19 among nursing students at SRM College of Nursing, Chengalpattu (DT). International Journal of Research in Pharmaceutical Sciences. 2020;11:1156-62.

925. Ran LY, Wang W, Ai M, Kong YT, Chen JM, Kuang L. Psychological resilience, depression, anxiety, and somatization symptoms in response to COVID-19: A study of the general population in China at the peak of its epidemic. Soc Sci Med. 2020;262.

926. Ran MS, Gao R, Lin JX, Zhang TM, Chan SKW, Deng XP, et al. The impacts of COVID-19 outbreak on mental health in general population in different areas in China. Psychol Med. 2020:1-10.

927. Rania N, Coppola I. Psychological Impact of the Lockdown in Italy Due to the COVID-19 Outbreak: Are There Gender Differences? Front Psychol. 2021;12:567470.

928. Rath A, Wong M, Wong N, Brockman R. Use of a mindfulness application to promote students' mental well-being during COVID-19-era. J Dent Educ. 2021;85:2049-51.

929. Rathod S, Pallikadavath S, Young AH, Graves L, Rahman MM, Brooks A, et al. Psychological impact of COVID-19 pandemic: Protocol and results of first three weeks from an international cross-section survey - focus on health professionals. J Affect Disord Rep. 2020;1:100005.

930. Raza SH, Haq W, Sajjad M. COVID-19: A Psychosocial Perspective. Front Psychol. 2020;11:554624.

931. Reddy P, Nagi R, Kumar P, Srivastava R, Singh Bhadauriya U. Assessment of knowledge and anxiety levels due to COVID-19 pandemic among health care professionals and general population in Indore City: A cross sectional study. Przegl Epidemiol. 2020;74(3):441-8.

932. Rehman U, Shahnawaz MG, Khan NH, Kharshiing KD, Khursheed M, Gupta K, et al. Depression, Anxiety and Stress Among Indians in Times of Covid-19 Lockdown. Community Ment Hlt J. 2021;57(1):42-8.

933. Ren Y, Qian W, Li Z, Liu Z, Zhou Y, Wang R, et al. Public mental health under the long-term influence of COVID-19 in China: Geographical and temporal distribution. J Affect Disord. 2020;277:893-900.

934. Ren Z, Xin Y, Ge J, Zhao Z, Liu D, Ho RCM, et al. Psychological Impact of COVID-19 on College Students After School Reopening: A Cross-Sectional Study Based on Machine Learning. Front Psychol. 2021;12:641806.

935. Ren Z, Zhou Y, Liu Y. The psychological burden experienced by Chinese citizens during the COVID-19 outbreak: prevalence and determinants. Bmc Public Health. 2020;20(1):1617.

936. Restar AJ, Jin H, Jarrett B, Adamson T, Baral SD, Howell S, et al. Characterising the impact of COVID-19 environment on mental health, gender affirming services and socioeconomic loss in a global sample of transgender and non-binary people: a structural equation modelling. BMJ Glob Health. 2021;6(3).

937. Rettew DC, McGinnis EW, Copel, W, Nardone HY, Bai Y, et al. Personality trait predictors of adjustment during the COVID pandemic among college students. Plos One. 2021;16(3):e0248895.

938. Revathishree K, Shyam Sudhakar S, Indu R, Srinivasan K. Covid-19 Demographics from a Tertiary Care Center: Does It Depreciate Quality-of-Life? Indian Journal of Otolaryngology and Head and Neck Surgery. 2020.

939. Reverté-Villarroya S, Ortega L, Lavedán A, Masot O, Burjalés-Martí MD, Ballester-Ferr, et al. The influence of COVID-19 on the mental health of final-year nursing students: comparing the situation before and during the pandemic. Int J Ment Health Nurs. 2021;30(3):694-702.

940. Ripley-Gonzalez JW, Zhou N, Li Q, Chen M, Hu Z, Zhang W, et al. The association between prior physical fitness and depression in young adults during the COVID-19 pandemic-a crosssectional, retrospective study. Peerj. 2021;9:11091.

941. Ripon RK, El-Sabban F, Sikder T, Hossain S, Mim SS, Ahmed HU, et al. Psychological and nutritional effects on a COVID-19-quarantined population in Bangladesh. Journal of Human Behavior in the Social Environment. 2021;31(1):271-82.

942. Ritvo P, Ahmad F, El Morr C, Pirbaglou M, Moineddin R. A Mindfulness-Based Intervention for Student Depression, Anxiety, and Stress: Randomized Controlled Trial. JMIR Ment Health. 2021;8(1):e23491.

943. Robbins R, Affouf M, Weaver MD, Czeisler M, Barger LK, Quan SF, et al. Estimated Sleep Duration Before and During the COVID-19 Pandemic in Major Metropolitan Areas on Different Continents: Observational Study of Smartphone App Data. J Med Internet Res. 2021;23(2):e20546.

944. Roca J, Canet-Velez O, Cemeli T, Lavedan A, Masot O, Botigue T. Experiences, emotional responses, and coping skills of nursing students as auxiliary health workers during the peak COVID-19 pandemic: A qualitative study. Int J Ment Health Nu. 2021;30(5):1080-92.

945. Rodriguez S, Valle A, Pineiro I, Rodriguez-Llorente C, Guerrero E, Martins L. Sociodemographic Characteristics and Stress of People from Spain Confined by COVID-19. Eur J Investig Health Psychol Educ. 2020;10(4):1095-105.

946. Rodriguez-Besteiro S, Tornero-Aguilera JF, Fernandez-Lucas J, Clemente-Suarez VJ. Gender Differences in the COVID-19 Pandemic Risk Perception, Psychology, and Behaviors of Spanish University Students. Int J Environ Res Public Health. 2021;18(8).

947. Rodriguez-Hidalgo AJ, Pantaleon Y, Dios I, Falla D. Fear of COVID-19, Stress, and Anxiety in University Undergraduate Students: A Predictive Model for Depression. Front Psychol. 2020;11:591797.

948. Rodriguez-Rey R, Garrido-Hernansaiz H, Collado S. Psychological impact of COVID-19 in Spain: Early data report. Psychol Trauma. 2020;12(5):550-2.

949. Rodríguez-Rey R, Garrido-Hernansaiz H, Collado S. Psychological Impact and Associated Factors During the Initial Stage of the Coronavirus (COVID-19) Pandemic Among the General Population in Spain. Front Psychol. 2020;11:1540.

950. Rogowska A, M. r, Pavlova I, Bodnar I, Kusnierz C, Ochnik D, et al. Does physical activity matter for the mental health of university students during the COVID-19 pandemic? Journal of Clinical Medicine. 2020;9(11):1-19.

951. Rogowska AM, Kusnierz C, Bokszczanin A. Examining Anxiety, Life Satisfaction, General Health, Stress and Coping Styles During COVID-19 Pandemic in Polish Sample of University Students. Psychol Res Behav Manag. 2020;13:797-811.

952. Roitblat Y, Burger J, Leit A, Nehuliaieva L, Umarova GS, Kaliberdenko V, et al. Stay-at-home circumstances do not produce sleep disorders: An international survey during the COVID-19 pandemic. J Psychosom Res. 2020;139:110282.

953. Roitblat Y, Burger J, Vaiman M, Nehuliaieva L, Buchris N, Shterenshis M. Owls and larks do not exist: COVID-19 quarantine sleep habits. Sleep Med. 2021;77:177-83.

954. Roman M, Plopeanu AP. The effectiveness of the emergency eLearning during COVID-19 pandemic. The case of higher education in economics in Romania. Int Rev Econ Educ. 2021;37.

955. Romeo A, Benfante A, Castelli L, Di Tella M. Psychological Distress among Italian University Students Compared to General Workers during the COVID-19 Pandemic. International Journal of Environmental Research and Public Health. 2021;18(5).

956. Romero-Blanco C, Rodriguez-Almagro J, Onieva-Zafra MD, Parra-Fernandez ML, Prado-Laguna MD, Hernandez-Martinez A. Physical Activity and Sedentary Lifestyle in University Students: Changes during Confinement Due to the COVID-19 Pandemic. International Journal of Environmental Research and Public Health. 2020;17(18).

957. Romero-Blanco C, Rodriguez-Almagro J, Onieva-Zafra MD, Parra-Fernandez ML, Prado-Laguna MDC, Hernandez-Martinez A. Sleep Pattern Changes in Nursing Students during the COVID-19 Lockdown. Int J Environ Res Public Health. 2020;17(14).

958. Rosario Sinta Gamonal L, Enrique Montero M, Juan Matias F, ez, Carlos R. Anxiety, worry and perceived stress in the world due to the COVID-19 pandemic, March 2020. Preliminary results. 2020.

959. Rosenthal L, Lee S, Jenkins P, Arbet J, Carrington S, Hoon S, et al. A Survey of Mental Health in Graduate Nursing Students during the COVID-19 Pandemic. Nurse Educ. 2021;46(4):215-20.

960. Rosset M, Baumann E, Altenmuller E. Studying Music During the Coronavirus Pandemic: Conditions of Studying and Health-Related Challenges. Front Psychol. 2021;12:651393.

961. Rossinot H, Fantin R, Venne J. Behavioral Changes During COVID-19 Confinement in France: A Web-Based Study. Int J Environ Res Public Health. 2020;17(22).

962. Rotonda C, Brennstuhl MJ, Eby E, Tarquinio C. Impacts of COVID-19 on population well-being: Results of a web survey conducted in France during the first quarantine in 2020. Eur J Trauma Dissoc. 2021;5(2):100218-.

963. Roy VK, Arora J, Kaur N, hi A. Effect of the present situation of coronavirus disease-2019 pandemic on the academic and personal life of undergraduate medical and paramedical students. National Journal of Physiology, Pharmacy and Pharmacology. 2020;10(9):795-8.

964. Ru J, Holly K, Holly B, Jessica C, Chris D, Jonathan B, et al. Experiences of the COVID-19 pandemic: cross-sectional analysis of risk perceptions and mental health in a student cohort. 2020.

965. Rudenstine S, McNeal K, Schulder T, Ettman CK, Hernandez M, Gvozdieva K, et al. Depression and Anxiety During the COVID-19 Pandemic in an Urban, Low-Income Public University Sample. J Trauma Stress. 2021;34(1):12-22.

966. Saadeh H, Saadeh M, Almobaideen W, Al Refaei A, Shewaikani N, Al Fayez RQ, et al. Effect of COVID-19 Quarantine on the Sleep Quality and the Depressive Symptom Levels of University Students in Jordan During the Spring of 2020. Front Psychiatry. 2021;12:605676.

967. Saddik B, Hussein A, Albanna A, Elbarazi I, Al-Shujairi A, Temsah MH, et al. The psychological impact of the COVID-19 pandemic on adults and children in the United Arab Emirates: a nationwide cross-sectional study. Bmc Psychiatry. 2021;21(1):224.

968. Saddik B, Hussein A, Sharif-Askari FS, Kheder W, Temsah MH, Koutaich RA, et al. Increased Levels of Anxiety Among Medical and Non-Medical University Students During the COVID-19 Pandemic in the United Arab Emirates. Risk Manag Healthc P. 2020;13:2395-406.

969. Sadeghzadeh M, Abbasi M, Khajavi Y, Amirazodi H. Psychological correlates of anxiety in response to COVID-19 outbreak among Iranian University students. Curr Psychol. 2021:1-10.

970. Saez-Delgado F, Olea-Gonzalez C, Mella-Norambuena J, Lopez-Angulo Y, Garcia-Vasquez H, Cobo-Rendon R, et al. Psychosocial Characterization and Mental Health in Families of Chilean Students during Physical Isolation by Covid-19. Rev Int Educ Justici. 2020;9(3):281-300.

971. Safa F, Anjum A, Hossain S, Trisa TI, Alam SF, Rafi MA, et al. Immediate psychological responses during the initial period of the COVID-19 pandemic among Bangladeshi medical students. Child Youth Serv Rev. 2021;122.

972. Saguem BN, Nakhli J, Romdhane I, Nasr SB. Predictors of sleep quality in medical students during COVID-19 confinement. Encephale. 2022;48(1):3-12.

973. Sahin CU, Aydin M, Kulakac N. Psychological impact of COVID-19 pandemic on university students: Turkey sample. Pakistan Journal of Medical and Health Sciences. 2020;14(3):1215-20.

974. Saita E, Facchin F, Pagnini F, Molgora S. In the Eye of the Covid-19 Storm: A Web-Based Survey of Psychological Distress Among People Living in Lombardy. Frontiers in Psychology. 2021;12.

975. Salehian MH, Hemayattalab A, Ghanati P. Comparison of the effect of resilience training and Qigong exercises on the perceived stress due to Corona Disease in students. Pakistan Journal of Medical & Health Sciences. 2021;15(2):611-7.

976. Salehian MH, Yadolazadeh A, Ranjbari S. Comparison of the effect of cognitive-spiritual method of hope therapy and tai chi exercises on anxiety caused by corona disease in university students. Pakistan Journal of Medical & Health Sciences. 2021;15(3):938-47.

977. Salerno JP, Shrader C-H, Algarin AB, Lee J-Y, Fish JN. Changes in alcohol use since the onset of COVID-19 are associated with psychological distress among sexual and gender minority university students in the US. Drug and Alcohol Dependence. 2021;221.

978. Salfi F, D'Atri A, Tempesta D, Ferrara M. Sleeping under the waves: A longitudinal study across the contagion peaks of the COVID-19 pandemic in Italy. J Sleep Res. 2021;30(5):e13313.

979. Sallam M, Dababseh D, Yaseen A, Al-Haidar A, Ababneh NA, Bakri FG, et al. Conspiracy Beliefs Are Associated with Lower Knowledge and Higher Anxiety Levels Regarding COVID-19 among Students at the University of Jordan. International Journal of Environmental Research and Public Health. 2020;17(14).

980. Salman M, Asif N, Ul Mustafa Z, Khan TM, Shehzadi N, Tahir H, et al. Psychological Impairment and Coping Strategies During the COVID-19 Pandemic Among Students in Pakistan: A Cross-Sectional Analysis. Disaster Med Public. 2022;16(3):920-6.

981. Salzman J, Williamson M, Epsina-Rey A, Kibble J, Kauffman C. Effects of voluntary attendance patterns on first-year medical students' wellness and academic performance during COVID-19. Adv Physiol Educ. 2021;45(3):634-43.

982. Saravanan C, Mahmoud I, Elshami W, Taha MH. Knowledge, Anxiety, Fear, and Psychological Distress About COVID-19 Among University Students in the United Arab Emirates. Frontiers in Psychiatry. 2020;11.

983. Saravanan K, Hariharan S, Karthikeyan R, Prasanth BK, Nagarathinam S. Depression and anxiety among students community during COVID-19 pandemic lockdown in Tamil nadu- A web based descriptive cross sectional study. European Journal of Molecular and Clinical Medicine. 2021;8(3):1076-80.

984. Saravia Bartra MM, Cazorla Saravia PCRL. Nivel de ansiedad de estudiantes de medicina de primer año de una universidad privada del Perú en tiempos de Covid-19. Rev Fac Med Hum. 2020;20(4):568-73.

985. Sathe S, Thodge K, Joshi A. Prevalence of depression in college students during covid-19 pandemic. Indian Journal of Forensic Medicine and Toxicology. 2020;14(4):6831-5.

986. Satici B, Saricali M, Satici SA, Griffiths MD. Intolerance of Uncertainty and Mental Wellbeing: Serial Mediation by Rumination and Fear of COVID-19. Int J Ment Health Addict. 2020:1-12.

987. Savage MJ, Hennis PJ, Magistro D, Donaldson J, Healy LC, James RM. Nine Months into the COVID-19 Pandemic: A Longitudinal Study Showing Mental Health and Movement Behaviours Are Impaired in UK Students. International Journal of Environmental Research and Public Health. 2021;18(6).

988. Savage MJ, James R, Magistro D, Donaldson J, Healy LC, Nevill M, et al. Mental health and movement behaviour during the COVID-19 pandemic in UK university students: Prospective cohort study. Ment Health Phys Act. 2020;19.

989. Savarese G, Curcio L, D'Elia D, Fasano O, Pecoraro N. Online University Counselling Services and Psychological Problems among Italian Students in Lockdown Due to Covid-19. Healthcare-Basel. 2020;8(4):440-.

990. Savitsky B, Findling Y, Ereli A, Hendel T. Anxiety and coping strategies among nursing students during the covid-19 pandemic. Nurse Educ Pract. 2020;46:102809.

991. Savitsky B, Findling Y, Ereli A, Hendel T. Nursing Students in Crisis Mode: Fluctuations in Anxiety During the COVID-19-Related Lockdown. Nurse Educ. 2021;46(3):E33-E8.

992. Sayeed A, Kundu S, Al Banna MH, Hasan MT, Begum MR, Khan MSI. Mental health outcomes during the COVID-19 and perceptions towards the pandemic: Findings from a cross sectional study among Bangladeshi students. Child Youth Serv Rev. 2020;119.

993. Sazakli E, Leotsinidis M, Bakola M, Kitsou KS, Katsifara A, Konstantopoulou A, et al. Prevalence and associated factors of anxiety and depression in students at a Greek university during COVID-19 lockdown. Journal of Public Health Research. 2021;10(3).

994. Schafer SK, Sopp MR, Schanz CG, Staginnus M, Goritz AS, Michael T. Impact of COVID-19 on Public Mental Health and the Buffering Effect of a Sense of Coherence. Psychother Psychosom. 2020;89(6):386-92.

995. Scharmer C, Martinez K, Gorrell S, Reilly EE, Donahue JM, Anderson DA. Eating disorder pathology and compulsive exercise during the COVID-19 public health emergency: Examining risk associated with COVID-19 anxiety and intolerance of uncertainty. Int J Eat Disord. 2020;53(12):2049-54.

996. Schiff M, Zasiekina L, Pat-Horenczyk R, Benbenishty R. COVID-Related Functional Difficulties and Concerns Among University Students During COVID-19 Pandemic: A Binational Perspective. J Commun Health. 2021;46(4):667-75.

997. Schlenz MA, Schmidt A, Wostmann B, May A, Howaldt HP, Albert D, et al. Perspectives from Dentists, Dental Assistants, Students, and Patients on Dental Care Adapted to the COVID-19 Pandemic: A Cross-Sectional Survey. Int J Environ Res Public Health. 2021;18(8).

998. Schlichtiger J, Brunner S, Steffen J, Huber BC. Mental health impairment triggered by the COVID-19 pandemic in a sample population of German students. J Investig Med. 2020;68(8):1394-6.

999. Schmits E, Glowacz F. Changes in Alcohol Use During the COVID-19 Pandemic: Impact of the Lockdown Conditions and Mental Health Factors. Int J Ment Health Ad. 2022;20(2):1147-58.

1000. Schmitt AA, Brenner AM, Alves LPD, Claudino FCD, Fleck MPD, Rocha NS. Potential predictors of depressive symptoms during the initial stage of the COVID-19 outbreak among Brazilian adults. Journal of Affective Disorders. 2021;282:1090-5.

1001. Schweda A, Weismuller B, Bauerle A, Dorrie N, Musche V, Fink M, et al. Phenotyping mental health: Age, community size, and depression differently modulate COVID-19-related fear and generalized anxiety. Compr Psychiatry. 2021;104:152218.

1002. Scotta AV, Cortez MV, Miranda AR. Insomnia is associated with worry, cognitive avoidance and low academic engagement in Argentinian university students during the COVID-19 social isolation. Psychol Health Med. 2022;27(1):199-214.

1003. Sebri V, Cincidda C, Savioni L, Ongaro G, Pravettoni G. Worry during the initial height of the COVID-19 crisis in an Italian sample. J Gen Psychol. 2021;148(3):327-59.

1004. Sengul H, Bulut A, Coskun SN. Psychological effect of COVID-19 pandemic on university students in Turkey. Ann Clin Anal Med. 2020;11(6):609-15.

1005. Şengün G, Toptaş V. Determination of the University Students’ Opinions About Coronavirus (covid-19) Global Outbreak. Milli Egitim. 2020;49(1):1011-31.

1006. Serafim AP, Duraes RSS, Rocca CCA, Goncalves PD, Saffi F, Cappellozza A, et al. Exploratory study on the psychological impact of COVID-19 on the general Brazilian population. Plos One. 2021;16(2):e0245868.

1007. Serin E, Koc MC. Examination of the eating behaviours and depression states of the university students who stay at home during the coronavirus pandemic in terms of different variables. Prog Nutr. 2020;22:33-43.

1008. Sethi D, Jabade M, Yadav P, Fern, es S, Anthony JW. Effect of lockdown on students studying in various colleges of nursing. Indian Journal of Forensic Medicine and Toxicology. 2020;14(4):3603-8.

1009. Seun AAAA, Uwadia aTG. Influence of COVID-19 on the Psychological Wellbeing of Tertiary Institution Students in Nigeria. Tanzania Journal of Science. 2021;47:70-9.

1010. Sfendla A, Hadrya F. Factors Associated with Psychological Distress and Physical Activity During the COVID-19 Pandemic. Health Secur. 2020;18(6):444-53.

1011. Shabahang R. Cognitive behavioural intervention for health anxiety, somatosensory amplification, and depression in coronavirus disease 2019 anxiety: an interventional study in Iran. Psychiatr Psychol Kl. 2020;20(2):87-93.

1012. Shabahang R, Aruguete MS, McCutcheon L. Video-based cognitive-behavioral intervention for COVID-19 anxiety: a randomized controlled trial. Trends Psychiatry Psychother. 2021;43(2):141-50.

1013. Shabahang R, Aruguete MS, McCutcheon LE. Online health information utilization and online news exposure as predictor of COVID-19 anxiety. North American Journal of Psychology. 2020;22(3):469-82.

1014. Shafiq S, Nipa SN, Sultana S, Rahman MR, Rahman MM. Exploring the triggering factors for mental stress of university students amid COVID-19 in Bangladesh: A perception-based study. Child Youth Serv Rev. 2021;120:105789.

1015. Shah SMA, Mohammad D, Qureshi MFH, Abbas MZ, Aleem S. Prevalence, Psychological Responses and Associated Correlates of Depression, Anxiety and Stress in a Global Population, During the Coronavirus Disease (COVID-19) Pandemic. Community Ment Health J. 2021;57(1):101-10.

1016. Shahbaz S, Ashraf MZ, Zakar R, Fischer F, Zakar MZ. Psychosocial effects of the COVID-19 pandemic and lockdown on university students: Understanding apprehensions through a phenomenographic approach. Plos One. 2021;16(5):e0251641.

1017. Shahriarirad R, Erfani A, Ranjbar K, Bazrafshan A, Mirahmadizadeh A. The mental health impact of COVID-19 outbreak: a Nationwide Survey in Iran. Int J Ment Health Syst. 2021;15(1):19.

1018. Shaikh S, Mohsin SF, Saleem Agwan MA, Ali S, Alsuwaydani ZA, AlSuwaydani SA. COVID-19: Fear and anxiety among healthcare students in Saudi Arabia: A cross-sectional study. European Journal of Molecular and Clinical Medicine. 2021;8(3):1638-47.

1019. Shailaja B, Singh H, Chaudhury S, Thyloth M. COVID-19 pandemic and its aftermath: Knowledge, attitude, behavior, and mental health-care needs of medical undergraduates. Ind Psychiatry J. 2020;29(1):51-60.

1020. Sharpe D, Rajabi M, Chileshe C, Joseph SM, Sesay I, Williams J, et al. Mental health and wellbeing implications of the COVID-19 quarantine for disabled and disadvantaged children and young people: evidence from a cross-cultural study in Zambia and Sierra Leone. BMC Psychol. 2021;9(1):79.

1021. Shinde R, Kaur J, Ramesh S. A study to assess anxiety and coping strategies adopted by the students regarding education during COVID-19 lockdown. Indian Journal of Forensic Medicine and Toxicology. 2020;14(4):3921-3.

1022. Shrestha C, Ghimire C, Acharya S, Kc P, Singh S, Sharma P. Mental Wellbeing during the Lockdown Period following the COVID-19 Pandemic in Nepal: A Descriptive Cross-sectional Study. JNMA J Nepal Med Assoc. 2020;58(230):744-50.

1023. Shrestha DB, Thapa BB, Katuwal N, Shrestha B, Pant C, Basnet B, et al. Psychological distress in Nepalese residents during COVID-19 pandemic: a community level survey. Bmc Psychiatry. 2020;20(1):491.

1024. Shrestha MV, Shrestha N, Sharma SC, Joshi SK. Gaming Disorder among Medical College Students during COVID-19 Pandemic Lockdown. Kathmandu Univ Med J (KUMJ). 2020;18(70):48-52.

1025. Shrivastava KJ, Nahar R, Parlani S, Murthy VJ. A cross-sectional virtual survey to evaluate the outcome of online dental education system among undergraduate dental students across India amid COVID-19 pandemic. Eur J Dent Educ. 2022;26(1):123-30.

1026. Shuwiekh HAM, Kira IA, Sous MSF, Ashby JS, Alhuwailah A, Baali SBA, et al. The differential mental health impact of COVID-19 in Arab countries. Curr Psychol. 2022;41(8):5678-92.

1027. Sifat RI. COVID-19 pandemic: Mental stress, depression, anxiety among the university students in Bangladesh. Int J Soc Psychiatr. 2021;67(5):609-10.

1028. Sigurvinsdottir R, Thorisdottir IE, Gylfason HF. The Impact of COVID-19 on Mental Health: The Role of Locus on Control and Internet Use. International Journal of Environmental Research and Public Health. 2020;17(19):1-15.

1029. Silva PGB, de Oliveira CAL, Borges MMF, Moreira DM, Alencar PNB, Avelar RL, et al. Distance learning during social seclusion by COVID-19: Improving the quality of life of undergraduate dentistry students. Eur J Dent Educ. 2021;25(1):124-34.

1030. Simegn W, Dagnew B, Yeshaw Y, Yitayih S, Woldegerima B, Dagne H. Depression, anxiety, stress and their associated factors among Ethiopian University students during an early stage of COVID-19 pandemic: An online-based cross-sectional survey. Plos One. 2021;16(5):e0251670.

1031. Simione L, Gnagnarella C. Differences Between Health Workers and General Population in Risk Perception, Behaviors, and Psychological Distress Related to COVID-19 Spread in Italy. Frontiers in Psychology. 2020;11.

1032. Simpson EMD. COVID-19 Restrictions, Emerging Adulthood and Higher Education in Kazakhstan – Mental Health Impacts on Students (preprint). 2020.

1033. Singh RMR. Mental health of young adults: A descriptive study. International Journal of Education and Management Studies. 2020;10(4):457-62.

1034. Sit SM, Lam TH, Lai AY, Wong BY, Wang MP, Ho SY. Fear of COVID-19 and its associations with perceived personal and family benefits and harms in Hong Kong. Transl Behav Med. 2021;11(3):793-801.

1035. Skalski S, Konaszewski K, Dobrakowski P, Surzykiewicz J, Lee SA. Pandemic grief in Poland: adaptation of a measure and its relationship with social support and resilience. Curr Psychol. 2022;41(10):7393-401.

1036. Skapinakis P, Bellos S, Oikonomou A, Dimitriadis G, Gkikas P, Perdikari E, et al. Depression and Its Relationship with Coping Strategies and Illness Perceptions during the COVID-19 Lockdown in Greece: A Cross-Sectional Survey of the Population. Depress Res Treat. 2020;2020:3158954.

1037. Slivkoff MD, Johnson C, Tackett S. First-Year Medical Student Experiences Adjusting to the Immediate Aftermath of COVID-19. Med Sci Educ. 2021;31(2):557-64.

1038. Šljivo A, Kaamakovi M, Quraishi I, Kulenovi AD. Fear and depression among residents of Bosnia and Herzegovina during covid-19 outbreak-Internet survey. Psychiatria Danubina. 2020;32(2):266-72.

1039. Smit AN, Juda M, Livingstone A, U SR, Mistlberger RE. Impact of COVID-19 social-distancing on sleep timing and duration during a university semester. Plos One. 2021;16(4):e0250793.

1040. Sögüt S, Dolu İ, Cangöl E. The relationship between COVID-19 knowledge levels and anxiety states of midwifery students during the outbreak: A cross-sectional web-based survey. Perspect Psychiatr Care. 2020;57(1):246-52.

1041. Sokolovsky AW, Hertel AW, Micalizzi L, White HR, Hayes KL, Jackson KM. Preliminary impact of the COVID-19 pandemic on smoking and vaping in college students. Addict Behav. 2021;115:106783.

1042. Solomou I, Constantinidou F. Prevalence and Predictors of Anxiety and Depression Symptoms during the COVID-19 Pandemic and Compliance with Precautionary Measures: Age and Sex Matter. Int J Environ Res Public Health. 2020;17(14):1-19.

1043. Somma A, Krueger RF, Markon KE, Gialdi G, Colanino M, Ferlito D, et al. A longitudinal study on clinically relevant self-reported depression, anxiety and acute stress features among Italian community-dwelling adults during the COVID-19 related lockdown: Evidence of a predictive role for baseline dysfunctional personality dimensions. Journal of Affective Disorders. 2021;282:364-71.

1044. Son C, Hegde S, Smith A, Wang X, Sasangohar F. Effects of COVID-19 on College Students' Mental Health in the United States: Interview Survey Study. J Med Internet Res. 2020;22(9):e21279.

1045. Song BJ, Zhao YL, Zhu JC. COVID-19-related Traumatic Effects and Psychological Reactions among International Students. J Epidemiol Glob Hea. 2021;11(1):117-23.

1046. Song HT, Ge CH, Chang LX, Zhao TT, Wu W, Ge DX, et al. Investigation on the psychological status of college students during the coronavirus disease-2019 epidemic. J Gen Psychol. 2022;149(4):456-67.

1047. Song S, Yao X, Wen N. What motivates Chinese consumers to avoid information about the COVID-19 pandemic?: The perspective of the stimulus-organism-response model. Information Processing and Management. 2021;58(1).

1048. Sood S, Sharma A. Resilience and Psychological Well-Being of Higher Education Students During COVID-19: The Mediating Role of Perceived Distress. J Health Manag. 2020;22(4):606-17.

1049. Soria KM, Horgos B. Factors Associated With College Students' Mental Health During the COVID-19 Pandemic. J Coll Student Dev. 2021;62(2):236-42.

1050. Spano G, D'Este M, Giannico V, Elia M, Cassibba R, Lafortezza R, et al. Association between indoor-outdoor green features and psychological health during the COVID-19 lockdown in Italy: A cross-sectional nationwide study. Urban for Urban Gree. 2021;62.

1051. Spasovski O, Kenig N. Psychological Well-Being in Students during Self-Isolation Due to the Covid-19 Pandemic. Primenj Psihol. 2020;13(4):427-47.

1052. Sreeram M, Mundada MA. Survey to assess perceived causes of stress, its manifestations and coping strategies among dental college students in maharashtra during COVID-19 lockdown. Biomedical and Pharmacology Journal. 2021;14(1):53-60.

1053. Srivastava S, Jacob J, Charles AS, Daniel P, Mathew JK, Shanthi P, et al. Emergency remote learning in anatomy during the COVID-19 pandemic: A study evaluating academic factors contributing to anxiety among first year medical students. Med J Armed Forces India. 2021;77:S90-s8.

1054. Stafford O, Berry A, Taylor LK, Wearen S, Prendergast C, Murphy E, et al. Comorbidity and COVID-19: investigating the relationship between medical and psychological well-being. Irish J Psychol Med. 2021;38(4):272-7.

1055. Steinmetz LCL, Leyes CA, Florio MAD, Fong SB, Steinmetz RLL, Godoy JC. Mental Health Impacts in Argentinean College Students During COVID-19 Quarantine. Frontiers in Psychiatry. 2021;12:557880.

1056. Steward M, Moses M, Chiluba M, Zikria S, Aubrey Chichoni K, Derick M, et al. Impact of the Coronavirus Disease (COVID-19) on the Mental Health and Physical Activity of Pharmacy Students at the University of Zambia: A Cross-Sectional Study. 2021.

1057. Stickley A, Matsubayashi T, Sueki H, Ueda M. COVID-19 preventive behaviours among people with anxiety and depressive symptoms: findings from Japan. Public Health. 2020;189:91-3.

1058. Stockman JK, Wood BA, Anderson KM. Racial and Ethnic Differences in COVID-19 Outcomes, Stressors, Fear, and Prevention Behaviors among U.S. Women: Web-based Cross-sectional Study. J Med Internet Res. 2021.

1059. Sturgill R, Martinasek M, Schmidt T, Goyal R. A Novel Artificial Intelligence-Powered Emotional Intelligence and Mindfulness App (Ajivar) for the College Student Population During the COVID-19 Pandemic: Quantitative Questionnaire Study. JMIR Form Res. 2021;5(1):e25372.

1060. Su F, Fan B, Song N, Dong X, Wang Y, Li J, et al. Survey on Public Psychological Intervention Demand and Influence Factors Analysis. Int J Environ Res Public Health. 2021;18(9).

1061. Suen YT, Chan RCH, Wong EMY. Effects of general and sexual minority-specific COVID-19-related stressors on the mental health of lesbian, gay, and bisexual people in Hong Kong. Psychiatry Res. 2020;292:113365.

1062. Suhail A, Dar KA, Iqbal N. COVID-19 related fear and mental health in Indian sample: The buffering effect of support system. Curr Psychol. 2022;41(1):480-91.

1063. Suhail A, Iqbal N, Smith J. Lived experiences of Indian Youth amid COVID-19 crisis: An interpretative phenomenological analysis. Int J Soc Psychiatry. 2021;67(5):559-66.

1064. Sujadi E, Kamil D, Ridha M, ri H, Juliawati D, Fadhli M, et al., editors. Does covid-19 significantly affect the quality of life? The impact analysis of covid-19 on work, financial, quality of worship, emotional and social aspects2020 2020.

1065. Suksatan W, Choompunuch B, Koontalay A, Posai V, Abusafia AH. Predictors of Health Behaviors Among Undergraduate Students During the COVID-19 Pandemic: A Cross-Sectional Predictive Study. J Multidiscip Health. 2021;14:727-34.

1066. Suliman WA, Abu-Moghli FA, Khalaf I, Zumot AF, Nabolsi M. Experiences of nursing students under the unprecedented abrupt online learning format forced by the national curfew due to COVID-19: A qualitative research study. Nurs Educ Today. 2021;100:104829.

1067. Sultana MS, Khan AH, Hossain S, Ansar A, Sikder MT, Hasan MT. Prevalence and predictors of post-traumatic stress symptoms and depressive symptoms among Bangladeshi students during COVID-19 mandatory home confinement: A nationwide cross-sectional survey. Child Youth Serv Rev. 2021;122.

1068. Sultana S, Shafique I, Majeed N, Jamshed S, Shahani AK, Qureshi F. Impact of Covid-19 outbreak on psychological health-The case of Bangladesh. Heliyon. 2021;7(4):e06772.

1069. Suman, Sidhu MS, Grover S, Dabas A, Dogra N, Jaglan A. Orthodontic practice in the times of COVID-19 pandamic: An online survey. Journal of Clinical and Diagnostic Research. 2021;15(3):ZC36-ZC44.

1070. Sumen A, Adibelli D. Nursing students' readiness and coping strategies for the Covid-19 pandemic in Turkey. J Prof Nurs. 2021;37(3):553-61.

1071. Sun L, Sun Z, Wu L, Zhu Z, Zhang F, Shang Z, et al. Prevalence and risk factors for acute posttraumatic stress disorder during the COVID-19 outbreak. J Affect Disord. 2021;283:123-9.

1072. Sun M, Wang D, Jing L, Zhou L. Changes in psychotic-like experiences and related influential factors in technical secondary school and college students during COVID-19. Schizophr Res. 2021;231:3-9.

1073. Sun S, Goldberg SB, Lin D, Qiao S, Operario D. Psychiatric symptoms, risk, and protective factors among university students in quarantine during the COVID-19 pandemic in China. Global Health. 2021;17(1):15.

1074. Sun Y, Li YY, Bao YP, Meng SQ, Sun YK, Schumann G, et al. Brief Report: Increased Addictive Internet and Substance Use Behavior During the COVID-19 Pandemic in China. Am J Addiction. 2020;29(4):268-70.

1075. Sun Y, Lin SY, Chung KKH. University Students' Perceived Peer Support and Experienced Depressive Symptoms during the COVID-19 Pandemic: The Mediating Role of Emotional Well-Being. Int J Environ Res Public Health. 2020;17(24).

1076. Sun Y, Wang D, Han Z, Gao J, Zhu S, Zhang H. Disease Prevention Knowledge, Anxiety, and Professional Identity during COVID-19 Pandemic in Nursing Students in Zhengzhou, China. J Korean Acad Nurs. 2020;50(4):533-40.

1077. Sundarasamy VG, Thamizharasan R, Lalan RH. Psychological impact of COVID-19 on medical college students. European Journal of Molecular and Clinical Medicine. 2020;7(10):435-43.

1078. Sundarasen S, Chinna K, Kamaludin K, Nurunnabi M, Baloch GM, Khoshaim HB, et al. Psychological Impact of COVID-19 and Lockdown among University Students in Malaysia: Implications and Policy Recommendations. International Journal of Environmental Research and Public Health. 2020;17(17).

1079. Sweeny K, Rankin K, Cheng X, Hou L, Long F, Meng Y, et al. Flow in the time of COVID-19: Findings from China. Plos One. 2020;15(11):e0242043.

1080. Syam A, Thahir AIA, Iqbal M, Maharani SA. The emotional state and physical condition of indonesian college students: An emerging situation during the coronavirus disease-19 crisis in Indonesia. Open Access Macedonian Journal of Medical Sciences. 2020;8:261-7.

1081. Syed M, Akhter N, Ibrahim M, Stanley L. The Effects of COVID-19 Pandemic Lockdown on Medical Students' Academic Performance and Stress Levels Based on their Gender and Age. Faseb J. 2021;35.

1082. Szabo A, Abel K, Boros S. Attitudes Toward COVID-19 and Stress Levels in Hungary: Effects of Age, Perceived Health Status, and Gender. Psychol Trauma-Us. 2020;12(6):572-5.

1083. Szczypińska M, Samełko A, Guszkowska M. Strategies for Coping With Stress in Athletes During the COVID-19 Pandemic and Their Predictors. Front Psychol. 2021;12:624949.

1084. Szkody E, Stearns M, Stanhope L, McKinney C. Stress-Buffering Role of Social Support during COVID-19. Fam Process. 2021;60(3):1002-15.

1085. Szovak E, Varga K, Pelyva IZ, Soos R, Jeges S, Kives Z, et al. Insights Gained in the Aftermath of the COVID-19 Pandemic: A Follow-Up Survey of a Recreational Training Program, Focusing on Sense of Coherence and Sleep Quality. Int J Environ Res Public Health. 2020;17(24).

1086. Tabari P, Amini M. Educational and psychological support for medical students during the COVID-19 outbreak. Med Educ. 2021;55(1):125-7.

1087. Taha H, Saber Ahmed C, Mohammad Main U, Nazmul Ahsan K. Mental Health Status of Adolescents During the COVID-19 Pandemic: A Cross-sectional Survey among the Bangladeshi Graduate Students at Dhaka City. 2020.

1088. Tamayo Garcia MR, Miraval Tarazona ZE, Mansilla Natividad P. Trastornos De Las Emociones a Consecuencia Del Covid-19 Y El Confinamiento En Universitarios De Las Diferentes Escuelas De La Universidad Nacional Hermilio Valdizan. Peru. Rev Comun Salud. 2020;10(2):343-54.

1089. Tang ACY, Kwong EWY, Chen LY, Cheng WLS. Associations between demographic characteristics, perceived threat, perceived stress, coping responses and adherence to COVID-19 prevention measures among Chinese healthcare students. J Adv Nurs. 2021;77(9):3759-71.

1090. Tang W, Hu T, Hu B, Jin C, Wang G, Xie C, et al. Prevalence and correlates of PTSD and depressive symptoms one month after the outbreak of the COVID-19 epidemic in a sample of home-quarantined Chinese university students. J Affect Disord. 2020;274:1-7.

1091. Tang W, Hu T, Yang L, Xu J. The role of alexithymia in the mental health problems of home-quarantined university students during the COVID-19 pandemic in China. Pers Individ Dif. 2020;165:110131.

1092. Taquet M, Quoidbach J, Fried EI, Goodwin GM. Mood Homeostasis Before and During the Coronavirus Disease 2019 (COVID-19) Lockdown Among Students in the Netherlands. Jama Psychiatry. 2021;78(1):110-2.

1093. Tasnim R, Islam MS, Sujan MSH, Sikder MT, Potenza MN. Suicidal ideation among Bangladeshi university students early during the COVID-19 pandemic: Prevalence estimates and correlates. Child Youth Serv Rev. 2020;119:105703.

1094. Tasso AF, Hisli Sahin N, San Roman GJ. COVID-19 disruption on college students: Academic and socioemotional implications. Psychol Trauma. 2021;13(1):9-15.

1095. Tavolacci MP, Wouters E, Van de Velde S, Buffel V, Dechelotte P, Van Hal G, et al. The Impact of COVID-19 Lockdown on Health Behaviors among Students of a French University. International Journal of Environmental Research and Public Health. 2021;18(8).

1096. Tee M, Wang CY, Tee C, Pan RY, Reyes PW, Wan XY, et al. Impact of the COVID-19 Pandemic on Physical and Mental Health in Lower and Upper Middle-Income Asian Countries: A Comparison Between the Philippines and China. Frontiers in Psychiatry. 2021;11.

1097. Tee ML, Tee CA, Anlacan JP, Aligam KJG, Reyes PWC, Kuruchittham V, et al. Psychological impact of COVID-19 pandemic in the Philippines. J Affect Disord. 2020;277:379-91.

1098. Teixeira LdAC, Costa RA, de Mattos RMPR, Pimentel D. Brazilian medical students' mental health during coronavirus disease 2019 pandemic. Jornal Brasileiro de Psiquiatria. 2021;70(1):21-9.

1099. Tendolkar VD, Suraj S, Rajurkar H. Psychosocial impact of lock down among college students in Nagpur. Indian Journal of Forensic Medicine and Toxicology. 2020;14(4):6273-8.

1100. Teotonio I, Hecht M, Castro LC, Gandolfi L, Pratesi R, Nakano EY, et al. Repercussion of COVID-19 Pandemic on Brazilians' Quality of Life: A Nationwide Cross-Sectional Study. Int J Environ Res Public Health. 2020;17(22):1-11.

1101. Teresa MT, Guss CD, Boyd L. Thriving during COVID-19: Predictors of psychological well-being and ways of coping. Plos One. 2021;16(3).

1102. Thum DiCesare JA, Segar DJ, Donoho D, Radwanski R, Zada G, Yang I. Democratizing Access to Neurosurgical Medical Education: National Efforts in a Medical Student Training Camp During Coronavirus Disease 2019. World Neurosurg. 2020;144:e237-e43.

1103. Tian F, Li H, Tian S, Yang J, Shao J, Tian C. Psychological symptoms of ordinary Chinese citizens based on SCL-90 during the level I emergency response to COVID-19. Psychiatry Res. 2020;288:112992.

1104. Torun F, Torun SD. The psychological impact of the COVID-19 pandemic on medical students in Turkey. Pak J Med Sci. 2020;36(6):1355-9.

1105. Toure AA, Camara LM, Magassouba AS, Doumbouya A, Camara G, Camara AY, et al. Psychosocial impacts of COVID-19 in the Guinean population. An online cross-sectional survey. Plos One. 2021;16(2):e0245751.

1106. Trammell Ph DJ, Joseph Ph DN, Harriger Ph DJ. Racial and ethnic minority disparities in COVID-19 related health, health beliefs and behaviors, and well-being among students. J Am Coll Health. 2021:1-7.

1107. Tran TM, Provosty W, Fang Y, Weissbecker K, Myint M. Covid-19 Effects on Mental Health and Well-Being of Tulane University School of Medicine Students. J Invest Med. 2021;69(2):450-.

1108. Traunmuller C, Stefitz R, Gaisbachgrabner K, Schwerdtfeger A. Psychological correlates of COVID-19 pandemic in the Austrian population. Bmc Public Health. 2020;20(1):1395.

1109. Tsai J, Elbogen EB, Huang M, North CS, Pietrzak RH. Psychological distress and alcohol use disorder during the COVID-19 era among middle- and low-income U.S. adults. Journal of Affective Disorders. 2021;288:41-9.

1110. Tsai J, Huang M, Elbogen E. Mental Health and Psychosocial Characteristics Associated With COVID-19 Among U.S. Adults. Psychiatr Serv. 2021;72(4):444-7.

1111. Tsibidaki A. Anxiety, meaning in life, self-efficacy and resilience in families with one or more members with special educational needs and disability during COVID-19 pandemic in Greece. Res Dev Disabil. 2021;109:103830.

1112. Tukayo IJH, Maay JKR, Nugroho HSW, Sirait P. The stress level and its effect on learning achievements of health students due to corona pandemic in Indonesia. Systematic Reviews in Pharmacy. 2020;11(12):2375-9.

1113. Tuna O, Enez Darcin A, Tarakcioglu MC, Aksoy UM. COVID-19 Positive Psychiatry Inpatient Unit: A unique experience. Asia Pac Psychiatry. 2020;12(4):e12410.

1114. Twenge JM, Joiner TE. U.S. Census Bureau-assessed prevalence of anxiety and depressive symptoms in 2019 and during the 2020 COVID-19 pandemic. Depress Anxiety. 2020;37(10):954-6.

1115. Ueda M, Stickley A, Sueki H, Matsubayashi T. Mental health status of the general population in Japan during the COVID-19 pandemic. Psychiatry Clin Neurosci. 2020;74(9):505-6.

1116. Uluoz E. Opinions of the Faculty of Sport Sciences Students on the Changes in Education System during COVID-19 Pandemic: A Qualitative Research. African Educational Research Journal. 2020;8(3):481-90.

1117. Upadhyaya GK, Jain VK, Iyengar KP, Patralekh MK, Vaish A. Impact of COVID-19 on post-graduate orthopaedic training in Delhi-NCR. J Clin Orthop Trauma. 2020;11:S687-S95.

1118. Usher K, Wynaden D, Bhullar N, Durkin J, Jackson D. The mental health impact of COVID-19 on pre-registration nursing students in Australia. Int J Ment Health Nurs. 2020;29(6):1015-7.

1119. Ustun G. Determining depression and related factors in a society affected by COVID-19 pandemic. Int J Soc Psychiatry. 2021;67(1):54-63.

1120. Vahedian-Azimi A, Moayed MS, Rahimibashar F, Shojaei S, Ashtari S, Pourhoseingholi MA. Comparison of the severity of psychological distress among four groups of an Iranian population regarding COVID-19 pandemic. Bmc Psychiatry. 2020;20(1):402.

1121. Vahratian A, Blumberg SJ, Terlizzi EP, Schiller JS. Symptoms of Anxiety or Depressive Disorder and Use of Mental Health Care Among Adults During the COVID-19 Pandemic - United States, August 2020-February 2021. Mmwr-Morbid Mortal W. 2021;70(13):490-4.

1122. Vala NH, Vachhani MV, Sorani AM. Study of anxiety, stress, and depression level among medical students during covid-19 pandemic phase in jamnagar city. National Journal of Physiology, Pharmacy and Pharmacology. 2020;10(12):1043-5.

1123. Valekar SS, Phaphe SA, Sarode KR. Mental Status of Dental Students during Lockdown Due to Covid-19 Pandemic - A Cross Sectional Study in Western Maharashtra. J Evol Med Dent Sci-. 2021;10(8):532-5.

1124. Vally Z. Public perceptions, anxiety and the perceived efficacy of health-protective behaviours to mitigate the spread of the SARS-Cov-2/COVID-19 pandemic. Public Health. 2020;187:67-73.

1125. van Dam L, Rhodes J, Spencer R. Youth-Initiated Mentoring as a Scalable Approach to Addressing Mental Health Problems During the COVID-19 Crisis. JAMA Psychiatry. 2021.

1126. Van Der Feltz-Cornelis CM, Varley D, Allgar VL, de Beurs E. Workplace Stress, Presenteeism, Absenteeism, and Resilience Amongst University Staff and Students in the COVID-19 Lockdown. Front Psychiatry. 2020;11:588803.

1127. van der Velden PG, Contino C, Das M, van Loon P, Bosmans MWG. Anxiety and depression symptoms, and lack of emotional support among the general population before and during the COVID-19 pandemic A prospective national study on prevalence and risk factors. Journal of Affective Disorders. 2020;277:540-8.

1128. van Zyl LE. Social Study Resources and Social Wellbeing Before and During the Intelligent COVID-19 Lockdown in The Netherlands. Soc Indic Res. 2021:1-23.

1129. van Zyl LE, Rothmann S, Zondervan-Zwijnenburg MAJ. Longitudinal Trajectories of Study Characteristics and Mental Health Before and During the COVID-19 Lockdown. Front Psychol. 2021;12:633533.

1130. Vanderbruggen N, Matthys F, Van Laere S, Zeeuws D, Santermans L, Van den Ameele S, et al. Self-reported alcohol, tobacco, and cannabis use during COVID-19 lockdown measures: results from a web-based survey. European Addiction Research. 2020;26(6):309-15.

1131. Vera-Ponce VJ, Valladares-Garrido MJ, Peralta CI, Astudillo D, Torres-Malca JR, Orihuela-Manrique EJ, et al. Factors associated with psychological coping with covid-19 during quarantine period. Revista Cubana de Medicina Militar. 2020;49(4):1-17.

1132. Verma K. The mental health impact of the COVID-19 epidemic on college students in India. Asian J Psychiatr. 2020;53:102398.

1133. Vermote B, Waterschoot J, Morbée S, Van der Kaap-Deeder J, Schrooyen C, Soenens B, et al. Do Psychological Needs Play a Role in Times of Uncertainty? Associations with Well-Being During the COVID-19 Crisis. J Happiness Stud. 2022:1-27.

1134. Vidas D, Larwood JL, Nelson NL, Dingle GA. Music Listening as a Strategy for Managing COVID-19 Stress in First-Year University Students. Frontiers in Psychology. 2021;12:647065.

1135. Vidyadhara S, Chakravarthy A, Pramod Kumar A, Sri Harsha C, Rahul R. Mental Health Status among the South Indian Pharmacy Students during Covid-19 Pandemic Quarantine Period: A Cross-Sectional Study. 2020.

1136. Vigo D, Jones L, Munthali R, Pei J, Westenberg J, Munro L, et al. Investigating the effect of COVID-19 dissemination on symptoms of anxiety and depression among university students. Bjpsych Open. 2021;7(2):e69.

1137. Villani L, Pastorino R, Molinari E, Anelli F, Ricciardi W, Graffigna G, et al. Impact of the COVID-19 pandemic on psychological well-being of students in an Italian university: a web-based cross-sectional survey. Global Health. 2021;17(1):39.

1138. Virginia C, Ines R, Ricardo G, Henrique B. Depression and anxiety before and during the COVID-19 lockdown: a longitudinal cohort study with university students. 2021.

1139. Visser M, Law-van Wyk E. University students' mental health and emotional wellbeing during the COVID-19 pandemic and ensuing lockdown. S Afr J Psychol. 2021;51(2):229-43.

1140. Vitale E, Moretti B, Noternicola A, Covelli I. How the Italian Nursing students deal the pandemic Covid-19 condition. Acta Biomed. 2020;91(12):e2020007.

1141. Voitsidis P, Nikopoulou VA, Holeva V, Parlapani E, Sereslis K, Tsipropoulou V, et al. The mediating role of fear of COVID-19 in the relationship between intolerance of uncertainty and depression. Psychol Psychother-T. 2021;94(3):884-93.

1142. Volk AA, Brazil KJ, Franklin-Luther P, Dane AV, Vaillancourt T. The influence of demographics and personality on COVID-19 coping in young adults. Pers Individ Dif. 2021;168:110398.

1143. Volken T, Zysset A, Amendola S, Klein Swormink A, Huber M, von Wyl A, et al. Depressive Symptoms in Swiss University Students during the COVID-19 Pandemic and Its Correlates. Int J Environ Res Public Health. 2021;18(4).

1144. Vrublevska J, Sibalova A, Aleskere I, Rezgale B, Smirnova D, Fountoulakis KN, et al. Factors related to depression, distress, and self-reported changes in anxiety, depression, and suicidal thoughts during the COVID-19 state of emergency in Latvia. Nord J Psychiatry. 2021;75(8):614-23.

1145. Vujcic I, Safiye T, Milikic B, Popovic E, Dubljanin D, Dubljanin E, et al. Coronavirus Disease 2019 (COVID-19) Epidemic and Mental Health Status in the General Adult Population of Serbia: A Cross-Sectional Study. Int J Environ Res Public Health. 2021;18(4).

1146. Vukotic M, Krivokapic D, Bubanja M, Zarubica M, Redzepagic S. The psychological impact of preexisting mental and physical health during the COVID-19 pandemic. Nutr Hosp. 2021;38(2):426.

1147. Wahyuni S, Cahyati Y, Rosdiana I. The relationship between Self-efficacy and student anxiety in facing final projects during the Covid-19 pandemic. European Journal of Molecular and Clinical Medicine. 2021;8(2):2282-94.

1148. Wallace S, Schuler MS, Kaulback M, Hunt K, Baker M. Nursing student experiences of remote learning during the COVID-19 pandemic. Nurs Forum. 2021;56(3):612-8.

1149. Wanberg CR, Csillag B, Douglass RP, Zhou L, Pollard MS. Socioeconomic status and well-being during COVID-19: A resource-based examination. J Appl Psychol. 2020;105(12):1382-96.

1150. Wang C, Pan R, Wan X, Tan Y, Xu L, McIntyre RS, et al. A longitudinal study on the mental health of general population during the COVID-19 epidemic in China. Brain Behav Immun. 2020;87:40-8.

1151. Wang C, Tee M, Roy AE, Fardin MA, Srichokchatchawan W, Habib HA, et al. The impact of COVID-19 pandemic on physical and mental health of Asians: A study of seven middle-income countries in Asia. Plos One. 2021;16(2):e0246824.

1152. Wang C, Zhao H. The Impact of COVID-19 on Anxiety in Chinese University Students. Front Psychol. 2020;11:1168.

1153. Wang CM, Song WD, Hu XH, Yan SG, Zhang X, Wang XQ, et al. Depressive, anxiety, and insomnia symptoms between population in quarantine and general population during the COVID-19 pandemic: a case-controlled study. Bmc Psychiatry. 2021;21(1):99.

1154. Wang CY, Chudzicka-Czupala A, Grabowski D, Pan RY, Adamus K, Wan XY, et al. The Association Between Physical and Mental Health and Face Mask Use During the COVID-19 Pandemic: A Comparison of Two Countries With Different Views and Practices. Frontiers in Psychiatry. 2020;11.

1155. Wang CY, Pan RY, Wan XY, Tan YL, Xu LK, Ho CS, et al. Immediate Psychological Responses and Associated Factors during the Initial Stage of the 2019 Coronavirus Disease (COVID-19) Epidemic among the General Population in China. International Journal of Environmental Research and Public Health. 2020;17(5).

1156. Wang CY, Zhao H, Zhang HR. Chinese College Students Have Higher Anxiety in New Semester of Online Learning During COVID-19: A Machine Learning Approach. Frontiers in Psychology. 2020;11:587413.

1157. Wang D, Chen H, Zhai S, Zhu Z, Huang S, Zhou X, et al. Is returning to school during the COVID-19 pandemic stressful? A study on immediate mental health status of Chinese college students. J Affect Disord. 2021;287:261-7.

1158. Wang H, Xia Q, Xiong Z, Li Z, Xiang W, Yuan Y, et al. The psychological distress and coping styles in the early stages of the 2019 coronavirus disease (COVID-19) epidemic in the general mainland Chinese population: A web-based survey. Plos One. 2020;15(5):e0233410.

1159. Wang HF, Jia LQ. Research on the countermeasures of college students' mental health education under the background of the epidemic. Int J Elec Eng Educ. 2021:00207209211005261-.

1160. Wang J, Gong YH, Chen ZY, Wu JX, Feng J, Yan SJ, et al. Sleep disturbances among Chinese residents during the Coronavirus Disease 2019 outbreak and associated factors. Sleep Medicine. 2020;74:199-203.

1161. Wang J, Liu WC, Zhang YM, Xie SQ, Yang B. Perceived Stress Among Chinese Medical Students Engaging in Online Learning in Light of COVID-19. Psychology Research and Behavior Management. 2021;14:549-62.

1162. Wang P, Wang R, Tian M, Sun Y, Ma J, Tu Y, et al. The Pathways from Type A Personality to Physical and Mental Health Amid COVID-19: A Multiple-Group Path Model of Frontline Anti-Epidemic Medical Staff and Ordinary People. Int J Environ Res Public Health. 2021;18(4).

1163. Wang S, Feng K, Zhang Y, Liu J, Wang W, Li Y. Antecedents of Public Mental Health During the COVID-19 Pandemic: Mediation of Pandemic-Related Knowledge and Self-Efficacy and Moderation of Risk Level. Front Psychiatry. 2020;11:567119.

1164. Wang T, Yang Y, Na L, editors. "overrated Anxiety" - - Impact of Corona Virus Disease 2019 Epidemic on Anxiety Levels of Chinese Population2021 2021.

1165. Wang X, Chen H, Liu L, Liu Y, Zhang N, Sun Z, et al. Anxiety and Sleep Problems of College Students During the Outbreak of COVID-19. Front Psychiatry. 2020;11:588693.

1166. Wang X, Hegde S, Son C, Keller B, Smith A, Sasangohar F. Investigating Mental Health of US College Students During the COVID-19 Pandemic: Cross-Sectional Survey Study. J Med Internet Res. 2020;22(9):e22817.

1167. Wang X, Zhang R, Wang Z, Li T. How Does Digital Competence Preserve University Students' Psychological Well-Being During the Pandemic? An Investigation From Self-Determined Theory. Front Psychol. 2021;12:652594.

1168. Wang Y, Jiang L, Ma S, Chen Q, Liu C, Ahmed F, et al. Media Exposure Related to the PTSS During COVID-19 Pandemic: The Mediating Role of Risk Perception. Front Psychiatry. 2021;12:654548.

1169. Wang Y, Jing X, Han W, Jing Y, Xu L. Positive and negative affect of university and college students during COVID-19 outbreak: a network-based survey. Int J Public Health. 2020;65(8):1437-43.

1170. Wang Y, Li YC, Jiang JW, Feng YY, Lu DH, Zhang W, et al. COVID-19 outbreak-related psychological distress among healthcare trainees: a cross-sectional study in China. Bmj Open. 2020;10(10):e041671.

1171. Wang YH, Shi L, Que JY, Lu QD, Liu L, Lu ZA, et al. The impact of quarantine on mental health status among general population in China during the COVID-19 pandemic. Mol Psychiatr. 2021;26(9):4813-22.

1172. Wang YN, Di Y, Ye JJ, Wei WB. Study on the public psychological states and its related factors during the outbreak of coronavirus disease 2019 (COVID-19) in some regions of China. Psychol Health Med. 2021;26(1):13-22.

1173. Wang ZH, Yang HL, Yang YQ, Liu D, Li ZH, Zhang XR, et al. Prevalence of anxiety and depression symptom, and the demands for psychological knowledge and interventions in college students during COVID-19 epidemic: A large cross-sectional study. J Affect Disord. 2020;275:188-93.

1174. Waqas M, Hania A, Hongbo L. Psychological Predictors of Anxious Responses to the COVID-19 Pandemic: Evidence from Pakistan. Psychiatry Investig. 2020;17(11):1096-104.

1175. Warren AM, Zolfaghari K, Fresnedo M, Bennett M, Pogue J, Waddimba A, et al. Anxiety sensitivity, COVID-19 fear, and mental health: results from a United States population sample. Cogn Behav Ther. 2021;50(3):204-16.

1176. Warrier U, John M, Warrier S. Leveraging Emotional Intelligence Competencies for Sustainable Development of Higher Education Institutions in the New Normal. Fiib Bus Rev. 2021;10(1):62-73.

1177. Wasil AR, Franzen RE, Gillespie S, Steinberg JS, Malhotra T, DeRubeis RJ. Commonly Reported Problems and Coping Strategies During the COVID-19 Crisis: A Survey of Graduate and Professional Students. Front Psychol. 2021;12:598557.

1178. Wasil AR, Taylor ME, Franzen RE, Steinberg JS, DeRubeis RJ. Promoting Graduate Student Mental Health During COVID-19: Acceptability, Feasibility, and Perceived Utility of an Online Single-Session Intervention. Front Psychol. 2021;12:569785.

1179. Wathelet M, Duhem S, Vaiva G, Baubet T, Habran E, Veerapa E, et al. Factors Associated With Mental Health Disorders Among University Students in France Confined During the COVID-19 Pandemic. Jama Netw Open. 2020;3(10):e2025591.

1180. Wathelet M, Fovet T, Jousset A, Duhem S, Habran E, Horn M, et al. Prevalence of and factors associated with post-traumatic stress disorder among French university students 1 month after the COVID-19 lockdown. Transl Psychiat. 2021;11(1):327.

1181. Weis R, Ray SD, Cohen TA. Mindfulness as a way to cope with covid‐19‐related stress and anxiety. Counselling & Psychotherapy Research. 2020.

1182. Wen FF, Zhu JL, Ye HX, Li LY, Ma Z, Wen XX, et al. Associations between insecurity and stress among Chinese university students: The mediating effects of hope and self-efficacy. Journal of Affective Disorders. 2021;281:447-53.

1183. Werneck AO, Silva DR, Malta DC, Lima MG, Souza PRB, Azevedo LO, et al. The mediation role of sleep quality in the association between the incidence of unhealthy movement behaviors during the COVID-19 quarantine and mental health. Sleep Medicine. 2020;76:10-5.

1184. Werneck AO, Silva DR, Malta DC, Souza PRB, Azevedo LO, Barros MBA, et al. Changes in the clustering of unhealthy movement behaviors during the COVID-19 quarantine and the association with mental health indicators among Brazilian adults. Transl Behav Med. 2021;11(2):323-31.

1185. Werneck AO, Silva DR, Malta DC, Souza-Junior PRB, Azevedo LO, Barros MBA, et al. Physical inactivity and elevated TV-viewing reported changes during the COVID-19 pandemic are associated with mental health: A survey with 43,995 Brazilian adults. J Psychosom Res. 2021;140:110292.

1186. Wickens CM, McDonald AJ, Elton-Marshall T, Wells S, Nigatu YT, Jankowicz D, et al. Loneliness in the COVID-19 pandemic: Associations with age, gender and their interaction. J Psychiatr Res. 2021;136:103-8.

1187. Widiyanto A, Atmojo JT, ayani RT, Fajriah AS, Kurniavie LE. The effect of social media exposure on depression and anxiety disorders in facing Covid-19 pandemic. European Journal of Molecular and Clinical Medicine. 2020;7(2):4635-43.

1188. Wieczorek T, Kolodziejczyk A, Ciulkowicz M, Maciaszek J, Misiak B, Rymaszewska J, et al. Class of 2020 in Poland: Students' Mental Health during the COVID-19 Outbreak in an Academic Setting. Int J Environ Res Public Health. 2021;18(6).

1189. Wielgus B, Urban W, Patriak A, Cichocki L. Examining the Associations between Psychological Flexibility, Mindfulness, Psychosomatic Functioning, and Anxiety during the COVID-19 Pandemic: A Path Analysis. International Journal of Environmental Research and Public Health. 2020;17(23):1-13.

1190. Wilczewski M, Gorbaniuk O, Giuri P. The Psychological and Academic Effects of Studying From the Home and Host Country During the COVID-19 Pandemic. Front Psychol. 2021;12:644096.

1191. Williams B, King C, Shannon B, Gosling C. Impact of COVID-19 on paramedicine students: A mixed methods study. International Emergency Nursing. 2021;56:100996.

1192. Williams E, Pallerla H, McDonald S, Malosh L, Doshi S, Cotton S. Evaluating 'creating caring communities': A medical student pilot wellness program to aid in the transition to medical school during COVID-19. Global Advances in Health and Medicine. 2021;10:30-1.

1193. Wilson JJ, McMullan I, Blackburn NE, Klempel N, Yakkundi A, Armstrong NC, et al. Changes in dietary fat intake and associations with mental health in a UK public sample during the COVID-19 pandemic. J Public Health (Oxf). 2021;43(4):687-94.

1194. Wilson OWA, Holland KE, Elliott LD, Duffey M, Bopp M. The Impact of the COVID-19 Pandemic on US College Students' Physical Activity and Mental Health. J Phys Act Health. 2021;18(3):272-8.

1195. Winkler P, Mohrova Z, Mlada K, Kuklova M, Kagstrom A, Mohr P, et al. Prevalence of current mental disorders before and during the second wave of COVID-19 pandemic: An analysis of repeated nationwide cross-sectional surveys. J Psychiatr Res. 2021;139:167-71.

1196. Wise J. Covid-19: Suicidal thoughts increased in young adults during lockdown, UK study finds. BMJ. 2020;371:m4095.

1197. Wong BYM, Lam TH, Lai AYK, Wang MP, Ho SY. Perceived Benefits and Harms of the COVID-19 Pandemic on Family Well-Being and Their Sociodemographic Disparities in Hong Kong: A Cross-Sectional Study. International Journal of Environmental Research and Public Health. 2021;18(3).

1198. Wong LP, Alias H. Temporal changes in psychobehavioural responses during the early phase of the COVID-19 pandemic in Malaysia. J Behav Med. 2021;44(1):18-28.

1199. Wong LP, Alias H, Md Fuzi AA, Omar IS, Mohamad Nor A, Tan MP, et al. Escalating progression of mental health disorders during the COVID-19 pandemic: Evidence from a nationwide survey. Plos One. 2021;16(3):e0248916.

1200. Wong LP, Hung CC, Alias H, Lee TS. Anxiety symptoms and preventive measures during the COVID-19 outbreak in Taiwan. Bmc Psychiatry. 2020;20(1):376.

1201. Woon LSC, Bin Abdullah MFIL, Sidi H, Mansor NS, Jaafar NRN. Depression, anxiety, and the COVID-19 pandemic: Severity of symptoms and associated factors among university students after the end of the movement lockdown. Plos One. 2021;16(5):e0252481.

1202. Wu A, Maddula V, Yu ACX, Goel R, Shimizu H, Chien CL, et al. An Observation of Healthcare Professions Students' Perceptions During the COVID-19 Pandemic. Med Sci Educ. 2021;31(2):401-9.

1203. Wu M, Han H, Lin T, Chen M, Wu J, Du X, et al. Prevalence and risk factors of mental distress in China during the outbreak of COVID-19: A national cross-sectional survey. Brain Behav. 2020;10(11):e01818.

1204. Wu S, Li Z, Li Z, Xiang W, Yuan Y, Liu Y, et al. The mental state and risk factors of Chinese medical staff and medical students in early stages of the COVID-19 epidemic. Compr Psychiatry. 2020;102:152202.

1205. Wu W, Zhang Y, Wang P, Zhang L, Wang G, Lei G, et al. Psychological stress of medical staffs during outbreak of COVID-19 and adjustment strategy. J Med Virol. 2020;92(10):1962-70.

1206. Wu XY, Tao SM, Zhang Y, Li SY, Ma L, Yu YZ, et al. Geographic Distribution of Mental Health Problems Among Chinese College Students During the COVID-19 Pandemic: Nationwide, Web-Based Survey Study. J Med Internet Res. 2021;23(1):e23126.

1207. Xiang MQ, Tan XM, Sun J, Yang HY, Zhao XP, Liu L, et al. Relationship of Physical Activity With Anxiety and Depression Symptoms in Chinese College Students During the COVID-19 Outbreak. Front Psychol. 2020;11:582436.

1208. Xiao H, Shu W, Li M, Li Z, Tao F, Wu X, et al. Social Distancing among Medical Students during the 2019 Coronavirus Disease Pandemic in China: Disease Awareness, Anxiety Disorder, Depression, and Behavioral Activities. Int J Environ Res Public Health. 2020;17(14).

1209. Xie J, Li X, Luo H, He L, Bai Y, Zheng F, et al. Depressive Symptoms, Sleep Quality and Diet During the 2019 Novel Coronavirus Epidemic in China: A Survey of Medical Students. Front Public Health. 2020;8:588578.

1210. Xie J, Luo SX, Furuya K, Sun DJ. Urban Parks as Green Buffers During the COVID-19 Pandemic. Sustainability. 2020;12(17).

1211. Xie L, Luo H, Li M, Ge W, Xing B, Miao Q. The immediate psychological effects of Coronavirus Disease 2019 on medical and non-medical students in China. Int J Public Health. 2020;65(8):1445-53.

1212. Xin M, Luo S, She R, Yu Y, Li L, Wang S, et al. Negative cognitive and psychological correlates of mandatory quarantine during the initial COVID-19 outbreak in China. Am Psychol. 2020;75(5):607-17.

1213. Xu W, Xiang L, Proverbs D, Xiong S. The Influence of COVID-19 on Community Disaster Resilience. Int J Environ Res Public Health. 2020;18(1).

1214. Xu YH, Shao JL, Zeng W, Wu XR, Huang DT, Zeng YQ, et al. Depression and Creativity During COVID-19: Psychological Resilience as a Mediator and Deliberate Rumination as a Moderator. Frontiers in Psychology. 2021;12:665961.

1215. Xu Z, Du J. A mental health informatics study on the mediating effect of the regulatory emotional self-efficacy. Math Biosci Eng. 2021;18(3):2775-88.

1216. Yadav RK, Baral S, Khatri E, Pandey S, Pandeya P, Neupane R, et al. Anxiety and Depression Among Health Sciences Students in Home Quarantine During the COVID-19 Pandemic in Selected Provinces of Nepal. Front Public Health. 2021;9:580561.

1217. Yalcin I, Can N, Mance Calisir O, Yalcin S, Colak B. Latent profile analysis of COVID-19 fear, depression, anxiety, stress, mindfulness, and resilience. Curr Psychol. 2022;41(1):459-69.

1218. Yan LL, Gan YQ, Ding X, Wu JH, Duan HX. The relationship between perceived stress and emotional distress during the COVID-19 outbreak: Effects of boredom proneness and coping style. Journal of Anxiety Disorders. 2021;77.

1219. Yan SYJ. Analysis and Countermeasures of College Students&#039; Mental Health based on the Novel Coronavirus Pneumonia Epidemic Background. International Journal of Social Science and Education Research. 2020;3(11):54-9.

1220. Yang C, Chen A, Chen Y. College students' stress and health in the COVID-19 pandemic: The role of academic workload, separation from school, and fears of contagion. Plos One. 2021;16(2):e0246676.

1221. Yang D, Tu CC, Dai X. The effect of the 2019 novel coronavirus pandemic on college students in Wuhan. Psychol Trauma. 2020;12(S1):S6-S14.

1222. Yang F. Coping strategies, cyberbullying behaviors, and depression among Chinese netizens during the COVID-19 pandemic: a web-based nationwide survey. Journal of Affective Disorders. 2021;281:138-44.

1223. Yang H, Bin P, He AJ. Opinions from the epicenter: an online survey of university students in Wuhan amidst the COVID-19 outbreak11. Journal of Chinese Governance. 2020;5(2):234-48.

1224. Yang HJ, Chen ZH, Fan YS, Hu XH, Wu T, Kang ST, et al. Knowledge, attitudes and anxiety toward COVID-19 among domestic and overseas Chinese college students. J Public Health-Uk. 2021;43(3):466-71.

1225. Yang KH, Wang L, Liu H, Li LX, Jiang XL. Impact of coronavirus disease 2019 on the mental health of university students in Sichuan Province, China: An online cross-sectional study. Int J Ment Health Nurs. 2021;30(4):875-84.

1226. Yang M, He P, Xu XM, Li D, Wang J, Wang YJ, et al. Disrupted rhythms of life, work and entertainment and their associations with psychological impacts under the stress of the COVID-19 pandemic: A survey in 5854 Chinese people with different sociodemographic backgrounds. Plos One. 2021;16(5):e0250770.

1227. Yang X, Hu HH, Zhao CJ, Xu HH, Tu XL, Zhang GH. A longitudinal study of changes in smart phone addiction and depressive symptoms and potential risk factors among Chinese college students. Bmc Psychiatry. 2021;21(1):252.

1228. Yang XY, Peng S, Yang T, Cottrell RR. Changing trends of mental and behavioral responses and associations during the COVID-19 epidemic in China: a panel study. Health Educ Res. 2021;36(2):151-8.

1229. Yang Y, Xiao YN, Liu YL, Li Q, Shan CS, Chang SL, et al. Mental Health and Psychological Impact on Students with or without Hearing Loss during the Recurrence of the COVID-19 Pandemic in China. International Journal of Environmental Research and Public Health. 2021;18(4).

1230. Ye B, Wu D, Im H, Liu M, Wang X, Yang Q. Stressors of COVID-19 and stress consequences: The mediating role of rumination and the moderating role of psychological support. Child Youth Serv Rev. 2020;118:105466.

1231. Ye B, Zhou X, Im H, Liu M, Wang XQ, Yang Q. Epidemic Rumination and Resilience on College Students' Depressive Symptoms During the COVID-19 Pandemic: The Mediating Role of Fatigue. Front Public Health. 2020;8:560983.

1232. Ye W, Ye X, Liu Y, Liu Q, Vafaei S, Gao Y, et al. Effect of the Novel Coronavirus Pneumonia Pandemic on Medical Students' Psychological Stress and Its Influencing Factors. Front Psychol. 2020;11:548506.

1233. Ye Z, Yang XY, Zeng CB, Wang YY, Shen ZJ, Li XM, et al. Resilience, Social Support, and Coping as Mediators between COVID-19-related Stressful Experiences and Acute Stress Disorder among College Students in China. Appl Psychol-Hlth We. 2020;12(4):1074-94.

1234. Yehudai M, Bender S, Gritsenko V, Konstantinov V, Reznik A, Isralowitz R. COVID-19 Fear, Mental Health, and Substance Misuse Conditions Among University Social Work Students in Israel and Russia. Int J Ment Health Ad. 2022;20(1):316-23.

1235. Yildirim M, Akgul O, Gecer E. The Effect of COVID-19 Anxiety on General Health: the Role of COVID-19 Coping. Int J Ment Health Addict. 2022;20(2):1110-21.

1236. Yildirim M, Gecer E, Akgul O. The impacts of vulnerability, perceived risk, and fear on preventive behaviours against COVID-19. Psychol Health Med. 2021;26(1):35-43.

1237. Yildirim M, Guler A. COVID-19 severity, self-efficacy, knowledge, preventive behaviors, and mental health in Turkey. Death Stud. 2022;46(4):979-86.

1238. Yildirim TT, Atas O. The evaluation of psychological state of dental students during the COVID-19 pandemic. Braz Oral Res. 2021;35:e069.

1239. Yildiz E. Posttraumatic growth and positive determinants in nursing students after COVID-19 alarm status: A descriptive cross-sectional study. Perspectives in Psychiatric Care. 2021;57(4):1876-87.

1240. Yıldırım M, Arslan G. Exploring the associations between resilience, dispositional hope, preventive behaviours, subjective well-being, and psychological health among adults during early stage of COVID-19. Curr Psychol. 2020:1-11.

1241. Yorguner N, Bulut NS, Akvardar Y. An Analysis of the Psychosocial Challenges Faced by the University Students During COVID-19 Pandemic, and the Students' Knowledge, Attitudes, and Practices Toward the Disease. Noropsikiyatri Ars. 2021;58(1):3-10.

1242. Yoshito N, Kanako O, Kazuki T, Mikako O, Hideharu H, Hitomi K, et al. Socio-educational Impact and Psychological Distress of Medical Students amid the COVID-19 Pandemic: A Japanese Cross-Sectional Survey. 2020.

1243. Young NA, Waugh CE, Minton AR, Charles ST, Haase CM, Mikels JA. Reactive, Agentic, Apathetic, or Challenged? Aging, Emotion, and Coping During the COVID-19 Pandemic. Gerontologist. 2021;61(2):217-27.

1244. Younis I, Longsheng C, Zulfiqar MI, Imran M, Shah SAA, Hussain M, et al. Regional disparities in Preventive measures of COVID-19 pandemic in China. A study from international students' prior knowledge, perception and vulnerabilities. Environ Sci Pollut Res Int. 2021;28(30):40355-70.

1245. Yu A, Wilkes M, Iosif AM, Rea M, Fisher A, Fine J, et al. Exploring the Relationships Between Resilience and News Monitoring with COVID Distress in Health Profession Students. Acad Psychiatr. 2021;45(5):566-74.

1246. Yu H, Li M, Li Z, Xiang W, Yuan Y, Liu Y, et al. Coping style, social support and psychological distress in the general Chinese population in the early stages of the COVID-19 epidemic. Bmc Psychiatry. 2020;20(1):426.

1247. Yu ML, Tian FQ, Cui Q, Wu H. Prevalence and its associated factors of depressive symptoms among Chinese college students during the COVID-19 pandemic. Bmc Psychiatry. 2021;21(1):66.

1248. Yu YJ, Yu YJ, Lin YG. Cross-lagged analysis of the interplay between meaning in life and positive mental health during the COVID-19 epidemic. Asian Journal of Psychiatry. 2020;54:102278.

1249. Yu YQ, She R, Luo ST, Xin MQ, Li LJ, Wang SH, et al. Factors Influencing Depression and Mental Distress Related to COVID-19 Among University Students in China: Online Cross-sectional Mediation Study. Jmir Ment Health. 2021;8(2):e22705.

1250. Yuan S, Liao Z, Huang H, Jiang B, Zhang X, Wang Y, et al. Comparison of the Indicators of Psychological Stress in the Population of Hubei Province and Non-Endemic Provinces in China During Two Weeks During the Coronavirus Disease 2019 (COVID-19) Outbreak in February 2020. Med Sci Monit. 2020;26:e923767.

1251. Yuan Y. Mindfulness training on the resilience of adolescents under the COVID-19 epidemic: A latent growth curve analysis. Pers Individ Dif. 2021;172:110560.

1252. Yuhua F, Xueting J, Xinoxino U, Yinli S, Linlin Z, Jinghao W. The role of mental problem evaluation and intervention in university or college students kept at home due lo serious Corona Virus Disease-2019 epidemic during high education. Pharmaceutical Care and Research. 2020;20(2):81.

1253. Yuksel D, McKee GB, Perrin PB, Alzueta E, Caffarra S, Ramos-Usuga D, et al. Sleeping when the world locks down: Correlates of sleep health during the COVID-19 pandemic across 59 countries. Sleep Health. 2021;7(2):134-42.

1254. Zabini F, Albanese L, Becheri FR, Gavazzi G, Giganti F, Giovanelli F, et al. Comparative Study of the Restorative Effects of Forest and Urban Videos during COVID-19 Lockdown: Intrinsic and Benchmark Values. Int J Environ Res Public Health. 2020;17(21).

1255. Zain MZMZRMHHANHZDMHHSWRA. DISTANCE EDUCATION: THE CHALLENGES AND EFFECTS OF THE COVID-19 PANDEMIC AMONG FASHION STUDENTS IN MALAYSIA. PalArch&#039;s Journal of Archaeology of Egypt/Egyptology. 2021;18(6):110-25.

1256. Zamanian M, Ahmadi D, Sindarreh S, Aleebrahim F, Vardanjani HM, Faghihi SH, et al. Fear and rumor associated with COVID-19 among Iranian adults, 2020. J Educ Health Promot. 2020;9(1).

1257. Zambelli Z, Fidalgo AR, Halstead EJ, Dimitriou D. Acute impact of a national lockdown during the COVID-19 pandemic on wellbeing outcomes among individuals with chronic pain. J Health Psychol. 2022;27(5):1099-110.

1258. Zarzecka J, Zarzecka-Francica E, Gala A, Gebczynski K, Pihut M. Dental Environmental Stress during the Covid-19 Pandemic at the Jagiellonian University Medical College, Krakow, Poland. Int J Occup Med Env. 2021;34(2):211-22.

1259. Zawadka J, Miekisz A, Nowakowska I, Plewko J, Kochanska M, Haman E. Remote learning among students with and without reading difficulties during the initial stages of the COVID-19 pandemic. Educ Inf Technol. 2021;26(6):6973-94.

1260. Zhang BY, Zaman A, Silenzio V, Kautz H, Hoque E. The Relationships of Deteriorating Depression and Anxiety With Longitudinal Behavioral Changes in Google and YouTube Use During COVID-19: Observational Study. Jmir Ment Health. 2020;7(11):e24012.

1261. Zhang K, Peng Y, Zhang X, Li L. Psychological Burden and Experiences Following Exposure to COVID-19: A Qualitative and Quantitative Study of Chinese Medical Student Volunteers. Int J Environ Res Public Health. 2021;18(8).

1262. Zhang L, Chen MA, Yao BX, Zhang Y. Aggression and Non-Suicidal Self-Injury among Depressed Youths: The Mediating Effect of Resilience. Iran J Public Health. 2021;50(2):288-96.

1263. Zhang L, Yao B, Zhang X, Xu H. Effects of Irritability of the Youth on Subjective Well-Being: Mediating Effect of Coping Styles. Iran J Public Health. 2020;49(10):1848-56.

1264. Zhang LP, Qi HB, Wang LQ, Wang FL, Huang J, Li FF, et al. Effects of the COVID-19 pandemic on acute stress disorder and career planning among healthcare students. Int J Ment Health Nu. 2021;30(4):907-16.

1265. Zhang WY, Yang XT, Zhao JF, Yang FZ, Jia YJ, Cui C, et al. Depression and Psychological-Behavioral Responses Among the General Public in China During the Early Stages of the COVID-19 Pandemic: Survey Study. J Med Internet Res. 2020;22(9).

1266. Zhang X, Li X, Liao Z, Zhao M, Zhuang Q. Evaluation of psychological stress in scientific researchers during the 2019-2020 COVID-19 outbreak in China. Peerj. 2020;8:e9497.

1267. Zhang Y, Cao XC, Aashiq, Xie YF, Zhong QY, Lei GH, et al. Psychological stress of university students in the hardest-hit areas at different stages of the COVID-19 epidemic. Child Youth Serv Rev. 2021;125:105980.

1268. Zhang Y, Cao XC, Wang P, Wang GX, Lei GH, Shou ZX, et al. Emotional "inflection point" in public health emergencies with the 2019 new coronavirus pneumonia (NCP) in China. Journal of Affective Disorders. 2020;276:797-803.

1269. Zhang Y, Chen YP, Wang J, Deng Y, Peng D, Zhao L. Anxiety Status and Influencing Factors of Rural Residents in Hunan During the Coronavirus Disease 2019 Epidemic: A Web-Based Cross-Sectional Survey. Front Psychiatry. 2020;11:564745.

1270. Zhang Y, Zhang HY, Ma XD, Di Q. Mental Health Problems during the COVID-19 Pandemics and the Mitigation Effects of Exercise: A Longitudinal Study of College Students in China. International Journal of Environmental Research and Public Health. 2020;17(10).

1271. Zhang YF, Ma ZF. Impact of the COVID-19 Pandemic on Mental Health and Quality of Life among Local Residents in Liaoning Province, China: A Cross-Sectional Study. International Journal of Environmental Research and Public Health. 2020;17(7).

1272. Zhang ZY, Feng Y, Song R, Yang D, Duan XF. Prevalence of psychiatric diagnosis and related psychopathological symptoms among patients with COVID-19 during the second wave of the pandemic. Globalization Health. 2021;17(1).

1273. Zhao B, Kong FL, Aung MN, Yuasa M, Nam EW. Novel Coronavirus (COVID-19) Knowledge, Precaution Practice, and Associated Depression Symptoms among University Students in Korea, China, and Japan. International Journal of Environmental Research and Public Health. 2020;17(18):6671-.

1274. Zhao B, Kong FL, Nam EW. Assessing Knowledge, Preventive Practices, and Depression among Chinese University Students in Korea and China during the COVID-19 Pandemic: An Online Cross-Sectional Study. Healthcare-Basel. 2021;9(4).

1275. Zhao H, Xiong J, Zhang Z, Qi C. Growth Mindset and College Students' Learning Engagement During the COVID-19 Pandemic: A Serial Mediation Model. Front Psychol. 2021;12:621094.

1276. Zhao L, Sznajder K, Cheng D, Wang S, Cui C, Yang X. Coping Styles Mediates the Effect of Resilience on Medical Students' Depression in the Context of Online Classes during the COVID-19 Pandemic. J Med Internet Res. 2021.

1277. Zhao N, Zhou GY. Social Media Use and Mental Health during the COVID-19 Pandemic: Moderator Role of Disaster Stressor and Mediator Role of Negative Affect. Appl Psychol-Hlth We. 2020;12(4):1019-38.

1278. Zhao N, Zhou GY. COVID-19 Stress and Addictive Social Media Use (SMU): Mediating Role of Active Use and Social Media Flow. Frontiers in Psychiatry. 2021;12:635546.

1279. Zhao SZ, Wong JYH, Luk TT, Wai AKC, Lam TH, Wang MP. Mental health crisis under COVID-19 pandemic in Hong Kong, China. Int J Infect Dis. 2020;100:431-3.

1280. Zhao XL, Lan MX, Li HX, Yang J. Perceived stress and sleep quality among the non-diseased general public in China during the 2019 coronavirus disease: a moderated mediation model. Sleep Medicine. 2021;77:339-45.

1281. Zhao Y, Zhou Q, Li J, Luan J, Wang B, Zhao Y, et al. Influence of psychological stress and coping styles in the professional identity of undergraduate nursing students after the outbreak of COVID-19: A cross-sectional study in China. Nurs Open. 2021;8(6):3527-37.

1282. Zhao YM, Jiang ZD, Guo SH, Wu P, Lu QD, Xu YY, et al. Association of Symptoms of Attention Deficit and Hyperactivity with Problematic Internet Use among University Students in Wuhan, China During the COVID-19 Pandemic. Journal of Affective Disorders. 2021;286:220-7.

1283. Zhao YQ, An YY, Tan X, Li XH. Mental Health and Its Influencing Factors among Self-Isolating Ordinary Citizens during the Beginning Epidemic of COVID-19. J Loss Trauma. 2020;25(6-7):580-93.

1284. Zheng J, Morstead T, Sin N, Klaiber P, Umberson D, Kamble S, et al. Psychological distress in North America during COVID-19: The role of pandemic-related stressors. Soc Sci Med. 2021;270:113687.

1285. Zheng L, Miao M, Gan Y. Perceived Control Buffers the Effects of the COVID-19 Pandemic on General Health and Life Satisfaction: The Mediating Role of Psychological Distance. Appl Psychol Health Well Being. 2020;12(4):1095-114.

1286. Zheng L, Miao M, Lim J, Li M, Nie S, Zhang X. Is lockdown bad for social anxiety in COVID-19 regions?: A national study in the SOR perspective. International Journal of Environmental Research and Public Health. 2020;17(12):1-12.

1287. Zheng YG, Xiao L, Xie YP, Wang HL, Wang GH. Prevalence and Characteristics of Obsessive-Compulsive Disorder Among Urban Residents in Wuhan During the Stage of Regular Control of Coronavirus Disease-19 Epidemic. Frontiers in Psychiatry. 2020;11:594167.

1288. Zhi XX, Lu LJ, Pu YL, Meng AF, Zhao Y, Cheng F, et al. Investigation and analysis of psychological stress and professional identity of nursing students during COVID-19 pandemic. Indian J Exp Biol. 2020;58(6):426-32.

1289. Zhooriyati SM, Crendy TYY, Sammi CSW, Lu X, Lu X. The perception, stress and psychological distress of the corona virus disease (COVID-19) after the outbreak in Malaysia. Annals of the Romanian Society for Cell Biology. 2021;25(3):6707-20.

1290. Zhou JH, Zhang QP. A Survey Study on U.S. College Students' Learning Experience in COVID-19. Educ Sci. 2021;11(5):248-.

1291. Zhou SJ, Wang LL, Yang R, Yang XJ, Zhang LG, Guo ZC, et al. Sleep problems among Chinese adolescents and young adults during the coronavirus-2019 pandemic. Sleep Medicine. 2020;74:39-47.

1292. Zhou Y, MacGeorge EL, Myrick JG. Mental Health and Its Predictors during the Early Months of the COVID-19 Pandemic Experience in the United States. Int J Environ Res Public Health. 2020;17(17):1-19.

1293. Zhou Y, Wade TD. The impact of COVID-19 on body-dissatisfied female university students. Int J Eat Disord. 2021;54(7):1283-8.

1294. Zhu J, Su L, Zhou Y, Qiao J, Hu W. The effect of nationwide quarantine on anxiety levels during the COVID-19 outbreak in China. Brain Behav. 2021;11(1):e01938.

1295. Zhu Y, Wang H, Wang A. An evaluation of mental health and emotion regulation experienced by undergraduate nursing students in China during the COVID-19 pandemic: A cross-sectional study. Int J Ment Health Nurs. 2021;30(5):1160-9.

1296. Zhu Y, Zhang L, Zhou X, Li C, Yang D. The impact of social distancing during COVID-19: A conditional process model of negative emotions, alienation, affective disorders, and post-traumatic stress disorder. J Affect Disord. 2021;281:131-7.

1297. Zhu Y, Zhao SY, Zhou W, Huang P, Hong CJ, Yuan SG, et al. Maybe we are stronger than we thought: Explore protective factors for the public's mental health in COVID-19. Asian Journal of Psychiatry. 2021;57:102555.

1298. Zhu Z, Liu Q, Jiang X, Manandhar U, Luo Z, Zheng X, et al. The psychological status of people affected by the COVID-19 outbreak in China. J Psychiatr Res. 2020;129:1-7.

1299. Znazen H, Slimani M, Bragazzi NL, Tod D. The Relationship between Cognitive Function, Lifestyle Behaviours and Perception of Stress during the COVID-19 Induced Confinement: Insights from Correlational and Mediation Analyses. International Journal of Environmental Research and Public Health. 2021;18(6).

1300. Zubayer AA, Rahman ME, Islam MB, Babu SZD, Rahman QM, Bhuiyan M, et al. Psychological states of Bangladeshi people four months after the COVID-19 pandemic: An online survey. Heliyon. 2020;6(9):e05057.

1301. 昌敬惠, 袁愈新, 王冬. Mental Health Status and Its Influencing Factors Among College Students During the Epidemic of New Coronavirus Pneumonia. 南方医科大学学报. 2020.
[truncated: 16,151 more chars]
